# Improving Interpretation of Cardiac Phenotypes and Enhancing Discovery With Expanded Knowledge in the Gene Ontology

**DOI:** 10.1161/CIRCGEN.117.001813

**Published:** 2018-02-13

**Authors:** Ruth C. Lovering, Paola Roncaglia, Douglas G. Howe, Stanley J.F. Laulederkind, Varsha K. Khodiyar, Tanya Z. Berardini, Susan Tweedie, Rebecca E. Foulger, David Osumi-Sutherland, Nancy H. Campbell, Rachael P. Huntley, Philippa J. Talmud, Judith A. Blake, Ross Breckenridge, Paul R. Riley, Pier D. Lambiase, Perry M. Elliott, Lucie Clapp, Andrew Tinker, David P. Hill

**Affiliations:** From the Institute of Cardiovascular Science (R.C.L., V.K.K., R.E.F., N.H.C., R.P.H., P.J.T., P.D.L., P.M.E., L.C.) and Metabolism and Experimental Therapeutics, Division of Medicine (R.B.), University College London, United Kingdom; European Bioinformatics Institute (EMBL-EBI), European Molecular Biology Laboratory, Hinxton, United Kingdom (P.R., D.O.-S.); Gene Ontology Consortium (P.R., T.Z.B., D.O.-S., J.A.B., D.P.H.); The Zebrafish Model Organism Database, University of Oregon, Eugene (D.G.H.); Rat Genome Database, Human Molecular Genetics Center, Medical College of Wisconsin, Milwaukee (S.J.F.L.); Arabidopsis Information Resource, Phoenix Bioinformatics, Fremont, CA (T.Z.B.); FlyBase, University of Cambridge, United Kingdom (S.T.); Mouse Genome Informatics, The Jackson Laboratory, Bar Harbor, ME (J.A.B., D.P.H.); Oxbridge BHF Centre of Regenerative Medicine, Department of Physiology, Anatomy and Genetics, University of Oxford, United Kingdom (P.R.R.); and William Harvey Heart Centre, Barts and The London School of Medicine and Dentistry, Queen Mary University of London, United Kingdom (A.T.).

**Keywords:** arrhythmias, cardiac, data curation, electrophysiology, gene ontology, genetics, transcriptome

## Abstract

**Background:**

A systems biology approach to cardiac physiology requires a comprehensive representation of how coordinated processes operate in the heart, as well as the ability to interpret relevant transcriptomic and proteomic experiments. The Gene Ontology (GO) Consortium provides structured, controlled vocabularies of biological terms that can be used to summarize and analyze functional knowledge for gene products.

**Methods and Results:**

In this study, we created a computational resource to facilitate genetic studies of cardiac physiology by integrating literature curation with attention to an improved and expanded ontological representation of heart processes in the Gene Ontology. As a result, the Gene Ontology now contains terms that comprehensively describe the roles of proteins in cardiac muscle cell action potential, electrical coupling, and the transmission of the electrical impulse from the sinoatrial node to the ventricles. Evaluating the effectiveness of this approach to inform data analysis demonstrated that Gene Ontology annotations, analyzed within an expanded ontological context of heart processes, can help to identify candidate genes associated with arrhythmic disease risk loci.

**Conclusions:**

We determined that a combination of curation and ontology development for heart-specific genes and processes supports the identification and downstream analysis of genes responsible for the spread of the cardiac action potential through the heart. Annotating these genes and processes in a structured format facilitates data analysis and supports effective retrieval of gene-centric information about cardiac defects.

## Abstract 2

Supplemental Digital Content is available in the text.

**Clinical Perspective**

Identification of critical proteins and RNAs as potential drug targets requires computationally accessible descriptors, both in terms of their roles in biological pathways and the molecules they interact with to fulfill their actions. This information is also vital in choosing prognostic and diagnostic biomarkers, particularly for multifactorial diseases such as heart disease that might benefit from measurement of multiple biomarkers. Recent improvements in omic technologies have led to projects such as the 100 000 Genomes Project and genome-wide association studies, which are producing vast amounts of genetic data pertinent to human health. Furthermore, it is now possible to catalog which proteins or RNAs are present in normal or disease tissues, or individual cells. Understanding the molecular network or biological processes associated with a drug target can help predict off-target effects or the potential for drug repurposing because many gene products are active in multiple pathways. In addition, these networks can be used to predict the most efficacious molecules within the networks, through the identification of key positions at which the whole networks may be perturbed; these molecules are often associated with disease-causing mutations or identified as drug targets. One of the major resources used by omic researchers is the Gene Ontology. This article explains the considerable improvements made by the Gene Ontology Consortium in the bioinformatic description of cardiac physiology. These new descriptions are all based on published data and are now included in all major biological databases, thus available for use by the global scientific community to enhance the understanding of cardiovascular-relevant data.

Cardiac electrical conduction systems enable coordinated regulation of heart contraction in metazoans, ranging from fly to human. Disturbances of normal heart rhythm can occur de novo, but, more critically and commonly, they are an important feature of many cardiac diseases, and have substantial impacts on patient morbidity and mortality. To gain insight into the mechanisms of arrhythmia, high-throughput genome-scale methodologies (including genome-wide association studies [GWAS], transcriptomics, exon sequencing, and proteomics) are being used.^1–3^ However, interpretation of these high-throughput experiments relying on descriptions of the cellular and physiological roles of gene products, and a computational approach to interrogation of cardiac gene function, is a bottleneck in these analyses. Our work aims to fill this gap by capturing information in the Gene Ontology (GO) resource in a structured way, thus integrating knowledge about genes, cells, tissues and organs. To support data interpretation, the GO Consortium (GOC) provides a freely available, structured, controlled vocabulary, the ontology,^4^ that enables association of defined terms, describing cellular roles and locations, with a protein or RNA. This association process, called GO annotation, provides a computer-interpretable summary of the results of many independent experiments. GO terms describe Molecular Functions (molecular activities of a gene product), Biological Processes (the broader context in which a gene product acts), and Cellular Components (the subcellular location of a gene product). Some examples of GO terms relevant to cardiac research are, respectively, *voltage-gated sodium channel activity, Purkinje myocyte action potential*, and *Z disc*.

GO provides users with a summary of experimentally verified or predicted functions of genes, proteins, and RNAs.^3,5^ Because of this high-impact information, GO data are incorporated into over 50 functional analysis tools and is routinely used to analyze large data sets.^6^ The coordinated action of gene products involved in cardiac conduction at the cellular level results in proper heart functioning at the organ level and in healthy conditions at the whole organism level. Therefore, detailed and interoperable knowledge about gene products’ roles is essential to better analyze heart failure phenotypes and ultimately to address potentially fatal conditions. The features listed above make GO an ideal resource to aid the interpretation of large-scale cardiac physiology investigations.

Before our expansion of the cardiac function domain, there were only 3 GO terms to describe how the cardiac cycle is coordinated: *cardiac conduction, membrane depolarization during atrial cardiac muscle cell action potential, and membrane repolarization during atrial cardiac muscle cell action potential*. In this article, we detail how GO editors and biocurators worked together with experimental researchers to ensure provision of accurate structured terminology^7^ that describes features of gene products involved in cardiac physiology, and to generate GO annotations^8^ that capture those roles based on published literature, thereby improving representation of this area of biology. In particular, we focused on processes involved in and regulating the coordinated contraction of the heart. We also show that our effort improves the investigation of heart-related GWAS and transcriptomic data sets.

## Methods

The data, analytic methods, and study materials have been made available to other researchers for purposes of reproducing the results or replicating the procedure. Protein, RNA, and macromolecular complex GO annotations are available from the UniProt GO annotation and GOC sites (https://www.ebi.ac.uk/GOA/downloads, http://www.geneontology.org/gene-associations/) and searchable in the AmiGO2^7^ and QuickGO^9^ browsers; GO terms are downloadable from the GOC site (http://www.geneontology.org/ontology/) and searchable in AmiGO2 and QuickGO. The annotations have also been incorporated into many biological knowledge bases, including Ensembl,^10^ UniProtKB,^11^ and NCBI-Gene,^12^ and exploited by numerous freely available functional analysis tools, including Database for Annotation, Visualization and Integrated Discovery,^13^ g:profiler,^14^ Protein Analysis Through Evolutionary Relationships (PANTHER),^15^ and Visual Annotation Display (VLAD).^16^ Full methods are available in the Data Supplement.

### Generation of a Prioritized List of Cardiac Physiology-Relevant Gene Products

A list of 88 human proteins known to be required for normal cardiac function was compiled based on 8 literature reviews^17–24^ (Table I in the Data Supplement). To keep the focus more specifically on ion channels, the following were not retained in the prioritized list of genes: ATPases, ATPase regulatory proteins, hormones, hormone receptors, and proteins required for normal heart development. However, angiotensinogen and 2 angiotensin I–converting enzymes were included in the prioritized protein list to ensure that the ontology was sufficiently developed to enable future projects to capture the role of gene products that regulate heart rate and contraction.^25^ Orthologs of these 88 human proteins were identified in mouse, rat, zebrafish, and *Drosophila* using the Protein Analysis Through Evolutionary Relationships orthology prediction tool.^15^ In addition, a list of 7 human microRNAs (miRNAs) identified as key players in modulating cardiac excitability at the time we started our curation effort was determined from 3 reviews^26–28^; literature available for these miRNAs was curated up to March 2016. Prioritized human gene products described above are listed in Table I in the Data Supplement, and for all of them, GO annotations supported by experimental data were included in the GO database according to established procedural guidelines^8,29,30^ (details are given in Methods in the Data Supplement).

### Identification of Candidate Genes Associated With Arrhythmia Risk Loci

We compiled a list of Mendelian inherited arrhythmia disorder genes associated with atrial fibrillation, long-QT syndrome, short-QT syndrome, catecholaminergic polymorphic ventricular tachycardia, and Brugada syndrome^1,31,32^ and candidate genes associated with GWAS risk loci for these disorders^2,31^ (literature search February 2016; Table II in the Data Supplement). Nine GO terms relevant to normal cardiac physiology (such as *cardiac conduction, muscle contraction,*and *response to oxygen levels*) were selected to investigate whether gene annotations to these normal processes could be correlated with arrhythmia candidate genes (Table III in the Data Supplement), and the associated annotations were downloaded on March 11, 2016 using the QuickGO browser^9^ (http://www.ebi.ac.uk/QuickGO/; Methods in the Data Supplement).

### GO Functional Analysis of Transcriptomic Data Sets

A ventricular cardiomyocyte (VCM) data set^3^ was analyzed using the BinGO plugin^33^ within the Cytoscape v3.3.0 tool,^34^ applying the recommended hypergeometric test and Benjamini and Hochberg False Discovery Rate correction, and a *P* value <0.05 (full details in Methods in the Data Supplement). To determine the impact of this focused project, we used gene association files from November 2011 (ie, the start of this project) and February 2016 (ie, the end of the project; files available from ftp://ftp.ebi.ac.uk/pub/databases/GO/goa/). For both analyses, the ontology version March 7, 2016 was included (available from http://geneontology.org/page/download-ontology).

## Results

### Representation of Cardiac-Relevant Gene Products’ Features Via Ontology Development

A coordinated working group of GO editors, GO biocurators, and field experts expanded the cardiac physiology domain by adding 197 new cardiac-relevant GO terms (Methods and Table IV in the Data Supplement). Of these, 87 refer to processes that contribute to cardiac conduction and thus describe the propagation of the action potential through the conduction system and cardiac chambers. An example of an ontology branch representing cardiac-relevant GO terms is shown in Figure.

**Figure.**
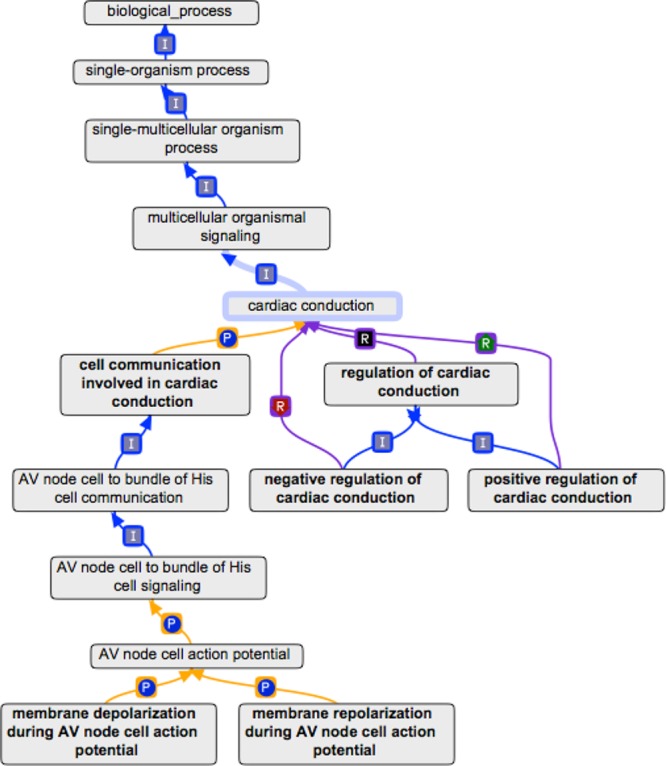
**Part of the ontology describing AV node cell action potential and the regulation of ventricular cardiac muscle cell action potential**. The entire model is not shown; this portion graphically illustrates how ontology terms relate to each other. Blue lines marked with I indicate the child term is a type of its parent, and orange lines marked with P indicate that child term is always a part of its parent. Purple lines marked with R indicate regulates, positively regulates, or negatively regulates relations.

The expansion of the cardiac-related processes in GO describes events that occur at 3 key biological levels: the single cell, the tissue (multicellular), and the organ. In addition to these biological levels, the ontology was expanded to cover generic processes (such as *regulation of potassium ion export*) or developmental processes (such as *cardiac pacemaker cell differentiation*) required for the annotation of cardiac-relevant gene products. These new ontology terms enable biocurators to capture the role of gene products in specific tissues of the heart (by applying GO terms such as *adrenergic receptor signaling pathway involved in cardiac muscle relaxation*), specific cell types (eg, *atrioventricular (AV) node cell to bundle of His cell communication*), and the whole organ (eg, *regulation of the force of heart contraction by cardiac conduction*). The addition of both generic terms, and terms that reference specific anatomical structures, allowed us to overcome challenges with respect to granularity of organism specificity. For example, the term *cell communication involved in cardiac conduction* can be used for any organism with a cardiac organ, and a child term like *AV node cell to bundle of His cell communication* can be used specifically for species that have the relevant anatomical structures, in this case AV node and bundle of His. By describing processes at the various levels, curators can choose appropriate terms with respect to the experiments and organisms they are curating. Where possible, terms were defined by creating necessary and sufficient statements using relationships between GO terms and terms from cross-referenced external ontologies, such as Uberon for anatomical structures, the Cell Ontology for cell types, and Chemicals of Biological Interest for chemicals (ChEBI), taking advantage of their species interoperability and thus making the terms accessible to computational reasoning.^35^

### Cell-to-Cell Communication in the Heart

Cell communication is a key aspect of the coordinated activity of the heart. We created new terms, such as *cell communication involved in cardiac conduction*, to bring together all processes that mediate interactions between a cell and its surroundings and that also contribute to the process of cardiac conduction. This part of the ontology now includes terms to describe cell-to-cell impulse propagation by means of cardiac action potentials and electrical coupling (such as *AV node cell to bundle of His cell communication by electrical coupling*), as well as terms to describe the attachment of one cell to another or to the extracellular matrix (such as *bundle of His cell-Purkinje myocyte adhesion involved in cell communication*). In addition, many of the 22 new cardiac conduction-relevant Molecular Function terms describe channel or transporter activities that are necessary to propagate the electrical signals between specific cells and across the heart (eg, *gap junction channel activity involved in cardiac conduction electric coupling*).

The cardiac action potential branch of GO includes terms referring to a variety of cell types within the heart (Figure), such as *AV node cell action potential*, *membrane depolarization during AV node cell action potential* and *membrane repolarization during AV node cell action potential*, as well as terms that describe the regulation of the action potential (not shown). Similar terms also describe action potentials in SA node cells, bundle of His cells, and Purkinje myocytes (not shown).

### Ensuring Capture of Robust Information About Genetic Contributions to Cardiac Physiology via GO Annotation

There is a considerable volume of literature describing the physiological characterization of the heart. This project brought together expertise from University College London,^36^ Queen Mary University of London, University of Oxford, Mouse Genome Informatics,^37^ the Rat Genome Database,^38^ the Zebrafish Information Network,^39^ and FlyBase.^40^ It was unrealistic for the information from all cardiac physiology research papers to be captured during this project; however, over 500 papers have been reviewed and used to annotate cardiac-relevant gene products with the expanded GO structure. A key aspect of gene product GO annotation is the recognition that a single gene product may have several functions, may participate in multiple processes, and can be found in a variety of subcellular locations. Consequently, fully describing how a gene functions typically results in >1 GO term associated with a single gene product^4^ (Table 1).

**Table 1.**
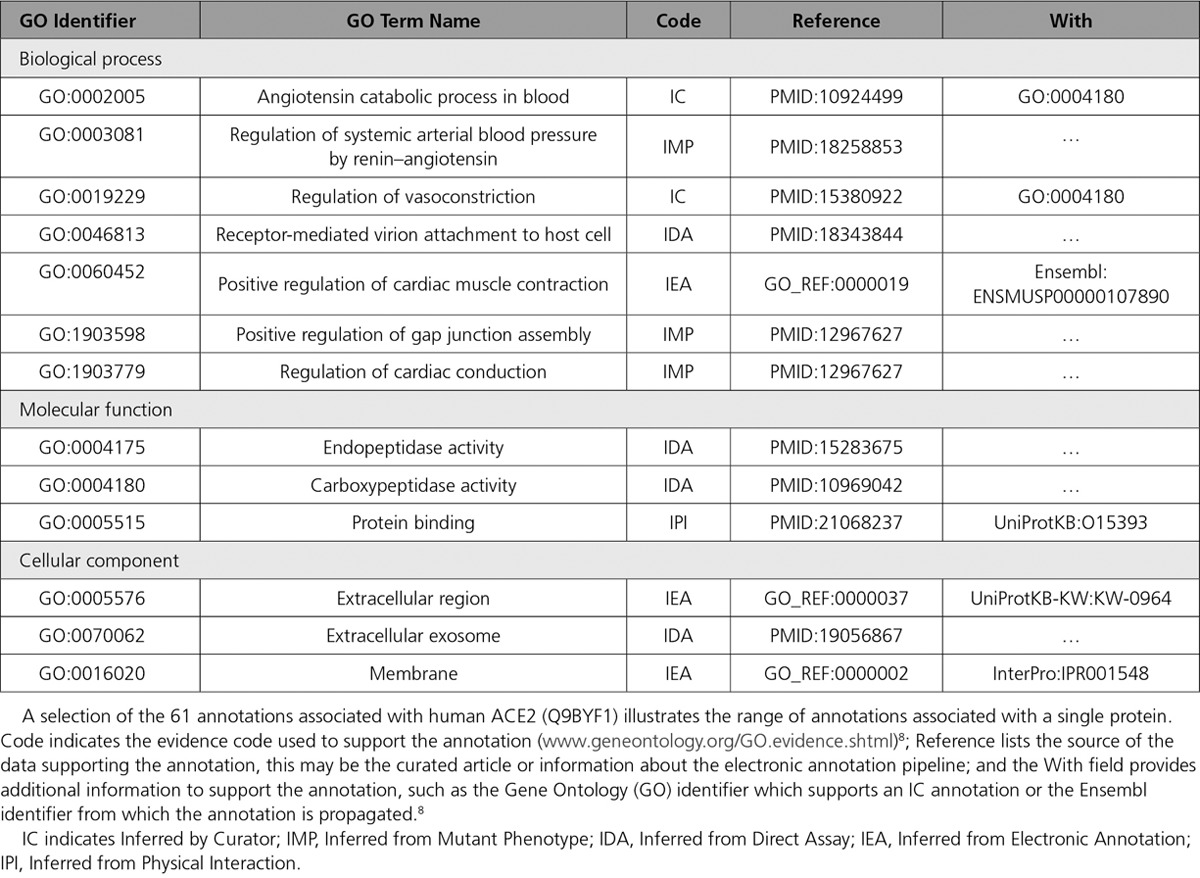
Selection of GO Annotations Associated With Human ACE2

The aim of creating multiple statements (annotations) about a single gene product is to comprehensively capture the knowledge about its biological role, as supported by published experimental data. For example, over 30 papers were curated to provide information about the human sodium channel SCN5A (Q14524). Individual experiments, such as immunostaining, expression of various sodium channel constructs in *Xenopus* oocytes or rat cardiomyocytes, combined with descriptions of cardiac disease associated with the protein, such as long-QT and Brugada syndromes, have provided evidence that this protein is found in the *Z disk*,^41^ has *voltage-gated sodium channel activity involved in cardiac muscle cell action potential*,^42^ and is involved in *membrane depolarization during Purkinje myocyte cell action potential*.^43^ In contrast, the experimental data described in a single paper^44^ supported 9 annotations for the human protein RNF207 (Q6ZRF8). This article also resulted in 18 experimentally supported annotations to 5 other human proteins (DNAJA1, P31689; HSPA1A, P0DMV8; HSPA1B, P0DMV9; HSPA8, P11142; KCNH2, Q12809) and 1 zebrafish protein (Rnf207b, E9QHE3).

There is increasing evidence for the role of miRNAs in controlling cardiac physiology, particularly in the regulation of potassium and calcium ion channel function, where dysregulation can lead to aberrant action potential duration and cardiac conduction.^27,28^ For this project, 7 miRNAs were prioritized for annotation (Methods and Table I in the Data Supplement), resulting in 27 annotations with relevance to cardiac function. For example, Li et al^45^ demonstrated that miR-1 (URS00001DC04F_9606) and miR-133a (URS00004C9052_9606) could regulate the slow delayed rectifier potassium current (*I*_Ks_) in human cells during simulated hyperglycemia. Furthermore, they showed that this was because of their ability to regulate the expression of 2 potassium channel proteins, KCNE1 (P15382) and KCNQ1 (P51787), which mediate *I*_Ks_. These roles of miR-1 and miR-133a have been captured by the terms *negative regulation of membrane repolarization during cardiac muscle cell action potential* and *negative regulation of delayed rectifier potassium channel activity*, as well as *gene silencing by miRNA*, with KCNE1 included as the target of miR-1 and KCNQ1 as the target of miR-133a in annotation extensions (Methods in the Data Supplement).

### The Application of Information Derived From Orthologs

Experimental data to support GO annotations are not always available for human proteins and especially not for miRNAs, but may be obtainable for model organism orthologs. Inferential assertions about the roles of human gene products can be achieved by mapping annotations from their orthologous gene products. The electronic pipeline Ensembl Compara ensures that experimentally supported annotations associated with those proteins that have a 1-to-1 ortholog across human, mouse, and rat are applied to all orthologs.^10^ For example, the predicted 1-to-1 orthology between the mouse ACE2 (Q8R0I0) and human ACE2 (Q9BYF1) proteins as defined by Compara has enabled 3 experimentally supported mouse ACE2 annotations (including *positive regulation of cardiac muscle contraction*) to be associated with human ACE2. The GOC also provides an expert curation tool, called Phylogenetic Annotation and Inference Tool,^46^ that enables biocurators to infer annotations across many species based on phylogenetic relationships and protein family membership. In addition, in some cases where knowledge from Compara and Phylogenetic Annotation and Inference Tool was not available, orthology was reviewed on a case-by-case basis and used to support transfer of annotations to the orthologous proteins^47^ (see next section and Methods in the Data Supplement).

### Comprehensive Capture of Information About Prioritized Gene Products Through Annotation

The manual annotation of the 88 heart-relevant human proteins that were prioritized in this effort (Table I in the Data Supplement) led to the submission of over 3100 annotations to the GO database. This represents a 4-fold increase in the number of manual annotations associated with these proteins. Furthermore, annotation of model organism experimental data has provided a further 2000 annotations to 82 of these proteins through the transfer of experimentally supported annotations to orthologous human proteins via the Ensembl Compara pipeline,^10^ Phylogenetic Annotation and Inference Tool,^46^ and other expert curation methods.^47^ To supplement the orthology-based curation, we manually transferred 50 relevant biological process annotations from mouse or rat to human, using the Inferred From Sequence or Structural Similarity evidence code.^8^ When annotations provided through other electronic pipelines^47^ (such as mappings between UniProtKB Keywords and InterPro) are included, the 88 prioritized human proteins have a total of 7263 annotations (as of March 2017). The number of annotations per protein ranges from 20 associated with RNF207 (Q6ZRF8) to 272 associated with CAV1 (Q03135), with an average of 82 annotations per protein. Over 140 annotations are associated with the 7 prioritized human miRNAs. Therefore, this curation effort allowed bioinformatic capture of knowledge of genes that were not represented before, and it enables more informative data analysis, as shown below.

### Using GO to Interpret GWAS

GWAS have identified many risk loci associated with cardiac disorders.^1,31^ In some cases, the impact of a variant on a protein-coding gene is relatively easy to identify because of predicted, and often experimentally verified, deleterious nonsynonymous substitutions. However, often risk variants identified in a genomic area fall within an intronic, intergenic, or regulatory region, making it difficult to identify the true causative variant(s) associated with the risk, and which protein-coding genes or functional RNA genes should be considered as candidates contributing to a disease.^2,31^ The cost of investigating a candidate gene’s role in a disease is considerable. Consequently, before experimental investigation, various in silico approaches are generally undertaken to try to narrow down the choice of which gene in a gene-rich region is involved in a disease. If nonsynonymous deleterious gene variants in a gene in the same region have already been associated with a similar phenotype, this is good supporting evidence for candidate gene choice. However, other approaches, such as those described by MacArthur et al,^48^ may also be valuable. These include considering whether any of the potential candidate genes encode a protein that interacts with proteins previously implicated in the disease (either using in silico network analysis or through coimmunoprecipitation experiments), or if the gene is expressed in tissues relevant to the disease (eg, by using expression quantitative trait loci data^31^).

MacArthur et al^48^ also describe selecting candidate genes based on a known function or role which is shared with other genes established as associated with the disease of interest or is consistent with their mutant phenotype. This approach can be easily undertaken using GO annotation data, and yet it is rarely included in the in silico investigations of GWAS results. After our focused annotation effort, the ability to use GO annotations to identify candidate genes associated with arrhythmia disorders was evaluated. Human proteins associated with 9 GO terms that are relevant to normal cardiac physiology were downloaded using QuickGO^9^ (GO terms are shown in Table IV in the Data Supplement). The corresponding genes were then compared with Mendelian genes for arrhythmia disorders (atrial fibrillation, long-QT syndrome, short-QT syndrome, catecholaminergic polymorphic ventricular tachycardia, Brugada syndrome^1,31,32^) and with GWAS candidate variants associated with QT interval length and atrial fibrillation risk loci, described by Tucker et al^2^ and Arking et al.^31^ Of 45 Mendelian arrhythmia-associated genes (described in 3 reviews to 2015^1,31,32^), 43 were annotated with ≥1 of the cardiac-relevant GO terms (Table II in the Data Supplement), with *SRL* and *GREM2* being the exceptions. The GO annotations associated with the 83 GWAS-identified candidate genes (associated with 48 risk loci) suggest that GO data can contribute to prioritization of candidate risk genes. Cardiac physiology-relevant GO terms suggest the prioritization of 36 of the 83 candidate genes. This selection approach compares favorably with that taken by Arking et al.^31^ In that study, authors identified 35 common variant QT interval loci and used expression quantitative trait loci, protein interaction, and coimmunoprecipitation data to identify 70 candidate genes associated with these loci (Table II in the Data Supplement). Both the GO and the expression quantitative trait loci approaches suggest 27 QT interval candidate genes, whereas coimmunoprecipitation suggests 12 genes and in silico protein interaction networks 15 genes. For example, GO annotations hint that *RNF207*, at 1p36, is a good candidate for variations in QT interval length, with annotations to 6 of the key GO terms, and that potassium transport-associated *KCNAB2* is a good alternative candidate. In contrast, in silico protein interaction data support 3 other genes at this locus (*ACOT7*, *KCNAB2*, and *RPL22*; Table 2). Similarly, GO annotation information narrows the candidates at the 3p21 locus to 3 genes (*MYL3*, *SCAP*, and *SETD2*; Table 2), with *MYL3* having the strongest annotation support, because of its role in muscle contraction and heart development; the analysis provided by Arking et al^31^ suggested 5 candidate genes at this locus. Notably, at the 1q24 risk locus, GO annotations, expression quantitative trait loci, and coimmunoprecipitation data all suggest *ATP1B1* as the candidate gene (Table 2). This illustrates that GO annotations can provide additional data to either support the choice of candidate genes or suggest alternative candidates.

**Table 2.**
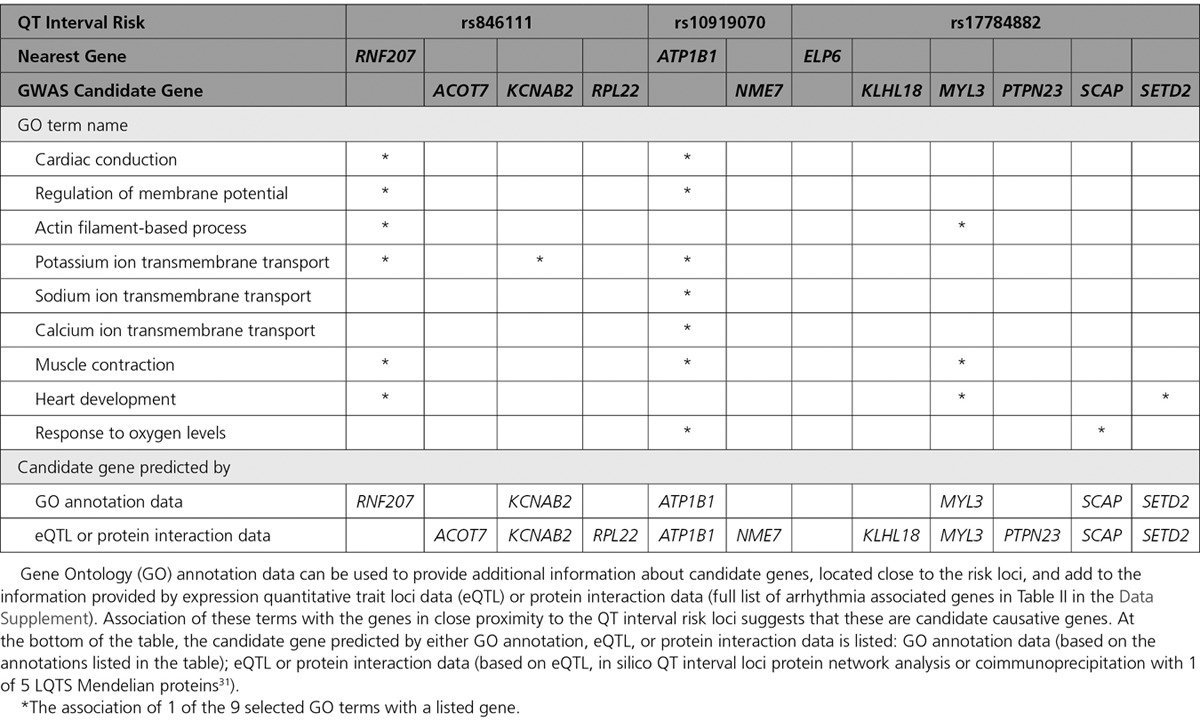
Using GO to Support the Identification of the Likely Causative Gene Associated With 3 QT Interval Risk Loci31

### Functional Analysis of Atrial and Ventricular High-Throughput Data Sets

To provide a measure of the impact of our cardiac-focused annotation project on data interpretation, we compared the functional analysis of a specific heart transcriptomic data set using GO annotations available in November 2016 to an equivalent analysis using GO annotation data available in February 2011. A VCM transcriptomic data set^3^ was used to investigate this, as we anticipated that a ventricular-specific transcriptome was likely to include the ion channels, and their regulators, required for cardiac repolarization and depolarization. Poon et al^3^ profiled the transcriptome of human embryonic stem cells and compared this to the transcriptome of adult human VCMs, after filtering out the transcripts that were not significantly differentially expressed between the 2 cell types.^3^ Poon et al^3^ found that GO terms describing translation elongation, as well as muscle system, contraction, and energy generation (their wording), were enriched within the 200 most abundant transcripts in adult human VCMs. Our analysis using the 2011 and 2016 GO annotation data sets confirms findings of Poon et al,^3^ with the majority of these proteins annotated to either *muscle contraction, mitochondrion organization, respiratory electron transport chain, gene expression, or developmental process* terms. Notably, our analysis also demonstrates that the terms *cardiac conduction, regulation of cardiac conduction,* and *regulation of the force of heart contraction* (among others) were significantly enriched in the 2016 analysis. Although VCMs are not involved in cardiac conduction, it is not unexpected that *cardiac conduction* and child terms will be enriched in a ventricular data set, because many of the same action potential-associated proteins are present in ventricular, atrial, and conduction tissues.^23^ The identification of the cardiac-specific terms within this data set does, however, confirm that the GO terms and annotations created by this focused project are sufficient to enable the identification of the increased expression of genes involved in these processes. In 2011, many of the cardiac physiology GO terms did not exist, and consequently, these terms are (understandably) not enriched using the 2011 annotation data set (Table 3; Table V in the Data Supplement).

**Table 3.**
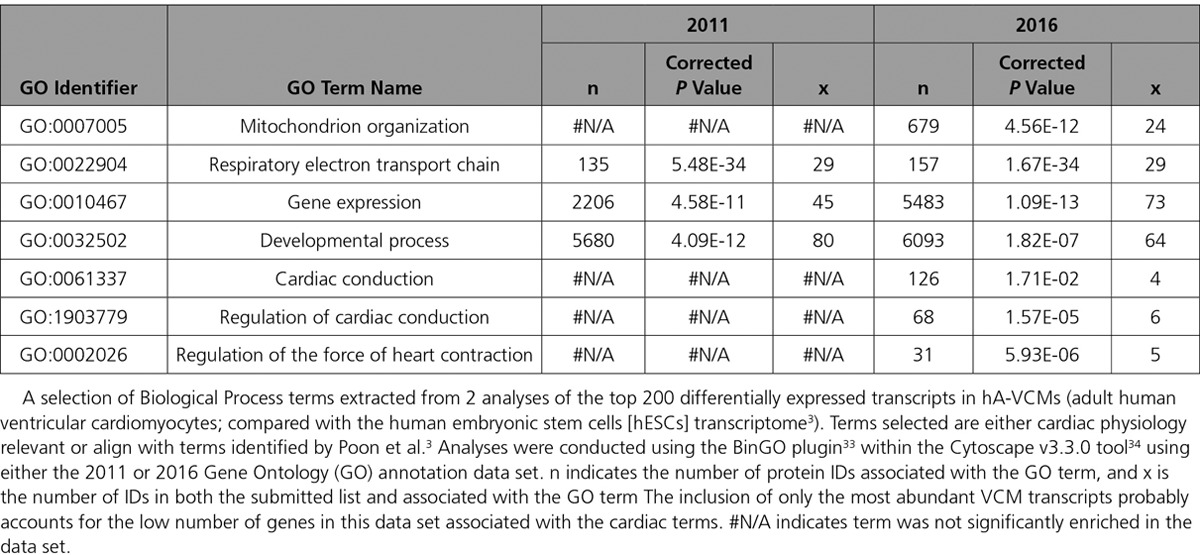
Comparison of Functional Analysis of Adult Ventricular Cardiomyocyte Data3 Using Gene Ontology (Full List of Enriched Terms in Table V in the Data Supplement)

## Discussion

In this article, we describe work to improve the GO resource in representing relevant cardiac processes, and in capturing roles of gene products from published literature through GO annotation. The majority of publications describing heart or cardiomyocyte transcriptomic and proteomic data sets use GO annotations to investigate the pathways associated with this important organ.^3,5^ We tested whether our recent cardiac-relevant annotations could be used to support a more informative interpretation of high-throughput studies. Our reanalysis of the top 200 differentially expressed transcripts in adult human VCMs^3^ (compared with the human embryonic stem cells transcriptome; Table V in the Data Supplement) confirms the original findings of Poon et al^3^ and, in addition, shows that the transcriptome of these cells is enriched for GO terms referring to cardiac physiology, including *cardiac conduction, regulation of cardiac conduction, regulation of cardiac muscle contraction by calcium ion signaling,* and *regulation of the force of heart contraction*. Notably, the enrichment of these terms is only possible because of the expansion of the GO resource in this area. These cardiac-relevant terms are identified despite more than half of these highly expressed proteins being associated with protein synthesis, mitochondrial respiratory systems, or having a structural cellular role. The significance of our work lies in the enhanced ability to identify cardiac-relevant genes within cardiac tissue; cardiac physiologists can now use the GO to test genomic profiles and confirm the presence of relevant genes. For example, researchers who are using in vitro cardiac cell differentiation systems can now test the profiles of gene expression in their cells to examine and confirm progression toward mature cardiomyocytes. This has potential application in the field of heart cell regeneration.

Furthermore, our analysis of QT interval length and atrial fibrillation risk loci, described by Tucker et al^2^ and Arking et al,^31^ demonstrates that our focused GO annotation of cardiac physiological processes provides an enhanced GO resource that can be used, in combination with other experimental and in silico approaches, to suggest candidate genes associated with cardiac disease. In particular, our results suggest that *ATP1B1*, a Na^+^/K^+^-ATPase β-subunit, is the most likely candidate gene for further study at the 1q24 risk locus because it is associated with cardiac-relevant GO annotations. Another potential use of cardiac GO annotations is to extend protein network analysis. Overlaying proteins with relevant annotations (and hence known cardiac role) onto an existing network would highlight their interacting partners, and these could be explored as candidate genes.

With researchers turning to *Drosophila* and zebrafish as model systems for heart disease,^49,50^ there is a requirement for GO to represent cardiac physiology using terms that are species independent.^6^ The *Drosophila* pulsatile heart tube, with its anterior aorta, looks structurally very distinct from the zebrafish 2-chambered and the mammalian 4-chambered hearts. However, despite this, many of the new GO terms created during this project can be applied across all species because many orthologous genes have similar functions. The major limiting factor to the GO annotation of nonmammalian systems is the paucity of research describing the roles of individual gene products in the cardiac cycle in those species, as the majority of these data relate to mammals. Indeed, the curation effort described in this article focuses on improving analysis tools for human cardiovascular research.

Our results show that collaboration between members of relevant model organism databases and focused curation groups can improve and extend the capture of information about physiological processes in GO, in this particular study, cardiac processes. This effort was enhanced by the collaboration with experts in the cardiac research field who helped us identify key aspects of the process, key genes, and key publications on which to focus. As knowledge of the cardiac system continues to advance, there will be opportunities to capture additional cardiac information in GO. We have provided a structured framework for the addition of new knowledge to the resource, for example, to better describe information about the proteins involved in the mechanical aspect of the heart, and to fully capture roles of gene products and hormones in regulating cardiac physiology. Our work also shows that collaboration between groups developing ontologies and creating biological annotations and scientists and clinicians engaged in active research can lead to substantial improvements in the computational representation of biological knowledge. The GOC welcomes input from researchers about any aspect of our work, including changes to the ontology and suggestions of papers and gene products to annotate (goannotation@ucl.ac.uk, http://geneontology.org/page/contributing-go). A variety of GitHub repositories with issue trackers are in place for specific queries: for general inquiries about GO (https://github.com/geneontology/helpdesk), for specific questions about annotations or annotation-related topics (https://github.com/geneontology/go-annotation/), and for specific questions or suggestions about the content and structure of the ontology (https://github.com/geneontology/go-ontology/). Such collaborations improve the value of the GO resource for the benefit of the entire cardiovascular research community and facilitate the interpretation of high-throughput data sets, toward the identification of dysregulated cardiac pathways, as well as variants and risk alleles, associated with cardiovascular diseases. This has important implications for cardiovascular physicians needing to interpret potentially pathological variants in their patients as a resource for the most up-to-date bioinformatic data to inform diagnosis and cascade screening in families.

## Acknowledgments

We are extremely grateful to all members of the Gene Ontology (GO) Consortium who contribute to annotation and ontology development on an ongoing basis.

## Sources of Funding

This work was funded through grants from the British Heart Foundation (BHF, SP/07/007/23671, RG/13/5/30112) and the National Institute for Health Research University College London Hospitals Biomedical Research Centre; The Zebrafish Model Organism Database: National Human Genome Research Institute (NHGRI, HG002659, HG004838, HG004834); The Rat Genome Database: National Heart, Lung, and Blood Institute on behalf of the NIH (HL64541); The Mouse Genome Database: NGHRI (HG003300); FlyBase: UK Medical Research Council (G1000968); and Gene Ontology Consortium: NIH NHGRI (U41 HG002273) to Drs Blake, Cherry, Lewis, Sternberg, and Thomas. Professor Riley received BHF personal chair award (CH/11/1/28798). Professors Lambiase and Tinker received support from BHF and UK Medical Research Council. Professor Tinker received National Institute for Health Research Biomedical Research Centre at Barts and BHF grant (RG/15/15/31742). Dr Roncaglia received EMBL-EBI Core funds.

## Disclosures

None.

## Supplementary Material
