# Supplementary material for: Improving Interpretation of Cardiac Phenotypes and Enhancing Discovery With Expanded Knowledge in the Gene Ontology
Source: Circ Genom Precis Med. 2018 Feb 13;11(2):e001813. doi: 10.1161/CIRCGEN.117.001813 (PMC5821137; doi:10.1161/CIRCGEN.117.001813)
Supplement: Supplementary file 1 [file hcg-11-e001813-s001.pdf]

SUPPLEMENTAL MATERIAL

Methods

Ontology engineering

GO provides more than a simple flat list of terms. Instead, the terms have defined relations between each other, which can be represented in a graphical view (see Figure 1, which is a view of the ontology created with the OBO-Edit tool Graph Editor function using the public OBO release of GO on April 26, 2017, http://purl.obolibrary.org/obo/go.obo).[1] Each GO term can have multiple, more general, 'parent' terms and multiple, more specific, 'child' terms. Different relations are available to define the connection between two GO terms[2]. Five core relations have been applied to the cardiac physiology part of the ontology: is_a, part_of, regulates, positively_regulates and negatively_regulates. The is_a relation denotes that the child term has all the characteristics of its parent but is a more specific term, with differences that distinguish it from other is_a children, for example 'AV node cell to bundle of His cell communication' is a subtype of 'cell communication involved in cardiac conduction' and is distinguished by the cells that are communicating. The part_of relation denotes that the child term is always a part of the parent term and together with additional parts make up the parent. For example, 'AV node cell action potential' is part of the process of 'AV node cell to bundle of His cell signaling' because it is required to propagate the electrical signal along the AV node cell. Another example is that GO Molecular Function terms describing channel activities required for the changes in membrane potential that are associated with an action potential have 'part_of' relationships with the relevant membrane depolarization and repolarization terms. Coupling the channel activities with the processes that regulate membrane potential in these sections of the ontology provides a good example of how the Molecular Function and Biological Process branches can be integrated. The various types of regulation relations describe when a process has a regulatory or controlling effect on another process, for example 'regulation of cardiac conduction' describes a type of process that has a regulatory effect on 'cardiac conduction'. The development of the ontology to describe cardiac-related processes involved more than placing the new GO terms in the ontology. Each GO term was assigned a unique GO identifier (ID), a descriptive definition and synonyms used in the literature. Synonyms and definitions facilitate the curation of a gene product's role by allowing easier retrieval of appropriate GO terms when searching the ontology. They also provide a clear, human-readable meaning of the term. Additionally, where applicable, GO terms are defined computationally by creating necessary and sufficient statements using relationships between GO terms and terms from cross-referenced external ontologies[3,4].

Expert curation using Gene Ontology

To create GO annotations supported by experimental data, expert GO curators read publications and summarised the data in annotations[5]. There is a vast number of publications describing the function of the gene products we have prioritized, therefore only a fraction of the available literature was curated. Because the curators are based at different institutes, different strategies were taken to identify the appropriate papers to annotate[6,7]. Since the focus of this project was to capture the role of specific gene products in cardiac physiology, the papers selected usually contained experimental data describing the prioritized proteins and their role in cardiac systems.

Whenever possible, annotations also specify additional contextual information, such as the location of a function or process in a 'regular ventricular cardiac myocyte' or 'heart left ventricle', by using

annotation extensions[4] that refer to external ontologies such as the Cell Ontology (CL)[8-10] or the Uberon multi-species anatomy ontology[11,12]. For example, human SCN5A is associated with the GO term 'Z disc' with the annotation extension 'part_of' 'cardiac muscle cell'[4]. Thus, this annotation specifies the cell, as well as the cellular, location of this protein.

Each annotation includes the gene product identifier, a GO term, an evidence code, and a reference[2]. The QuickGO browser (http://www.ebi.ac.uk/QuickGO/)[13] or the AmiGO2 browser (http://amigo.geneontology.org/amigo/search/ontology)[14] were used to search for the most specific GO terms to 'capture' the experimental data presented in each paper. Appropriate evidence codes were given to each annotation based on the type of experimental data presented in the published papers (www.geneontology.org/GO.evidence.shtml)[15]. The evidence code 'Inferred by Curator' (IC) was applied when a combination of experimental data from more than one paper supported the annotation. To ensure full annotation of the human, mouse and rat proteomes, experimentally supported annotations associated with model organism and human gene products are automatically transferred to the orthologous mammalian gene products via the Ensembl Compara pipeline[16]. However, 13 of the 88 prioritized proteins are members of highly conserved protein families and therefore do not have 1-to-1 orthologs in human, mouse and rat, as defined by Ensembl Compara. In these cases, GO annotations with experimental evidence codes were associated to orthologous human, mouse and rat proteins, by the expert curators, using the evidence code ISS (Inferred from Sequence Similarity), or ISO (Inferred from Orthology) based on confirmation of predicted orthology by the HUGO Gene Nomenclature Committee (http://www.genenames.org/) ortholog prediction tool (HCOP)[17]. The procedure for curation of miRNAs is detailed in Huntley et al. 2016[5].

Identifying experimental data to annotate

The PubMed database[18] was used to locate recent papers reviewing the literature for cardiac electrophysiology and channelopathies. Gene products with a known role in the cardiac cycle were identified from these reviews. PubMed searches, with each individual gene symbol, name or synonym and additional filters, were conducted to provide a comprehensive coverage of the role of these proteins with respect to the cardiac physiology. These searches included specific filters, in addition to the relevant gene-symbol and name: 'AND cardiac, 'AND heart, or 'AND conduction'. The selection of papers to curate was then based on whether: 1) they contained experimental data; 2) new information would be added to the current GO annotation data associated with the protein; 3) it was possible to identify the species the protein or expression construct was derived from. Only papers that met all three criteria were curated. The choice of papers curated was, therefore, influenced by the information captured previously (i.e. papers already annotated with existing cardiac terms were excluded). While human gene products were the main focus of this annotation project, other species gene products were also curated where information was available.

Identification of candidate genes associated with arrhythmia risk loci

The QuickGO browser[13], with 'taxon' and 'GO term' filters applied, was used to download all human proteins associated with nine GO terms that are relevant to normal cardiac physiology (these nine GO terms are listed in Supplemental Table 4). The Microsoft Excel VLOOKUP function was used to identify the Mendelian genes and the candidate genes (listed in Supplemental Table 3) that were associated with the selected GO terms.

GO functional analysis of transcriptomic datasets

A ventricular cardiomyocyte dataset[19] was analysed using the BinGO plugin[20] within the Cytoscape v3.3.0 tool[21], according to the tools recommended procedure. BinGO has the facility to choose which GO annotation dataset to include in the analysis, allowing 2011 and 2016 annotation datasets to be compared. The ontology go-basic.obo (dated 7/03/2016) was downloaded from the GO Consortium website (http://geneontology.org/page/download-ontology), and the gene association files (containing the annotation datasets) gene_association.goa_human.104.gz (dated 14/11/2011) and gene_association.goa_human.154.gz (dated 16/02/2016), were downloaded from ftp://ftp.ebi.ac.uk/pub/databases/GO/goa/old/HUMAN/ and ftp://ftp.ebi.ac.uk/pub/databases/GO/goa/HUMAN/ respectively. For the analysis UniProtKB accessions were submitted with the following settings: assess overrepresentation; hypergeometric test; Benjamini & Hochberg False Discovery Rate correction; $p<0.05$ significance level; 'whole annotation' was used as reference set, leading to a comparison of the GO terms associated with the ventricular cardiomyocyte dataset[19] with the GO terms associated with the human proteome.

Supplemental Tables

Supplemental Table S1. 88 Cardiac physiology gene products prioritized for annotation. Human proteins with a role in cardiac excitability were compiled based on the description of proteins listed in eleven cardiac physiology reviews[22-32]. This list was then filtered to remove hormones and hormone receptors involved in regulating the heart rate (with the exception of AGT, ACE and ACE2) and proteins involved in regulating heart development. This enabled us to focus more specifically on ion channels and ATPases and the proteins directly regulating their activity.

Approved symbol	Approved name	UniProt ID/RNA central ID	Link to QuickGO or AmiGO annotation record
ACE	angiotensin I converting enzyme	P12821	http://www.ebi.ac.uk/QuickGO/GProtein?ac=P12821
ACE2	angiotensin I converting enzyme 2	Q9BYF1	http://www.ebi.ac.uk/QuickGO/GProtein?ac=Q9BYF1
AGT	angiotensinogen	P01019	http://www.ebi.ac.uk/QuickGO/GProtein?ac=P01019
AKAP6	A-kinase anchoring protein 6	Q13023	http://www.ebi.ac.uk/QuickGO/GProtein?ac=Q13023
AKAP9	A-kinase anchoring protein 9	Q99996	http://www.ebi.ac.uk/QuickGO/GProtein?ac=Q99996
ANK2	ankyrin 2	Q01484	http://www.ebi.ac.uk/QuickGO/GProtein?ac=Q01484
ANK3	ankyrin 3	Q12955	http://www.ebi.ac.uk/QuickGO/GProtein?ac=Q12955
ASPH	aspartate beta-hydroxylase	Q12797	http://www.ebi.ac.uk/QuickGO/GProtein?ac=Q12797
ATP1A1	ATPase Na+/K+ transporting subunit alpha 1	P05023	http://www.ebi.ac.uk/QuickGO/GProtein?ac=P05023
ATP1A2	ATPase Na+/K+ transporting subunit alpha 2	P50993	http://www.ebi.ac.uk/QuickGO/GProtein?ac=P50993
ATP1A3	ATPase Na+/K+ transporting subunit alpha 3	P13637	http://www.ebi.ac.uk/QuickGO/GProtein?ac=P13637
ATP1B1	ATPase Na+/K+ transporting subunit beta 1	P05026	http://www.ebi.ac.uk/QuickGO/GProtein?ac=P05026
ATP2A2	ATPase sarcoplasmic/endoplasmic reticulum Ca2+ transporting 2	P16615	http://www.ebi.ac.uk/QuickGO/GProtein?ac=P16615
ATP2B4	ATPase plasma membrane Ca2+ transporting 4	P23634	http://www.ebi.ac.uk/QuickGO/GProtein?ac=P23634
CACNA1C	calcium voltage-gated channel subunit alpha1 C	Q13936	http://www.ebi.ac.uk/QuickGO/GProtein?ac=Q13936
CACNA1D	calcium voltage-gated channel subunit alpha1 D	Q01668	http://www.ebi.ac.uk/QuickGO/GProtein?ac=Q01668
CACNA1G	calcium voltage-gated channel subunit alpha1 G	O43497	http://www.ebi.ac.uk/QuickGO/GProtein?ac=O43497
CACNA2D1	calcium voltage-gated channel auxiliary subunit alpha2delta 1	P54289	http://www.ebi.ac.uk/QuickGO/GProtein?ac=P54289
CACNB2	calcium voltage-gated channel auxiliary subunit beta 2	Q08289	http://www.ebi.ac.uk/QuickGO/GProtein?ac=Q08289
CALM1	calmodulin 1	P62158	http://www.ebi.ac.uk/QuickGO/GProtein?ac=P62158
CAMK2D	calcium/calmodulin dependent protein kinase II delta	Q13557	http://www.ebi.ac.uk/QuickGO/GProtein?ac=Q13557
CASQ2	calsequestrin 2	O14958	http://www.ebi.ac.uk/QuickGO/GProtein?ac=O14958
CAV1	caveolin 1	Q03135	http://www.ebi.ac.uk/QuickGO/GProtein?ac=Q03135
CAV2	caveolin 2	P51636	http://www.ebi.ac.uk/QuickGO/GProtein?ac=P51636
CAV3	caveolin 3	P56539	http://www.ebi.ac.uk/QuickGO/GProtein?ac=P56539
CLIC2	chloride intracellular channel 2	O15247	http://www.ebi.ac.uk/QuickGO/GProtein?ac=O15247
CNOT1	CCR4-NOT transcription complex subunit 1	A5YKK6	http://www.ebi.ac.uk/QuickGO/GProtein?ac=A5YKK6
DLG1	discs large MAGUK scaffold protein 1	Q12959	http://www.ebi.ac.uk/QuickGO/GProtein?ac=Q12959
DMD	dystrophin	P11532	http://www.ebi.ac.uk/QuickGO/GProtein?ac=P11532
DSG2	desmoglein 2	Q14126	http://www.ebi.ac.uk/QuickGO/GProtein?ac=Q14126
FGF12	fibroblast growth factor 12	P61328	http://www.ebi.ac.uk/QuickGO/GProtein?ac=P61328
FGF13	fibroblast growth factor 13	Q92913	http://www.ebi.ac.uk/QuickGO/GProtein?ac=Q92913
FKBP1B	FK506 binding protein 1B	P68106	http://www.ebi.ac.uk/QuickGO/GProtein?ac=P68106
FLNA	filamin A	P21333	http://www.ebi.ac.uk/QuickGO/GProtein?ac=P21333
FXYD1	FXYD domain containing ion transport regulator 1	O00168	http://www.ebi.ac.uk/QuickGO/GProtein?ac=O00168
GJA1	gap junction protein alpha 1	P17302	http://www.ebi.ac.uk/QuickGO/GProtein?ac=P17302
GJA5	gap junction protein alpha 5	P36382	http://www.ebi.ac.uk/QuickGO/GProtein?ac=P36382
GPD1L	glycerol-3-phosphate dehydrogenase 1 like	Q8N335	http://www.ebi.ac.uk/QuickGO/GProtein?ac=Q8N335
GSTM2	glutathione S-transferase mu 2	P28161	http://www.ebi.ac.uk/QuickGO/GProtein?ac=P28161
HCN4	hyperpolarization activated cyclic nucleotide gated potassium channel 4	Q9Y3Q4	http://www.ebi.ac.uk/QuickGO/GProtein?ac=Q9Y3Q4
HRC	histidine rich calcium binding protein	P23327	http://www.ebi.ac.uk/QuickGO/GProtein?ac=P23327
KCNA5	potassium voltage-gated channel subfamily A member 5	P22460	http://www.ebi.ac.uk/QuickGO/GProtein?ac=P22460
KCND3	potassium voltage-gated channel subfamily D member 3	Q9UK17	http://www.ebi.ac.uk/QuickGO/GProtein?ac=Q9UK17
KCNE1	potassium voltage-gated channel subfamily E regulatory subunit 1	P15382	http://www.ebi.ac.uk/QuickGO/GProtein?ac=P15382
KCNE2	potassium voltage-gated channel subfamily E regulatory subunit 2	Q9Y6J6	http://www.ebi.ac.uk/QuickGO/GProtein?ac=Q9Y6J6
KCNE3	potassium voltage-gated channel subfamily E regulatory subunit 3	Q9Y6H6	http://www.ebi.ac.uk/QuickGO/GProtein?ac=Q9Y6H6
KCNE5	potassium voltage-gated channel subfamily E regulatory subunit 5	Q9UJ90	http://www.ebi.ac.uk/QuickGO/GProtein?ac=Q9UJ90
KCNH2	potassium voltage-gated channel subfamily H member 2	Q12809	http://www.ebi.ac.uk/QuickGO/GProtein?ac=Q12809
KCNJ2	potassium voltage-gated channel subfamily J member 2	P63252	http://www.ebi.ac.uk/QuickGO/GProtein?ac=P63252
KCNJ5	potassium voltage-gated channel subfamily J member 5	P48544	http://www.ebi.ac.uk/QuickGO/GProtein?ac=P48544
KCNJ8	potassium voltage-gated channel subfamily J member 8	Q15842	http://www.ebi.ac.uk/QuickGO/GProtein?ac=Q15842
KCNQ1	potassium voltage-gated channel subfamily Q member 1	P51787	http://www.ebi.ac.uk/QuickGO/GProtein?ac=P51787
LIG3	DNA ligase 3	P49916	http://www.ebi.ac.uk/QuickGO/GProtein?ac=P49916
LITAF	lipopolysaccharide induced TNF factor	Q99732	http://www.ebi.ac.uk/QuickGO/GProtein?ac=Q99732
NDRG4	NDRG family member 4	Q9ULP0	http://www.ebi.ac.uk/QuickGO/GProtein?ac=Q9ULP0
NEDD4L	neural precursor cell expressed, developmentally down-regulated 4-like, E3 ubiquitin protein ligase	Q96PU5	http://www.ebi.ac.uk/QuickGO/GProtein?ac=Q96PU5
NOS1	nitric oxide synthase 1	P29475	http://www.ebi.ac.uk/QuickGO/GProtein?ac=P29475
NOS1AP	nitric oxide synthase 1 adaptor protein	O75052	http://www.ebi.ac.uk/QuickGO/GProtein?ac=O75052
NPPA	natriuretic peptide A	P01160	http://www.ebi.ac.uk/QuickGO/GProtein?ac=P01160
NUP155	nucleoporin 155	O75694	http://www.ebi.ac.uk/QuickGO/GProtein?ac=O75694
PDE4B	phosphodiesterase 4B	Q07343	http://www.ebi.ac.uk/QuickGO/GProtein?ac=Q07343
PDE4D	phosphodiesterase 4D	Q08499	http://www.ebi.ac.uk/QuickGO/GProtein?ac=Q08499
PKP2	plakophilin 2	Q99959	http://www.ebi.ac.uk/QuickGO/GProtein?ac=Q99959
PLN	phospholamban	P26678	http://www.ebi.ac.uk/QuickGO/GProtein?ac=P26678
PRKACA	protein kinase cAMP-activated catalytic subunit alpha	P17612	http://www.ebi.ac.uk/QuickGO/GProtein?ac=P17612
PTPN3	protein tyrosine phosphatase, non-receptor type 3	P26045	http://www.ebi.ac.uk/QuickGO/GProtein?ac=P26045
RANGRF	RAN guanine nucleotide release factor	Q9HD47	http://www.ebi.ac.uk/QuickGO/GProtein?ac=Q9HD47
RNF207	ring finger protein 207	Q6ZRF8	http://www.ebi.ac.uk/QuickGO/GProtein?ac=Q6ZRF8
RYR2	ryanodine receptor 2	Q92736	http://www.ebi.ac.uk/QuickGO/GProtein?ac=Q92736
SCN10A	sodium voltage-gated channel alpha subunit 10	Q9Y5Y9	http://www.ebi.ac.uk/QuickGO/GProtein?ac=Q9Y5Y9
SCN1B	sodium voltage-gated channel beta subunit 1	Q07699	http://www.ebi.ac.uk/QuickGO/GProtein?ac=Q07699
SCN2B	sodium voltage-gated channel beta subunit 2	O60939	http://www.ebi.ac.uk/QuickGO/GProtein?ac=O60939
SCN3B	sodium voltage-gated channel beta subunit 3	Q9NY72	http://www.ebi.ac.uk/QuickGO/GProtein?ac=Q9NY72
SCN4B	sodium voltage-gated channel beta subunit 4	Q8IWT1	http://www.ebi.ac.uk/QuickGO/GProtein?ac=Q8IWT1
SCN5A	sodium voltage-gated channel alpha subunit 5	Q14524	http://www.ebi.ac.uk/QuickGO/GProtein?ac=Q14524
SLC8A1	solute carrier family 8 member A1	P32418	http://www.ebi.ac.uk/QuickGO/GProtein?ac=P32418
SLC8B1	solute carrier family 8 member B1	Q6J4K2	http://www.ebi.ac.uk/QuickGO/GProtein?ac=Q6J4K2
SLC9A1	solute carrier family 9 member A1	P19634	http://www.ebi.ac.uk/QuickGO/GProtein?ac=P19634
SLMAP	sarcolemma associated protein	Q14BN4	http://www.ebi.ac.uk/QuickGO/GProtein?ac=Q14BN4
SNTA1	syntrophin alpha 1	Q13424	http://www.ebi.ac.uk/QuickGO/GProtein?ac=Q13424
SPTBN4	spectrin beta, non-erythrocytic 4	Q9H254	http://www.ebi.ac.uk/QuickGO/GProtein?ac=Q9H254
SRI	sorcin	P30626	http://www.ebi.ac.uk/QuickGO/GProtein?ac=P30626
TCAP	titin-cap	O15273	http://www.ebi.ac.uk/QuickGO/GProtein?ac=O15273
TRDN	triadin	Q13061	http://www.ebi.ac.uk/QuickGO/GProtein?ac=Q13061
TRPM4	transient receptor potential cation channel subfamily M member 4	Q8TD43	http://www.ebi.ac.uk/QuickGO/GProtein?ac=Q8TD43
YWHAE	tyrosine 3-monooxygenase/tryptophan 5-monooxygenase activation protein epsilon	P62258	http://www.ebi.ac.uk/QuickGO/GProtein?ac=P62258
YWHAH	tyrosine 3-monooxygenase/tryptophan 5-monooxygenase activation protein eta	Q04917	http://www.ebi.ac.uk/QuickGO/GProtein?ac=Q04917
hsa-miR-1-3p	miR-1	URS00001DC04F_9606	http://amigo.geneontology.org/amigo/gene_product/RNAcentral:URS00001DC04F_9606
hsa-miR-26a-5p	miR-26	URS000019B0F7_9606	http://amigo.geneontology.org/amigo/gene_product/RNAcentral:URS000019B0F7_9606
hsa-miR-133a-3p	miR-133a	URS00004C9052_9606	http://amigo.geneontology.org/amigo/gene_product/RNAcentral:URS00004C9052_9606
hsa-miR-208a-3p	miR-208a	URS00000E5433_9606	http://amigo.geneontology.org/amigo/gene_product/RNAcentral:URS00000E5433_9606
hsa-miR-212-3p	miR-212	URS00001D6BAE_9606	http://amigo.geneontology.org/amigo/gene_product/RNAcentral:URS00001D6BAE_9606
hsa-miR-328-3p	miR-328	URS00005FDE70_9606	http://amigo.geneontology.org/amigo/gene_product/RNAcentral:URS00005FDE70_9606
hsa-miR-499-5p	miR-499	URS00000C7662_9606	http://amigo.geneontology.org/amigo/gene_product/RNAcentral:URS00000C7662_9606

Supplemental Table S2. Using Gene Ontology to support the identification of the likely causative gene associated with QT interval length and AF risk loci. 117 genes identified as either a known Mendelian gene associated with AF, LQTS, SQTS, CPVT or BrS[33,34], or a candidate gene associated with AF risk loci or loci associated with QT interval length, by GWAS[33,35,36]. Nine GO terms were selected based on their potential relevance to cardiac physiology (Supplemental Table S3). QuickGO[13] was used to download all human proteins associated with these terms either directly or to a child term with one of the following relations: part_of, is_a, regulates, negatively_regulates, positively_regulates (11 March 2016). The association of these 9 GO terms with each of the AF or QT interval-associated genes was then determined, indicated by solid filled cell. Candidate genes identified by annotation to a relevant GO term were then compared with arrhythmia disorder Mendelian genes and candidate genes identified using eQTL, *in silico* QT interval loci protein network analysis (PPI-QT) or co-immunoprecipitation with one of 5 LQTS Mendelian proteins (CoIP)[33]. ELP6 is the approved symbol for C3orf75.

GWAS: QT interval - nearest gene or locus Arking 2014	Genetic variant or nearest gene Arking 2014, Spears 2015, Tucker 2014, 2016	GWAS identified gene Arking 2014	HGNC approved gene name	UniProt ID	GWAS: QT interval - SNP Arking 2014	GWAS: Atrial Fibrullation - SNP Tucker 2016	SNP location	cardiac conduction	regulation of membrane potential	actin filament-based process	potassium ion transmembrane transport	sodium ion transmembrane transport	calcium ion transmembrane transport	muscle contraction	heart development	response to oxygen levels	GWAS: eQTL, *in silico* QT interval loci protein network analysis (PPI-QT loci), CoIP with one of 5 LQTS Mendelian proteins (5 bait CoIP) Arking 2014	Genetic variants: LQTS Spears 2015 (Arking 2014)	Genetic variants: Atrial Fibrullation Tucker 2014	Other inherited primary arrhythmia disorders Spears 2015	Gene suggested by GO annotations	Gene suggested by eQTL or PPI analysis Arking 2014	other information
RNF207	RNF207		ring finger protein 207	Q6ZRF8	rs846111		1p36	■	■	■	■			■	■						RNF207		
RNF207		ACOT7	acyl-CoA thioesterase 7	O00154	rs846111		1p36										PPI-QT loci					ACOT7	
RNF207		KCNAB2	potassium voltage-gated channel subfamily A regulatory beta subunit 2	Q13303	rs846111		1p36				■						PPI-QT loci				KCNAB2	KCNAB2	
RNF207		RPL22	ribosomal protein L22	P35268	rs846111		1p36										PPI-QT loci					RPL22	
TCEA3	TCEA3		transcription elongation factor A3	O75764	rs2298632		1p36										eQTL					TCEA3	
NOS1AP	NOS1AP		nitric oxide synthase 1 adaptor protein	O75052	rs12143842		1q23		■	■	■		■	■							NOS1AP		
ATP1B1	ATP1B1		ATPase Na+/K+ transporting subunit beta 1	P05026	rs10919070		1q24	■	■		■	■	■	■		■	eQTL, 5 bait CoIP				ATP1B1	ATP1B1	
ATP1B1		NME7	NME/NM23 family member 7	Q9Y5B8	rs10919070		1q24										eQTL					NME7	
SLC8A1	SLC8A1		solute carrier family 8 member A1	P32418	rs12997023		2p22	■	■			■	■	■	■	■	PPI-QT loci	(LQTS)			SLC8A1		
SP3	SP3		Sp3 transcription factor	Q02447	rs938291		2q31.1																
TTN-CCDC141		CCDC141	coiled-coil domain containing 141	Q6ZP82	rs7561149		2q31.2										5 bait CoIP					CCDC141	
TTN-CCDC141		TTN	titin	Q8WZ42	rs7561149		2q31.2			■				■	■		5 bait CoIP				TTN	TTN	
SPATS2L	SPATS2L		spermatogenesis associated serine rich 2 like	Q9NUQ6	rs295140		2q33										eQTL					SPATS2L	
SPATS2L		SGO2	shugoshin 2	Q562F6	rs295140		2q33										PPI-QT loci					SGOL2	
ELP6 (C3orf75)	ELP6 (C3orf75)		elongator acetyltransferase complex subunit 6	Q0PNE2	rs17784882		3p21																
ELP6 (C3orf75)		KLHL18	kelch like family member 18	O94889	rs17784882		3p21										eQTL					KLHL18	
ELP6 (C3orf75)		MYL3	myosin light chain 3	P08590	rs17784882		3p21			■				■	■		5 bait CoIP				MYL3	MYL3	
ELP6 (C3orf75)		PTPN23	protein tyrosine phosphatase, non-receptor type 23	Q9H3S7	rs17784882		3p21										eQTL					PTPN23	positive regulation of adherens junction organization
ELP6 (C3orf75)		SCAP	SREBF chaperone	Q12770	rs17784882		3p21									■	eQTL				SCAP	SCAP	
ELP6 (C3orf75)		SETD2	SET domain containing 2	Q9BYW2	rs17784882		3p21								■		eQTL				SETD2	SETD2	
SCN5A-SCN10A		SCN5A	sodium voltage-gated channel alpha subunit 5	Q14524	rs6793245		3p22	■	■	■		■		■	■		PPI-QT loci	LQTS	AF	BrS1	SCN5A	SCN5A	
SCN5A-SCN10A		SCN10A	sodium voltage-gated channel alpha subunit 10	Q9Y5Y9	rs6793245		3p22	■	■			■		■							SCN10A		
SLC4A4	SLC4A4		solute carrier family 4 member 4	Q9Y6R1	rs2363719		4q13					■									SLC4A4		
SMARCAD1	SMARCAD1		SWI/SNF-related, matrix-associated actin-dependent regulator of chromatin,	Q9H4L7	rs3857067		4q22																
GFRA3	GFRA3		GDNF family receptor alpha 3	O60609	rs10040989		5q31																sympathetic nervous system development
GFRA3		ETF1	eukaryotic translation termination factor 1	P62495	rs10040989		5q31										PPI-QT loci					ETF1	
GFRA3		FAM13B	family with sequence similarity 13 member B	Q9NYF5	rs10040989		5q31										eQTL					FAM13B	
GMPR	GMPR		guanosine monophosphate reductase	P36959	rs7765828		6p22																
GMPR		ATXN1	ataxin 1	P54253	rs7765828		6p22		■								eQTL, PPI-QT loci				ATXN1	ATXN1	
SLC35F1-PLN		PLN	phospholamban	P26678	rs11153730		6q22	■	■	■			■	■	■		5 bait CoIP				PLN	PLN	
SLC35F1-PLN		SLC35F1	solute carrier family 35 member F1	Q5T1Q4	rs11153730		6q22																
CAV1	CAV1		caveolin 1	Q03135	rs9920	rs3807989	7q31	■	■	■	■			■		■	PPI-QT loci, 5 bait CoIP				CAV1	CAV1	caveola assembly
CAV1		CAV2	caveolin 2	P51636	rs9920		7q31										PPI-QT loci, 5 bait CoIP					CAV2	caveola assembly
CAV1		CAV3	caveolin 3	P56539	rs9920		7q31		■	■	■	■	■	■	■		not tested	LQTS			CAV3		caveola assembly
KCNH2	KCNH2		potassium voltage-gated channel subfamily H member 2	Q12809	rs2072413		7q36	■	■	■	■			■			PPI-QT loci	LQTS	AF	SQTS1	KCNH2	KCNH2	
NCOA2	NCOA2		nuclear receptor coactivator 2	Q15596	rs16936870		8q13																thyroid hormone receptor coactivator activity
LAPTM4B	LAPTM4B		lysosomal protein transmembrane 4 beta	Q86VI4	rs11779860		8q22.1																
AZIN1	AZIN1		antizyme inhibitor 1	O14977	rs1961102		8q22.3																
GBF1	GBF1		golgi brefeldin A resistant guanine nucleotide exchange factor 1	Q92538	rs2485376		10q24															ACTR1A	
GBF1		ACTR1A	ARP1 actin related protein 1 homolog A	P61163	rs2485376		10q24										5 bait CoIP						
KCNQ1	KCNQ1		potassium voltage-gated channel subfamily Q member 1	P51787	rs7122937		11p15	■	■	■	■			■			PPI-QT loci	LQTS	AF	SQTS2	KCNQ1	KCNQ1	
KCNQ1		C11orf21	chromosome 11 open reading frame 21	Q9P2W6	rs7122937		11p15										eQTL					C11orf21	
KCNQ1		TSPAN32	tetraspanin 32	Q96QS1	rs7122937		11p15										eQTL					TSPAN32	
FEN1-FADS2		FADS1	fatty acid desaturase 1	O60427	rs174583		11q12										eQTL					FADS1	
FEN1-FADS2		FADS2	fatty acid desaturase 2	O95864	rs174583		11q12										eQTL					FADS2	
FEN1-FADS2		FADS3	fatty acid desaturase 3	Q9Y5Q0	rs174583		11q12										eQTL					FADS3	
FEN1-FADS2		FEN1	flap structure-specific endonuclease 1	P39748	rs174583		11q12																
ATP2A2	ATP2A2		ATPase sarcoplasmic/endoplasmic reticulum Ca2+ transporting 2	P16615	rs3026445		12q24	■	■				■	■			PPI-QT loci, 5 bait CoIP	(LQTS)			ATP2A2	ATP2A2	
ATP2A2		ARPC3	actin related protein 2/3 complex subunit 3	O15145	rs3026445		12q24			■							eQTL				ARPC3	ARPC3	
ATP2A2		FAM216A	family with sequence similarity 216 member A	Q8WUB2	rs3026445		12q24										eQTL					FAM216A	
ATP2A2		GPN3	GPN-loop GTPase 3	Q9UHW5	rs3026445		12q24										eQTL					GPN3	
ATP2A2		VPS29	VPS29, retromer complex component	Q9UBQ0	rs3026445		12q24										eQTL					VPS29	
KLF12	KLF12		Kruppel like factor 12	Q9Y4X4	rs728926		13q22										eQTL					KLF12	
ANKRD9	ANKRD9		ankyrin repeat domain 9	Q96BM1	rs2273905		14q32										eQTL					ANKRD9	
USP50-TRPM7		TRPM7	transient receptor potential cation channel subfamily M member 7	Q96QT4	rs3105593		15q21			■			■					(LQTS)			TRPM7		
USP50-TRPM7		USP50	ubiquitin specific peptidase 50	Q70EL3	rs3105593		15q21																
LITAF	LITAF		lipopolysaccharide induced TNF factor	Q99732	rs735951		16p13										eQTL					LITAF	
MKL2	MKL2		MKL1/myocardin like 2	Q9ULH7	rs246185		16p13.12								■						MKL2		
CREBBP	CREBBP		CREB binding protein	Q92793	rs1296720		16p13.3									■	5 bait CoIP				CREBBP	CREBBP	negative regulation of cellular respiration
CREBBP		SRL	sarcalumenin	Q86TD4	rs1296720		16p13.3											(LQTS)					Sarcalumenin involved with Ca2+ handling of the sarcoplasmic reticulum
CREBBP		TRAP1	TNF receptor-associated protein 1	Q12931	rs1296720		16p13.3																
CNOT1	CNOT1		CCR4-NOT transcription complex subunit 1	A5YKK6	rs246196		16q21										eQTL				NDRG4	CNOT1	
CNOT1		GOT2	glutamic-oxaloacetic transaminase 2	P00505	rs246196		16q21										5 bait CoIP					GOT2	
CNOT1		NDRG4	NDRG family member 4	Q9ULP0	rs246196		16q21								■		eQTL					NDRG4	
LIG3	LIG3		DNA ligase 3	P49916	rs1052536		17q12										eQTL					LIG3	
LIG3		CCT6B	chaperonin containing TCP1 subunit 6B	Q92526	rs1052536		17q12										eQTL					CCT6B	
LIG3		UNC45B	unc-45 myosin chaperone B	Q8IWX7	rs1052536		17q12										5 bait CoIP					UNC45B	
KCNJ2	KCNJ2		potassium voltage-gated channel subfamily J member 2	P63252	rs1396515		17q24	■	■	■	■			■			not tested	LQTS	AF	SQTS3	KCNJ2		
PRKCA	PRKCA		protein kinase C alpha	P17252	rs9892651		17q24							■			eQTL, PPI-QT loci				PRKCA	PRKCA	
KCNE1	KCNE1		potassium voltage-gated channel subfamily E regulatory subunit 1	P15382	rs1805128		21q22	■	■	■	■			■			PPI-QT loci	LQTS	AF		KCNE1	KCNE1	
	KCNN3		potassium calcium-activated channel subfamily N member 3	Q9UGI6		rs6666258	1q21				■										KCNN3		
	PRRX1		paired related homeobox 1	P54821		rs3903239	1q24																
	CAND2		cullin associated and neddylation dissociated 2 (putative)	O75155		rs4642101	3p25																
	PITX2		paired like homeodomain 2	Q99697		rs6817105	4q25								■				AF		PITX2		
	GJA1		gap junction protein alpha 1	P17302		rs13216675	6q22	■	■	■				■	■						GJA1		
	C9orf3		chromosome 9 open reading frame 3	Q8N6M6		rs10821415	9q22																
	MYOZ1		myozenin 1	Q9NP98		rs10824026	10q22			■											MYOZ1		
	NEURL		neuralized E3 ubiquitin protein ligase 1	O76050		rs12415501	10q24																
	CUX2		cut like homeobox 2	O14529		rs6490029	12q24		■												CUX2		
	TBX5		T-box 5	Q99593		rs10507248	12q24								■						TBX5		
	SYNE2		spectrin repeat containing nuclear envelope 2	Q8WXH0		rs1152591	14q23			■											SYNE2		
	HCN4		hyperpolarization activated cyclic nucleotide gated potassium channel 4	Q9Y3Q4		rs7164883	15q24	■	■		■	■		■					AF	BrS8	HCN4		
	ZFHX3		zinc finger homeobox 3	Q15911		rs2106261	16q22																
	AKAP9		A-kinase anchoring protein 9	Q99996				■	■		■							LQTS					
	ANK2		ankyrin 2	Q01484				■	■	■	■		■	■	■			LQTS					
	CACNA1C		calcium voltage-gated channel subunit alpha1 C	Q13936				■	■				■	■				LQTS		BrS3			
	CALM1		calmodulin 1	P62158				■					■	■				LQTS		CPVT4			
	SNTA1		syntrophin alpha 1	Q13424					■	■		■		■				LQTS					
	KCNE2		potassium voltage-gated channel subfamily E regulatory subunit 2	Q9Y6J6				■	■	■	■		■	■				LQTS	AF				
	KCNJ5		potassium voltage-gated channel subfamily J member 5	P48544							■							LQTS	AF				
	SCN4B		sodium voltage-gated channel beta subunit 4	Q8IWT1				■	■	■		■		■				LQTS	AF				
	ABCC9		ATP binding cassette subfamily C member 9	O60706				■			■								AF				
	GATA4		GATA binding protein 4	P43694						■				■	■				AF				
	GATA5		GATA binding protein 5	Q9BWX5											■				AF				
	GATA6		GATA binding protein 6	Q92908											■	■			AF				
	GJA5		gap junction protein alpha 5	P36382				■	■	■				■	■				AF				
	GREM2		gremlin 2, DAN family BMP antagonist	Q9H772															AF				
	JPH2		junctophilin 2	Q9BR39									■		■				AF				
	KCNA5		potassium voltage-gated channel subfamily A member 5	P22460				■	■	■	■			■		■			AF				
	KCND3		potassium voltage-gated channel subfamily D member 3	Q9UK17				■	■		■								AF				
	KCNE3		potassium voltage-gated channel subfamily E regulatory subunit 3	Q9Y6H6					■		■		■						AF	BrS6			
	KCNE5		potassium voltage-gated channel subfamily E regulatory subunit 5	Q9UJ90				■	■	■	■			■					AF				
	KCNJ8		potassium voltage-gated channel subfamily J member 8	Q15842							■				■				AF				
	LMNA		lamin A/C	P02545											■	■			AF				
	NKX2-5		NK2 homeobox 5	P52952				■		■				■	■				AF				
	NPPA		natriuretic peptide A	P01160				■	■		■		■	■	■	■			AF				
	NUP155		nucleoporin 155	O75694				■	■	■				■					AF				
	RYR2		ryanodine receptor 2	Q92736				■	■	■			■	■	■	■			AF	CPVT1			
	SCN1B		sodium voltage-gated channel beta subunit 1	Q07699				■	■	■		■		■					AF	BrS5			
	SCN2B		sodium voltage-gated channel beta subunit 2	O60939				■	■	■		■		■					AF				
	SCN3B		sodium voltage-gated channel beta subunit 3	Q9NY72				■	■	■		■		■					AF	BrS7			
	CACNB2		calcium voltage-gated channel auxiliary subunit beta 2	Q08289				■					■							BrS4			
	CASQ2		calsequestrin 2	O14958				■	■	■	■		■	■						CPVT2			
	GPD1L		glycerol-3-phosphate dehydrogenase 1 like	Q8N335					■	■		■		■						BrS2			
	TRDN		triadin	Q13061				■	■				■	■						CPVT5			

Supplemental Table S3. Cardiac physiology relevant *Biological Process* GO terms. Nine GO terms were selected based on their potential relevance to cardiac physiology.

GO identifier	GO term name
GO:0061337	cardiac conduction
GO:0042391	regulation of membrane potential
GO:0030029	actin filament-based process
GO:0071805	potassium ion transmembrane transport
GO:0035725	sodium ion transmembrane transport
GO:0070588	calcium ion transmembrane transport
GO:0006936	muscle contraction
GO:0007507	heart development
GO:0070482	response to oxygen levels

Supplemental Table S4. List of new cardiac physiology relevant GO terms.

197 new GO terms were created to support the annotation of 88 prioritized proteins and 7 miRNAs and their role in cardiac processes. These were created by the GO editors following an ontology development meeting in November 2011 and individual requests by GO curators. Table S4 lists 200 GO terms as it includes 3 terms, 'membrane depolarization during atrial cardiac muscle cell action potential' and 'membrane repolarization during atrial cardiac muscle cell action potential', as well as 'cardiac conduction', indicated by *, that were already in the ontology). The column header 'Aspect' lists the ontology to which the GO term belongs, either Molecular Function or Biological Process. Information about the number of annotations associated with each GO term is provided (based on data available 18 October 2017) in two ways: as the number of direct annotations (number of times each GO term has been associated with a gene product from any species) and number of annotations to child term/s (number of times a child term for each GO term has been associated with a gene product from any species). Terms were considered as child terms if they had an is_a, part_of, occurs_in, regulates, positively_regulates or negatively_regulates relation to the parent term. The graph nature of the ontology means that some annotations will be represented more than once in this table.

Of the 197 new GO terms created, 136 have been directly associated with a gene product. Of the 63 terms without direct annotations, 22 provide a parent GO term that will facilitate gene grouping during functional analyses using GO datasets. For example, 'atrial cardiac muscle cell membrane repolarization' has no direct annotations, but its child terms have been used in 226 annotations. 41 terms, mostly leaf nodes, have not yet been associated with any gene product either directly or via child terms; many of these refer either to processes occurring in specific cell types, such as 'negative regulation of AV node cell action potential', or describe very specific functions, e.g. 'stretch-activated, cation-selective, calcium channel activity involved in regulation of action potential'. Some of these areas of study did not fall under the focus of this project, and relevant literature was not examined in detail. Future annotation projects, for example looking at signaling pathways that regulate both the rate and strength of heart contractions, may lead to the association of proteins or RNAs with these terms.

Aspect	GO ID	GO term name	Number annotations using term listed	Number annotations using child term
**Child terms of GO term cardiac conduction*, GO:0061337**				
Biological Process	GO:0086014	atrial cardiac muscle cell action potential	215	
Biological Process	GO:0099624	atrial cardiac muscle cell membrane repolarization	0	226
Biological Process	GO:0086066	atrial cardiac muscle cell to AV node cell communication	13	
Biological Process	GO:0086044	atrial cardiac muscle cell to AV node cell communication by electrical coupling	76	
Biological Process	GO:0086026	atrial cardiac muscle cell to AV node cell signaling	0	604
Biological Process	GO:0086071	atrial cardiac muscle cell-AV node cell adhesion involved in cell communication	0	0
Biological Process	GO:0086016	AV node cell action potential	112	
Biological Process	GO:0086067	AV node cell to bundle of His cell communication	52	
Biological Process	GO:0086053	AV node cell to bundle of His cell communication by electrical coupling	121	
Biological Process	GO:0086027	AV node cell to bundle of His cell signaling	44	
Biological Process	GO:0086072	AV node cell-bundle of His cell adhesion involved in cell communication	4	
Biological Process	GO:0086043	bundle of His cell action potential	32	
Biological Process	GO:0086069	bundle of His cell to Purkinje myocyte communication	4	
Biological Process	GO:0086054	bundle of His cell to Purkinje myocyte communication by electrical coupling	35	
Biological Process	GO:0086028	bundle of His cell to Purkinje myocyte signaling	0	285
Biological Process	GO:0086073	bundle of His cell-Purkinje myocyte adhesion involved in cell communication	181	
Biological Process	GO:0086064	cell communication by electrical coupling involved in cardiac conduction	91	
Biological Process	GO:0086065	cell communication involved in cardiac conduction	39	
Biological Process	GO:0086019	cell-cell signaling involved in cardiac conduction	40	
Biological Process	GO:0098912	membrane depolarization during atrial cardiac muscle cell action potential*	71	
Biological Process	GO:0086045	membrane depolarization during AV node cell action potential	140	
Biological Process	GO:0086048	membrane depolarization during bundle of His cell action potential	62	
Biological Process	GO:0086047	membrane depolarization during Purkinje myocyte cell action potential	60	
Biological Process	GO:0086046	membrane depolarization during SA node cell action potential	138	
Biological Process	GO:0098914	membrane repolarization during atrial cardiac muscle cell action potential*	93	
Biological Process	GO:0086049	membrane repolarization during AV node cell action potential	0	0
Biological Process	GO:0086050	membrane repolarization during bundle of His cell action potential	27	
Biological Process	GO:0086051	membrane repolarization during Purkinje myocyte action potential	0	0
Biological Process	GO:0086052	membrane repolarization during SA node cell action potential	29	
Biological Process	GO:1903948	negative regulation of atrial cardiac muscle cell action potential	0	0
Biological Process	GO:1903950	negative regulation of AV node cell action potential	0	0
Biological Process	GO:1903780	negative regulation of cardiac conduction	0	0
Biological Process	GO:1901845	negative regulation of cell communication by electrical coupling involved in cardiac conduction	0	0
Biological Process	GO:1905028	negative regulation of membrane depolarization during AV node cell action potential	0	0
Biological Process	GO:1905001	negative regulation of membrane repolarization during atrial cardiac muscle cell action potential	0	0
Biological Process	GO:1903953	negative regulation of voltage-gated potassium channel activity involved in atrial cardiac muscle cell action potential repolarization	0	0
Biological Process	GO:1903949	positive regulation of atrial cardiac muscle cell action potential	3	
Biological Process	GO:1903951	positive regulation of AV node cell action potential	0	0
Biological Process	GO:1903781	positive regulation of cardiac conduction	0	8
Biological Process	GO:1901846	positive regulation of cell communication by electrical coupling involved in cardiac conduction	2	
Biological Process	GO:1905029	positive regulation of membrane depolarization during AV node cell action potential	0	0
Biological Process	GO:1905002	positive regulation of membrane repolarization during atrial cardiac muscle cell action potential	0	0
Biological Process	GO:1903954	positive regulation of voltage-gated potassium channel activity involved in atrial cardiac muscle cell action potential repolarization	3	
Biological Process	GO:0086017	Purkinje myocyte action potential	0	131
Biological Process	GO:0086068	Purkinje myocyte to ventricular cardiac muscle cell communication	0	160
Biological Process	GO:0086055	Purkinje myocyte to ventricular cardiac muscle cell communication by electrical coupling	1	
Biological Process	GO:0086029	Purkinje myocyte to ventricular cardiac muscle cell signaling	27	
Biological Process	GO:0086074	Purkinje myocyte-ventricular cardiac muscle cell adhesion involved in cell communication	0	0

Biological Process	GO:0098910	regulation of atrial cardiac muscle cell action potential	127	
Biological Process	GO:0098904	regulation of AV node cell action potential	113	
Biological Process	GO:0098905	regulation of bundle of His cell action potential	99	
Biological Process	GO:1903779	regulation of cardiac conduction	447	
Biological Process	GO:1901844	regulation of cell communication by electrical coupling involved in cardiac conduction	106	
Biological Process	GO:0086091	regulation of heart rate by cardiac conduction	1171	
Biological Process	GO:1905027	regulation of membrane depolarization during AV node cell action potential	0	0
Biological Process	GO:1905000	regulation of membrane repolarization during atrial cardiac muscle cell action potential	2	
Biological Process	GO:0098906	regulation of Purkinje myocyte action potential	35	
Biological Process	GO:0098907	regulation of SA node cell action potential	84	
Biological Process	GO:0086092	regulation of the force of heart contraction by cardiac conduction	68	
Biological Process	GO:1903952	regulation of voltage-gated potassium channel activity involved in atrial cardiac muscle cell action potential repolarization	0	3
Biological Process	GO:0086015	SA node cell action potential	165	
Biological Process	GO:0086070	SA node cell to atrial cardiac muscle cell communication	70	
Biological Process	GO:0086021	SA node cell to atrial cardiac muscle cell communication by electrical coupling	3	
Biological Process	GO:0086018	SA node cell to atrial cardiac muscle cell signalling	44	
Biological Process	GO:0086022	SA node cell-atrial cardiac muscle cell adhesion involved in cell communication	0	0
Molecular Function	GO:0086082	cell adhesive protein binding involved in AV node cell-bundle of His cell communication	2	
Molecular Function	GO:0086083	cell adhesive protein binding involved in bundle of His cell-Purkinje myocyte communication	17	
Molecular Function	GO:0086076	gap junction channel activity involved in atrial cardiac muscle cell-AV node cell electrical coupling	40	
Molecular Function	GO:0086077	gap junction channel activity involved in AV node cell-bundle of His cell electrical coupling	85	
Molecular Function	GO:0086078	gap junction channel activity involved in bundle of His cell-Purkinje myocyte electrical coupling	35	
Molecular Function	GO:0086075	gap junction channel activity involved in cardiac conduction electrical coupling	40	
Molecular Function	GO:0086079	gap junction channel activity involved in Purkinje myocyte-ventricular cardiac muscle cell electrical coupling	1	
Molecular Function	GO:0086020	gap junction channel activity involved in SA node cell-atrial cardiac muscle cell electrical coupling	3	
Molecular Function	GO:0086056	voltage-gated calcium channel activity involved in AV node cell action potential	112	
Molecular Function	GO:0086057	voltage-gated calcium channel activity involved in bundle of His cell action potential	34	
Molecular Function	GO:0086058	voltage-gated calcium channel activity involved in Purkinje myocyte cell action potential	0	0
Molecular Function	GO:0086059	voltage-gated calcium channel activity involved SA node cell action potential	104	
Molecular Function	GO:0086089	voltage-gated potassium channel activity involved in atrial cardiac muscle cell action potential repolarization	90	
Molecular Function	GO:0086086	voltage-gated potassium channel activity involved in AV node cell action potential repolarization	0	0
Molecular Function	GO:0086087	voltage-gated potassium channel activity involved in bundle of His cell action potential repolarization	27	
Molecular Function	GO:0086088	voltage-gated potassium channel activity involved in Purkinje myocyte action potential repolarization	0	0
Molecular Function	GO:0086041	voltage-gated potassium channel activity involved in SA node cell action potential depolarization	29	
Molecular Function	GO:0086090	voltage-gated potassium channel activity involved in SA node cell action potential repolarization	29	
Molecular Function	GO:0086060	voltage-gated sodium channel activity involved in AV node cell action potential	4	
Molecular Function	GO:0086061	voltage-gated sodium channel activity involved in bundle of His cell action potential	4	
Molecular Function	GO:0086062	voltage-gated sodium channel activity involved in Purkinje myocyte action potential	36	
Molecular Function	GO:0086063	voltage-gated sodium channel activity involved in SA node cell action potential	6	

Other cardiac-relevant GO terms

Biological Process	GO:0086096	adenylate cyclase-inhibiting adrenergic receptor signaling pathway involved in heart process	0	0
Biological Process	GO:0086102	adenylate cyclase-inhibiting G-protein coupled acetylcholine receptor signaling pathway involved in negative regulation of heart rate	0	0
Biological Process	GO:0086030	adrenergic receptor signaling pathway involved in cardiac muscle relaxation	3	
Biological Process	GO:0086023	adrenergic receptor signaling pathway involved in heart process	62	
Biological Process	GO:0086024	adrenergic receptor signaling pathway involved in positive regulation of heart rate	2	
Biological Process	GO:0038166	angiotensin-activated signaling pathway	784	
Biological Process	GO:0086098	angiotensin-activated signaling pathway involved in heart process	73	
Biological Process	GO:0090662	ATP hydrolysis coupled transmembrane transport	2577	
Biological Process	GO:1903515	calcium ion transport from cytosol to endoplasmic reticulum	56	
Biological Process	GO:1903514	calcium ion transport from endoplasmic reticulum to cytosol	36	
Biological Process	GO:0086001	cardiac muscle cell action potential	65	
Biological Process	GO:0086002	cardiac muscle cell action potential involved in contraction	436	
Biological Process	GO:0086003	cardiac muscle cell contraction	12	
Biological Process	GO:0086042	cardiac muscle cell-cardiac muscle cell adhesion	63	
Biological Process	GO:0014898	cardiac muscle hypertrophy in response to stress	440	
Biological Process	GO:0060926	cardiac pacemaker cell development	0	100
Biological Process	GO:0060920	cardiac pacemaker cell differentiation	0	150
Biological Process	GO:0060927	cardiac pacemaker cell fate commitment	0	33
Biological Process	GO:1903513	endoplasmic reticulum to cytosol transport	0	1787
Biological Process	GO:0086100	endothelin receptor signaling pathway	561	
Biological Process	GO:0086101	endothelin receptor signaling pathway involved in heart process	0	0
Biological Process	GO:0086093	G-protein coupled acetylcholine receptor signaling pathway involved in involved in heart process	0	0
Biological Process	GO:0086033	G-protein coupled acetylcholine receptor signaling pathway involved in negative regulation of heart rate	0	0
Biological Process	GO:0086103	G-protein coupled receptor signaling pathway involved in heart process	35	
Biological Process	GO:0086010	membrane depolarization during action potential	757	
Biological Process	GO:0086012	membrane depolarization during cardiac muscle cell action potential	183	
Biological Process	GO:0098913	membrane depolarization during ventricular cardiac muscle cell action potential	0	0

Biological Process	GO:0086009	membrane repolarization	310	
Biological Process	GO:0086011	membrane repolarization during action potential	142	
Biological Process	GO:0086013	membrane repolarization during cardiac muscle cell action potential	143	
Biological Process	GO:0098915	membrane repolarization during ventricular cardiac muscle cell action potential	156	
Biological Process	GO:1901205	negative regulation of adrenergic receptor signaling pathway involved in heart process	32	
Biological Process	GO:1903246	negative regulation of adrenergic receptor signaling pathway involved in positive regulation of heart rate	0	0
Biological Process	GO:1903280	negative regulation of calcium:sodium antiporter activity	6	
Biological Process	GO:1902260	negative regulation of delayed rectifier potassium channel activity	182	
Biological Process	GO:1903597	negative regulation of gap junction assembly	36	
Biological Process	GO:1903609	negative regulation of inward rectifier potassium channel activity	36	
Biological Process	GO:1900826	negative regulation of membrane depolarization during cardiac muscle cell action potential	34	
Biological Process	GO:1905032	negative regulation of membrane repolarization during cardiac muscle cell action potential	0	36
Biological Process	GO:1905025	negative regulation of membrane repolarization during ventricular cardiac muscle cell action potential	36	
Biological Process	GO:1902309	negative regulation of peptidyl-serine dephosphorylation	102	
Biological Process	GO:1902303	negative regulation of potassium ion export	28	
Biological Process	GO:1903765	negative regulation of potassium ion export across plasma membrane	36	
Biological Process	GO:1903287	negative regulation of potassium ion import	0	0
Biological Process	GO:1903783	negative regulation of sodium ion import across plasma membrane	0	0
Biological Process	GO:1902306	negative regulation of sodium ion transmembrane transport	95	
Biological Process	GO:0098736	negative regulation of the force of heart contraction	31	
Biological Process	GO:1903946	negative regulation of ventricular cardiac muscle cell action potential	0	36
Biological Process	GO:1903761	negative regulation of voltage-gated potassium channel activity involved in ventricular cardiac muscle cell action potential repolarization	0	0
Biological Process	GO:0086097	phospholipase C-activating angiotensin-activated signaling pathway	44	
Biological Process	GO:0086099	phospholipase C-activating angiotensin-activated signaling pathway involved in heart process	0	0
Biological Process	GO:0003301	physiological cardiac muscle hypertrophy	13	
Biological Process	GO:1901206	positive regulation of adrenergic receptor signaling pathway involved in heart process	29	
Biological Process	GO:1903247	positive regulation of adrenergic receptor signaling pathway involved in positive regulation of heart rate	0	0
Biological Process	GO:1903281	positive regulation of calcium:sodium antiporter activity	75	
Biological Process	GO:1903598	positive regulation of gap junction assembly	114	
Biological Process	GO:0086095	positive regulation of IKACh channel activity by G-protein coupled acetylcholine receptor signaling pathway involved in negative regulation of heart rate	0	0
Biological Process	GO:1900827	positive regulation of membrane depolarization during cardiac muscle cell action potential	3	
Biological Process	GO:1905033	positive regulation of membrane repolarization during cardiac muscle cell action potential	0	40
Biological Process	GO:1905026	positive regulation of membrane repolarization during ventricular cardiac muscle cell action potential	0	37
Biological Process	GO:1902310	positive regulation of peptidyl-serine dephosphorylation	85	
Biological Process	GO:1902304	positive regulation of potassium ion export	6	
Biological Process	GO:1903766	positive regulation of potassium ion export across plasma membrane	34	
Biological Process	GO:1903288	positive regulation of potassium ion import	138	
Biological Process	GO:0086094	positive regulation of ryanodine-sensitive calcium-release channel activity by adrenergic receptor signaling pathway involved in positive regulation of cardiac muscle contraction	0	0
Biological Process	GO:1903784	positive regulation of sodium ion import across plasma membrane	0	0
Biological Process	GO:1902307	positive regulation of sodium ion transmembrane transport	6	
Biological Process	GO:0098735	positive regulation of the force of heart contraction	161	
Biological Process	GO:1903947	positive regulation of ventricular cardiac muscle cell action potential	0	37
Biological Process	GO:1903762	positive regulation of voltage-gated potassium channel activity involved in ventricular cardiac muscle cell action potential repolarization	37	
Biological Process	GO:0003165	Purkinje myocyte development	34	
Biological Process	GO:0003168	Purkinje myocyte differentiation	33	
Biological Process	GO:1901204	regulation of adrenergic receptor signaling pathway involved in heart process	0	61
Biological Process	GO:1903245	regulation of adrenergic receptor signaling pathway involved in positive regulation of heart rate	0	0
Biological Process	GO:1903279	regulation of calcium:sodium antiporter activity	0	81
Biological Process	GO:0098901	regulation of cardiac muscle cell action potential	20	
Biological Process	GO:0098909	regulation of cardiac muscle cell action potential involved in regulation of contraction	163	
Biological Process	GO:0086004	regulation of cardiac muscle cell contraction	262	
Biological Process	GO:0086036	regulation of cardiac muscle cell membrane potential	200	
Biological Process	GO:1903596	regulation of gap junction assembly	0	150
Biological Process	GO:1900825	regulation of membrane depolarization during cardiac muscle cell action potential	133	
Biological Process	GO:0060306	regulation of membrane repolarization	341	
Biological Process	GO:1905031	regulation of membrane repolarization during cardiac muscle cell action potential	3	
Biological Process	GO:1905024	regulation of membrane repolarization during ventricular cardiac muscle cell action potential	0	104
Biological Process	GO:1902308	regulation of peptidyl-serine dephosphorylation	0	187
Biological Process	GO:1902302	regulation of potassium ion export	0	135
Biological Process	GO:1903764	regulation of potassium ion export across plasma membrane	31	
Biological Process	GO:1903286	regulation of potassium ion import	31	
Biological Process	GO:0014861	regulation of skeletal muscle contraction via regulation of action potential	43	
Biological Process	GO:1903782	regulation of sodium ion import across plasma membrane	0	0
Biological Process	GO:1902305	regulation of sodium ion transmembrane transport	167	
Biological Process	GO:0098911	regulation of ventricular cardiac muscle cell action potential	337	
Biological Process	GO:0060307	regulation of ventricular cardiac muscle cell membrane repolarization	461	
Biological Process	GO:1903760	regulation of voltage-gated potassium channel activity involved in ventricular cardiac muscle cell action potential repolarization	31	

Biological Process	GO:1903416	response to glycoside	78	
Biological Process	GO:0086005	ventricular cardiac muscle cell action potential	392	
Biological Process	GO:0099625	ventricular cardiac muscle cell membrane repolarization	34	
Molecular Function	GO:0086039	calcium-transporting ATPase activity involved in regulation of cardiac muscle cell membrane potential	10	
Molecular Function	GO:0086038	calcium:sodium antiporter activity involved in regulation of cardiac muscle cell membrane potential	42	
Molecular Function	GO:0086081	cell adhesive protein binding involved in atrial cardiac muscle cell-AV node cell communication	0	0
Molecular Function	GO:0086084	cell adhesive protein binding involved in Purkinje myocyte-ventricular cardiac muscle cell communication	0	0
Molecular Function	GO:0086085	cell adhesive protein binding involved in SA cardiac muscle cell-atrial cardiac muscle cell communication	0	0
Molecular Function	GO:1903763	gap junction channel activity involved in cell communication by electrical coupling	150	
Molecular Function	GO:0086080	protein binding involved in heterotypic cell-cell adhesion	123	
Molecular Function	GO:0086037	sodium:potassium-exchanging ATPase activity involved in regulation of cardiac muscle cell membrane potential	26	
Molecular Function	GO:0086040	sodium:proton antiporter activity involved in regulation of cardiac muscle cell membrane potential	1	
Molecular Function	GO:0097364	stretch-activated, cation-selective, calcium channel activity involved in regulation of action potential	0	0
Molecular Function	GO:0097365	stretch-activated, cation-selective, calcium channel activity involved in regulation of cardiac muscle cell action potential	0	0
Molecular Function	GO:0086007	voltage-gated calcium channel activity involved in cardiac muscle cell action potential	76	
Molecular Function	GO:0086008	voltage-gated potassium channel activity involved in cardiac muscle cell action potential repolarization	89	
Molecular Function	GO:1902282	voltage-gated potassium channel activity involved in ventricular cardiac muscle cell action potential repolarization	157	
Molecular Function	GO:0086006	voltage-gated sodium channel activity involved in cardiac muscle cell action potential	225	
		**Total direct annotations**	16,935	

Supplemental Table S5. Functional analysis of adult ventricular cardiomyocyte data using Gene Ontology.

Supplemental Table S5A. hA-VCMs protein list. The 200 most abundant transcripts in hA-VCMs, as compared to hESCs transcripts, as listed by Poon et al.[19]

HGNC Symbol	EntrezGene ID	UniProtKB ID	comments
MYL2	4633	P10916	
MYH7	4625	P12883	
MYL3	4634	P08590	
ACTC1	70	P68032	
HS.508682			Not available
CRYAB	1410	P02511	
ACTA1	58	P68133	
CKM	1158	P06732	
ATP5B	506	P06576	
COX4I1	1327	P13073	
FARSB	10056	Q9NSD9	
TNNC1	7134	P63316	
RPS16	6217	P62249	
HSPB7	27129	Q9UBY9	
RN7SL1	6029		RNA, 7SL, cytoplasmic 1
MYOM1	8736	P52179	
HSPB1	3315	P04792	
MYBPC3	4607	Q14896	
TMSB4XP8	7117		thymosin beta 4, X-linked pseudogene 8
MYL12A	10627	P19105	
COX7C	1350	P15954	
SLC25A4	291	P12235	
RPLP2	6181	P05387	
DES	1674	P17661	
NDUFA1	4694	O15239	
EEF1A2	1917	Q05639	
ATP5A1	498	P25705	
OAZ1	4946	P54368	
COX7A1	1346	P24310	
MDH1	4190	P40925	
TPT1	7178	P13693	
NDUFA4	4697	O00483	
ATP5E	514	P56381	
RPS25	6230	P62851	
CD81	975	P60033	
MYH6	4624	P13533	
COX5B	1329	P10606	
TCAP	8557	O15273	
MYOM2	9172	P54296	
RPS27	6232	P42677	
UQCRQ	27089	O14949	
TNNI3	7137	P19429	
MDH2	4191	P40926	
CHCHD10	400916	Q8WYQ3	
COX5A	9377	P20674	
CASQ2	845	O14958	
UBC	7316	P0CG48	
LAIR1	3903	Q6GTX8	
CKMT2	1160	P17540	
MB	4151	P02144	
TPM1	7168	P09493	
RPL38	6169	P63173	
SLC7A5P2	387254		solute carrier family 7 member 5 pseudogene 2
TOMM7	54543	Q9P0U1	
FLNC	2318	Q14315	
PSAP	5660	P07602	
TNNT2	7139	P45379	
RPS10	6204	P46783	
CDH2	1000	P19022	
F2R	2149	P25116	
C19ORF31	404664		HGNC listed as a cloning artifact
RACK1	10399	P63244	
PTGDS	5730	P41222	
HRC	3270	P23327	
EEF2	1938	P13639	
RPS19	6223	P39019	
YBX3	8531	P16989	
RPL11	6135	P62913	

COX6C	1345	P09669	
RPL18A	6142	Q02543	
NDUFB8	4714	O95169	
GPX3	2878	P22352	
PAM	5066	P19021	
MFGE8	4240	Q08431	
ATP5H	10476	O75947	
EEF1A1	1915	P68104	
IL18	3606	Q14116	
NDUFAB1	4706	O14561	
ECH1	1891	Q13011	
CMYA5	202333	Q8N3K9	
GAPDH	2597	P04406	
RPS29	6235	P62273	
FTL	2512	P02792	
SRL	6345	Q86TD4	
SYNPO2L	79933	Q9H987	
APOD	347	P05090	
MYL7	58498	Q01449	
COX7A2	1347	P14406	
RPS27A	6233	P62979	
ORC6	23594	Q9Y5N6	
FHL2	2274	Q14192	
RPS20	6224	P60866	
RPLP1	6176	P05386	
UBB	7314	P0CG47	
COX8A	1351	P10176	
TUBA1B	10376	P68363	
CST3	1471	P01034	
RPL41	6171	P62945	
NAG18	57051		withdrawn by NCBI
EIF4A2	1974	Q14240	
HBB	3043	P68871	
HSPA1A	3303	P0DMV8	
ROCK2	9475	O75116	
RPL19	6143	P84098	
MSH3	4437	P20585	
NPPB	4879	P16860	
ZNF486	90649	Q96H40	
HBA1	3039	P69905	
UQCRFS1	7386	P47985	
CCNI	10983	Q14094	
RPL24	6152	P83731	
TUBA3FP	113691		tubulin alpha 3f pseudogene
RPL27	6155	P61353	
ATP2A2	488	P16615	
HSPB3	8988	Q12988	
LILRB3	11025	Q6PI73	
RPS11	6205	P62280	
NDUFS5	4725	O43920	
SOD1	6647	P00441	
CHCHD2	51142	Q9Y6H1	
JUND	3727	P17535	
ENO3	2027	P13929	
CYC1	1537	P08574	
H3F3A	3020	P84243	
RPS2	6187	P15880	
NDUFS8	4728	O00217	
PDK4	5166	Q16654	
ATP5O	539	P48047	
RPL35A	6165	P18077	
LDHB	3945	P07195	
RPS3A	6189	P61247	
NUCB1	4924	Q02818	
IDH2	3418	P48735	
EIF3E	3646	P60228	
RPS6	6194	P62753	
GOT2	2806	P00505	
NDUFA3	4696	O95167	

RHOQ	23433	P17081
VDAC3	7419	Q9Y277
VWF	7450	P04275
COX6B1	1340	P14854
MGST3	4259	O14880
RPL18	6141	Q07020
RBPMS2	348093	Q6ZRY4
HSPA1B	3304	P0DMV9
GOT1	2805	P17174
RPS12	6206	P25398
SRP14	6727	P37108
RPL6	6128	Q02878
RPL39	6170	P62891
IFITM3	10410	Q01628
NDUFB10	4716	O96000
RPS17	6218	P08708
NDUFA8	4702	P51970
TMSB10	9168	P63313
ATP5J	522	P18859
AURKAIP1	54998	Q9NWT8
ATP5C1	509	P36542
GHITM	27069	Q9H3K2
UBA52	7311	P62987
SPARCL1	8404	Q14515
RPL35	11224	P42766
RPS14	6208	P62263
DUSP3	1845	P51452
LGALS1	3956	P09382
NACA	4666	Q13765
RPL27A	6157	P46776
NMRK2	27231	Q9NPI5
FAU	2197	P35544
NPPA	4878	P01160
NCOA4	8031	Q13772
RPL3	6122	P39023
PCLAF	9768	Q15004
GABPB2	126626	Q8TAK5
MGP	4256	P08493
NDUFB5	4711	O43674
PDE4C	5143	Q08493
MT1X	4501	P80297
ALKBH5	54890	Q6P6C2
RPL10A	4736	P62906
AKR1D1	6718	P51857
RPS28	6234	P62857
HSPB6	126393	O14558
GPD1L	23171	Q8N335
PDCD7	10081	Q8N8D1
WBP2	23558	Q969T9
RPL5	6125	P46777
ACAT1	38	P24752
NDUFB2	4708	O95178
COX7B	1349	P24311
CCR6	1235	P51684
MYL6	4637	P60660
BSG	682	P35613
DECR1	1666	Q16698
TIMP1	7076	P01033
MSRB2	22921	Q9Y3D2
CALM2	805	P62158
TSPAN9	10867	O75954
RPS18	6222	P62269
SLC25A3	5250	Q00325

Supplemental Table S5B. hA-VCMs BinGO 2016. BinGO analysis of the adult ventricular cardiomyocyte (hA-VCMs) data[19] using the 2016 GO annotation dataset. **x** is the number of IDs in both the submitted list and associated with the GO term, **n** indicates the number of protein IDs associated with the GO term, **X** is the number of protein IDs submitted for analysis, **N** is the number of protein IDs used as the background set.

GO-ID	GO term	p-value	corr p-value	x	n	X	N	Proteins submitted in analysis that are associated with the GO term
GO:0000027	ribosomal large subunit assembly	2.49E-09	3.37E-08	6	24	190	35980	Q02878|P63173|P39023|P83731|P62913|P46777
GO:0000028	ribosomal small subunit assembly	5.13E-10	7.26E-09	6	19	190	35980	P42677|P63173|P08708|P62263|P46783|P39019
GO:0000075	cell cycle checkpoint	1.57E-02	4.33E-02	5	275	190	35980	P0CG47|P62987|P83731|P62979|P0CG48
GO:0000077	DNA damage checkpoint	1.28E-02	3.77E-02	4	170	190	35980	P0CG47|P62987|P62979|P0CG48
GO:0000082	G1/S transition of mitotic cell cycle	2.50E-03	1.02E-02	5	176	190	35980	P0CG47|P62987|Q9Y5N6|P62979|P0CG48
GO:0000086	G2/M transition of mitotic cell cycle	6.95E-03	2.33E-02	4	142	190	35980	P0CG47|P62987|P62979|P0CG48
GO:0000165	MAPK cascade	1.39E-03	6.35E-03	8	401	190	35980	P0CG47|P51452|Q14116|P62987|P62979|P02511|P62158|P0CG48
GO:0000184	nuclear-transcribed mRNA catabolic process, nonsense-mediated decay	6.70E-55	3.32E-52	37	125	190	35980	P25398|P62280|P39019|P62891|P42766|Q02878|P39023|P62273|P46783|P61247|P83731|P62857|P62913|P62979|P62851|P62753|Q07020|P08708|P62906|P18077|P42677|P84098|P15880|P63173|Q02543|P61353|P62263|P62987|P62945|P46777|P05386|P60866|P46776|P62269|P05387|P60228|P62249
GO:0000186	activation of MAPKK activity	1.96E-03	8.37E-03	6	244	190	35980	P0CG47|P62987|P62979|P62158|P25116|P0CG48
GO:0000187	activation of MAPK activity	1.32E-04	8.21E-04	6	146	190	35980	P0CG47|P60033|P62987|P62979|P00441|P0CG48
GO:0000271	polysaccharide biosynthetic process	1.13E-04	7.13E-04	4	47	190	35980	P0CG47|P62987|P62979|P0CG48
GO:0000302	response to reactive oxygen species	1.97E-07	2.07E-06	10	210	190	35980	P02144|P05090|P13639|P68871|P09493|P22352|P02511|P69905|P00441|P01034
GO:0000422	mitophagy	6.34E-06	5.43E-05	8	183	190	35980	P0CG47|P52179|Q9P0U1|P40925|P62987|P62979|P10176|P0CG48
GO:0000423	macromitophagy	1.68E-06	1.57E-05	8	153	190	35980	P0CG47|P52179|Q9P0U1|P40925|P62987|P62979|P10176|P0CG48
GO:0000462	maturation of SSU-rRNA from tricistronic rRNA transcript (SSU-rRNA, 5.8S rRNA, LSU-rRNA)	3.45E-04	1.91E-03	3	26	190	35980	P62263|P62249|P39019
GO:0000715	nucleotide-excision repair, DNA damage recognition	5.12E-06	4.49E-05	4	22	190	35980	P0CG47|P62987|P62979|P0CG48
GO:0000724	double-strand break repair via homologous recombination	8.20E-03	2.68E-02	4	149	190	35980	P0CG47|P62987|P62979|P0CG48
GO:0000725	recombinational repair	8.39E-03	2.72E-02	4	150	190	35980	P0CG47|P62987|P62979|P0CG48
GO:0000956	nuclear-transcribed mRNA catabolic process	3.50E-48	6.12E-46	38	200	190	35980	P25398|P62280|P39019|P62891|P42766|Q02878|P39023|P62273|P46783|P61247|P83731|P62857|P62913|P62979|P62851|P62753|Q07020|P08708|P62906|P18077|P42677|P84098|P15880|P63173|Q02543|P61353|P62263|P62987|Q14240|P62945|P46777|P05386|P60866|P46776|P62269|P05387|P60228|P62249
GO:0001503	ossification	5.25E-04	2.73E-03	7	263	190	35980	P63244|P63173|P08493|Q14192|P62280|P17535|P06576
GO:0001649	osteoblast differentiation	6.10E-04	3.10E-03	5	128	190	35980	P63244|Q14192|P62280|P17535|P06576
GO:0001666	response to hypoxia	5.41E-06	4.72E-05	10	303	190	35980	P0CG47|P02144|P01160|P19021|P62987|P62979|P02511|Q6P6C2|P0CG48|P01034
GO:0001775	cell activation	5.90E-04	3.01E-03	12	737	190	35980	P07602|P01033|Q14116|P09382|P68871|P04275|P62158|P04792|P19105|P25116|P00441|P01034
GO:0001817	regulation of cytokine production	4.56E-04	2.43E-03	11	614	190	35980	P0CG47|Q14116|P05090|P0DMV8|P0DMV9|P62987|P62979|P04792|P25116|P00441|P0CG48
GO:0001818	negative regulation of cytokine production	7.43E-03	2.45E-02	5	228	190	35980	P0CG47|P05090|P62987|P62979|P0CG48
GO:0001819	positive regulation of cytokine production	5.02E-05	3.54E-04	10	393	190	35980	P0CG47|Q14116|P0DMV8|P0DMV9|P62987|P62979|P04792|P25116|P00441|P0CG48
GO:0001887	selenium compound metabolic process	2.79E-54	8.29E-52	36	116	190	35980	P25398|P62280|P39019|P62891|P42766|Q02878|P39023|P62273|P46783|P61247|P83731|P62857|P62913|P62979|P62851|P62753|Q07020|P08708|P62906|P18077|P42677|P84098|P15880|P63173|Q02543|P61353|P62263|P62987|P62945|P46777|P05386|P60866|P46776|P62269|P05387|P62249
GO:0001932	regulation of protein phosphorylation	2.73E-03	1.09E-02	17	1505	190	35980	P07602|P0CG47|P51452|Q14116|O75116|P19022|P0CG48|P63244|P60033|P62987|P23327|P62979|P62158|P04792|P25116|Q8N335|P00441
GO:0001934	positive regulation of protein phosphorylation	4.24E-03	1.61E-02	13	1057	190	35980	P07602|P0CG47|Q14116|O75116|P19022|P0CG48|P63244|P60033|P62987|P62979|P62158|P25116|P00441
GO:0001959	regulation of cytokine-mediated signaling pathway	1.12E-05	9.10E-05	7	142	190	35980	P0CG47|P63244|P0DMV8|P0DMV9|P62987|P62979|P0CG48
GO:0002026	regulation of the force of heart contraction	5.92E-07	5.93E-06	5	31	190	35980	P10916|P08590|P16615|P13533|P12883
GO:0002027	regulation of heart rate	5.27E-11	7.84E-10	10	90	190	35980	Q14896|O14958|P01160|P09493|P16615|P23327|P62158|P13533|Q8N335|P12883
GO:0002218	activation of innate immune response	2.58E-03	1.05E-02	6	258	190	35980	P0CG47|P51452|P62987|P62979|P62158|P0CG48
GO:0002220	innate immune response activating cell surface receptor	5.09E-04	2.65E-03	5	123	190	35980	P0CG47|P62987|P62979|P62158|P0CG48
GO:0002221	pattern recognition receptor signaling pathway	1.48E-03	6.64E-03	5	156	190	35980	P0CG47|P51452|P62987|P62979|P0CG48
GO:0002223	stimulatory C-type lectin receptor signaling pathway	4.54E-04	2.43E-03	5	120	190	35980	P0CG47|P62987|P62979|P62158|P0CG48
GO:0002224	toll-like receptor signaling pathway	8.29E-04	4.08E-03	5	137	190	35980	P0CG47|P51452|P62987|P62979|P0CG48
GO:0002230	positive regulation of defense response to virus by host	3.67E-05	2.69E-04	6	116	190	35980	P52179|P40925|P62987|Q16654|Q6P6C2|P10176
GO:0002262	myeloid cell homeostasis	2.14E-04	1.26E-03	5	102	190	35980	P02144|P08708|P62263|P00441|P39019
GO:0002479	antigen processing and presentation of exogenous peptide antigen via MHC class I, TAP-dependent	6.95E-03	2.33E-02	4	142	190	35980	P0CG47|P62987|P62979|P0CG48
GO:0002576	platelet degranulation	9.07E-05	5.88E-04	5	85	190	35980	P07602|P01033|P04275|P62158|P00441
GO:0002682	regulation of immune system process	7.80E-04	3.88E-03	21	1842	190	35980	P0CG47|P51452|P52179|Q14116|Q6GTX8|P51684|P0DMV8|P0DMV9|P40925|Q6P6C2|P10176|P0CG48|P39019|P05090|P60033|P09382|P62987|Q16654|P62979|P62158|P00441
GO:0002684	positive regulation of immune system process	5.84E-03	2.01E-02	13	1099	190	35980	P0CG47|P51452|Q14116|P51684|P0DMV8|P0DMV9|P0CG48|P39019|P60033|P09382|P62987|P62979|P62158
GO:0002697	regulation of immune effector process	8.55E-03	2.76E-02	7	434	190	35980	P52179|P40925|P62987|Q16654|Q6P6C2|P10176|P39019
GO:0002753	cytoplasmic pattern recognition receptor signaling	3.09E-05	2.32E-04	4	34	190	35980	P0CG47|P62987|P62979|P0CG48
GO:0002755	MyD88-dependent toll-like receptor signaling pathway	1.46E-04	9.01E-04	5	94	190	35980	P0CG47|P51452|P62987|P62979|P0CG48
GO:0002756	MyD88-independent toll-like receptor signaling pathway	9.07E-05	5.88E-04	5	85	190	35980	P0CG47|P51452|P62987|P62979|P0CG48
GO:0002758	innate immune response-activating signal transduction	2.17E-03	9.08E-03	6	249	190	35980	P0CG47|P51452|P62987|P62979|P62158|P0CG48
GO:0002831	regulation of response to biotic stimulus	1.55E-03	6.90E-03	6	233	190	35980	P52179|P40925|P62987|Q16654|Q6P6C2|P10176
GO:0003007	heart morphogenesis	4.59E-09	5.96E-08	12	232	190	35980	O15273|Q14896|P10916|P19429|Q14192|P08590|P09493|P68032|P45379|P13533|P12883|P63316
GO:0003008	system process	2.48E-09	3.36E-08	34	2042	190	35980	P52179|P10916|P01160|P54296|P16615|P16860|Q01449|P19429|P68871|P09493|P23327|P62158|P45379|P00441|P02144|O15273|Q14896|P13639|O75116|P68133|P17661|P68032|P13533|P19105|P12883|O14958|P17540|P63173|P60660|P08590|P02511|P25116|Q8N335|P63316
GO:0003009	skeletal muscle contraction	1.07E-08	1.33E-07	6	30	190	35980	P02144|O15273|P19429|P13639|P12883|P63316
GO:0003012	muscle system process	1.48E-28	7.11E-27	30	319	190	35980	P52179|P10916|P01160|P54296|P16615|Q01449|P19429|P09493|P23327|P62158|P45379|P00441|P02144|O15273|Q14896|P13639|O75116|P68133|P17661|P68032|P13533|P19105|P12883|O14958|P17540|P60660|P08590|P02511|Q8N335|P63316
GO:0003013	circulatory system process	6.73E-12	1.06E-10	18	404	190	35980	O15273|Q14896|P10916|P01160|P68032|P13533|P12883|P16860|O14958|P19429|P68871|P08590|P09493|P45379|P25116|Q8N335|P00441|P63316
GO:0003015	heart process	2.21E-17	4.73E-16	14	85	190	35980	O15273|Q14896|P10916|P68032|P13533|P12883|O14958|P19429|P08590|P09493|P45379|Q8N335|P00441|P63316
GO:0003018	vascular process in circulatory system	3.47E-03	1.35E-02	5	190	190	35980	P16860|P01160|P68871|P25116|P00441
GO:0003073	regulation of systemic arterial blood pressure	1.53E-03	6.79E-03	4	93	190	35980	P19429|P01160|P09493|P25116
GO:0003205	cardiac chamber development	7.43E-10	1.03E-08	11	155	190	35980	Q13765|Q14896|P10916|P19429|Q14192|P08590|P09493|P45379|P13533|P12883|P63316
GO:0003206	cardiac chamber morphogenesis	3.29E-10	4.70E-09	10	108	190	35980	Q14896|P10916|P19429|Q14192|P08590|P09493|P45379|P13533|P12883|P63316
GO:0003208	cardiac ventricle morphogenesis	4.89E-11	7.34E-10	9	63	190	35980	Q14896|P10916|P19429|P08590|P09493|P45379|P13533|P12883|P63316
GO:0003229	ventricular cardiac muscle tissue development	4.52E-12	7.42E-11	9	49	190	35980	Q14896|P10916|P19429|P08590|P09493|P45379|P13533|P12883|P63316
GO:0003231	cardiac ventricle development	5.15E-10	7.26E-09	10	113	190	35980	Q13765|Q14896|P10916|P19429|P08590|P09493|P45379|P13533|P12883|P63316
GO:0005975	carbohydrate metabolic process	4.55E-03	1.71E-02	16	1451	190	35980	P0CG47|P13929|P40926|P04406|P40925|P0CG48|P05090|P00505|P07195|P17174|P62987|Q16654|P62979|P62158|Q8N335|P48735
GO:0005976	polysaccharide metabolic process	1.19E-04	7.48E-04	5	90	190	35980	P0CG47|P62987|P62979|P62158|P0CG48
GO:0005977	glycogen metabolic process	2.68E-05	2.05E-04	5	66	190	35980	P0CG47|P62987|P62979|P62158|P0CG48
GO:0005978	glycogen biosynthetic process	1.85E-05	1.45E-04	4	30	190	35980	P0CG47|P62987|P62979|P0CG48
GO:0005996	monosaccharide metabolic process	1.01E-08	1.27E-07	13	304	190	35980	P0CG47|P13929|P40926|P04406|P40925|P0CG48|P05090|P00505|P17174|P62987|Q16654|P62979|P62158
GO:0006006	glucose metabolic process	1.42E-11	2.20E-10	13	178	190	35980	P0CG47|P13929|P40926|P04406|P40925|P0CG48|P05090|P00505|P17174|P62987|Q16654|P62979|P62158
GO:0006073	cellular glucan metabolic process	2.88E-05	2.17E-04	5	67	190	35980	P0CG47|P62987|P62979|P62158|P0CG48
GO:0006082	organic acid metabolic process	3.55E-30	1.85E-28	56	1625	190	35980	P06732|Q9NSD9|P40926|P40925|P62280|P35613|P39019|P00505|P39023|P62273|P07195|P46783|P83731|P62913|P62753|P24752|Q07020|P04406|O14561|P19021|P84098|P63173|P61353|P46777|P05386|P60866|P46776|P05387|P62249|Q13011|P13929|P25398|P62891|P42766|Q02878|P17174|P61247|P62857|Q16654|Q16698|P62979|P62851|P08708|P62906|P18077|P51857|P42677|P41222|P15880|P17540|Q02543|P62263|P62987|P62945|P62269|P48735
GO:0006090	pyruvate metabolic process	2.26E-03	9.40E-03	5	172	190	35980	P13929|P04406|P07195|Q16654|P35613
GO:0006091	generation of precursor metabolites and energy	1.18E-36	1.06E-34	43	574	190	35980	P0CG47|P13929|O75947|P09669|O43674|P40926|P40925|O00483|P14854|P36542|P10176|O95178|P12235|P0CG48|O96000|P13073|P25705|P18859|O14949|P62979|P56381|P24310|P62158|P24311|Q8WYQ3|O15239|P20674|O00217|P10606|O95169|P51970|P48047|P04406|O14561|O43920|P15954|O95167|Q00325|P08574|P62987|P47985|P06576|P48735
GO:0006094	gluconeogenesis	2.75E-06	2.52E-05	6	74	190	35980	P13929|P00505|P40926|P04406|P40925|P17174
GO:0006099	tricarboxylic acid cycle	2.65E-03	1.06E-02	3	52	190	35980	P40926|P40925|P48735
GO:0006101	citrate metabolic process	3.27E-03	1.28E-02	3	56	190	35980	P40926|P40925|P48735

GO:0006103	2-oxoglutarate metabolic process	1.55E-04	9.48E-04	3	20	190	35980	P00505|P17174|P48735
GO:0006107	oxaloacetate metabolic process	2.42E-07	2.51E-06	4	11	190	35980	P00505|P40926|P40925|P17174
GO:0006112	energy reserve metabolic process	2.82E-04	1.60E-03	6	168	190	35980	P0CG47|P62987|P62979|P62158|P12235|P0CG48
GO:0006119	oxidative phosphorylation	5.13E-20	1.27E-18	16	93	190	35980	O00217|O95169|P51970|O43674|O14561|O00483|O43920|P36542|O95167|P10176|O95178|O96000|P13073|Q8WYQ3|O15239|P20674
GO:0006120	mitochondrial electron transport, NADH to ubiquinone	2.08E-15	4.14E-14	11	50	190	35980	O00217|O95169|P51970|O43674|O14561|O00483|O43920|O95167|O95178|O96000|O15239
GO:0006123	mitochondrial electron transport, cytochrome c to oxygen	1.69E-05	1.33E-04	3	10	190	35980	P13073|P10176|P20674
GO:0006139	nucleobase-containing compound metabolic process	3.68E-25	1.42E-23	98	6597	190	35980	O75947|O43674|O96000|P16860|P13073|P07195|P46783|P83731|P62913|P62753|O15239|P20674|Q13765|O95169|P48047|P04406|O14561|Q9Y5N6|P16989|Q9Y6H1|O95167|P84098|P63173|P68104|P46777|Q13772|P60866|P46776|P60228|P62249|Q8N335|P25398|P10176|O95178|P62891|P25705|P18859|Q14192|P56381|O00217|Q08493|P08708|P18077|Q6P6C2|P17535|P12883|Q8N8D1|P42677|P15880|Q02543|P62263|Q14240|P62945|P62269|P0CG47|Q9NSD9|P0DMV8|P0DMV9|P01160|P40926|P40925|P62280|Q8TAK5|P36542|P0CG48|P39019|P39023|P62273|P24311|Q8WYQ3|Q07020|P10606|Q96H40|O43920|P61353|P05386|P17081|P05387|P13929|P09669|O00483|P14854|P42766|Q02878|P84243|Q9NPI5|P61247|P62857|P62979|Q15004|P62851|P51970|P62906|P15954|P13533|P62987|P20585|P06576
GO:0006163	purine nucleotide metabolic process	1.25E-23	4.01E-22	32	561	190	35980	P13929|O75947|P0DMV8|P0DMV9|P01160|O43674|O00483|P36542|P10176|O95178|O96000|P16860|P13073|P25705|P18859|P56381|Q8WYQ3|O15239|P20674|O00217|Q08493|O95169|P51970|P48047|P04406|O14561|O43920|P13533|O95167|P12883|P17081|P06576
GO:0006164	purine nucleotide biosynthetic process	3.16E-07	3.24E-06	10	221	190	35980	O75947|P16860|P25705|P48047|P18859|P01160|P36542|P56381|Q8WYQ3|P06576
GO:0006283	transcription-coupled nucleotide-excision repair	6.17E-04	3.14E-03	4	73	190	35980	P0CG47|P62987|P62979|P0CG48
GO:0006289	nucleotide-excision repair	7.12E-03	2.37E-02	4	143	190	35980	P0CG47|P62987|P62979|P0CG48
GO:0006297	nucleotide-excision repair, DNA gap filling	6.17E-06	5.31E-05	4	23	190	35980	P0CG47|P62987|P62979|P0CG48
GO:0006301	postreplication repair	2.31E-05	1.79E-04	5	64	190	35980	P0CG47|P62987|P62979|Q15004|P0CG48
GO:0006352	DNA-templated transcription, initiation	8.68E-09	1.10E-07	14	359	190	35980	P0CG47|P10606|P09669|P01160|O00483|P14854|P15954|P10176|P0CG48|P13073|P62987|P62979|P24311|P20674
GO:0006364	rRNA processing	4.67E-08	5.28E-07	10	180	190	35980	P42766|P08708|P62263|P18077|P62857|P62913|P46777|P62753|P62249|P39019
GO:0006366	transcription from RNA polymerase II promoter	4.03E-05	2.90E-04	15	833	190	35980	P0CG47|P10606|P09669|P01160|O00483|P14854|P15954|P10176|P17535|P0CG48|P13073|P62987|P62979|P24311|P20674
GO:0006367	transcription initiation from RNA polymerase II promoter	3.10E-10	4.46E-09	14	277	190	35980	P0CG47|P10606|P09669|P01160|O00483|P14854|P15954|P10176|P0CG48|P13073|P62987|P62979|P24311|P20674
GO:0006396	RNA processing	1.58E-02	4.33E-02	12	1115	190	35980	P42766|P08708|P62263|P18077|P62857|P62913|P46777|Q6P6C2|P62753|P62249|P39019|Q8N8D1
GO:0006401	RNA catabolic process	2.19E-44	2.84E-42	38	248	190	35980	P25398|P62280|P39019|P62891|P42766|Q02878|P39023|P62273|P46783|P61247|P83731|P62857|P62913|P62979|P62851|P62753|Q07020|P08708|P62906|P18077|P42677|P84098|P15880|P63173|Q02543|P61353|P62263|P62987|Q14240|P62945|P46777|P05386|P60866|P46776|P62269|P05387|P60228|P62249
GO:0006402	mRNA catabolic process	9.90E-47	1.55E-44	38	217	190	35980	P25398|P62280|P39019|P62891|P42766|Q02878|P39023|P62273|P46783|P61247|P83731|P62857|P62913|P62979|P62851|P62753|Q07020|P08708|P62906|P18077|P42677|P84098|P15880|P63173|Q02543|P61353|P62263|P62987|Q14240|P62945|P46777|P05386|P60866|P46776|P62269|P05387|P60228|P62249
GO:0006412	translation	2.90E-31	1.63E-29	47	985	190	35980	P25398|Q9NSD9|P62280|P12235|P39019|P62891|P42766|Q02878|P39023|P62273|P46783|P61247|P83731|P62857|P62913|P62979|P62851|P62753|Q13765|Q07020|P13639|P08708|P62906|P18077|P37108|P42677|P84098|P15880|Q05639|P63173|P68104|Q02543|Q9NWT8|P61353|Q00325|P62263|P62987|Q14240|P62945|P46777|P05386|P60866|P46776|P62269|P05387|P60228|P62249
GO:0006413	translational initiation	2.69E-42	3.20E-40	39	306	190	35980	P25398|P62280|P39019|P62891|P42766|Q02878|P39023|P62273|P46783|P61247|P83731|P62857|P62913|P62979|P62851|P62753|Q07020|P08708|P62906|P18077|P42677|P84098|P15880|P63173|Q02543|Q9NWT8|P61353|P62263|P62987|Q14240|P62945|P46777|P05386|P60866|P46776|P62269|P05387|P60228|P62249
GO:0006414	translational elongation	1.19E-49	2.52E-47	40	223	190	35980	P25398|P62280|P39019|P62891|P42766|Q02878|P39023|P62273|P46783|P61247|P83731|P62857|P62913|P62979|P62851|P62753|Q07020|P13639|P08708|P62906|P18077|P42677|P84098|P15880|Q05639|P63173|P68104|Q02543|Q9NWT8|P61353|P62263|P62987|P62945|P46777|P05386|P60866|P46776|P62269|P05387|P62249
GO:0006415	translational termination	5.34E-48	8.83E-46	37	183	190	35980	P25398|P62280|P39019|P62891|P42766|Q02878|P39023|P62273|P46783|P61247|P83731|P62857|P62913|P62979|P62851|P62753|Q07020|P08708|P62906|P18077|P42677|P84098|P15880|P63173|Q02543|Q9NWT8|P61353|P62263|P62987|P62945|P46777|P05386|P60866|P46776|P62269|P05387|P62249
GO:0006417	regulation of translation	5.45E-04	2.82E-03	9	435	190	35980	P63244|P13639|P63173|P04406|P62263|Q14240|P16989|P04792|P60228
GO:0006461	protein complex assembly	1.70E-08	2.08E-07	26	1366	190	35980	O43674|O95178|P39019|O96000|P84243|P68871|P22352|Q16698|P69905|P45379|P24752|O15239|O00217|O15273|Q14896|O95169|P51970|O14561|P19021|P19022|O43920|O95167|O14958|Q9Y3D2|P02511|P04275
GO:0006518	peptide metabolic process	8.01E-30	4.10E-28	49	1183	190	35980	Q9NSD9|P62280|P39019|P39023|P62273|P46783|P83731|P62913|P62753|Q13765|Q07020|P13639|P19021|P37108|P84098|Q05639|P63173|P68104|P61353|P46777|P05386|P60866|P46776|P05387|P60228|P62249|P25398|P12235|P62891|P42766|Q02878|P61247|P62857|P62979|P62851|P00441|P08708|P62906|P18077|P42677|P15880|Q02543|Q9NWT8|Q00325|P62263|P62987|Q14240|P62945|P62269
GO:0006520	cellular amino acid metabolic process	2.61E-31	1.49E-29	42	722	190	35980	P06732|P25398|Q9NSD9|P62280|P39019|P62891|P42766|Q02878|P00505|P39023|P62273|P17174|P46783|P61247|P83731|P62857|P62913|P62979|P62851|P62753|P24752|Q07020|P08708|P62906|P18077|P42677|P84098|P15880|P17540|P63173|Q02543|P61353|P62263|P62987|P62945|P46777|P05386|P60866|P46776|P62269|P05387|P62249
GO:0006575	cellular modified amino acid metabolic process	3.29E-39	3.50E-37	40	397	190	35980	P06732|P25398|P62280|P39019|P62891|P42766|Q02878|P00505|P39023|P62273|P46783|P61247|P83731|P62857|P62913|P62979|P62851|P62753|P00441|Q07020|P08708|P62906|P18077|P42677|P84098|P15880|P17540|P63173|Q02543|P61353|P62263|P62987|P62945|P46777|P05386|P60866|P46776|P62269|P05387|P62249
GO:0006595	polyamine metabolic process	1.71E-03	7.50E-03	4	96	190	35980	P54368|P06732|P17540|P17174
GO:0006605	protein targeting	2.78E-37	2.67E-35	41	479	190	35980	P25398|P62280|P35613|P39019|P62891|P42766|Q02878|P39023|P62273|P46783|P61247|P83731|P62857|P62913|P62979|P62851|P62753|Q07020|Q9P0U1|P08708|P62906|P18077|P37108|Q9Y6H1|P42677|P84098|P15880|P63173|Q02543|P61353|P62263|P62987|P62945|P46777|P05386|P60866|P46776|P62269|P05387|P25116|P62249
GO:0006612	protein targeting to membrane	1.73E-49	3.44E-47	38	186	190	35980	P25398|P62280|P35613|P39019|P62891|P42766|Q02878|P39023|P62273|P46783|P61247|P83731|P62857|P62913|P62979|P62851|P62753|Q07020|P08708|P62906|P18077|P37108|P42677|P84098|P15880|P63173|Q02543|P61353|P62263|P62987|P62945|P46777|P05386|P60866|P46776|P62269|P05387|P62249
GO:0006613	cotranslational protein targeting to membrane	9.45E-55	3.51E-52	37	126	190	35980	P25398|P62280|P39019|P62891|P42766|Q02878|P39023|P62273|P46783|P61247|P83731|P62857|P62913|P62979|P62851|P62753|Q07020|P08708|P62906|P18077|P37108|P42677|P84098|P15880|P63173|Q02543|P61353|P62263|P62987|P62945|P46777|P05386|P60866|P46776|P62269|P05387|P62249
GO:0006614	SRP-dependent cotranslational protein targeting to membrane	3.34E-55	1.98E-52	37	123	190	35980	P25398|P62280|P39019|P62891|P42766|Q02878|P39023|P62273|P46783|P61247|P83731|P62857|P62913|P62979|P62851|P62753|Q07020|P08708|P62906|P18077|P37108|P42677|P84098|P15880|P63173|Q02543|P61353|P62263|P62987|P62945|P46777|P05386|P60866|P46776|P62269|P05387|P62249
GO:0006725	cellular aromatic compound metabolic process	3.26E-24	1.13E-22	99	6919	190	35980	O75947|O43674|O96000|P16860|P13073|P07195|P46783|P83731|P62913|P62753|O15239|P20674|Q13765|O95169|P48047|P04406|O14561|Q9Y5N6|P16989|Q9Y6H1|O95167|P84098|P63173|P68104|P46777|Q13772|P60866|P46776|P60228|P62249|Q8N335|P25398|P10176|O95178|P62891|P25705|P18859|Q14192|P56381|O00217|Q08493|P08708|P18077|Q6P6C2|P17535|P12883|Q8N8D1|P42677|P15880|Q02543|P62263|Q14240|P62945|P62269|P0CG47|Q9NSD9|P0DMV8|P0DMV9|P01160|P40926|P40925|P62280|Q8TAK5|P36542|P0CG48|P39019|P00505|P39023|P62273|P24311|Q8WYQ3|Q07020|P10606|Q96H40|O43920|P61353|P05386|P17081|P05387|P13929|P09669|O00483|P14854|P42766|Q02878|P84243|Q9NPI5|P61247|P62857|P62979|Q15004|P62851|P51970|P62906|P15954|P13533|P62987|P20585|P06576
GO:0006732	coenzyme metabolic process	1.40E-02	4.02E-02	7	478	190	35980	P13929|P40926|P04406|Q9NPI5|P07195|P40925|Q8N335
GO:0006733	oxidoreduction coenzyme metabolic process	4.47E-04	2.39E-03	7	256	190	35980	P13929|P40926|P04406|Q9NPI5|P07195|P40925|Q8N335
GO:0006734	NADH metabolic process	1.48E-06	1.41E-05	5	37	190	35980	P13929|P40926|P04406|P40925|Q8N335

<table>
<tr><td>GO:0006753</td><td>nucleoside phosphate metabolic process</td><td>6.37E-24</td><td>2.06E-22</td><td>37</td><td>809</td><td>190</td><td>35980</td><td>P13929|O75947|P0DMV8|P0DMV9|P01160|O43674|P40926|P40925|O00483|P36542|P10176|O95178|O96000|P16860|P13073|P25705|P18859|Q9NPI5|P07195|P56381|Q8WYQ3|O15239|P20674|O00217|Q08493|O95169|P51970|P48047|P04406|O14561|O43920|P13533|O95167|P12883|P17081|Q8N335|P06576</td></tr>
<tr><td>GO:0006754</td><td>ATP biosynthetic process</td><td>5.96E-10</td><td>8.36E-09</td><td>8</td><td>56</td><td>190</td><td>35980</td><td>O75947|P25705|P48047|P18859|P36542|P56381|Q8WYQ3|P06576</td></tr>
<tr><td>GO:0006793</td><td>phosphorus metabolic process</td><td>6.88E-09</td><td>8.78E-08</td><td>50</td><td>4056</td><td>190</td><td>35980</td><td>P0CG47|O75947|P06732|P0DMV8|P0DMV9|P01160|O43674|P40926|P40925|P36542|P0CG48|O96000|P16860|P13073|P07195|P62158|Q8WYQ3|O15239|P20674|O95169|P48047|P04406|O14561|O43920|O95167|P02511|P17081|Q8N335|P13929|O00483|P10176|O95178|P25705|P18859|Q9NPI5|Q16654|P62979|P56381|P51452|O00217|Q14116|Q08493|P51970|O75116|P13533|P12883|P17540|P62987|P25116|P06576</td></tr>
<tr><td>GO:0006796</td><td>phosphate-containing compound metabolic process</td><td>3.67E-09</td><td>4.78E-08</td><td>50</td><td>3980</td><td>190</td><td>35980</td><td>P0CG47|O75947|P06732|P0DMV8|P0DMV9|P01160|O43674|P40926|P40925|P36542|P0CG48|O96000|P16860|P13073|P07195|P62158|Q8WYQ3|O15239|P20674|O95169|P48047|P04406|O14561|O43920|O95167|P02511|P17081|Q8N335|P13929|O00483|P10176|O95178|P25705|P18859|Q9NPI5|Q16654|P62979|P56381|P51452|O00217|Q14116|Q08493|P51970|O75116|P13533|P12883|P17540|P62987|P25116|P06576</td></tr>
<tr><td>GO:0006807</td><td>nitrogen compound metabolic process</td><td>5.01E-27</td><td>2.26E-25</td><td>115</td><td>8603</td><td>190</td><td>35980</td><td>O75947|O43674|O96000|P16860|P13073|P07195|P46783|P83731|P62913|P62753|P24752|O15239|P20674|Q13765|O95169|P13639|P48047|P04406|O14561|Q9Y5N6|P16989|Q9Y6H1|O95167|P84098|P63173|P68104|P46777|Q13772|P60866|P46776|P60228|P62249|Q8N335|P07602|P25398|P10176|O95178|P12235|P62891|P25705|P18859|Q14192|P56381|P00441|P54368|O00217|Q08493|P08708|P18077|Q6P6C2|P17535|P12883|Q8N8D1|P42677|P15880|P17540|Q02543|Q9NWT8|P62263|Q14240|P62945|P62269|P0CG47|P06732|Q9NSD9|P0DMV8|P0DMV9|P01160|P40926|P40925|P62280|Q8TAK5|P36542|P0CG48|P39019|P00505|P39023|P62273|P62158|P24311|Q8WYQ3|Q07020|P10606|Q96H40|O14880|P19021|P37108|O43920|Q05639|P61353|P05386|P17081|P05387|P13929|P09669|O00483|P14854|P42766|Q02878|P84243|Q9NPI5|P17174|P61247|P62857|P62979|Q15004|P62851|P51970|P62906|P15954|P13533|Q00325|P62987|P20585|P06576</td></tr>
<tr><td>GO:0006810</td><td>transport</td><td>1.36E-18</td><td>3.12E-17</td><td>90</td><td>6916</td><td>190</td><td>35980</td><td>P0CG47|O75947|P0DMV8|P16615|P62280|P36542|P35613|P0CG48|P39019|P16860|P05090|P13073|P00505|O14949|P39023|P62273|P46783|P83731|P62913|P24310|P69905|P62158|P24311|P62753|P20674|Q13765|Q07020|P01033|P10606|P48047|Q9Y277|P19021|P14406|P37108|Q9Y6H1|Q08431|P84098|O14958|P63173|P61353|P47985|P46777|P05386|P60866|P46776|P05387|P62249|P07602|P02792|P25398|P09669|P68363|O00483|P14854|P10176|P12235|P13693|P62891|P42766|Q02878|P25705|P18859|P60033|P68871|P61247|P62857|P62979|P56381|P62851|P00441|P54368|P02144|Q9P0U1|P08708|P62906|P18077|P15954|Q6P6C2|P42677|P41222|P15880|Q02543|Q00325|P62263|P62987|P62945|P04275|P62269|P25116|P06576</td></tr>
<tr><td>GO:0006811</td><td>ion transport</td><td>1.47E-07</td><td>1.56E-06</td><td>34</td><td>2414</td><td>190</td><td>35980</td><td>P0CG47|O75947|P02792|P09669|O00483|P14854|P16615|P36542|P10176|P0CG48|P13693|P13073|P25705|P00505|P18859|O14949|P68871|P62979|P56381|P24310|P69905|P24311|P20674|P10606|P48047|Q9Y277|P14406|P15954|O14958|Q00325|P62987|P47985|P25116|P06576</td></tr>
<tr><td>GO:0006812</td><td>cation transport</td><td>1.49E-06</td><td>1.41E-05</td><td>24</td><td>1503</td><td>190</td><td>35980</td><td>O75947|P10606|P02792|P09669|P48047|P14406|O00483|P14854|P16615|P36542|P15954|P10176|P13693|P13073|P25705|P18859|O14949|P47985|P56381|P24310|P24311|P25116|P06576|P20674</td></tr>
<tr><td>GO:0006818</td><td>hydrogen transport</td><td>1.88E-16</td><td>3.89E-15</td><td>20</td><td>298</td><td>190</td><td>35980</td><td>O75947|P10606|P09669|P48047|P14406|O00483|P14854|P36542|P15954|P10176|P13073|P25705|P18859|O14949|P47985|P56381|P24310|P24311|P06576|P20674</td></tr>
<tr><td>GO:0006839</td><td>mitochondrial transport</td><td>2.45E-07</td><td>2.54E-06</td><td>10</td><td>215</td><td>190</td><td>35980</td><td>O75947|P0DMV8|P25705|Q9P0U1|P48047|P18859|P36542|Q9Y6H1|P56381|P06576</td></tr>
<tr><td>GO:0006873</td><td>cellular ion homeostasis</td><td>5.79E-04</td><td>2.96E-03</td><td>10</td><td>533</td><td>190</td><td>35980</td><td>P13693|P02792|P51684|O14958|P19429|P16615|P23327|P25116|P00441|P06576</td></tr>
<tr><td>GO:0006874</td><td>cellular calcium ion homeostasis</td><td>1.67E-03</td><td>7.33E-03</td><td>7</td><td>321</td><td>190</td><td>35980</td><td>P13693|P51684|O14958|P19429|P16615|P23327|P25116</td></tr>
<tr><td>GO:0006875</td><td>cellular metal ion homeostasis</td><td>9.41E-04</td><td>4.57E-03</td><td>9</td><td>470</td><td>190</td><td>35980</td><td>P13693|P02792|P51684|O14958|P19429|P16615|P23327|P25116|P00441</td></tr>
<tr><td>GO:0006886</td><td>intracellular protein transport</td><td>7.28E-23</td><td>2.14E-21</td><td>41</td><td>1120</td><td>190</td><td>35980</td><td>P25398|P62280|P35613|P39019|P62891|P42766|Q02878|P39023|P62273|P46783|P61247|P83731|P62857|P62913|P62979|P62851|P62753|Q07020|Q9P0U1|P08708|P62906|P18077|P37108|Q9Y6H1|P42677|P84098|P15880|P63173|Q02543|P61353|P62263|P62987|P62945|P46777|P05386|P60866|P46776|P62269|P05387|P25116|P62249</td></tr>
<tr><td>GO:0006914</td><td>autophagy</td><td>1.02E-04</td><td>6.52E-04</td><td>10</td><td>428</td><td>190</td><td>35980</td><td>P0CG47|P52179|Q9P0U1|P40925|P62987|Q16654|P62979|Q6P6C2|P10176|P0CG48</td></tr>
<tr><td>GO:0006915</td><td>apoptotic process</td><td>4.33E-03</td><td>1.64E-02</td><td>14</td><td>1185</td><td>190</td><td>35980</td><td>P0CG47|P04406|Q9H3K2|P68032|P12235|P0CG48|Q8N8D1|P63244|P09382|P62987|P62979|P02511|P25116|P01034</td></tr>
<tr><td>GO:0006928</td><td>movement of cell or subcellular component</td><td>6.42E-08</td><td>7.07E-07</td><td>30</td><td>1882</td><td>190</td><td>35980</td><td>P0CG47|P10916|P51684|P35613|P0CG48|P39019|P19429|P09493|P83731|P62979|P62158|P45379|P00441|O15273|Q14896|O75116|P19022|P68133|P17661|P68032|P13533|P19105|P12883|P60660|P08590|P62987|P04792|Q8N335|P06576|P63316</td></tr>
<tr><td>GO:0006935</td><td>chemotaxis</td><td>7.38E-03</td><td>2.44E-02</td><td>11</td><td>880</td><td>190</td><td>35980</td><td>P0CG47|P51684|P60660|O75116|P62987|P83731|P62979|P62158|P19105|P0CG48|P39019</td></tr>
<tr><td>GO:0006936</td><td>muscle contraction</td><td>9.35E-27</td><td>4.09E-25</td><td>27</td><td>264</td><td>190</td><td>35980</td><td>P52179|P10916|P54296|Q01449|P19429|P09493|P23327|P62158|P45379|P02144|O15273|Q14896|P13639|O75116|P68133|P17661|P68032|P13533|P19105|P12883|O14958|P17540|P60660|P08590|P02511|Q8N335|P63316</td></tr>
<tr><td>GO:0006937</td><td>regulation of muscle contraction</td><td>2.42E-15</td><td>4.80E-14</td><td>16</td><td>179</td><td>190</td><td>35980</td><td>Q14896|P10916|P01160|P16615|P12883|O14958|P19429|P08590|P09493|P23327|P62158|P45379|P25116|O14558|P00441|P63316</td></tr>
<tr><td>GO:0006940</td><td>regulation of smooth muscle contraction</td><td>6.62E-03</td><td>2.24E-02</td><td>3</td><td>72</td><td>190</td><td>35980</td><td>P19429|P25116|P00441</td></tr>
<tr><td>GO:0006941</td><td>striated muscle contraction</td><td>7.91E-18</td><td>1.74E-16</td><td>15</td><td>101</td><td>190</td><td>35980</td><td>P02144|O15273|Q14896|P10916|P13639|P68032|P13533|P12883|O14958|P19429|P08590|P09493|P45379|Q8N335|P63316</td></tr>
<tr><td>GO:0006942</td><td>regulation of striated muscle contraction</td><td>6.20E-10</td><td>8.65E-09</td><td>9</td><td>83</td><td>190</td><td>35980</td><td>Q14896|P10916|O14958|P01160|P08590|P16615|P23327|P62158|P12883</td></tr>
<tr><td>GO:0006950</td><td>response to stress</td><td>2.36E-08</td><td>2.77E-07</td><td>53</td><td>4615</td><td>190</td><td>35980</td><td>P0CG47|P0DMV8|P0DMV9|Q12988|P01160|P40925|P16615|P35613|P0CG48|P05090|P09493|P69905|P62158|P24752|P01034|Q13765|P01033|P13639|P04406|P19021|P16989|P19105|P09382|P02511|P07602|P52179|P13929|P51684|P10176|P62891|P84243|P60033|P68871|Q9UBY9|Q16654|P22352|P62979|Q15004|Q01628|P00441|P51452|O00217|P02144|Q14116|O15273|Q9P0U1|Q6P6C2|Q9Y3D2|P62987|P04275|P04792|P25116|P20585</td></tr>
<tr><td>GO:0006952</td><td>defense response</td><td>1.52E-02</td><td>4.33E-02</td><td>18</td><td>1947</td><td>190</td><td>35980</td><td>P0CG47|P51452|P52179|Q14116|P51684|P04406|P40925|Q6P6C2|P10176|P0CG48|P62891|P62987|Q16654|P62979|P62158|Q01628|P25116|P01034</td></tr>
<tr><td>GO:0006977</td><td>DNA damage response, signal transduction by p53 class mediator resulting in cell cycle arrest</td><td>4.20E-04</td><td>2.27E-03</td><td>4</td><td>66</td><td>190</td><td>35980</td><td>P0CG47|P62987|P62979|P0CG48</td></tr>
<tr><td>GO:0006979</td><td>response to oxidative stress</td><td>2.80E-08</td><td>3.27E-07</td><td>15</td><td>461</td><td>190</td><td>35980</td><td>O00217|P02144|P0DMV8|P13639|P0DMV9|P16615|P05090|Q9Y3D2|P68871|P09493|P22352|P02511|P69905|P00441|P01034</td></tr>
<tr><td>GO:0006996</td><td>organelle organization</td><td>3.70E-11</td><td>5.62E-10</td><td>58</td><td>4481</td><td>190</td><td>35980</td><td>P0CG47|P10916|P0DMV8|O43674|P40925|P16615|P0CG48|P39019|O96000|P39023|P09493|P46783|P83731|P62913|P45379|Q8WYQ3|O15239|Q14896|O95169|P04406|O14561|O43920|Q9Y6H1|P68032|O95167|O14958|P63173|P46777|P02511|P17081|P52179|P68363|P10176|O95178|P12235|Q02878|P84243|P62979|Q15004|P00441|O00217|O15273|P51970|Q9P0U1|P08708|O75116|P68133|P17661|P13533|P42677|Q9NWT8|Q9Y3D2|P62263|P62987|P63313|P25116|P20585|P06576</td></tr>
<tr><td>GO:0007005</td><td>mitochondrion organization</td><td>2.58E-13</td><td>4.56E-12</td><td>24</td><td>679</td><td>190</td><td>35980</td><td>P0CG47|P52179|O00217|O95169|P0DMV8|P51970|Q9P0U1|O43674|O14561|P40925|O43920|Q9Y6H1|O95167|P10176|O95178|P12235|P0CG48|O96000|Q9NWT8|P62987|P62979|Q8WYQ3|P06576|O15239</td></tr>
<tr><td>GO:0007009</td><td>plasma membrane organization</td><td>1.28E-02</td><td>3.76E-02</td><td>5</td><td>261</td><td>190</td><td>35980</td><td>O75116|P19022|P16615|P35613|P00441</td></tr>
<tr><td>GO:0007010</td><td>cytoskeleton organization</td><td>3.37E-04</td><td>1.88E-03</td><td>19</td><td>1479</td><td>190</td><td>35980</td><td>O15273|Q14896|P10916|P04406|O75116|P68363|P68133|P17661|P68032|P13533|O14958|Q9Y3D2|P09493|P02511|Q15004|P63313|P17081|P45379|P00441</td></tr>
<tr><td>GO:0007015</td><td>actin filament organization</td><td>1.28E-02</td><td>3.77E-02</td><td>6</td><td>362</td><td>190</td><td>35980</td><td>O15273|Q9Y3D2|P68133|P68032|P63313|P45379</td></tr>
<tr><td>GO:0007093</td><td>mitotic cell cycle checkpoint</td><td>3.47E-03</td><td>1.35E-02</td><td>5</td><td>190</td><td>190</td><td>35980</td><td>P0CG47|P62987|P83731|P62979|P0CG48</td></tr>
<tr><td>GO:0007167</td><td>enzyme linked receptor protein signaling pathway</td><td>7.18E-04</td><td>3.62E-03</td><td>16</td><td>1207</td><td>190</td><td>35980</td><td>P0CG47|P51452|P01160|O75116|P13533|P19105|P0CG48|P16860|P60660|P62987|Q16654|P62979|P62158|P17081|P04792|P62753</td></tr>
<tr><td>GO:0007169</td><td>transmembrane receptor protein tyrosine kinase signaling pathway</td><td>1.33E-03</td><td>6.10E-03</td><td>13</td><td>923</td><td>190</td><td>35980</td><td>P0CG47|P51452|O75116|P19105|P0CG48|P60660|P62987|Q16654|P62979|P62158|P17081|P04792|P62753</td></tr>
<tr><td>GO:0007179</td><td>transforming growth factor beta receptor signaling</td><td>1.31E-02</td><td>3.82E-02</td><td>4</td><td>171</td><td>190</td><td>35980</td><td>P0CG47|P62987|P62979|P0CG48</td></tr>
<tr><td>GO:0007219</td><td>Notch signaling pathway</td><td>3.61E-04</td><td>1.99E-03</td><td>6</td><td>176</td><td>190</td><td>35980</td><td>P0CG47|P17174|P62987|P62979|P0CG48|P39019</td></tr>
<tr><td>GO:0007220</td><td>Notch receptor processing</td><td>6.17E-06</td><td>5.31E-05</td><td>4</td><td>23</td><td>190</td><td>35980</td><td>P0CG47|P62987|P62979|P0CG48</td></tr>
<tr><td>GO:0007249</td><td>I-kappaB kinase/NF-kappaB signaling</td><td>4.37E-05</td><td>3.12E-04</td><td>5</td><td>73</td><td>190</td><td>35980</td><td>P0CG47|O75116|P62987|P62979|P0CG48</td></tr>
<tr><td>GO:0007254</td><td>JNK cascade</td><td>1.30E-03</td><td>6.00E-03</td><td>4</td><td>89</td><td>190</td><td>35980</td><td>P0CG47|P62987|P62979|P0CG48</td></tr>
<tr><td>GO:0007265</td><td>Ras protein signal transduction</td><td>1.18E-02</td><td>3.48E-02</td><td>6</td><td>355</td><td>190</td><td>35980</td><td>P0CG47|O75116|P62987|P62979|P62158|P0CG48</td></tr>
</table>

GO:0007275	multicellular organism development	1.72E-07	1.82E-06	55	5183	190	35980	P0CG47|P10916|P01160|P35613|P0CG48|P39019|P63244|P05090|O14949|P09493|P83731|P62158|P45379|P24311|P24752|P01034|Q13765|P01033|Q14896|P13639|P19021|P19022|P16989|P68032|Q08431|P19105|P63173|P08493|P60660|P09382|P02511|Q13772|P07602|P14854|P19429|P25705|P84243|P18859|Q14192|P62979|P00441|P02144|Q14116|O15273|O75116|P68133|P13533|P12883|P08590|P62263|P62987|P04275|P20585|P06576|P63316
GO:0007399	nervous system development	1.81E-02	4.91E-02	22	2582	190	35980	P0CG47|P13639|O75116|P19021|P19022|P14854|P19105|P0CG48|P05090|P84243|P18859|O14949|P60660|P09382|P62987|P83731|P62979|P62158|P24311|P24752|P00441|P01034
GO:0007409	axonogenesis	1.30E-02	3.82E-02	9	705	190	35980	P0CG47|P60660|O75116|P62987|P83731|P62979|P62158|P19105|P0CG48
GO:0007411	axon guidance	4.48E-03	1.68E-02	9	593	190	35980	P0CG47|P60660|O75116|P62987|P83731|P62979|P62158|P19105|P0CG48
GO:0007417	central nervous system development	5.11E-03	1.85E-02	12	957	190	35980	P0CG47|P05090|P84243|P18859|O14949|P19021|P19022|P14854|P62158|P24311|P24752|P01034
GO:0007420	brain development	6.06E-03	2.07E-02	10	737	190	35980	P0CG47|P05090|P84243|P18859|O14949|P19022|P14854|P62158|P24752|P01034
GO:0007507	heart development	3.22E-08	3.74E-07	16	537	190	35980	Q13765|P02144|O15273|Q14896|P10916|P01160|P19021|P68032|P13533|P12883|P19429|Q14192|P08590|P09493|P45379|P63316
GO:0007512	adult heart development	7.72E-05	5.20E-04	3	16	190	35980	O15273|P13533|P12883
GO:0007517	muscle organ development	8.22E-11	1.22E-09	15	300	190	35980	O15273|Q14896|P10916|P13639|P68133|P68032|P13533|P12883|P19429|P60660|P08590|P09493|P02511|P45379|P63316
GO:0007519	skeletal muscle tissue development	5.83E-03	2.01E-02	4	135	190	35980	P13639|P60660|P08590|P68133
GO:0007565	female pregnancy	4.40E-03	1.66E-02	5	201	190	35980	P01160|P19021|P35613|P00441|P01034
GO:0007566	embryo implantation	1.86E-03	8.00E-03	3	46	190	35980	P35613|P00441|P01034
GO:0007568	aging	7.91E-04	3.92E-03	7	282	190	35980	P01033|P13929|P05090|P13639|P02511|P17535|P00441
GO:0007596	blood coagulation	3.41E-05	2.54E-04	12	541	190	35980	P07602|P01033|P84243|P68871|P16615|P04275|P62158|P04792|P35613|P19105|P25116|P00441
GO:0007599	hemostasis	3.73E-05	2.73E-04	12	546	190	35980	P07602|P01033|P84243|P68871|P16615|P04275|P62158|P04792|P35613|P19105|P25116|P00441
GO:0007623	circadian rhythm	2.24E-04	1.30E-03	6	161	190	35980	P0CG47|P62987|P62979|P17535|P0CG48|P01034
GO:0008015	blood circulation	5.95E-12	9.50E-11	18	401	190	35980	O15273|Q14896|P10916|P01160|P68032|P13533|P12883|P16860|O14958|P19429|P68871|P08590|P09493|P45379|P25116|Q8N335|P00441|P63316
GO:0008016	regulation of heart contraction	2.90E-13	5.04E-12	16	243	190	35980	Q14896|P10916|P01160|P16615|P17661|P13533|P12883|O14958|P19429|Q9UBY9|P08590|P09493|P23327|P62158|P45379|Q8N335
GO:0008104	protein localization	2.01E-14	3.83E-13	46	2432	190	35980	P25398|Q969T9|P62280|P35613|P39019|P62891|P42766|Q02878|P60033|P39023|P62273|P46783|P61247|P83731|P62857|P62913|P62979|P62851|P62753|Q13765|Q07020|Q9P0U1|P08708|O75116|P62906|P19022|P18077|P37108|Q9Y6H1|P42677|P84098|P15880|P63173|Q02543|P61353|P62263|P62987|P62945|P46777|P05386|P60866|P46776|P62269|P05387|P25116|P62249
GO:0008152	metabolic process	1.65E-10	2.39E-09	148	20093	190	35980	O75947|O43674|P16615|O96000|P16860|P13073|O14949|P07195|P46783|P83731|P62913|P62753|P24752|O15239|P20674|Q13765|O95169|P13639|P48047|P04406|O14561|P80297|Q9Y5N6|P16989|Q9Y6H1|O95167|Q08431|P84098|P63173|P68104|P08574|P47985|P46777|Q13772|P60866|P46776|P60228|P62249|Q13011|Q8N335|P07602|P52179|P25398|P68363|P10176|O95178|P12235|P62891|P25705|P18859|Q14192|P68871|Q16654|P56381|P00441|P54368|O00217|Q14116|Q08493|P08708|O75116|P18077|Q6P6C2|P17535|P12883|Q8N8D1|P42677|P41222|P15880|P17540|Q02543|Q9NWT8|Q9Y3D2|P62263|Q14240|P62945|P62269|P0CG47|P06732|Q9NSD9|P0DMV8|P0DMV9|P01160|P40926|P40925|P62280|Q8TAK5|P36542|P35613|P0CG48|P39019|P63244|P05090|P00505|P39023|P62273|P24310|P69905|P62158|P24311|Q8WYQ3|P01034|Q07020|P10606|Q96H40|O14880|P19021|P37108|O43920|Q05639|P61353|P02511|P05386|P17081|P05387|P13929|P09669|O00483|P14854|P42766|Q02878|P84243|P60033|Q9NPI5|P17174|P61247|P62857|P22352|Q16698|P62979|Q15004|P62851|P51452|P51970|Q9P0U1|P62906|P15954|P51857|P13533|Q00325|P08590|P62987|P04275|P04792|P25116|P20585|P06576|P48735
GO:0008217	regulation of blood pressure	7.43E-06	6.20E-05	8	187	190	35980	P16860|P19429|P01160|P68871|P09493|P13533|P25116|P00441
GO:0008219	cell death	7.25E-03	2.41E-02	14	1259	190	35980	P0CG47|P04406|Q9H3K2|P68032|P12235|P0CG48|Q8N8D1|P63244|P09382|P62987|P62979|P02511|P25116|P01034
GO:0008286	insulin receptor signaling pathway	2.33E-04	1.35E-03	8	305	190	35980	P0CG47|P62987|Q16654|P62979|P62158|P17081|P62753|P0CG48
GO:0008406	gonad development	7.84E-03	2.57E-02	5	231	190	35980	P0CG47|P16989|Q13772|P00441|P01034
GO:0008584	male gonad development	6.29E-03	2.14E-02	4	138	190	35980	P0CG47|P16989|Q13772|P01034
GO:0009056	catabolic process	3.68E-24	1.24E-22	58	2321	190	35980	P0CG47|P40925|P62280|P0CG48|P39019|P00505|P39023|P62273|P46783|P83731|P62913|P69905|P62158|P62753|P24752|Q07020|P04406|P84098|P63173|P61353|P46777|P05386|P60866|P46776|P05387|P60228|P62249|Q13011|Q8N335|P52179|P13929|P25398|P10176|P62891|P42766|Q02878|P68871|P17174|P61247|P62857|P22352|Q16698|P62979|P62851|Q08493|Q9P0U1|P08708|P62906|P18077|P51857|P42677|P15880|Q02543|P62263|P62987|Q14240|P62945|P62269
GO:0009057	macromolecule catabolic process	7.48E-22	2.04E-20	41	1193	190	35980	P0CG47|P25398|P62280|P0CG48|P39019|P62891|P42766|Q02878|P39023|P62273|P46783|P61247|P83731|P62857|P62913|P62979|P62158|P62851|P62753|Q07020|P08708|P62906|P18077|P42677|P84098|P15880|P63173|Q02543|P61353|P62263|P62987|Q14240|P62945|P46777|P05386|P60866|P46776|P62269|P05387|P60228|P62249
GO:0009058	biosynthetic process	8.64E-21	2.25E-19	94	6964	190	35980	P0CG47|O75947|P06732|Q9NSD9|P01160|P40926|P40925|P62280|Q8TAK5|P36542|P0CG48|P39019|P16860|P13073|P00505|P39023|P62273|P46783|P83731|P62913|P24311|P62753|Q8WYQ3|P24752|P20674|Q13765|Q07020|P10606|Q96H40|P13639|P48047|P04406|O14561|O14880|Q9Y5N6|P37108|P16989|Q9Y6H1|P84098|Q05639|P63173|P68104|P61353|P46777|P05386|Q13772|P60866|P46776|P05387|P60228|P62249|Q8N335|P13929|P25398|P09669|O00483|P14854|P10176|P12235|P62891|P42766|Q02878|P25705|P18859|Q14192|Q9NPI5|P17174|P61247|P62857|P62979|P56381|Q15004|P62851|P00441|P54368|Q14116|P08708|P62906|P18077|P15954|P51857|P17535|P42677|P41222|P15880|Q02543|Q9NWT8|Q00325|P62263|P62987|Q14240|P62945|P62269|P06576
GO:0009059	macromolecule biosynthetic process	5.89E-13	1.01E-11	68	5325	190	35980	P0CG47|Q9NSD9|P01160|P62280|Q8TAK5|P0CG48|P39019|P13073|P39023|P62273|P46783|P83731|P62913|P24311|P62753|P20674|Q13765|Q07020|P10606|Q96H40|P13639|Q9Y5N6|P37108|P16989|Q9Y6H1|P84098|Q05639|P63173|P68104|P61353|P46777|P05386|Q13772|P60866|P46776|P05387|P60228|P62249|P25398|P09669|O00483|P14854|P10176|P12235|P62891|P42766|Q02878|Q14192|P61247|P62857|P62979|Q15004|P62851|P08708|P62906|P18077|P15954|P17535|P42677|P15880|Q02543|Q9NWT8|Q00325|P62263|P62987|Q14240|P62945|P62269
GO:0009060	aerobic respiration	8.71E-04	4.27E-03	4	80	190	35980	P13073|P40926|P40925|P48735
GO:0009069	serine family amino acid metabolic process	3.86E-51	8.83E-49	36	138	190	35980	P25398|P62280|P39019|P62891|P42766|Q02878|P39023|P62273|P46783|P61247|P83731|P62857|P62913|P62979|P62851|P62753|Q07020|P08708|P62906|P18077|P42677|P84098|P15880|P63173|Q02543|P61353|P62263|P62987|P62945|P46777|P05386|P60866|P46776|P62269|P05387|P62249
GO:0009116	nucleoside metabolic process	1.22E-20	3.16E-19	29	545	190	35980	P13929|O75947|P0DMV8|P0DMV9|O43674|O00483|P36542|P10176|O95178|O96000|P13073|P25705|P18859|P56381|Q8WYQ3|O15239|P20674|O00217|O95169|P51970|P48047|P04406|O14561|O43920|P13533|O95167|P12883|P17081|P06576
GO:0009117	nucleotide metabolic process	3.06E-24	1.07E-22	37	792	190	35980	P13929|O75947|P0DMV8|P0DMV9|P01160|O43674|P40926|P40925|O00483|P36542|P10176|O95178|O96000|P16860|P13073|P25705|P18859|Q9NPI5|P07195|P56381|Q8WYQ3|O15239|P20674|O00217|Q08493|O95169|P51970|P48047|P04406|O14561|O43920|P13533|O95167|P12883|P17081|Q8N335|P06576
GO:0009119	ribonucleoside metabolic process	1.50E-21	4.05E-20	29	505	190	35980	P13929|O75947|P0DMV8|P0DMV9|O43674|O00483|P36542|P10176|O95178|O96000|P13073|P25705|P18859|P56381|Q8WYQ3|O15239|P20674|O00217|O95169|P51970|P48047|P04406|O14561|O43920|P13533|O95167|P12883|P17081|P06576
GO:0009123	nucleoside monophosphate metabolic process	4.63E-23	1.38E-21	28	403	190	35980	P13929|O75947|P0DMV8|P0DMV9|O43674|O00483|P36542|P10176|O95178|O96000|P13073|P25705|P18859|P56381|Q8WYQ3|O15239|P20674|O00217|O95169|P51970|P48047|P04406|O14561|O43920|P13533|O95167|P12883|P06576
GO:0009124	nucleoside monophosphate biosynthetic process	4.61E-07	4.65E-06	8	129	190	35980	O75947|P25705|P48047|P18859|P36542|P56381|Q8WYQ3|P06576
GO:0009126	purine nucleoside monophosphate metabolic process	4.48E-24	1.48E-22	28	370	190	35980	P13929|O75947|P0DMV8|P0DMV9|O43674|O00483|P36542|P10176|O95178|O96000|P13073|P25705|P18859|P56381|Q8WYQ3|O15239|P20674|O00217|O95169|P51970|P48047|P04406|O14561|O43920|P13533|O95167|P12883|P06576
GO:0009127	purine nucleoside monophosphate biosynthetic process	6.41E-08	7.07E-07	8	100	190	35980	O75947|P25705|P48047|P18859|P36542|P56381|Q8WYQ3|P06576
GO:0009141	nucleoside triphosphate metabolic process	1.00E-24	3.68E-23	29	389	190	35980	P13929|O75947|P0DMV8|P0DMV9|O43674|O00483|P36542|P10176|O95178|O96000|P13073|P25705|P18859|P56381|Q8WYQ3|O15239|P20674|O00217|O95169|P51970|P48047|P04406|O14561|O43920|P13533|O95167|P12883|P17081|P06576

<table>
<tr><th></th><th></th><th></th><th></th><th></th><th></th><th></th><th></th><th></th></tr>
<tr><td>GO:0009142</td><td>nucleoside triphosphate biosynthetic process</td><td>1.55E-07</td><td>1.65E-06</td><td>8</td><td>112</td><td>190</td><td>35980</td><td>O75947|P25705|P48047|P18859|P36542|P56381|Q8WYQ3|P06576</td></tr>
<tr><td>GO:0009144</td><td>purine nucleoside triphosphate metabolic process</td><td>1.10E-25</td><td>4.48E-24</td><td>29</td><td>360</td><td>190</td><td>35980</td><td>P13929|O75947|P0DMV8|P0DMV9|O43674|O00483|P36542|P10176|O95178|O96000|P13073|P25705|P18859|P56381|Q8WYQ3|O15239|P20674|O00217|O95169|P51970|P48047|P04406|O14561|O43920|P13533|O95167|P12883|P17081|P06576</td></tr>
<tr><td>GO:0009145</td><td>purine nucleoside triphosphate biosynthetic process</td><td>3.94E-08</td><td>4.50E-07</td><td>8</td><td>94</td><td>190</td><td>35980</td><td>O75947|P25705|P48047|P18859|P36542|P56381|Q8WYQ3|P06576</td></tr>
<tr><td>GO:0009150</td><td>purine ribonucleotide metabolic process</td><td>1.75E-24</td><td>6.25E-23</td><td>32</td><td>526</td><td>190</td><td>35980</td><td>P13929|O75947|P0DMV8|P0DMV9|P01160|O43674|O00483|P36542|P10176|O95178|O96000|P16860|P13073|P25705|P18859|P56381|Q8WYQ3|O15239|P20674|O00217|Q08493|O95169|P51970|P48047|P04406|O14561|O43920|P13533|O95167|P12883|P17081|P06576</td></tr>
<tr><td>GO:0009152</td><td>purine ribonucleotide biosynthetic process</td><td>1.73E-07</td><td>1.82E-06</td><td>10</td><td>207</td><td>190</td><td>35980</td><td>O75947|P16860|P25705|P48047|P18859|P01160|P36542|P56381|Q8WYQ3|P06576</td></tr>
<tr><td>GO:0009156</td><td>ribonucleoside monophosphate biosynthetic process</td><td>2.18E-07</td><td>2.27E-06</td><td>8</td><td>117</td><td>190</td><td>35980</td><td>O75947|P25705|P48047|P18859|P36542|P56381|Q8WYQ3|P06576</td></tr>
<tr><td>GO:0009161</td><td>ribonucleoside monophosphate metabolic process</td><td>1.43E-23</td><td>4.52E-22</td><td>28</td><td>386</td><td>190</td><td>35980</td><td>P13929|O75947|P0DMV8|P0DMV9|O43674|O00483|P36542|P10176|O95178|O96000|P13073|P25705|P18859|P56381|Q8WYQ3|O15239|P20674|O00217|O95169|P51970|P48047|P04406|O14561|O43920|P13533|O95167|P12883|P06576</td></tr>
<tr><td>GO:0009163</td><td>nucleoside biosynthetic process</td><td>7.43E-06</td><td>6.20E-05</td><td>8</td><td>187</td><td>190</td><td>35980</td><td>O75947|P25705|P48047|P18859|P36542|P56381|Q8WYQ3|P06576</td></tr>
<tr><td>GO:0009165</td><td>nucleotide biosynthetic process</td><td>2.47E-06</td><td>2.28E-05</td><td>11</td><td>344</td><td>190</td><td>35980</td><td>O75947|P16860|P25705|P48047|P18859|P01160|Q9NPI5|P36542|P56381|Q8WYQ3|P06576</td></tr>
<tr><td>GO:0009167</td><td>purine ribonucleoside monophosphate metabolic process</td><td>4.16E-24</td><td>1.39E-22</td><td>28</td><td>369</td><td>190</td><td>35980</td><td>P13929|O75947|P0DMV8|P0DMV9|O43674|O00483|P36542|P10176|O95178|O96000|P13073|P25705|P18859|P56381|Q8WYQ3|O15239|P20674|O00217|O95169|P51970|P48047|P04406|O14561|O43920|P13533|O95167|P12883|P06576</td></tr>
<tr><td>GO:0009168</td><td>purine ribonucleoside monophosphate biosynthetic</td><td>6.41E-08</td><td>7.07E-07</td><td>8</td><td>100</td><td>190</td><td>35980</td><td>O75947|P25705|P48047|P18859|P36542|P56381|Q8WYQ3|P06576</td></tr>
<tr><td>GO:0009187</td><td>cyclic nucleotide metabolic process</td><td>1.29E-02</td><td>3.78E-02</td><td>3</td><td>92</td><td>190</td><td>35980</td><td>P16860|Q08493|P01160</td></tr>
<tr><td>GO:0009199</td><td>ribonucleoside triphosphate metabolic process</td><td>1.02E-25</td><td>4.20E-24</td><td>29</td><td>359</td><td>190</td><td>35980</td><td>P13929|O75947|P0DMV8|P0DMV9|O43674|O00483|P36542|P10176|O95178|O96000|P13073|P25705|P18859|P56381|Q8WYQ3|O15239|P20674|O00217|O95169|P51970|P48047|P04406|O14561|O43920|P13533|O95167|P12883|P17081|P06576</td></tr>
<tr><td>GO:0009201</td><td>ribonucleoside triphosphate biosynthetic process</td><td>5.92E-08</td><td>6.60E-07</td><td>8</td><td>99</td><td>190</td><td>35980</td><td>O75947|P25705|P48047|P18859|P36542|P56381|Q8WYQ3|P06576</td></tr>
<tr><td>GO:0009205</td><td>purine ribonucleoside triphosphate metabolic process</td><td>6.27E-26</td><td>2.63E-24</td><td>29</td><td>353</td><td>190</td><td>35980</td><td>P13929|O75947|P0DMV8|P0DMV9|O43674|O00483|P36542|P10176|O95178|O96000|P13073|P25705|P18859|P56381|Q8WYQ3|O15239|P20674|O00217|O95169|P51970|P48047|P04406|O14561|O43920|P13533|O95167|P12883|P17081|P06576</td></tr>
<tr><td>GO:0009206</td><td>purine ribonucleoside triphosphate biosynthetic process</td><td>3.62E-08</td><td>4.17E-07</td><td>8</td><td>93</td><td>190</td><td>35980</td><td>O75947|P25705|P48047|P18859|P36542|P56381|Q8WYQ3|P06576</td></tr>
<tr><td>GO:0009250</td><td>glucan biosynthetic process</td><td>1.85E-05</td><td>1.45E-04</td><td>4</td><td>30</td><td>190</td><td>35980</td><td>P0CG47|P62987|P62979|P0CG48</td></tr>
<tr><td>GO:0009259</td><td>ribonucleotide metabolic process</td><td>4.63E-24</td><td>1.51E-22</td><td>32</td><td>543</td><td>190</td><td>35980</td><td>P13929|O75947|P0DMV8|P0DMV9|P01160|O43674|O00483|P36542|P10176|O95178|O96000|P16860|P13073|P25705|P18859|P56381|Q8WYQ3|O15239|P20674|O00217|Q08493|O95169|P51970|P48047|P04406|O14561|O43920|P13533|O95167|P12883|P17081|P06576</td></tr>
<tr><td>GO:0009260</td><td>ribonucleotide biosynthetic process</td><td>3.30E-07</td><td>3.37E-06</td><td>10</td><td>222</td><td>190</td><td>35980</td><td>O75947|P16860|P25705|P48047|P18859|P01160|P36542|P56381|Q8WYQ3|P06576</td></tr>
<tr><td>GO:0009266</td><td>response to temperature stimulus</td><td>2.58E-03</td><td>1.05E-02</td><td>6</td><td>258</td><td>190</td><td>35980</td><td>P0DMV8|P0DMV9|Q9UBY9|P16989|P02511|P00441</td></tr>
<tr><td>GO:0009408</td><td>response to heat</td><td>2.63E-03</td><td>1.06E-02</td><td>5</td><td>178</td><td>190</td><td>35980</td><td>P0DMV8|P0DMV9|Q9UBY9|P02511|P00441</td></tr>
<tr><td>GO:0009605</td><td>response to external stimulus</td><td>3.25E-06</td><td>2.92E-05</td><td>31</td><td>2393</td><td>190</td><td>35980</td><td>P0CG47|P52179|P51684|P01160|P40925|P10176|P0CG48|P39019|P13693|P62891|P13073|P83731|Q16654|P62979|P62158|P24752|Q01628|P00441|P01034|Q14116|O15273|P13639|O75116|P68133|Q6P6C2|P17535|P19105|P60660|P62987|P04792|P25116</td></tr>
<tr><td>GO:0009607</td><td>response to biotic stimulus</td><td>2.11E-03</td><td>8.89E-03</td><td>13</td><td>973</td><td>190</td><td>35980</td><td>P52179|Q14116|P40925|Q6P6C2|P10176|P17535|P13693|P62891|P62987|Q16654|P04792|Q01628|P25116</td></tr>
<tr><td>GO:0009611</td><td>response to wounding</td><td>5.60E-08</td><td>6.28E-07</td><td>19</td><td>798</td><td>190</td><td>35980</td><td>P07602|Q13765|P01033|P13929|P16615|P35613|P19105|P05090|P84243|P60033|P09382|P68871|P09493|P04275|P62158|P04792|P25116|P00441|P01034</td></tr>
<tr><td>GO:0009628</td><td>response to abiotic stimulus</td><td>4.10E-06</td><td>3.64E-05</td><td>21</td><td>1269</td><td>190</td><td>35980</td><td>P0CG47|P02144|O15273|P0DMV8|P0DMV9|P01160|P19021|P68133|P16989|Q6P6C2|P17535|P0CG48|Q05639|Q9UBY9|P62987|P62979|P02511|Q15004|P62158|P00441|P01034</td></tr>
<tr><td>GO:0009636</td><td>response to toxic substance</td><td>1.76E-03</td><td>7.66E-03</td><td>7</td><td>324</td><td>190</td><td>35980</td><td>P68871|O14880|P47985|P22352|P69905|P00441|P01034</td></tr>
<tr><td>GO:0009653</td><td>anatomical structure morphogenesis</td><td>3.67E-08</td><td>4.21E-07</td><td>37</td><td>2626</td><td>190</td><td>35980</td><td>P0CG47|P10916|P35613|P0CG48|P63244|P05090|P19429|Q14192|P09493|P83731|P62979|P62158|P45379|P00441|Q13765|Q14116|O15273|Q14896|O75116|P19021|P19022|P68133|P68032|P13533|Q08431|P19105|P12883|O14958|P63173|P08493|P60660|P08590|P62987|P02511|P25116|P06576|P63316</td></tr>
<tr><td>GO:0009719</td><td>response to endogenous stimulus</td><td>2.39E-12</td><td>3.94E-11</td><td>37</td><td>1852</td><td>190</td><td>35980</td><td>P0CG47|Q969T9|P01160|P16615|P35613|P0CG48|P84243|P60033|Q14192|P17174|Q16654|P62979|P62158|P62753|P24752|P00441|P01034|P01033|P02144|P13639|O75116|O14880|P19021|P68133|P13533|Q08431|P17535|Q8N8D1|P41222|O14958|P68104|P08574|P62987|P47985|P02511|Q13772|P17081</td></tr>
<tr><td>GO:0009725</td><td>response to hormone</td><td>2.10E-13</td><td>3.76E-12</td><td>31</td><td>1187</td><td>190</td><td>35980</td><td>P0CG47|Q969T9|P01160|P16615|P35613|P0CG48|P84243|Q14192|P17174|Q16654|P62979|P62158|P62753|P24752|P01034|P01033|P02144|P13639|O75116|P19021|P68133|Q08431|P17535|Q8N8D1|P41222|P08574|P62987|P47985|P02511|Q13772|P17081</td></tr>
<tr><td>GO:0009887</td><td>organ morphogenesis</td><td>9.13E-07</td><td>8.96E-06</td><td>19</td><td>959</td><td>190</td><td>35980</td><td>O15273|Q14896|P10916|P19021|P19022|P68133|P68032|P13533|P35613|P12883|P19429|P63173|P08493|Q14192|P08590|P09493|P45379|P00441|P63316</td></tr>
<tr><td>GO:0009888</td><td>tissue development</td><td>4.91E-09</td><td>6.35E-08</td><td>30</td><td>1677</td><td>190</td><td>35980</td><td>P07602|P0CG47|P13929|P10916|P01160|P16615|P35613|P05090|P19429|Q14192|P09493|P45379|P24752|P00441|P01034|Q13765|P01033|O15273|Q14896|P13639|P19022|P68133|P68032|P13533|P12883|P63173|P08493|P60660|P08590|P63316</td></tr>
<tr><td>GO:0009891</td><td>positive regulation of biosynthetic process</td><td>3.09E-04</td><td>1.74E-03</td><td>23</td><td>1969</td><td>190</td><td>35980</td><td>P0CG47|Q13765|P0DMV8|P13639|P0DMV9|Q969T9|P16989|Q8TAK5|Q9Y6H1|P17535|P0CG48|P63244|P60033|Q14192|P68871|P62987|P62979|Q13772|P62158|P17081|P04792|P25116|P01034</td></tr>
<tr><td>GO:0009892</td><td>negative regulation of metabolic process</td><td>3.28E-05</td><td>2.45E-04</td><td>34</td><td>3090</td><td>190</td><td>35980</td><td>P0CG47|P0DMV8|P0DMV9|Q8N3K9|P0CG48|P39019|P63244|P05090|P19429|P84243|Q14192|P62979|P62158|P45379|Q8WYQ3|Q01628|P00441|P01034|P51452|Q13765|P54368|P01033|P04406|O75116|P16989|P17535|P62263|P62987|Q14240|P02511|P04792|P60228|P20585|Q8N335</td></tr>
<tr><td>GO:0009893</td><td>positive regulation of metabolic process</td><td>1.12E-04</td><td>7.11E-04</td><td>41</td><td>4288</td><td>190</td><td>35980</td><td>P07602|P0CG47|P0DMV8|P0DMV9|Q969T9|Q8TAK5|P0CG48|P39019|P63244|P84243|P60033|Q14192|P68871|P09493|P62979|P62158|P45379|P00441|P01034|Q13765|P54368|Q14116|Q14896|P13639|O75116|P19022|P68133|P16989|Q9Y6H1|P68032|P17535|P15880|Q05639|Q9NWT8|P08590|P62987|Q13772|P17081|P04792|P25116|P20585</td></tr>
<tr><td>GO:0009894</td><td>regulation of catabolic process</td><td>1.70E-03</td><td>7.46E-03</td><td>11</td><td>723</td><td>190</td><td>35980</td><td>P0CG47|P54368|P01033|P63244|Q05639|P68104|P60033|P62987|P62979|P0CG48|P01034</td></tr>
<tr><td>GO:0009896</td><td>positive regulation of catabolic process</td><td>5.01E-03</td><td>1.85E-02</td><td>7</td><td>392</td><td>190</td><td>35980</td><td>P0CG47|P54368|P63244|P60033|P62987|P62979|P0CG48</td></tr>
<tr><td>GO:0009966</td><td>regulation of signal transduction</td><td>6.34E-03</td><td>2.16E-02</td><td>27</td><td>3080</td><td>190</td><td>35980</td><td>P07602|P0CG47|P0DMV8|P0DMV9|Q8N3K9|P0CG48|P13693|P63244|P05090|P60033|P23327|P62979|P62158|P00441|P51452|P01033|Q14116|P19022|P16989|Q8N8D1|O14958|P09382|P62987|P17081|P04792|P25116|Q8N335</td></tr>
<tr><td>GO:0009967</td><td>positive regulation of signal transduction</td><td>8.56E-03</td><td>2.76E-02</td><td>16</td><td>1554</td><td>190</td><td>35980</td><td>P07602|P0CG47|Q14116|P0DMV8|P0DMV9|P19022|P0CG48|Q8N8D1|P63244|P60033|P09382|P62987|P62979|P62158|P25116|P00441</td></tr>
<tr><td>GO:0009968</td><td>negative regulation of signal transduction</td><td>2.12E-04</td><td>1.25E-03</td><td>17</td><td>1192</td><td>190</td><td>35980</td><td>P0CG47|P51452|P0DMV8|P0DMV9|P19022|P16989|Q8N3K9|P0CG48|P13693|P63244|P05090|O14958|P62987|P62979|P62158|P04792|Q8N335</td></tr>
<tr><td>GO:0009987</td><td>cellular process</td><td>5.78E-15</td><td>1.13E-13</td><td>179</td><td>25915</td><td>190</td><td>35980</td><td>O75947|P10916|Q969T9|O43674|Q9H3K2|P16615|O96000|P16860|P13073|O14949|P09493|P07195|P46783|P83731|P62913|P62753|P24752|O15239|P20674|Q13765|O95169|P13639|P48047|Q9Y277|P04406|O14561|P80297|Q9Y5N6|P16989|Q9Y6H1|O95167|Q08431|P19105|P84098|O14958|P63173|P68104|P09382|P08574|P47985|P46777|Q13772|P60866|P46776|P60228|P62249|Q13011|Q8N335|P07602|P52179|P25398|P68363|P10176|O95178|P12235|P13693|P62891|P19429|P25705|P18859|Q14192|P68871|Q16654|P56381|Q01628|P00441|P54368|O00217|Q14116|O15273|Q08493|P08708|Q14515|O75116|P68133|P18077|P17661|Q6P6C2|P17535|P12883|Q8N8D1|P42677|P41222|P15880|P17540|Q02543|Q9NWT8|Q9Y3D2|P62263|Q14240|P62945|P62269|P0CG47|P06732|Q9NSD9|P0DMV8|P0DMV9|P01160|P40926|P40925|P62280|Q8TAK5|P36542|P35613|P0CG48|P39019|P63244|P05090|P00505|P39023|P62273|P24310|P69905|P62158|P45379|P24311|Q8WYQ3|P01034|Q07020|P01033|P10606|Q14896|Q96H40|O14880|P19021|P19022|P14406|P37108|O43920|P68032|Q05639|P08493|P61353|P60660|P02511|P05386|P17081|P05387|P13929|P02792|P09669|P51684|O00483|P14854|P42766|Q02878|P84243|P60033|Q9NPI5|P17174|P61247|P62857|P23327|P22352|Q16698|P62979|Q15004|P62851|P51452|P02144|Q14315|P51970|Q9P0U1|O75954|P62906|P15954|P51857|P13533|Q00325|P08590|P62987|P04275|P63313|P04792|P25116|P20585|P06576|P63316|P48735</td></tr>
<tr><td>GO:0009991</td><td>response to extracellular stimulus</td><td>3.73E-03</td><td>1.43E-02</td><td>8</td><td>471</td><td>190</td><td>35980</td><td>P13073|P13639|P68133|Q16654|P24752|P00441|P39019|P01034</td></tr>
</table>

GO:0010033	response to organic substance	1.37E-16	2.87E-15	57	3237	190	35980	P0CG47|Q12988|Q969T9|P01160|P16615|P35613|P0CG48|P63244|P00505|P39023|P62158|P62753|P24752|P01034|P01033|P13639|P04406|O14880|P80297|P19021|P16989|P68032|Q08431|O14958|P68104|P09382|P08574|P47985|P02511|Q13772|P17081|P07602|P51684|P68363|P84243|P60033|Q14192|Q9UBY9|P17174|Q16654|P22352|P62979|Q01628|P00441|P51452|P02144|Q14116|O75116|P68133|P13533|P17535|Q8N8D1|P41222|P62987|Q14240|P04792|P25116
GO:0010035	response to inorganic substance	2.74E-09	3.68E-08	17	517	190	35980	P02144|P13639|P80297|P19021|P68133|P35613|P17535|O14958|Q05639|P68871|P02511|P69905|P62158|P45379|P00441|P63316|P01034
GO:0010038	response to metal ion	2.03E-05	1.58E-04	10	353	190	35980	O14958|P80297|P19021|P68133|P62158|P45379|P35613|P17535|P00441|P63316
GO:0010243	response to organonitrogen compound	1.74E-06	1.63E-05	19	1002	190	35980	P0CG47|P01033|P13639|P01160|O14880|P16615|P35613|P17535|P0CG48|O14958|P17174|P08574|P62987|Q16654|P62979|P62158|P17081|P62753|P00441
GO:0010257	NADH dehydrogenase complex assembly	1.13E-12	1.88E-11	10	62	190	35980	O00217|O95169|P51970|O43674|O14561|O43920|O95167|O95178|O96000|O15239
GO:0010460	positive regulation of heart rate	8.74E-06	7.22E-05	4	25	190	35980	P01160|P09493|P16615|P23327
GO:0010467	gene expression	5.56E-15	1.09E-13	73	5483	190	35980	P0CG47|Q9NSD9|P0DMV8|P01160|P62280|Q8TAK5|P0CG48|P39019|P63244|P13073|P39023|P62273|P46783|P83731|P62913|P24311|P62753|P20674|Q13765|Q07020|P10606|Q96H40|P13639|P37108|P16989|Q9Y6H1|P84098|Q05639|P63173|P68104|P61353|P46777|P05386|Q13772|P60866|P46776|P05387|P60228|P62249|P25398|P09669|O00483|P14854|P10176|P12235|P62891|P42766|Q02878|P84243|Q14192|P61247|P62857|P62979|P62851|P08708|P62906|P18077|P15954|Q6P6C2|P17535|Q8N8D1|P42677|P15880|Q02543|Q9NWT8|Q00325|P62263|P62987|Q14240|P62945|P04792|P62269|P25116
GO:0010522	regulation of calcium ion transport into cytosol	1.65E-03	7.26E-03	4	95	190	35980	O14958|P23327|P62158|P25116
GO:0010557	positive regulation of macromolecule biosynthetic process	1.56E-03	6.94E-03	20	1815	190	35980	P0CG47|Q13765|P0DMV8|P13639|P0DMV9|Q969T9|P16989|Q8TAK5|Q9Y6H1|P17535|P0CG48|P60033|Q14192|P62987|P62979|Q13772|P17081|P04792|P25116|P01034
GO:0010562	positive regulation of phosphorus metabolic process	5.72E-03	1.98E-02	14	1224	190	35980	P07602|P0CG47|Q14116|O75116|P19022|P0CG48|P63244|Q05639|P60033|P62987|P62979|P62158|P25116|P00441
GO:0010604	positive regulation of macromolecule metabolic process	2.15E-04	1.26E-03	32	3122	190	35980	P07602|P0CG47|P0DMV8|P0DMV9|Q969T9|Q8TAK5|P0CG48|P63244|P84243|P60033|Q14192|P62979|P62158|P00441|P01034|Q13765|P54368|Q14116|P13639|O75116|P19022|P68133|P16989|Q9Y6H1|P68032|P17535|Q9NWT8|P62987|Q13772|P17081|P04792|P25116
GO:0010605	negative regulation of macromolecule metabolic process	5.64E-04	2.90E-03	28	2733	190	35980	P0CG47|P0DMV8|P0DMV9|Q8N3K9|P0CG48|P63244|P05090|P84243|Q14192|P62979|P62158|Q01628|P01034|P51452|Q13765|P01033|P04406|O75116|P16989|P17535|P62263|P62987|Q14240|P02511|P04792|P60228|P20585|Q8N335
GO:0010608	posttranscriptional regulation of gene expression	2.08E-06	1.93E-05	14	564	190	35980	P0CG47|P0DMV8|P13639|P04406|P16989|P0CG48|P63244|P63173|P62263|P62987|Q14240|P62979|P04792|P60228
GO:0010628	positive regulation of gene expression	1.83E-04	1.10E-03	23	1898	190	35980	P0CG47|Q13765|Q14116|P0DMV8|P13639|P0DMV9|Q969T9|O75116|P68133|P16989|Q8TAK5|Q9Y6H1|P68032|P17535|P0CG48|P84243|P60033|Q14192|P62987|P62979|Q13772|P17081|P25116
GO:0010634	positive regulation of epithelial cell migration	1.79E-02	4.86E-02	3	104	190	35980	P51684|O75116|P04792
GO:0010646	regulation of cell communication	5.42E-03	1.90E-02	30	3496	190	35980	P07602|P0CG47|P0DMV8|P0DMV9|P16615|Q8N3K9|P12235|P0CG48|P13693|P63244|P05090|P60033|P23327|P62979|P62158|P00441|P51452|P01033|Q14116|Q08493|P19022|P16989|Q8N8D1|O14958|P09382|P62987|P17081|P04792|P25116|Q8N335
GO:0010648	negative regulation of cell communication	2.32E-04	1.34E-03	18	1317	190	35980	P0CG47|P51452|Q08493|P0DMV8|P0DMV9|P19022|P16989|Q8N3K9|P0CG48|P13693|P63244|P05090|O14958|P62987|P62979|P62158|P04792|Q8N335
GO:0010649	regulation of cell communication by electrical coupling	1.69E-05	1.33E-04	3	10	190	35980	O14958|P23327|P62158
GO:0010803	regulation of tumor necrosis factor-mediated signaling	8.45E-09	1.07E-07	7	50	190	35980	P0CG47|P63244|P0DMV8|P0DMV9|P62987|P62979|P0CG48
GO:0010880	regulation of release of sequestered calcium ion into cytosol by sarcoplasmic reticulum	3.45E-04	1.91E-03	3	26	190	35980	O14958|P23327|P62158
GO:0010881	regulation of cardiac muscle contraction by regulation of the release of sequestered calcium ion	1.55E-04	9.48E-04	3	20	190	35980	O14958|P23327|P62158
GO:0010882	regulation of cardiac muscle contraction by calcium ion	7.37E-06	6.19E-05	4	24	190	35980	O14958|P16615|P23327|P62158
GO:0010927	cellular component assembly involved in morphogenesis	4.27E-05	3.07E-04	9	309	190	35980	O15273|Q14896|P10916|O14958|P09493|P68133|P68032|P45379|P13533
GO:0010939	regulation of necrotic cell death	7.72E-08	8.36E-07	6	41	190	35980	P0CG47|P62987|P16989|P62979|P12235|P0CG48
GO:0010941	regulation of cell death	8.76E-08	9.40E-07	29	1800	190	35980	P07602|P0CG47|P0DMV8|P0DMV9|P12235|P0CG48|P13693|P63244|Q14192|P68871|P61247|Q16654|P62979|P69905|P62753|P00441|P01034|Q13765|P01033|P16989|P68032|P17535|Q8N8D1|Q05639|P09382|P62987|P02511|P04792|P25116
GO:0010942	positive regulation of cell death	3.84E-04	2.11E-03	12	702	190	35980	P0CG47|P63244|Q05639|P68871|P62987|P62979|P69905|P62753|P25116|P00441|P0CG48|Q8N8D1
GO:0010959	regulation of metal ion transport	1.18E-02	3.48E-02	6	355	190	35980	O14958|P01160|P23327|P62158|P25116|Q8N335
GO:0012501	programmed cell death	4.97E-03	1.83E-02	14	1204	190	35980	P0CG47|P04406|Q9H3K2|P68032|P12235|P0CG48|Q8N8D1|P63244|P09382|P62987|P62979|P02511|P25116|P01034
GO:0014070	response to organic cyclic compound	2.18E-07	2.27E-06	21	1055	190	35980	Q14116|P13639|Q969T9|O75116|P19021|P68133|P35613|Q08431|P17535|Q8N8D1|P41222|O14958|Q14192|P09382|P17174|P02511|Q13772|P62158|P24752|P00441|P01034
GO:0014074	response to purine-containing compound	1.61E-02	4.41E-02	4	182	190	35980	O14958|P35613|P17535|P00441
GO:0014706	striated muscle tissue development	5.02E-12	8.07E-11	16	293	190	35980	O15273|Q14896|P10916|P13639|P01160|P68133|P68032|P13533|P12883|P19429|Q14192|P60660|P08590|P09493|P45379|P63316
GO:0014733	regulation of skeletal muscle adaptation	1.30E-06	1.27E-05	4	16	190	35980	P16615|Q8N3K9|P12883|P63316
GO:0014866	skeletal myofibril assembly	4.99E-06	4.39E-05	3	7	190	35980	O15273|P68133|P68032
GO:0014883	transition between fast and slow fiber	1.19E-05	9.64E-05	3	9	190	35980	P16615|P12883|P63316
GO:0014888	striated muscle adaptation	3.45E-04	1.91E-03	3	26	190	35980	O15273|P01160|P68133
GO:0015031	protein transport	7.63E-16	1.53E-14	42	1852	190	35980	P25398|P62280|P35613|P39019|P62891|P42766|Q02878|P39023|P62273|P46783|P61247|P83731|P62857|P62913|P62979|P62851|P62753|Q13765|Q07020|Q9P0U1|P08708|P62906|P18077|P37108|Q9Y6H1|P42677|P84098|P15880|P63173|Q02543|P61353|P62263|P62987|P62945|P46777|P05386|P60866|P46776|P62269|P05387|P25116|P62249
GO:0015669	gas transport	1.24E-03	5.79E-03	3	40	190	35980	P02144|P68871|P69905
GO:0015671	oxygen transport	9.10E-04	4.44E-03	3	36	190	35980	P02144|P68871|P69905
GO:0015672	monovalent inorganic cation transport	1.90E-08	2.29E-07	20	827	190	35980	O75947|P10606|P09669|P48047|P14406|O00483|P14854|P36542|P15954|P10176|P13073|P25705|P18859|O14949|P47985|P56381|P24310|P24311|P06576|P20674
GO:0015980	energy derivation by oxidation of organic compounds	3.54E-36	2.92E-34	38	402	190	35980	P0CG47|O75947|P09669|O43674|P40926|P40925|O00483|P14854|P36542|P10176|O95178|P12235|P0CG48|O96000|P13073|P25705|P18859|O14949|P62979|P56381|P62158|P24311|O15239|P20674|O00217|P10606|O95169|P51970|P48047|O14561|O43920|P15954|O95167|P08574|P62987|P47985|P06576|P48735
GO:0015985	energy coupled proton transport, down electrochemical	3.33E-09	4.40E-08	7	44	190	35980	O75947|P25705|P48047|P18859|P36542|P56381|P06576
GO:0015986	ATP synthesis coupled proton transport	3.33E-09	4.40E-08	7	44	190	35980	O75947|P25705|P48047|P18859|P36542|P56381|P06576
GO:0015992	proton transport	1.65E-16	3.44E-15	20	296	190	35980	O75947|P10606|P09669|P48047|P14406|O00483|P14854|P36542|P15954|P10176|P13073|P25705|P18859|O14949|P47985|P56381|P24310|P24311|P06576|P20674
GO:0016032	viral process	1.56E-34	1.05E-32	46	783	190	35980	P0CG47|P25398|P0DMV8|P0DMV9|P62280|P12235|P0CG48|P39019|P62891|P42766|P63244|Q02878|P60033|P39023|P62273|P46783|P61247|P83731|P62857|P62913|P62979|P62851|P62753|Q13765|Q07020|P08708|P62906|P18077|Q08431|P42677|P84098|P15880|P63173|Q02543|P61353|P62263|P62987|Q14240|P62945|P46777|P05386|P60866|P46776|P62269|P05387|P62249
GO:0016043	cellular component organization	3.35E-24	1.14E-22	101	7195	190	35980	P10916|O43674|P16615|O96000|P09493|P46783|P83731|P62913|P62753|P24752|O15239|O95169|P04406|O14561|Q9Y6H1|O95167|Q08431|P19105|P84098|O14958|P63173|P46777|P60866|P46776|P60228|P62249|P52179|P25398|P68363|P10176|O95178|P12235|P62891|P68871|P00441|O00217|O15273|P08708|O75116|P68133|P18077|P17661|P42677|P15880|Q02543|Q9NWT8|Q9Y3D2|P62263|P62945|P62269|P0CG47|P0DMV8|P40925|P62280|P35613|P0CG48|P39019|P05090|P39023|P62273|P69905|P62158|P45379|Q8WYQ3|P01034|Q07020|P01033|Q14896|P19021|P19022|P37108|O43920|P68032|P61353|P60660|P02511|P05386|P17081|P05387|P02792|P42766|Q02878|P84243|P61247|P62857|P22352|Q16698|P62979|Q15004|P62851|Q14315|P51970|Q9P0U1|P62906|P13533|P62987|P04275|P63313|P25116|P20585|P06576
GO:0016051	carbohydrate biosynthetic process	4.20E-08	4.78E-07	10	178	190	35980	P0CG47|P13929|P00505|P40926|P04406|P40925|P17174|P62987|P62979|P0CG48
GO:0016054	organic acid catabolic process	2.39E-03	9.85E-03	6	254	190	35980	P00505|P17174|Q16698|P51857|P24752|Q13011

GO:0016070	RNA metabolic process	7.22E-13	1.23E-11	62	4569	190	35980	P0CG47|Q9NSD9|P01160|P62280|Q8TAK5|P0CG48|P39019|P13073|P39023|P62273|P46783|P83731|P62913|P24311|P62753|P20674|Q13765|Q07020|P10606|Q96H40|P16989|Q9Y6H1|P84098|P63173|P68104|P61353|P46777|P05386|Q13772|P60866|P46776|P05387|P60228|P62249|P25398|P09669|O00483|P14854|P10176|P62891|P42766|Q02878|Q14192|P61247|P62857|P62979|P62851|P08708|P62906|P18077|P15954|Q6P6C2|P17535|Q8N8D1|P42677|P15880|Q02543|P62263|P62987|Q14240|P62945|P62269
GO:0016071	mRNA metabolic process	1.60E-28	7.53E-27	40	743	190	35980	P25398|P62280|P39019|P62891|P42766|Q02878|P39023|P62273|P46783|P61247|P83731|P62857|P62913|P62979|P62851|P62753|Q07020|P08708|P62906|P18077|Q6P6C2|Q8N8D1|P42677|P84098|P15880|P63173|Q02543|P61353|P62263|P62987|Q14240|P62945|P46777|P05386|P60866|P46776|P62269|P05387|P60228|P62249
GO:0016072	rRNA metabolic process	7.76E-08	8.36E-07	10	190	190	35980	P42766|P08708|P62263|P18077|P62857|P62913|P46777|P62753|P62249|P39019
GO:0016236	macroautophagy	1.82E-06	1.70E-05	10	268	190	35980	P0CG47|P52179|Q9P0U1|P40925|P62987|Q16654|P62979|Q6P6C2|P10176|P0CG48
GO:0016259	selenocysteine metabolic process	4.22E-59	6.27E-56	36	90	190	35980	P25398|P62280|P39019|P62891|P42766|Q02878|P39023|P62273|P46783|P61247|P83731|P62857|P62913|P62979|P62851|P62753|Q07020|P08708|P62906|P18077|P42677|P84098|P15880|P63173|Q02543|P61353|P62263|P62987|P62945|P46777|P05386|P60866|P46776|P62269|P05387|P62249
GO:0016265	death	7.25E-03	2.41E-02	14	1259	190	35980	P0CG47|P04406|Q9H3K2|P68032|P12235|P0CG48|Q8N8D1|P63244|P09382|P62987|P62979|P02511|P25116|P01034
GO:0016310	phosphorylation	6.95E-06	5.88E-05	32	2611	190	35980	P0CG47|P13929|P06732|O43674|O00483|P36542|P10176|O95178|P0CG48|O96000|P13073|Q9NPI5|Q16654|P62979|P62158|Q8WYQ3|O15239|P20674|P51452|O00217|Q14116|O95169|P51970|P04406|O14561|O75116|O43920|O95167|P17540|P62987|P02511|P25116
GO:0016482	cytoplasmic transport	3.21E-25	1.26E-23	42	1030	190	35980	P25398|P16615|P62280|P39019|P62891|P42766|Q02878|P39023|P62273|P46783|P61247|P83731|P62857|P62913|P62979|P62851|P62753|Q07020|Q9P0U1|P08708|P62906|P18077|P37108|Q9Y6H1|Q6P6C2|P42677|P84098|P15880|P63173|Q02543|P61353|P62263|P62987|P62945|P46777|P05386|P60866|P46776|P62269|P05387|P25116|P62249
GO:0016485	protein processing	5.21E-03	1.85E-02	6	298	190	35980	P0CG47|P63244|P62987|P62979|P25116|P0CG48
GO:0017015	regulation of transforming growth factor beta receptor	3.30E-03	1.29E-02	4	115	190	35980	P0CG47|P62987|P62979|P0CG48
GO:0018130	heterocycle biosynthetic process	2.81E-19	6.63E-18	68	3999	190	35980	P0CG47|O75947|P01160|P62280|Q8TAK5|P36542|P0CG48|P39019|P16860|P13073|P39023|P62273|P46783|P83731|P62913|P24311|P62753|Q8WYQ3|P20674|Q13765|Q07020|P10606|Q96H40|P48047|P16989|Q9Y6H1|P84098|P63173|P68104|P61353|P46777|P05386|Q13772|P60866|P46776|P05387|P62249|P25398|P09669|O00483|P14854|P10176|P62891|P42766|Q02878|P25705|P18859|Q14192|Q9NPI5|P61247|P62857|P62979|P56381|Q15004|P62851|P08708|P62906|P18077|P15954|P17535|P42677|P15880|Q02543|P62263|P62987|P62945|P62269|P06576
GO:0019058	viral life cycle	1.56E-46	2.31E-44	41	288	190	35980	P0CG47|P25398|P0DMV8|P0DMV9|P62280|P0CG48|P39019|P62891|P42766|Q02878|P60033|P39023|P62273|P46783|P61247|P83731|P62857|P62913|P62979|P62851|P62753|Q07020|P08708|P62906|P18077|P42677|P84098|P15880|P63173|Q02543|P61353|P62263|P62987|P62945|P46777|P05386|P60866|P46776|P62269|P05387|P62249
GO:0019068	virion assembly	4.83E-05	3.41E-04	4	38	190	35980	P0CG47|P62987|P62979|P0CG48
GO:0019080	viral gene expression	4.65E-57	4.60E-54	38	124	190	35980	P0CG47|P25398|P62280|P0CG48|P39019|P62891|P42766|Q02878|P39023|P62273|P46783|P61247|P83731|P62857|P62913|P62979|P62851|P62753|Q07020|P08708|P62906|P18077|P42677|P84098|P15880|P63173|Q02543|P61353|P62263|P62987|P62945|P46777|P05386|P60866|P46776|P62269|P05387|P62249
GO:0019082	viral protein processing	3.61E-07	3.67E-06	4	12	190	35980	P0CG47|P62987|P62979|P0CG48
GO:0019083	viral transcription	9.15E-55	3.51E-52	36	113	190	35980	P25398|P62280|P39019|P62891|P42766|Q02878|P39023|P62273|P46783|P61247|P83731|P62857|P62913|P62979|P62851|P62753|Q07020|P08708|P62906|P18077|P42677|P84098|P15880|P63173|Q02543|P61353|P62263|P62987|P62945|P46777|P05386|P60866|P46776|P62269|P05387|P62249
GO:0019216	regulation of lipid metabolic process	1.50E-03	6.70E-03	7	315	190	35980	P07602|P63244|P05090|Q05639|P60033|Q16654|P00441
GO:0019220	regulation of phosphate metabolic process	5.96E-03	2.04E-02	19	1904	190	35980	P07602|P0CG47|P51452|Q14116|O75116|P19022|Q8N3K9|P0CG48|P63244|Q05639|P60033|P62987|P23327|P62979|P62158|P04792|P25116|Q8N335|P00441
GO:0019318	hexose metabolic process	1.11E-09	1.53E-08	13	253	190	35980	P0CG47|P13929|P40926|P04406|P40925|P0CG48|P05090|P00505|P17174|P62987|Q16654|P62979|P62158
GO:0019319	hexose biosynthetic process	3.48E-06	3.11E-05	6	77	190	35980	P13929|P00505|P40926|P04406|P40925|P17174
GO:0019362	pyridine nucleotide metabolic process	2.23E-04	1.30E-03	7	228	190	35980	P13929|P40926|P04406|Q9NPI5|P07195|P40925|Q8N335
GO:0019438	aromatic compound biosynthetic process	1.93E-19	4.62E-18	68	3971	190	35980	P0CG47|O75947|P01160|P62280|Q8TAK5|P36542|P0CG48|P39019|P16860|P13073|P39023|P62273|P46783|P83731|P62913|P24311|P62753|Q8WYQ3|P20674|Q13765|Q07020|P10606|Q96H40|P48047|P16989|Q9Y6H1|P84098|P63173|P68104|P61353|P46777|P05386|Q13772|P60866|P46776|P05387|P62249|P25398|P09669|O00483|P14854|P10176|P62891|P42766|Q02878|P25705|P18859|Q14192|Q9NPI5|P61247|P62857|P62979|P56381|Q15004|P62851|P08708|P62906|P18077|P15954|P17535|P42677|P15880|Q02543|P62263|P62987|P62945|P62269|P06576
GO:0019439	aromatic compound catabolic process	2.91E-34	1.88E-32	39	489	190	35980	P25398|P62280|P39019|P62891|P42766|Q02878|P39023|P62273|P46783|P61247|P83731|P62857|P62913|P62979|P62851|P62753|Q07020|Q08493|P08708|P62906|P18077|P42677|P84098|P15880|P63173|Q02543|P61353|P62263|P62987|Q14240|P62945|P46777|P05386|P60866|P46776|P62269|P05387|P60228|P62249
GO:0019538	protein metabolic process	6.70E-06	5.72E-05	70	8155	190	35980	P0CG47|Q9NSD9|P01160|P40926|P62280|P0CG48|P39019|P63244|P39023|P62273|P46783|P83731|P62913|P62158|P62753|P01034|Q13765|Q07020|P13639|P04406|O14561|P19021|P37108|Q9Y6H1|Q08431|P84098|Q05639|P63173|P68104|P61353|P46777|P02511|P05386|P60866|P46776|P05387|P60228|P62249|P25398|P68363|P12235|P62891|P42766|Q02878|P84243|P61247|P62857|Q16654|P62979|P62851|P51452|Q14116|Q9P0U1|P08708|O75116|P62906|P18077|P42677|P15880|Q02543|Q9NWT8|Q9Y3D2|Q00325|P62263|P62987|Q14240|P62945|P04275|P62269|P25116
GO:0019637	organophosphate metabolic process	1.77E-17	3.82E-16	39	1418	190	35980	P13929|O75947|P06732|P0DMV8|P0DMV9|P01160|O43674|P40926|P40925|O00483|P36542|P10176|O95178|O96000|P16860|P13073|P25705|P18859|Q9NPI5|P07195|P56381|P62158|Q8WYQ3|O15239|P20674|O00217|Q08493|O95169|P51970|P48047|P04406|O14561|O43920|P13533|O95167|P12883|P17081|Q8N335|P06576
GO:0019674	NAD metabolic process	3.03E-07	3.12E-06	7	83	190	35980	P13929|P40926|P04406|Q9NPI5|P07195|P40925|Q8N335
GO:0019693	ribose phosphate metabolic process	1.83E-23	5.72E-22	32	568	190	35980	P13929|O75947|P0DMV8|P0DMV9|P01160|O43674|O00483|P36542|P10176|O95178|O96000|P16860|P13073|P25705|P18859|P56381|Q8WYQ3|O15239|P20674|O00217|Q08493|O95169|P51970|P48047|P04406|O14561|O43920|P13533|O95167|P12883|P17081|P06576
GO:0019722	calcium-mediated signaling	1.99E-03	8.47E-03	4	100	190	35980	O14958|P16615|P23327|P62158
GO:0019725	cellular homeostasis	1.47E-04	9.03E-04	14	831	190	35980	P0CG47|P02792|P51684|P16615|P0CG48|P13693|P63244|O14958|P19429|P09382|P23327|P25116|P00441|P06576
GO:0019752	carboxylic acid metabolic process	2.19E-32	1.30E-30	56	1471	190	35980	P06732|Q9NSD9|P40926|P40925|P62280|P35613|P39019|P00505|P39023|P62273|P07195|P46783|P83731|P62913|P62753|P24752|Q07020|P04406|O14561|P19021|P84098|P63173|P61353|P46777|P05386|P60866|P46776|P05387|P62249|Q13011|P13929|P25398|P62891|P42766|Q02878|P17174|P61247|P62857|Q16654|Q16698|P62979|P62851|P08708|P62906|P18077|P51857|P42677|P41222|P15880|P17540|Q02543|P62263|P62987|P62945|P62269|P48735
GO:0019932	second-messenger-mediated signaling	1.41E-02	4.06E-02	4	175	190	35980	O14958|P16615|P23327|P62158
GO:0019985	translesion synthesis	7.51E-06	6.26E-05	5	51	190	35980	P0CG47|P62987|P62979|Q15004|P0CG48
GO:0021762	substantia nigra development	1.86E-03	8.00E-03	3	46	190	35980	P18859|P14854|P62158
GO:0022411	cellular component disassembly	3.25E-36	2.76E-34	45	673	190	35980	P0CG47|P52179|P25398|P40925|P62280|P10176|P35613|P0CG48|P39019|P62891|P42766|Q02878|P39023|P62273|P46783|P61247|P83731|P62857|P62913|P62979|P62851|P62753|Q07020|P01033|Q9P0U1|P08708|P62906|P18077|P42677|P84098|P15880|P63173|Q02543|Q9NWT8|P61353|P62263|P62987|P62945|P46777|P05386|P60866|P46776|P62269|P05387|P62249
GO:0022607	cellular component assembly	1.54E-14	2.96E-13	47	2516	190	35980	P0CG47|P10916|O43674|O95178|P0CG48|P39019|O96000|Q02878|P84243|P39023|P68871|P09493|P46783|P83731|P62913|P22352|Q16698|P62979|P69905|P45379|P24752|O15239|O00217|Q14315|O15273|Q14896|O95169|P51970|P08708|O14561|P19021|P19022|P68133|O43920|P68032|P13533|O95167|P42677|O14958|P63173|Q9Y3D2|P62263|P62987|P46777|P02511|P04275|P60228
GO:0022613	ribonucleoprotein complex biogenesis	8.20E-10	1.13E-08	17	477	190	35980	P08708|P18077|P39019|P42677|P42766|Q02878|P63173|P39023|P62263|P46783|P83731|P62857|P62913|P46777|P62753|P60228|P62249
GO:0022618	ribonucleoprotein complex assembly	1.06E-08	1.33E-07	12	250	190	35980	P42677|Q02878|P63173|P08708|P39023|P62263|P46783|P83731|P62913|P46777|P60228|P39019

GO:0022898	regulation of transmembrane transporter activity	5.08E-03	1.85E-02	5	208	190	35980	O14958|P01160|P23327|P62158|Q8N335
GO:0022900	electron transport chain	3.89E-35	2.82E-33	29	173	190	35980	O75947|P09669|O43674|O00483|P14854|P36542|P10176|O95178|O96000|P13073|P25705|P18859|O14949|P56381|P24311|O15239|P20674|O00217|P10606|O95169|P51970|P48047|O14561|O43920|P15954|O95167|P08574|P47985|P06576
GO:0022904	respiratory electron transport chain	1.90E-36	1.67E-34	29	157	190	35980	O75947|P09669|O43674|O00483|P14854|P36542|P10176|O95178|O96000|P13073|P25705|P18859|O14949|P56381|P24311|O15239|P20674|O00217|P10606|O95169|P51970|P48047|O14561|O43920|P15954|O95167|P08574|P47985|P06576
GO:0023014	signal transduction by protein phosphorylation	3.68E-03	1.41E-02	8	470	190	35980	P0CG47|P51452|Q14116|P62987|P62979|P02511|P62158|P0CG48
GO:0023051	regulation of signaling	1.51E-03	6.76E-03	32	3500	190	35980	P07602|P0CG47|P0DMV8|P0DMV9|P01160|P16615|Q8N3K9|P12235|P0CG48|P13693|P63244|P05090|P19429|P60033|P23327|P62979|P62158|P00441|P51452|P01033|Q14116|Q08493|P19022|P16989|Q8N8D1|O14958|P09382|P62987|P17081|P04792|P25116|Q8N335
GO:0023056	positive regulation of signaling	1.83E-02	4.94E-02	16	1696	190	35980	P07602|P0CG47|Q14116|P0DMV8|P0DMV9|P19022|P0CG48|Q8N8D1|P63244|P60033|P09382|P62987|P62979|P62158|P25116|P00441
GO:0023057	negative regulation of signaling	2.08E-04	1.23E-03	18	1305	190	35980	P0CG47|P51452|Q08493|P0DMV8|P0DMV9|P19022|P16989|Q8N3K9|P0CG48|P13693|P63244|P05090|O14958|P62987|P62979|P62158|P04792|Q8N335
GO:0030003	cellular cation homeostasis	4.72E-04	2.49E-03	10	519	190	35980	P13693|P02792|P51684|O14958|P19429|P16615|P23327|P25116|P00441|P06576
GO:0030029	actin filament-based process	1.75E-08	2.13E-07	20	823	190	35980	O15273|Q14896|P10916|O75116|P68133|P17661|P68032|P13533|P12883|O14958|P19429|Q9Y3D2|P60660|P08590|P09493|P63313|P17081|P45379|Q8N335|P63316
GO:0030036	actin cytoskeleton organization	1.95E-04	1.16E-03	13	751	190	35980	O15273|Q14896|P10916|O75116|P68133|P68032|P13533|O14958|Q9Y3D2|P09493|P63313|P17081|P45379
GO:0030048	actin filament-based movement	3.01E-18	6.74E-17	15	95	190	35980	O15273|Q14896|P10916|P68133|P17661|P68032|P13533|P12883|P19429|P60660|P08590|P09493|P45379|Q8N335|P63316
GO:0030049	muscle filament sliding	1.08E-22	3.13E-21	14	39	190	35980	O15273|Q14896|P10916|P68133|P17661|P68032|P13533|P12883|P19429|P60660|P08590|P09493|P45379|P63316
GO:0030111	regulation of Wnt signaling pathway	1.01E-02	3.16E-02	6	343	190	35980	P0CG47|P63244|P19022|P62987|P62979|P0CG48
GO:0030154	cell differentiation	1.41E-06	1.36E-05	42	3701	190	35980	P07602|P0CG47|P10916|P01160|P16615|P62280|P0CG48|P39019|P63244|P05090|P19429|O14949|Q14192|P09493|P61247|P83731|P62979|P62158|P45379|P00441|P01034|P02144|Q14315|O15273|Q14896|P13639|O75116|P19022|P68133|P68032|Q6P6C2|P13533|P17535|P19105|O14958|P08493|P60660|P09382|P62263|P62987|P02511|P06576
GO:0030162	regulation of proteolysis	1.37E-02	3.97E-02	10	835	190	35980	P0CG47|P01033|P63244|Q9NWT8|P62987|P62979|P02511|P25116|P0CG48|P01034
GO:0030168	platelet activation	8.58E-06	7.11E-05	9	252	190	35980	P07602|P01033|P68871|P04275|P62158|P04792|P19105|P25116|P00441
GO:0030177	positive regulation of Wnt signaling pathway	1.33E-02	3.87E-02	4	172	190	35980	P0CG47|P62987|P62979|P0CG48
GO:0030178	negative regulation of Wnt signaling pathway	1.03E-03	4.92E-03	6	215	190	35980	P0CG47|P63244|P19022|P62987|P62979|P0CG48
GO:0030218	erythrocyte differentiation	7.41E-03	2.45E-02	3	75	190	35980	P02144|P62263|P39019
GO:0030239	myofibril assembly	2.25E-11	3.46E-10	9	58	190	35980	O15273|Q14896|P10916|O14958|P09493|P68133|P68032|P45379|P13533
GO:0030240	skeletal muscle thin filament assembly	1.44E-06	1.38E-05	3	5	190	35980	O15273|P68133|P68032
GO:0030260	entry into host cell	1.11E-02	3.31E-02	3	87	190	35980	P0DMV8|P0DMV9|P60033
GO:0030308	negative regulation of cell growth	2.32E-03	9.60E-03	5	173	190	35980	P63244|P16860|P10916|P01160|P02511
GO:0030330	DNA damage response, signal transduction by p53 class	1.53E-03	6.79E-03	4	93	190	35980	P0CG47|P62987|P62979|P0CG48
GO:0030490	maturation of SSU-rRNA	6.42E-04	3.25E-03	3	32	190	35980	P62263|P62249|P39019
GO:0030512	negative regulation of transforming growth factor beta receptor signaling pathway	9.13E-04	4.44E-03	4	81	190	35980	P0CG47|P62987|P62979|P0CG48
GO:0030522	intracellular receptor signaling pathway	1.52E-03	6.78E-03	6	232	190	35980	P0CG47|Q14192|P62987|P62979|Q13772|P0CG48
GO:0030901	midbrain development	1.30E-03	6.00E-03	4	89	190	35980	P18859|O14949|P14854|P62158
GO:0031032	actomyosin structure organization	2.77E-09	3.70E-08	9	98	190	35980	O15273|Q14896|P10916|O14958|P09493|P68133|P68032|P45379|P13533
GO:0031098	stress-activated protein kinase signaling cascade	2.39E-04	1.38E-03	6	163	190	35980	P0CG47|P51452|P62987|P62979|P02511|P0CG48
GO:0031145	anaphase-promoting complex-dependent proteasomal ubiquitin-dependent protein catabolic process	1.14E-03	5.40E-03	4	86	190	35980	P0CG47|P62987|P62979|P0CG48
GO:0031324	negative regulation of cellular metabolic process	4.85E-04	2.55E-03	28	2707	190	35980	P0CG47|P0DMV8|P0DMV9|Q8N3K9|P0CG48|P63244|P05090|P84243|Q14192|P62979|P62158|Q01628|P01034|P51452|Q13765|P01033|P04406|O75116|P16989|P17535|P62263|P62987|Q14240|P02511|P04792|P60228|P20585|Q8N335
GO:0031325	positive regulation of cellular metabolic process	1.20E-03	5.64E-03	30	3158	190	35980	P07602|P0CG47|P0DMV8|P0DMV9|Q969T9|Q8TAK5|P0CG48|P63244|P60033|Q14192|P68871|P62979|P62158|P00441|P01034|Q13765|Q14116|P13639|O75116|P19022|P16989|Q9Y6H1|P17535|Q05639|Q9NWT8|P62987|Q13772|P17081|P04792|P25116
GO:0031328	positive regulation of cellular biosynthetic process	2.45E-04	1.41E-03	23	1937	190	35980	P0CG47|Q13765|P0DMV8|P13639|P0DMV9|Q969T9|P16989|Q8TAK5|Q9Y6H1|P17535|P0CG48|P63244|P60033|Q14192|P68871|P62987|P62979|Q13772|P62158|P17081|P04792|P25116|P01034
GO:0031329	regulation of cellular catabolic process	1.60E-03	7.06E-03	10	610	190	35980	P0CG47|P01033|P63244|Q05639|P68104|P60033|P62987|P62979|P0CG48|P01034
GO:0031331	positive regulation of cellular catabolic process	8.42E-03	2.72E-02	6	330	190	35980	P0CG47|P63244|P60033|P62987|P62979|P0CG48
GO:0031347	regulation of defense response	1.04E-05	8.52E-05	16	835	190	35980	P0CG47|P51452|P52179|Q14116|P0DMV8|P0DMV9|P40925|Q6P6C2|P10176|P0CG48|P39019|P05090|P62987|Q16654|P62979|P62158
GO:0031349	positive regulation of defense response	1.97E-03	8.40E-03	8	424	190	35980	P0CG47|P51452|Q14116|P62987|P62979|P62158|P0CG48|P39019
GO:0031396	regulation of protein ubiquitination	5.13E-03	1.85E-02	6	297	190	35980	P0CG47|P0DMV8|P0DMV9|P62987|P62979|P0CG48
GO:0031397	negative regulation of protein ubiquitination	1.82E-04	1.09E-03	6	155	190	35980	P0CG47|P0DMV8|P0DMV9|P62987|P62979|P0CG48
GO:0031398	positive regulation of protein ubiquitination	1.55E-02	4.33E-02	4	180	190	35980	P0CG47|P62987|P62979|P0CG48
GO:0031399	regulation of protein modification process	1.83E-03	7.92E-03	21	1974	190	35980	P07602|P0CG47|P51452|Q14116|P0DMV8|P0DMV9|Q969T9|O75116|P19022|Q8N3K9|P0CG48|P63244|P60033|P62987|P23327|P62979|P62158|P04792|P25116|Q8N335|P00441
GO:0031400	negative regulation of protein modification process	6.92E-05	4.74E-04	13	676	190	35980	P0CG47|P51452|P0DMV8|P0DMV9|O75116|Q8N3K9|P0CG48|P63244|P62987|P62979|P62158|P04792|Q8N335
GO:0031401	positive regulation of protein modification process	8.05E-03	2.64E-02	14	1275	190	35980	P07602|P0CG47|Q14116|Q969T9|O75116|P19022|P0CG48|P63244|P60033|P62987|P62979|P62158|P25116|P00441
GO:0031570	DNA integrity checkpoint	1.58E-02	4.33E-02	4	181	190	35980	P0CG47|P62987|P62979|P0CG48
GO:0031571	mitotic G1 DNA damage checkpoint	7.92E-04	3.92E-03	4	78	190	35980	P0CG47|P62987|P62979|P0CG48
GO:0031647	regulation of protein stability	5.95E-03	2.04E-02	5	216	190	35980	P0DMV8|P0DMV9|Q9P0U1|P60033|P04406
GO:0031960	response to corticosteroid	2.75E-03	1.10E-02	5	180	190	35980	P41222|P19021|P17174|P62158|Q8N8D1
GO:0032101	regulation of response to external stimulus	1.43E-03	6.49E-03	13	931	190	35980	P52179|Q14116|P51684|P40925|Q6P6C2|P10176|P39019|P05090|P62987|Q16654|P62158|P04792|P25116
GO:0032147	activation of protein kinase activity	4.89E-03	1.81E-02	8	493	190	35980	P0CG47|P60033|P62987|P62979|P62158|P25116|P00441|P0CG48
GO:0032268	regulation of cellular protein metabolic process	3.64E-05	2.67E-04	32	2838	190	35980	P07602|P0CG47|P0DMV8|P0DMV9|Q969T9|Q8N3K9|P0CG48|P63244|P60033|P23327|P62979|P62158|P00441|P01034|P51452|P01033|Q14116|P13639|P04406|O75116|P19022|P16989|P63173|Q9NWT8|P62263|P62987|Q14240|P02511|P04792|P60228|P25116|Q8N335
GO:0032269	negative regulation of cellular protein metabolic process	1.01E-04	6.52E-04	18	1231	190	35980	P0CG47|P51452|P01033|P0DMV8|P0DMV9|P04406|O75116|Q8N3K9|P0CG48|P63244|P62987|P62979|P02511|P62158|P04792|P60228|Q8N335|P01034
GO:0032270	positive regulation of cellular protein metabolic process	3.84E-03	1.47E-02	17	1557	190	35980	P07602|P0CG47|Q14116|P13639|Q969T9|O75116|P19022|P16989|P0CG48|P63244|Q9NWT8|P60033|P62987|P62979|P62158|P25116|P00441
GO:0032355	response to estradiol	8.20E-03	2.68E-02	4	149	190	35980	P13639|P19021|P02511|P01034
GO:0032386	regulation of intracellular transport	2.52E-07	2.60E-06	16	625	190	35980	P54368|Q14116|Q14896|P0DMV8|Q9P0U1|P16615|P63244|P05090|O14958|Q02818|P23327|P02511|P62158|P45379|P25116|P63316
GO:0032387	negative regulation of intracellular transport	1.10E-03	5.23E-03	5	146	190	35980	P05090|O14958|P0DMV8|P02511|P62158
GO:0032388	positive regulation of intracellular transport	1.32E-02	3.83E-02	6	364	190	35980	P54368|Q14116|P63244|Q9P0U1|P62158|P25116
GO:0032409	regulation of transporter activity	6.66E-03	2.25E-02	5	222	190	35980	O14958|P01160|P23327|P62158|Q8N335
GO:0032412	regulation of ion transmembrane transporter activity	4.69E-03	1.75E-02	5	204	190	35980	O14958|P01160|P23327|P62158|Q8N335
GO:0032479	regulation of type I interferon production	4.57E-03	1.71E-02	4	126	190	35980	P0CG47|P62987|P62979|P0CG48
GO:0032480	negative regulation of type I interferon production	1.03E-04	6.61E-04	4	46	190	35980	P0CG47|P62987|P62979|P0CG48
GO:0032481	positive regulation of type I interferon production	1.30E-03	6.00E-03	4	89	190	35980	P0CG47|P62987|P62979|P0CG48
GO:0032501	multicellular organismal process	3.49E-09	4.59E-08	74	7347	190	35980	P0CG47|P10916|P01160|P54296|P16615|P62280|P35613|P0CG48|P39019|P63244|P16860|P05090|Q01449|Q14094|O14949|P09493|P83731|P62158|P45379|P24311|P24752|P01034|Q13765|P01033|P10606|Q14896|P13639|P19021|P19022|P16989|P68032|Q08431|P19105|O14958|P63173|P08493|P60660|P09382|P02511|Q13772|Q8N335|P07602|P52179|P14854|P19429|P25705|P84243|P18859|Q14192|P68871|P23327|P62979|P00441|P02144|Q14116|O15273|O75116|P68133|P17661|P51857|Q6P6C2|P13533|P17535|P12883|P17540|P08590|P62263|P62987|P04275|P04792|P25116|P20585|P06576|P63316

GO:0032502	developmental process	1.47E-08	1.82E-07	64	6093	190	35980	P0CG47|P10916|P01160|P16615|P62280|P35613|P0CG48|P39019|P63244|P05090|O14949|P09493|P83731|P62158|P45379|P24311|P24752|P01034|Q13765|P01033|Q14896|P13639|P19021|P19022|P16989|P68032|Q08431|P19105|O14958|P63173|P08493|P60660|P09382|P02511|Q13772|P07602|P13929|P14854|P19429|P25705|P84243|P18859|Q14192|P61247|P62979|P00441|P02144|Q14315|Q14116|O15273|O75116|P68133|Q6P6C2|P13533|P17535|P12883|P08590|P62263|P62987|P04275|P25116|P20585|P06576|P63316
GO:0032677	regulation of interleukin-8 production	4.17E-03	1.58E-02	3	61	190	35980	P0DMV8|P0DMV9|P25116
GO:0032757	positive regulation of interleukin-8 production	1.75E-03	7.63E-03	3	45	190	35980	P0DMV8|P0DMV9|P25116
GO:0032774	RNA biosynthetic process	3.70E-16	7.53E-15	57	3310	190	35980	P0CG47|P01160|P62280|Q8TAK5|P0CG48|P39019|P13073|P39023|P62273|P46783|P83731|P62913|P24311|P62753|P20674|Q13765|Q07020|P10606|Q96H40|P16989|Q9Y6H1|P84098|P63173|P68104|P61353|P46777|P05386|Q13772|P60866|P46776|P05387|P62249|P25398|P09669|O00483|P14854|P10176|P62891|P42766|Q02878|Q14192|P61247|P62857|P62979|P62851|P08708|P62906|P18077|P15954|P17535|P42677|P15880|Q02543|P62263|P62987|P62945|P62269
GO:0032781	positive regulation of ATPase activity	7.91E-05	5.31E-04	4	43	190	35980	Q14896|P08590|P09493|P45379
GO:0032787	monocarboxylic acid metabolic process	9.66E-05	6.24E-04	13	699	190	35980	P13929|P04406|O14561|P19021|P51857|P35613|P41222|P00505|P07195|Q16654|Q16698|Q13011|P48735
GO:0032844	regulation of homeostatic process	3.97E-03	1.51E-02	8	476	190	35980	P63244|P16860|O14958|Q16654|P23327|P62158|P25116|Q8N335
GO:0032868	response to insulin	1.10E-04	6.99E-04	10	432	190	35980	P0CG47|P01160|P17174|P62987|Q16654|P62979|P62158|P17081|P62753|P0CG48
GO:0032869	cellular response to insulin stimulus	1.74E-04	1.05E-03	9	372	190	35980	P0CG47|P17174|P62987|Q16654|P62979|P62158|P17081|P62753|P0CG48
GO:0032870	cellular response to hormone stimulus	1.85E-04	1.10E-03	14	850	190	35980	P0CG47|Q969T9|O75116|P17535|P0CG48|Q14192|P17174|P62987|Q16654|P62979|Q13772|P62158|P17081|P62753
GO:0032879	regulation of localization	5.28E-06	4.62E-05	33	2703	190	35980	P51684|P0DMV8|P01160|P16615|P12235|P39019|P63244|P16860|P05090|P60033|P09493|Q02818|P23327|P62158|P45379|P54368|P01033|Q14116|Q08493|Q14896|Q9P0U1|Q9Y277|O75116|P19021|P19022|Q08431|O14958|P02511|P17081|P04792|P25116|Q8N335|P63316
GO:0032880	regulation of protein localization	3.39E-03	1.33E-02	13	1029	190	35980	P54368|Q14116|Q08493|Q9P0U1|P19021|P19022|P12235|P63244|P05090|Q02818|P17081|P25116|Q8N335
GO:0032970	regulation of actin filament-based process	3.15E-03	1.24E-02	8	458	190	35980	Q14896|P09493|O75116|P19021|P16615|P17081|P45379|P63316
GO:0032971	regulation of muscle filament sliding	5.78E-07	5.80E-06	3	4	190	35980	Q14896|P45379|P63316
GO:0032981	mitochondrial respiratory chain complex I assembly	1.13E-12	1.88E-11	10	62	190	35980	O00217|O95169|P51970|O43674|O14561|O43920|O95167|O95178|O96000|O15239
GO:0032984	macromolecular complex disassembly	7.76E-42	8.87E-40	37	262	190	35980	P25398|P62280|P39019|P62891|P42766|Q02878|P39023|P62273|P46783|P61247|P83731|P62857|P62913|P62979|P62851|P62753|Q07020|P08708|P62906|P18077|P42677|P84098|P15880|P63173|Q02543|Q9NWT8|P61353|P62263|P62987|P62945|P46777|P05386|P60866|P46776|P62269|P05387|P62249
GO:0032989	cellular component morphogenesis	1.37E-04	8.49E-04	19	1377	190	35980	P0CG47|O15273|Q14896|P10916|O75116|P68133|P68032|P13533|P19105|P0CG48|O14958|P60660|P09493|P62987|P83731|P62979|P62158|P45379|P00441
GO:0033036	macromolecule localization	4.88E-14	8.96E-13	50	2915	190	35980	Q969T9|P62280|P35613|P39019|P05090|P00505|P39023|P62273|P46783|P83731|P62913|P62753|Q13765|Q07020|P19022|P37108|Q9Y6H1|P84098|P63173|P61353|P46777|P05386|P60866|P46776|P05387|P62249|P07602|P25398|P62891|P42766|Q02878|P60033|P61247|P62857|P62979|P62851|Q9P0U1|P08708|O75116|P62906|P18077|Q6P6C2|P42677|P15880|Q02543|P62263|P62987|P62945|P62269|P25116
GO:0033108	mitochondrial respiratory chain complex assembly	4.71E-11	7.10E-10	10	89	190	35980	O00217|O95169|P51970|O43674|O14561|O43920|O95167|O95178|O96000|O15239
GO:0033157	regulation of intracellular protein transport	1.63E-02	4.46E-02	6	382	190	35980	P54368|Q14116|P63244|P05090|Q9P0U1|Q02818
GO:0033209	tumor necrosis factor-mediated signaling pathway	1.44E-03	6.49E-03	5	155	190	35980	P0CG47|P63244|P62987|P62979|P0CG48
GO:0033275	actin-myosin filament sliding	1.08E-22	3.13E-21	14	39	190	35980	O15273|Q14896|P10916|P68133|P17661|P68032|P13533|P12883|P19429|P60660|P08590|P09493|P45379|P63316
GO:0033365	protein localization to organelle	4.18E-32	2.44E-30	41	644	190	35980	P25398|P62280|P39019|P62891|P42766|Q02878|P60033|P39023|P62273|P46783|P61247|P83731|P62857|P62913|P62979|P62851|P62753|Q07020|Q9P0U1|P08708|P62906|P18077|P37108|Q9Y6H1|P42677|P84098|P15880|P63173|Q02543|P61353|P62263|P62987|P62945|P46777|P05386|P60866|P46776|P62269|P05387|P25116|P62249
GO:0033554	cellular response to stress	2.51E-03	1.02E-02	22	2165	190	35980	P0CG47|P51452|P52179|P0DMV8|P13639|P0DMV9|Q9P0U1|P40925|P16615|P16989|P10176|P0CG48|P05090|P09493|P62987|Q16654|P62979|P02511|Q15004|P20585|P00441|P01034
GO:0033674	positive regulation of kinase activity	1.00E-02	3.16E-02	9	675	190	35980	P0CG47|Q05639|P60033|P62987|P62979|P62158|P25116|P00441|P0CG48
GO:0033683	nucleotide-excision repair, DNA incision	5.36E-05	3.72E-04	4	39	190	35980	P0CG47|P62987|P62979|P0CG48
GO:0033692	cellular polysaccharide biosynthetic process	8.67E-05	5.65E-04	4	44	190	35980	P0CG47|P62987|P62979|P0CG48
GO:0033993	response to lipid	5.80E-06	5.02E-05	18	989	190	35980	Q14116|P13639|Q969T9|O75116|P19021|P68133|Q08431|P17535|Q8N8D1|P41222|Q14192|P17174|Q16654|P02511|Q13772|P62158|P25116|P01034
GO:0034097	response to cytokine	2.25E-04	1.31E-03	15	974	190	35980	P0CG47|P01033|P51684|P04406|P80297|P68363|P16989|P17535|P0CG48|P63244|P39023|P62987|Q14240|P62979|Q01628
GO:0034101	erythrocyte homeostasis	1.00E-03	4.79E-03	4	83	190	35980	P02144|P08708|P62263|P39019
GO:0034103	regulation of tissue remodeling	5.65E-03	1.96E-02	3	68	190	35980	Q14116|Q16654|P01034
GO:0034134	toll-like receptor 2 signaling pathway	5.66E-05	3.91E-04	5	77	190	35980	P0CG47|P51452|P62987|P62979|P0CG48
GO:0034138	toll-like receptor 3 signaling pathway	1.01E-04	6.52E-04	5	87	190	35980	P0CG47|P51452|P62987|P62979|P0CG48
GO:0034142	toll-like receptor 4 signaling pathway	2.35E-04	1.35E-03	5	104	190	35980	P0CG47|P51452|P62987|P62979|P0CG48
GO:0034146	toll-like receptor 5 signaling pathway	3.33E-05	2.48E-04	5	69	190	35980	P0CG47|P51452|P62987|P62979|P0CG48
GO:0034162	toll-like receptor 9 signaling pathway	5.31E-05	3.71E-04	5	76	190	35980	P0CG47|P51452|P62987|P62979|P0CG48
GO:0034166	toll-like receptor 10 signaling pathway	3.57E-05	2.63E-04	5	70	190	35980	P0CG47|P51452|P62987|P62979|P0CG48
GO:0034220	ion transmembrane transport	4.32E-08	4.90E-07	28	1636	190	35980	P0CG47|O75947|P09669|O00483|P14854|P16615|P36542|P10176|P0CG48|P13073|P25705|P18859|O14949|P62979|P56381|P24310|P24311|P20674|P10606|P48047|P14406|P15954|O14958|Q00325|P62987|P47985|P25116|P06576
GO:0034248	regulation of cellular amide metabolic process	8.35E-04	4.09E-03	9	462	190	35980	P63244|P13639|P63173|P04406|P62263|Q14240|P16989|P04792|P60228
GO:0034470	ncRNA processing	1.99E-04	1.18E-03	10	465	190	35980	P42766|P08708|P62263|P18077|P62857|P62913|P46777|P62753|P62249|P39019
GO:0034599	cellular response to oxidative stress	1.21E-03	5.67E-03	6	222	190	35980	P0DMV8|P0DMV9|P09493|P16615|P00441|P01034
GO:0034612	response to tumor necrosis factor	3.41E-03	1.33E-02	6	273	190	35980	P0CG47|P63244|P62987|P16989|P62979|P0CG48
GO:0034613	cellular protein localization	2.70E-19	6.41E-18	44	1641	190	35980	P25398|P62280|P35613|P39019|P62891|P42766|Q02878|P60033|P39023|P62273|P46783|P61247|P83731|P62857|P62913|P62979|P62851|P62753|Q07020|Q9P0U1|P08708|O75116|P62906|P19022|P18077|P37108|Q9Y6H1|P42677|P84098|P15880|P63173|Q02543|P61353|P62263|P62987|P62945|P46777|P05386|P60866|P46776|P62269|P05387|P25116|P62249
GO:0034622	cellular macromolecular complex assembly	3.44E-14	6.43E-13	28	885	190	35980	O43674|O95178|P39019|O96000|Q02878|P84243|P39023|P46783|P83731|P62913|O15239|O00217|O15273|Q14896|O95169|P51970|P08708|O14561|O43920|O95167|P42677|O14958|P63173|Q9Y3D2|P62263|P46777|P02511|P60228
GO:0034637	cellular carbohydrate biosynthetic process	1.97E-05	1.54E-04	5	62	190	35980	P0CG47|P17174|P62987|P62979|P0CG48
GO:0034641	cellular nitrogen compound metabolic process	1.26E-27	5.87E-26	112	8039	190	35980	O75947|O43674|O96000|P16860|P13073|P07195|P46783|P83731|P62913|P62753|P24752|O15239|P20674|Q13765|O95169|P13639|P48047|P04406|O14561|Q9Y5N6|P16989|Q9Y6H1|O95167|P84098|P63173|P68104|P46777|Q13772|P60866|P46776|P60228|P62249|Q8N335|P25398|P10176|O95178|P12235|P62891|P25705|P18859|Q14192|P56381|P00441|P54368|O00217|Q08493|P08708|P18077|Q6P6C2|P17535|P12883|Q8N8D1|P42677|P15880|P17540|Q02543|Q9NWT8|P62263|Q14240|P62945|P62269|P0CG47|P06732|Q9NSD9|P0DMV8|P0DMV9|P01160|P40926|P40925|P62280|Q8TAK5|P36542|P0CG48|P39019|P00505|P39023|P62273|P24311|Q8WYQ3|Q07020|P10606|Q96H40|P19021|P37108|O43920|Q05639|P61353|P05386|P17081|P05387|P13929|P09669|O00483|P14854|P42766|Q02878|P84243|Q9NPI5|P17174|P61247|P62857|P62979|Q15004|P62851|P51970|P62906|P15954|P13533|Q00325|P62987|P20585|P06576
GO:0034645	cellular macromolecule biosynthetic process	2.51E-13	4.46E-12	68	5231	190	35980	P0CG47|Q9NSD9|P01160|P62280|Q8TAK5|P0CG48|P39019|P13073|P39023|P62273|P46783|P83731|P62913|P24311|P62753|P20674|Q13765|Q07020|P10606|Q96H40|P13639|Q9Y5N6|P37108|P16989|Q9Y6H1|P84098|Q05639|P63173|P68104|P61353|P46777|P05386|Q13772|P60866|P46776|P60228|P62249|P25398|P09669|O00483|P14854|P10176|P12235|P62891|P42766|Q02878|Q14192|P61247|P62857|P62979|Q15004|P62851|P08708|P62906|P18077|P15954|P17535|P42677|P15880|Q02543|Q9NWT8|Q00325|P62263|P62987|Q14240|P62945|P62269

GO:0034654	nucleobase-containing compound biosynthetic process	3.06E-20	7.71E-19	68	3838	190	35980	P0CG47|O75947|P01160|P62280|Q8TAK5|P36542|P0CG48|P39019|P16860|P13073|P39023|P62273|P46783|P83731|P62913|P24311|P62753|Q8WYQ3|P20674|Q13765|Q07020|P10606|Q96H40|P48047|P16989|Q9Y6H1|P84098|P63173|P68104|P61353|P46777|P05386|Q13772|P60866|P46776|P05387|P62249|P25398|P09669|O00483|P14854|P10176|P62891|P42766|Q02878|P25705|P18859|Q14192|Q9NPI5|P61247|P62857|P62979|P56381|Q15004|P62851|P08708|P62906|P18077|P15954|P17535|P42677|P15880|Q02543|P62263|P62987|P62945|P62269|P06576
GO:0034655	nucleobase-containing compound catabolic process	1.28E-37	1.27E-35	39	401	190	35980	P25398|P62280|P39019|P62891|P42766|Q02878|P39023|P62273|P46783|P61247|P83731|P62857|P62913|P62979|P62851|P62753|Q07020|Q08493|P08708|P62906|P18077|P42677|P84098|P15880|P63173|Q02543|P61353|P62263|P62987|Q14240|P62945|P46777|P05386|P60866|P46776|P62269|P05387|P60228|P62249
GO:0034660	ncRNA metabolic process	1.70E-03	7.46E-03	11	723	190	35980	P42766|Q9NSD9|P08708|P62263|P18077|P62857|P62913|P46777|P62753|P62249|P39019
GO:0034762	regulation of transmembrane transport	9.86E-03	3.11E-02	8	557	190	35980	P54368|O14958|Q9Y277|P01160|P23327|P62158|P25116|Q8N335
GO:0034763	negative regulation of transmembrane transport	1.22E-02	3.60E-02	3	90	190	35980	P54368|O14958|P62158
GO:0035051	cardiocyte differentiation	3.85E-05	2.81E-04	6	117	190	35980	O15273|P10916|Q14192|P01160|P68032|P13533
GO:0035150	regulation of tube size	1.99E-03	8.47E-03	5	167	190	35980	P16860|P01160|P68871|P25116|P00441
GO:0035637	multicellular organismal signaling	2.26E-03	9.40E-03	5	172	190	35980	O14958|P19429|P01160|P16615|P00441
GO:0035666	TRIF-dependent toll-like receptor signaling pathway	7.21E-05	4.89E-04	5	81	190	35980	P0CG47|P51452|P62987|P62979|P0CG48
GO:0035872	nucleotide-binding domain, leucine rich repeat containing receptor signaling pathway	1.03E-04	6.61E-04	4	46	190	35980	P0CG47|P62987|P62979|P0CG48
GO:0036293	response to decreased oxygen levels	6.99E-06	5.91E-05	10	312	190	35980	P0CG47|P02144|P01160|P19021|P62987|P62979|P02511|Q6P6C2|P0CG48|P01034
GO:0036294	cellular response to decreased oxygen levels	6.78E-03	2.28E-02	4	141	190	35980	P0CG47|P62987|P62979|P0CG48
GO:0036297	interstrand cross-link repair	7.21E-05	4.89E-04	4	42	190	35980	P0CG47|P62987|P62979|P0CG48
GO:0038061	NIK/NF-kappaB signaling	1.92E-03	8.23E-03	4	99	190	35980	P0CG47|P62987|P62979|P0CG48
GO:0038123	toll-like receptor TLR1:TLR2 signaling pathway	5.31E-05	3.71E-04	5	76	190	35980	P0CG47|P51452|P62987|P62979|P0CG48
GO:0038124	toll-like receptor TLR6:TLR2 signaling pathway	5.31E-05	3.71E-04	5	76	190	35980	P0CG47|P51452|P62987|P62979|P0CG48
GO:0040008	regulation of growth	3.51E-04	1.94E-03	12	695	190	35980	Q13765|P63244|P16860|P10916|P84243|P60033|P01160|P80297|P16989|P02511|P13533|P00441
GO:0040011	locomotion	4.58E-03	1.71E-02	17	1585	190	35980	P0CG47|P51684|P0DMV8|P0DMV9|O75116|P19022|P35613|P19105|P0CG48|P39019|P60033|P60660|P62987|P83731|P62979|P62158|P06576
GO:0040012	regulation of locomotion	4.51E-03	1.69E-02	11	822	190	35980	P01033|P63244|P05090|P51684|P60033|P09382|P09493|O75116|P04792|Q01628|P25116
GO:0042058	regulation of epidermal growth factor receptor signaling	9.56E-04	4.63E-03	4	82	190	35980	P0CG47|P62987|P62979|P0CG48
GO:0042059	negative regulation of epidermal growth factor receptor	7.21E-05	4.89E-04	4	42	190	35980	P0CG47|P62987|P62979|P0CG48
GO:0042060	wound healing	1.32E-06	1.28E-05	16	709	190	35980	P07602|Q13765|P01033|P13929|P16615|P35613|P19105|P05090|P84243|P68871|P09493|P04275|P62158|P04792|P25116|P00441
GO:0042176	regulation of protein catabolic process	4.17E-04	2.26E-03	9	419	190	35980	P0CG47|P54368|P01033|P63244|P60033|P62987|P62979|P0CG48|P01034
GO:0042221	response to chemical	1.53E-18	3.47E-17	74	4840	190	35980	P0CG47|P0DMV8|P0DMV9|Q12988|Q969T9|P01160|P16615|P35613|P0CG48|P39019|P63244|P05090|P13073|P00505|P39023|P09493|Q02818|P83731|P69905|P62158|P45379|P62753|P24752|P01034|P01033|P13639|P04406|O14880|P80297|P19021|P37108|P16989|P68032|Q08431|P19105|O14958|Q05639|P68104|P60660|P09382|P08574|P47985|P02511|Q13772|P17081|P07602|P13929|P51684|P68363|P84243|P60033|Q14192|P68871|Q9UBY9|P17174|Q16654|P22352|P62979|Q01628|P00441|P51452|P02144|Q14116|O75116|P68133|P13533|P17535|Q8N8D1|P41222|P62987|Q14240|P04792|P25116|P63316
GO:0042246	tissue regeneration	3.98E-03	1.51E-02	3	60	190	35980	Q13765|P13929|P05090
GO:0042254	ribosome biogenesis	5.02E-12	8.07E-11	16	293	190	35980	P08708|P18077|P39019|P42677|P42766|Q02878|P63173|P39023|P62263|P46783|P83731|P62857|P62913|P46777|P62753|P62249
GO:0042255	ribosome assembly	3.22E-14	6.07E-13	11	63	190	35980	P42677|Q02878|P63173|P08708|P39023|P62263|P46783|P83731|P62913|P46777|P39019
GO:0042273	ribosomal large subunit biogenesis	2.32E-10	3.34E-09	8	50	190	35980	P42766|Q02878|P63173|P39023|P18077|P83731|P62913|P46777
GO:0042274	ribosomal small subunit biogenesis	6.63E-12	1.05E-10	9	51	190	35980	P42677|P63173|P08708|P62263|P46783|P62857|P62753|P62249|P39019
GO:0042276	error-prone translesion synthesis	2.74E-06	2.52E-05	4	19	190	35980	P0CG47|P62987|P62979|P0CG48
GO:0042278	purine nucleoside metabolic process	2.17E-22	6.15E-21	29	471	190	35980	P13929|O75947|P0DMV8|P0DMV9|O43674|O00483|P36542|P10176|O95178|O96000|P13073|P25705|P18859|P56381|Q8WYQ3|O15239|P20674|O00217|O95169|P51970|P48047|P04406|O14561|O43920|P13533|O95167|P12883|P17081|P06576
GO:0042325	regulation of phosphorylation	2.58E-03	1.05E-02	18	1628	190	35980	P07602|P0CG47|P51452|Q14116|O75116|P19022|P0CG48|P63244|Q05639|P60033|P62987|P23327|P62979|P62158|P04792|P25116|Q8N335|P00441
GO:0042327	positive regulation of phosphorylation	2.24E-03	9.31E-03	14	1100	190	35980	P07602|P0CG47|Q14116|O75116|P19022|P0CG48|P63244|Q05639|P60033|P62987|P62979|P62158|P25116|P00441
GO:0042330	taxis	7.38E-03	2.44E-02	11	880	190	35980	P0CG47|P51684|P60660|O75116|P62987|P83731|P62979|P62158|P19105|P0CG48|P39019
GO:0042391	regulation of membrane potential	3.72E-03	1.43E-02	7	371	190	35980	P0CG47|P63244|O14958|P01160|P16615|Q8N335|P00441
GO:0042451	purine nucleoside biosynthetic process	1.45E-06	1.38E-05	8	150	190	35980	O75947|P25705|P48047|P18859|P36542|P56381|Q8WYQ3|P06576
GO:0042455	ribonucleoside biosynthetic process	4.96E-06	4.38E-05	8	177	190	35980	O75947|P25705|P48047|P18859|P36542|P56381|Q8WYQ3|P06576
GO:0042493	response to drug	3.57E-05	2.63E-04	11	458	190	35980	P13929|P05090|P13639|P09382|P19021|P47985|P37108|P68032|P17535|P00441|P01034
GO:0042542	response to hydrogen peroxide	2.78E-06	2.53E-05	7	115	190	35980	P02144|P13639|P68871|P02511|P69905|P00441|P01034
GO:0042590	antigen processing and presentation of exogenous peptide antigen via MHC class I	7.65E-03	2.52E-02	4	146	190	35980	P0CG47|P62987|P62979|P0CG48
GO:0042592	homeostatic process	1.25E-05	1.00E-04	23	1590	190	35980	P0CG47|P02144|P02792|P51684|P08708|P19022|P16615|P0CG48|P39019|P13693|P63244|O14958|P19429|P09382|P62263|P17174|Q16654|P23327|P04792|P62753|P25116|P00441|P06576
GO:0042692	muscle cell differentiation	5.11E-11	7.63E-10	15	290	190	35980	Q14315|O15273|Q14896|P10916|P01160|P19022|P68133|P16615|P68032|P13533|O14958|Q14192|P09382|P09493|P45379
GO:0042743	hydrogen peroxide metabolic process	2.41E-05	1.85E-04	4	32	190	35980	P68871|P22352|P69905|P00441
GO:0042744	hydrogen peroxide catabolic process	1.32E-04	8.21E-04	3	19	190	35980	P68871|P22352|P69905
GO:0042769	DNA damage response, detection of DNA damage	3.89E-05	2.81E-04	4	36	190	35980	P0CG47|P62987|P62979|P0CG48
GO:0042770	signal transduction in response to DNA damage	2.63E-03	1.06E-02	4	108	190	35980	P0CG47|P62987|P62979|P0CG48
GO:0042773	ATP synthesis coupled electron transport	1.55E-17	3.37E-16	14	83	190	35980	O00217|O95169|P51970|O43674|O14561|O00483|O43920|O95167|P10176|O95178|O96000|P13073|O15239|P20674
GO:0042775	mitochondrial ATP synthesis coupled electron transport	5.07E-18	1.12E-16	14	77	190	35980	O00217|O95169|P51970|O43674|O14561|O00483|O43920|O95167|P10176|O95178|O96000|P13073|O15239|P20674
GO:0042776	mitochondrial ATP synthesis coupled proton transport	4.89E-12	7.99E-11	7	19	190	35980	O75947|P25705|P48047|P18859|P36542|P56381|P06576
GO:0042981	regulation of apoptotic process	3.10E-05	2.32E-04	23	1685	190	35980	P0CG47|Q13765|P01033|P0DMV8|P0DMV9|P16989|P68032|P0CG48|Q8N8D1|P13693|P63244|Q05639|Q14192|P09382|P62987|P61247|Q16654|P62979|P02511|P04792|P62753|P25116|P00441
GO:0043043	peptide biosynthetic process	1.35E-30	7.32E-29	47	1020	190	35980	P25398|Q9NSD9|P62280|P12235|P39019|P62891|P42766|Q02878|P39023|P62273|P46783|P61247|P83731|P62857|P62913|P62979|P62851|P62753|Q13765|Q07020|P13639|P08708|P62906|P18077|P37108|P42677|P84098|P15880|Q05639|P63173|P68104|Q02543|Q9NWT8|P61353|Q00325|P62263|P62987|Q14240|P62945|P46777|P05386|P60866|P46776|P62269|P05387|P60228|P62249
GO:0043065	positive regulation of apoptotic process	2.73E-03	1.09E-02	10	657	190	35980	P0CG47|P63244|Q05639|P62987|P62979|P62753|P25116|P00441|P0CG48|Q8N8D1
GO:0043066	negative regulation of apoptotic process	2.93E-06	2.66E-05	18	941	190	35980	P0CG47|Q13765|P01033|P0DMV8|P0DMV9|P16989|P68032|P0CG48|P13693|Q14192|P62987|P61247|Q16654|P62979|P02511|P04792|P25116|P00441
GO:0043067	regulation of programmed cell death	3.72E-06	3.31E-05	25	1698	190	35980	P0CG47|P0DMV8|P0DMV9|P12235|P0CG48|P13693|P63244|Q14192|P61247|Q16654|P62979|P62753|P00441|P01034|Q13765|P01033|P16989|P68032|Q8N8D1|Q05639|P09382|P62987|P02511|P04792|P25116
GO:0043068	positive regulation of programmed cell death	2.88E-03	1.14E-02	10	662	190	35980	P0CG47|P63244|Q05639|P62987|P62979|P62753|P25116|P00441|P0CG48|Q8N8D1
GO:0043069	negative regulation of programmed cell death	8.07E-07	7.97E-06	19	951	190	35980	P0CG47|Q13765|P01033|P0DMV8|P0DMV9|P16989|P68032|P12235|P0CG48|P13693|Q14192|P62987|P61247|Q16654|P62979|P02511|P04792|P25116|P00441
GO:0043086	negative regulation of catalytic activity	4.54E-05	3.23E-04	17	1048	190	35980	P0CG47|P51452|P54368|P01033|O75116|Q8N3K9|P0CG48|P63244|P19429|P62987|Q14240|P62979|P02511|P04792|P45379|Q8WYQ3|P01034
GO:0043122	regulation of I-kappaB kinase/NF-kappaB signaling	5.62E-05	3.89E-04	8	248	190	35980	P0CG47|Q14116|P09382|P62987|P62979|P04792|P25116|P0CG48
GO:0043123	positive regulation of I-kappaB kinase/NF-kappaB	7.95E-05	5.33E-04	7	193	190	35980	P0CG47|Q14116|P09382|P62987|P62979|P25116|P0CG48

GO:0043170	macromolecule metabolic process	2.06E-04	1.22E-03	93	13022	190	35980	P0CG47|Q9NSD9|P0DMV8|P01160|P40926|P62280|Q8TAK5|P0CG48|P39019|P63244|P13073|P39023|P62273|P46783|P83731|P62913|P62158|P24311|P62753|P20674|P01034|Q13765|Q07020|P10606|Q96H40|P13639|P04406|O14561|P19021|Q9Y5N6|P37108|P16989|Q9Y6H1|Q08431|P84098|Q05639|P63173|P68104|P61353|P46777|P02511|P05386|Q13772|P60866|P46776|P05387|P60228|P62249|P25398|P09669|P68363|O00483|P14854|P10176|P12235|P62891|P42766|Q02878|P84243|P60033|Q14192|P61247|P62857|Q16654|P62979|Q15004|P62851|P51452|Q14116|Q9P0U1|P08708|O75116|P62906|P18077|P15954|Q6P6C2|P17535|Q8N8D1|P42677|P15880|Q02543|Q9NWT8|Q9Y3D2|Q00325|P62263|P62987|Q14240|P62945|P04275|P04792|P62269|P25116|P20585
GO:0043207	response to external biotic stimulus	1.43E-03	6.49E-03	13	931	190	35980	P52179|Q14116|P40925|Q6P6C2|P10176|P17535|P13693|P62891|P62987|Q16654|P04792|Q01628|P25116
GO:0043241	protein complex disassembly	2.00E-42	2.48E-40	37	253	190	35980	P25398|P62280|P39019|P62891|P42766|Q02878|P39023|P62273|P46783|P61247|P83731|P62857|P62913|P62979|P62851|P62753|Q07020|P08708|P62906|P18077|P42677|P84098|P15880|P63173|Q02543|Q9NWT8|P61353|P62263|P62987|P62945|P46777|P05386|P60866|P46776|P62269|P05387|P62249
GO:0043405	regulation of MAP kinase activity	1.31E-03	6.05E-03	9	493	190	35980	P0CG47|P51452|P60033|P62987|P62979|P62158|P25116|P00441|P0CG48
GO:0043406	positive regulation of MAP kinase activity	1.05E-03	5.03E-03	6	216	190	35980	P0CG47|P60033|P62987|P62979|P00441|P0CG48
GO:0043408	regulation of MAPK cascade	5.56E-03	1.93E-02	11	846	190	35980	P07602|P0CG47|P51452|P60033|P19022|P62987|P62979|P62158|P25116|P00441|P0CG48
GO:0043410	positive regulation of MAPK cascade	2.31E-03	9.57E-03	10	642	190	35980	P07602|P0CG47|P60033|P19022|P62987|P62979|P62158|P25116|P00441|P0CG48
GO:0043434	response to peptide hormone	6.75E-07	6.71E-06	15	591	190	35980	P0CG47|P01033|P01160|P16615|P35613|P17535|P0CG48|P17174|P08574|P62987|Q16654|P62979|P62158|P17081|P62753
GO:0043436	oxoacid metabolic process	1.89E-30	1.01E-28	56	1605	190	35980	P06732|Q9NSD9|P40926|P40925|P62280|P35613|P39019|P00505|P39023|P62273|P07195|P46783|P83731|P62913|P62753|P24752|Q07020|P04406|O14561|P19021|P84098|P63173|P61353|P46777|P05386|P60866|P46776|P05387|P62249|Q13011|P13929|P25398|P62891|P42766|Q02878|P17174|P61247|P62857|Q16654|Q16698|P62979|P62851|P08708|P62906|P18077|P51857|P42677|P41222|P15880|P17540|Q02543|P62263|P62987|P62945|P62269|P48735
GO:0043462	regulation of ATPase activity	5.51E-08	6.20E-07	7	65	190	35980	Q14896|P19429|P08590|P09493|P45379|P13533|P63316
GO:0043487	regulation of RNA stability	1.22E-05	9.89E-05	7	144	190	35980	P0CG47|P0DMV8|P62987|P16989|P62979|P04792|P0CG48
GO:0043488	regulation of mRNA stability	1.02E-05	8.39E-05	7	140	190	35980	P0CG47|P0DMV8|P62987|P16989|P62979|P04792|P0CG48
GO:0043500	muscle adaptation	6.42E-04	3.25E-03	3	32	190	35980	O15273|P01160|P68133
GO:0043502	regulation of muscle adaptation	5.55E-04	2.86E-03	4	71	190	35980	P16615|Q8N3K9|P12883|P63316
GO:0043549	regulation of kinase activity	8.60E-03	2.76E-02	12	1025	190	35980	P0CG47|P51452|P63244|Q05639|P60033|P62987|P62979|P62158|P04792|P25116|P00441|P0CG48
GO:0043603	cellular amide metabolic process	1.34E-26	5.76E-25	49	1396	190	35980	Q9NSD9|P62280|P39019|P39023|P62273|P46783|P83731|P62913|P62753|Q13765|Q07020|P13639|P19021|P37108|P84098|Q05639|P63173|P68104|P61353|P46777|P05386|P60866|P46776|P05387|P60228|P62249|P25398|P12235|P62891|P42766|Q02878|P61247|P62857|P62979|P62851|P00441|P08708|P62906|P18077|P42677|P15880|Q02543|Q9NWT8|Q00325|P62263|P62987|Q14240|P62945|P62269
GO:0043604	amide biosynthetic process	7.21E-29	3.57E-27	47	1117	190	35980	P25398|Q9NSD9|P62280|P12235|P39019|P62891|P42766|Q02878|P39023|P62273|P46783|P61247|P83731|P62857|P62913|P62979|P62851|P62753|Q13765|Q07020|P13639|P08708|P62906|P18077|P37108|P42677|P84098|P15880|Q05639|P63173|P68104|Q02543|Q9NWT8|P61353|Q00325|P62263|P62987|Q14240|P62945|P46777|P05386|P60866|P46776|P62269|P05387|P60228|P62249
GO:0043618	regulation of transcription from RNA polymerase II promoter in response to stress	3.31E-04	1.86E-03	4	62	190	35980	P0CG47|P62987|P62979|P0CG48
GO:0043620	regulation of DNA-templated transcription in response to	4.45E-04	2.39E-03	4	67	190	35980	P0CG47|P62987|P62979|P0CG48
GO:0043623	cellular protein complex assembly	1.61E-08	1.99E-07	15	442	190	35980	O00217|O15273|Q14896|O95169|P51970|O43674|O14561|O43920|O95167|O95178|O96000|O14958|Q9Y3D2|P02511|O15239
GO:0043624	cellular protein complex disassembly	1.71E-44	2.31E-42	37	224	190	35980	P25398|P62280|P39019|P62891|P42766|Q02878|P39023|P62273|P46783|P61247|P83731|P62857|P62913|P62979|P62851|P62753|Q07020|P08708|P62906|P18077|P42677|P84098|P15880|P63173|Q02543|Q9NWT8|P61353|P62263|P62987|P62945|P46777|P05386|P60866|P46776|P62269|P05387|P62249
GO:0043627	response to estrogen	1.39E-03	6.35E-03	6	228	190	35980	P13639|Q969T9|P19021|P02511|Q08431|P01034
GO:0043648	dicarboxylic acid metabolic process	3.45E-04	1.91E-03	5	113	190	35980	P00505|P40926|P40925|P17174|P48735
GO:0043666	regulation of phosphoprotein phosphatase activity	1.37E-02	3.96E-02	3	94	190	35980	O75116|Q8N3K9|P62158
GO:0043900	regulation of multi-organism process	3.47E-04	1.92E-03	10	499	190	35980	P52179|P01033|P41222|P09382|P40925|P62987|Q16654|Q6P6C2|P10176|Q01628
GO:0043933	macromolecular complex subunit organization	2.72E-25	1.08E-23	67	3020	190	35980	O43674|P62280|P39019|O96000|P39023|P62273|P46783|P83731|P62913|P69905|P45379|P62753|P24752|O15239|Q07020|Q14896|O95169|O14561|P19021|P19022|O43920|P68032|O95167|P84098|O14958|P63173|P61353|P46777|P02511|P05386|P60866|P46776|P05387|P60228|P62249|P25398|O95178|P62891|P42766|Q02878|P84243|P68871|P61247|P62857|P22352|Q16698|P62979|P62851|O00217|O15273|P51970|P08708|P62906|P68133|P18077|P17661|P42677|P15880|Q02543|Q9NWT8|Q9Y3D2|P62263|P62987|P62945|P04275|P63313|P62269
GO:0044033	multi-organism metabolic process	3.79E-56	2.82E-53	38	130	190	35980	P0CG47|P25398|P62280|P0CG48|P39019|P62891|P42766|Q02878|P39023|P62273|P46783|P61247|P83731|P62857|P62913|P62979|P62851|P62753|Q07020|P08708|P62906|P18077|P42677|P84098|P15880|P63173|Q02543|P61353|P62263|P62987|P62945|P46777|P05386|P60866|P46776|P62269|P05387|P62249
GO:0044042	glucan metabolic process	2.88E-05	2.17E-04	5	67	190	35980	P0CG47|P62987|P62979|P62158|P0CG48
GO:0044057	regulation of system process	4.48E-13	7.75E-12	22	568	190	35980	Q14896|P10916|P01160|P16615|P17661|Q8N3K9|P13533|P12883|P16860|O14958|P19429|Q9UBY9|P08590|P09493|P23327|P62158|P45379|P25116|O14558|Q8N335|P00441|P63316
GO:0044085	cellular component biogenesis	2.53E-16	5.18E-15	52	2752	190	35980	P0CG47|P10916|O43674|P0CG48|P39019|O96000|P39023|P09493|P46783|P83731|P62913|P69905|P45379|P62753|P24752|O15239|Q14896|O95169|O14561|P19021|P19022|O43920|P68032|O95167|O14958|P63173|P46777|P02511|P60228|P62249|O95178|P42766|Q02878|P84243|P68871|P62857|P22352|Q16698|P62979|O00217|Q14315|O15273|P51970|P08708|P68133|P18077|P13533|P42677|Q9Y3D2|P62263|P62987|P04275
GO:0044092	negative regulation of molecular function	7.18E-05	4.89E-04	19	1310	190	35980	P0CG47|P51452|P54368|P01033|O75116|Q8N3K9|P0CG48|P63244|O14958|P19429|P62987|Q14240|P62979|P02511|P62158|P04792|P45379|Q8WYQ3|P01034
GO:0044237	cellular metabolic process	5.94E-21	1.56E-19	140	14451	190	35980	O75947|O43674|O96000|P16860|P13073|O14949|P07195|P46783|P83731|P62913|P62753|P24752|O15239|P20674|Q13765|O95169|P13639|P48047|P04406|O14561|Q9Y5N6|P16989|Q9Y6H1|O95167|Q08431|P84098|P63173|P68104|P08574|P47985|P46777|Q13772|P60866|P46776|P60228|P62249|Q13011|Q8N335|P07602|P25398|P68363|P10176|O95178|P12235|P62891|P25705|P18859|Q14192|P68871|Q16654|P56381|P00441|P54368|O00217|Q14116|Q08493|P08708|O75116|P18077|Q6P6C2|P17535|P12883|Q8N8D1|P42677|P41222|P15880|P17540|Q02543|Q9NWT8|Q9Y3D2|P62263|Q14240|P62945|P62269|P0CG47|P06732|Q9NSD9|P0DMV8|P0DMV9|P01160|P40926|P40925|P62280|Q8TAK5|P36542|P35613|P0CG48|P39019|P00505|P39023|P62273|P24310|P69905|P62158|P24311|Q8WYQ3|P01034|Q07020|P10606|Q96H40|O14880|P19021|P37108|O43920|Q05639|P61353|P02511|P05386|P17081|P05387|P13929|P09669|O00483|P14854|P42766|Q02878|P84243|P60033|Q9NPI5|P17174|P61247|P62857|P22352|Q16698|P62979|Q15004|P62851|P51452|P51970|Q9P0U1|P62906|P15954|P51857|P13533|Q00325|P62987|P25116|P20585|P06576|P48735
GO:0044238	primary metabolic process	2.73E-13	4.81E-12	132	15593	190	35980	O75947|O43674|O96000|P16860|P13073|P07195|P46783|P83731|P62913|P62753|P24752|O15239|P20674|Q13765|O95169|P13639|P48047|P04406|O14561|Q9Y5N6|P16989|Q9Y6H1|O95167|Q08431|P84098|P63173|P68104|P46777|Q13772|P60866|P46776|P60228|P62249|Q13011|Q8N335|P07602|P25398|P68363|P10176|O95178|P12235|P62891|P25705|P18859|Q14192|Q16654|P56381|O00217|Q14116|Q08493|P08708|O75116|P18077|Q6P6C2|P17535|P12883|Q8N8D1|P42677|P41222|P15880|P17540|Q02543|Q9NWT8|Q9Y3D2|P62263|Q14240|P62945|P62269|P0CG47|P06732|Q9NSD9|P0DMV8|P0DMV9|P01160|P40926|P40925|P62280|Q8TAK5|P36542|P0CG48|P39019|P63244|P05090|P00505|P39023|P62273|P62158|P24311|Q8WYQ3|P01034|Q07020|P10606|Q96H40|O14880|P19021|P37108|O43920|Q05639|P61353|P02511|P05386|P17081|P05387|P13929|P09669|O00483|P14854|P42766|Q02878|P84243|Q9NPI5|P17174|P61247|P62857|Q16698|P62979|Q15004|P62851|P51452|P51970|Q9P0U1|P62906|P15954|P51857|P13533|Q00325|P62987|P04275|P25116|P20585|P06576|P48735

<table>
<tr><td></td><td></td><td></td><td></td><td></td><td></td><td></td><td></td><td></td></tr>
<tr><td>GO:0044248</td><td>cellular catabolic process</td><td>8.42E-25</td><td>3.13E-23</td><td>51</td><td>1684</td><td>190</td><td>35980</td><td>P0CG47|P62280|P0CG48|P39019|P00505|P39023|P62273|P46783|P83731|P62913|P69905|P62158|P62753|P24752|Q07020|P84098|P63173|P61353|P46777|P05386|P60866|P46776|P05387|P60228|P62249|Q13011|P25398|P62891|P42766|Q02878|P68871|P17174|P61247|P62857|P22352|Q16698|P62979|P62851|Q08493|P08708|P62906|P18077|P51857|P42677|P15880|Q02543|P62263|P62987|Q14240|P62945|P62269</td></tr>
<tr><td>GO:0044249</td><td>cellular biosynthetic process</td><td>1.09E-18</td><td>2.51E-17</td><td>88</td><td>6618</td><td>190</td><td>35980</td><td>P0CG47|O75947|Q9NSD9|P01160|P62280|Q8TAK5|P36542|P0CG48|P39019|P16860|P13073|P00505|P39023|P62273|P46783|P83731|P62913|P24311|P62753|Q8WYQ3|P24752|P20674|Q13765|Q07020|P10606|Q96H40|P13639|P48047|O14561|O14880|Q9Y5N6|P37108|P16989|Q9Y6H1|P84098|Q05639|P63173|P68104|P61353|P46777|P05386|Q13772|P60866|P46776|P05387|P60228|P62249|Q8N335|P25398|P09669|O00483|P14854|P10176|P12235|P62891|P42766|Q02878|P25705|P18859|Q14192|Q9NPI5|P17174|P61247|P62857|P62979|P56381|Q15004|P62851|P00441|P54368|P08708|P62906|P18077|P15954|P51857|P17535|P42677|P41222|P15880|Q02543|Q9NWT8|Q00325|P62263|P62987|Q14240|P62945|P62269|P06576</td></tr>
<tr><td>GO:0044260</td><td>cellular macromolecule metabolic process</td><td>4.88E-06</td><td>4.32E-05</td><td>89</td><td>11251</td><td>190</td><td>35980</td><td>P0CG47|Q9NSD9|P01160|P40926|P62280|Q8TAK5|P0CG48|P39019|P13073|P39023|P62273|P46783|P83731|P62913|P62158|P24311|P62753|P20674|P01034|Q13765|Q07020|P10606|Q96H40|P13639|P04406|O14561|P19021|Q9Y5N6|P37108|P16989|Q9Y6H1|Q08431|P84098|Q05639|P63173|P68104|P61353|P46777|P02511|P05386|Q13772|P60866|P46776|P05387|P60228|P62249|P25398|P09669|P68363|O00483|P14854|P10176|P12235|P62891|P42766|Q02878|P84243|P60033|Q14192|P61247|P62857|Q16654|P62979|Q15004|P62851|P51452|Q14116|Q9P0U1|P08708|O75116|P62906|P18077|P15954|Q6P6C2|P17535|Q8N8D1|P42677|P15880|Q02543|Q9NWT8|Q9Y3D2|Q00325|P62263|P62987|Q14240|P62945|P62269|P25116|P20585</td></tr>
<tr><td>GO:0044262</td><td>cellular carbohydrate metabolic process</td><td>1.19E-04</td><td>7.49E-04</td><td>7</td><td>206</td><td>190</td><td>35980</td><td>P0CG47|P17174|P62987|P62979|P62158|P0CG48|P48735</td></tr>
<tr><td>GO:0044264</td><td>cellular polysaccharide metabolic process</td><td>8.10E-05</td><td>5.40E-04</td><td>5</td><td>83</td><td>190</td><td>35980</td><td>P0CG47|P62987|P62979|P62158|P0CG48</td></tr>
<tr><td>GO:0044265</td><td>cellular macromolecule catabolic process</td><td>2.97E-23</td><td>8.93E-22</td><td>40</td><td>1029</td><td>190</td><td>35980</td><td>P0CG47|P25398|P62280|P0CG48|P39019|P62891|P42766|Q02878|P39023|P62273|P46783|P61247|P83731|P62857|P62913|P62979|P62851|P62753|Q07020|P08708|P62906|P18077|P42677|P84098|P15880|P63173|Q02543|P61353|P62263|P62987|Q14240|P62945|P46777|P05386|P60866|P46776|P62269|P05387|P60228|P62249</td></tr>
<tr><td>GO:0044267</td><td>cellular protein metabolic process</td><td>3.63E-09</td><td>4.75E-08</td><td>68</td><td>6461</td><td>190</td><td>35980</td><td>P0CG47|Q9NSD9|P01160|P40926|P62280|P0CG48|P39019|P39023|P62273|P46783|P83731|P62913|P62158|P62753|P01034|Q13765|Q07020|P13639|P04406|O14561|P19021|P37108|Q9Y6H1|Q08431|P84098|Q05639|P63173|P68104|P61353|P46777|P02511|P05386|P60866|P46776|P05387|P60228|P62249|P25398|P68363|P12235|P62891|P42766|Q02878|P84243|P61247|P62857|Q16654|P62979|P62851|P51452|Q14116|Q9P0U1|P08708|O75116|P62906|P18077|P42677|P15880|Q02543|Q9NWT8|Q9Y3D2|Q00325|P62263|P62987|Q14240|P62945|P62269|P25116</td></tr>
<tr><td>GO:0044270</td><td>cellular nitrogen compound catabolic process</td><td>8.72E-35</td><td>6.17E-33</td><td>39</td><td>474</td><td>190</td><td>35980</td><td>P25398|P62280|P39019|P62891|P42766|Q02878|P39023|P62273|P46783|P61247|P83731|P62857|P62913|P62979|P62851|P62753|Q07020|Q08493|P08708|P62906|P18077|P42677|P84098|P15880|P63173|Q02543|P61353|P62263|P62987|Q14240|P62945|P46777|P05386|P60866|P46776|P62269|P05387|P60228|P62249</td></tr>
<tr><td>GO:0044271</td><td>cellular nitrogen compound biosynthetic process</td><td>1.96E-20</td><td>5.03E-19</td><td>78</td><td>4964</td><td>190</td><td>35980</td><td>P0CG47|O75947|Q9NSD9|P01160|P62280|Q8TAK5|P36542|P0CG48|P39019|P16860|P13073|P39023|P62273|P46783|P83731|P62913|P24311|P62753|Q8WYQ3|P20674|Q13765|Q07020|P10606|Q96H40|P13639|P48047|P37108|P16989|Q9Y6H1|P84098|Q05639|P63173|P68104|P61353|P46777|P05386|Q13772|P60866|P46776|P05387|P60228|P62249|P25398|P09669|O00483|P14854|P10176|P12235|P62891|P42766|Q02878|P25705|P18859|Q14192|Q9NPI5|P61247|P62857|P62979|P56381|Q15004|P62851|P54368|P08708|P62906|P18077|P15954|P17535|P42677|P15880|Q02543|Q9NWT8|Q00325|P62263|P62987|Q14240|P62945|P62269|P06576</td></tr>
<tr><td>GO:0044281</td><td>small molecule metabolic process</td><td>2.67E-59</td><td>6.27E-56</td><td>107</td><td>3389</td><td>190</td><td>35980</td><td>O75947|O43674|O96000|P16860|P13073|O14949|P07195|P46783|P83731|P62913|P62753|P24752|O15239|P20674|O95169|P48047|P04406|O14561|P80297|O95167|P84098|P63173|P08574|P47985|P46777|P60866|P46776|P62249|Q13011|Q8N335|P07602|P25398|P10176|O95178|P12235|P62891|P25705|P18859|Q14192|P68871|Q16654|P56381|P54368|O00217|Q08493|P08708|P18077|P12883|P42677|P41222|P15880|P17540|Q02543|P62263|P62945|P62269|P0CG47|P06732|Q9NSD9|P0DMV8|P0DMV9|P01160|P40926|P40925|P62280|P36542|P35613|P0CG48|P39019|P05090|P00505|P39023|P62273|P69905|P62158|P24311|Q8WYQ3|Q07020|P10606|O14880|P19021|O43920|P61353|P05386|P17081|P05387|P13929|P09669|O00483|P14854|P42766|Q02878|Q9NPI5|P17174|P61247|P62857|Q16698|P62979|P62851|P51970|P62906|P15954|P51857|P13533|P62987|P06576|P48735</td></tr>
<tr><td>GO:0044282</td><td>small molecule catabolic process</td><td>1.02E-03</td><td>4.89E-03</td><td>8</td><td>382</td><td>190</td><td>35980</td><td>P13929|P00505|P04406|P17174|Q16698|P51857|P24752|Q13011</td></tr>
<tr><td>GO:0044283</td><td>small molecule biosynthetic process</td><td>1.58E-03</td><td>6.98E-03</td><td>10</td><td>609</td><td>190</td><td>35980</td><td>P13929|P41222|P00505|P40926|P04406|O14561|P40925|P17174|P51857|P24752</td></tr>
<tr><td>GO:0044403</td><td>symbiosis, encompassing mutualism through parasitism</td><td>2.04E-33</td><td>1.26E-31</td><td>46</td><td>830</td><td>190</td><td>35980</td><td>P0CG47|P25398|P0DMV8|P0DMV9|P62280|P12235|P0CG48|P39019|P62891|P42766|P63244|Q02878|P60033|P39023|P62273|P46783|P61247|P83731|P62857|P62913|P62979|P62851|P62753|Q13765|Q07020|P08708|P62906|P18077|Q08431|P42677|P84098|P15880|P63173|Q02543|P61353|P62263|P62987|Q14240|P62945|P46777|P05386|P60866|P46776|P62269|P05387|P62249</td></tr>
<tr><td>GO:0044409</td><td>entry into host</td><td>1.11E-02</td><td>3.31E-02</td><td>3</td><td>87</td><td>190</td><td>35980</td><td>P0DMV8|P0DMV9|P60033</td></tr>
<tr><td>GO:0044419</td><td>interspecies interaction between organisms</td><td>2.04E-33</td><td>1.26E-31</td><td>46</td><td>830</td><td>190</td><td>35980</td><td>P0CG47|P25398|P0DMV8|P0DMV9|P62280|P12235|P0CG48|P39019|P62891|P42766|P63244|Q02878|P60033|P39023|P62273|P46783|P61247|P83731|P62857|P62913|P62979|P62851|P62753|Q13765|Q07020|P08708|P62906|P18077|Q08431|P42677|P84098|P15880|P63173|Q02543|P61353|P62263|P62987|Q14240|P62945|P46777|P05386|P60866|P46776|P62269|P05387|P62249</td></tr>
<tr><td>GO:0044699</td><td>single-organism process</td><td>3.25E-21</td><td>8.72E-20</td><td>174</td><td>22141</td><td>190</td><td>35980</td><td>O75947|P10916|O43674|Q9H3K2|P16615|O96000|P16860|P13073|O14949|P09493|P07195|P46783|P83731|P62913|P62753|P24752|O15239|P20674|Q13765|O95169|P13639|P48047|Q9Y277|P04406|O14561|P80297|Q9Y5N6|P16989|Q9Y6H1|O95167|Q08431|P19105|P84098|O14958|P63173|P09382|P08574|P47985|P46777|Q13772|P60866|P46776|P62249|Q13011|Q8N335|P07602|P52179|P25398|P68363|P10176|O95178|P12235|P13693|P62891|P19429|P25705|P18859|Q14192|P68871|Q16654|P56381|Q01628|P00441|P54368|O00217|Q14116|O15273|Q08493|P08708|Q14515|O75116|P68133|P18077|P17661|Q6P6C2|P17535|P12883|Q8N8D1|P42677|P41222|P15880|P17540|Q02543|Q9NWT8|Q9Y3D2|P62263|Q14240|P62945|P62269|P0CG47|P06732|Q9NSD9|P0DMV8|P0DMV9|P01160|P40926|P40925|P62280|P36542|P35613|P0CG48|P39019|P63244|P05090|Q14094|P00505|P39023|P62273|P24310|P69905|P62158|P45379|P24311|Q8WYQ3|P01034|Q07020|P01033|P10606|Q14896|O14880|P19021|P19022|P14406|P37108|O43920|P68032|P08493|P61353|P60660|P02511|P05386|P17081|P05387|P13929|P02792|P09669|P51684|O00483|P14854|P42766|Q02878|P84243|P60033|Q9NPI5|P17174|P61247|P62857|P23327|P22352|Q16698|P62979|Q15004|P62851|P51452|P02144|Q14315|P51970|Q9P0U1|O75954|P62906|P15954|P51857|P13533|Q00325|P08590|P62987|P04275|P63313|P04792|P25116|P20585|P06576|P63316|P48735</td></tr>
<tr><td>GO:0044706</td><td>multi-multicellular organism process</td><td>1.10E-02</td><td>3.29E-02</td><td>5</td><td>251</td><td>190</td><td>35980</td><td>P01160|P19021|P35613|P00441|P01034</td></tr>
<tr><td>GO:0044707</td><td>single-multicellular organism process</td><td>1.97E-08</td><td>2.37E-07</td><td>64</td><td>6139</td><td>190</td><td>35980</td><td>P0CG47|P10916|P01160|P16615|P62280|P35613|P0CG48|P39019|P63244|P05090|O14949|P09493|P83731|P62158|P45379|P24311|P24752|P01034|Q13765|P01033|P10606|Q14896|P13639|P19021|P19022|P16989|P68032|Q08431|P19105|O14958|P63173|P08493|P60660|P09382|P02511|Q13772|P07602|P14854|P19429|P25705|P84243|P18859|Q14192|P68871|P62979|P00441|P02144|Q14116|O15273|O75116|P68133|P51857|P13533|P17535|P12883|P08590|P62263|P62987|P04275|P04792|P25116|P20585|P06576|P63316</td></tr>
</table>

GO:0044710	single-organism metabolic process	2.89E-35	2.15E-33	122	8018	190	35980	O75947|O43674|O96000|P16860|P13073|O14949|P07195|P46783|P83731|P62913|P62753|P24752|O15239|P20674|O95169|P13639|P48047|P04406|O14561|P80297|O95167|P84098|P63173|P08574|P47985|P46777|P60866|P46776|P62249|Q13011|Q8N335|P07602|P52179|P25398|P10176|O95178|P12235|P62891|P25705|P18859|Q14192|P68871|Q16654|P56381|P00441|P54368|O00217|Q14116|Q08493|P08708|P18077|Q6P6C2|P12883|P42677|P41222|P15880|P17540|Q02543|Q9NWT8|Q9Y3D2|P62263|P62945|P62269|P0CG47|P06732|Q9NSD9|P0DMV8|P0DMV9|P01160|P40926|P40925|P62280|P36542|P35613|P0CG48|P39019|P05090|P00505|P39023|P62273|P69905|P62158|P24311|Q8WYQ3|Q07020|P10606|O14880|P19021|O43920|P61353|P02511|P05386|P17081|P05387|P13929|P09669|O00483|P14854|P42766|Q02878|P60033|Q9NPI5|P17174|P61247|P62857|P22352|Q16698|P62979|Q15004|P62851|P51452|P51970|Q9P0U1|P62906|P15954|P51857|P13533|P62987|P04275|P20585|P06576|P48735
GO:0044711	single-organism biosynthetic process	1.32E-07	1.42E-06	30	1946	190	35980	P0CG47|P13929|O75947|P01160|P40926|P40925|P36542|P0CG48|P16860|P25705|P00505|P18859|Q9NPI5|P17174|P62979|P56381|Q15004|Q8WYQ3|P24752|P13639|P48047|P04406|O14561|O14880|P51857|P41222|Q9NWT8|P62987|Q8N335|P06576
GO:0044712	single-organism catabolic process	2.34E-06	2.17E-05	21	1224	190	35980	P0CG47|P52179|P13929|Q08493|Q9P0U1|P04406|P40925|P51857|P10176|P0CG48|P00505|P68871|P17174|P62987|P22352|Q16698|P62979|P69905|P62158|P24752|Q13011
GO:0044723	single-organism carbohydrate metabolic process	1.26E-03	5.88E-03	14	1034	190	35980	P0CG47|P13929|P40926|P04406|P40925|P0CG48|P05090|P00505|P17174|P62987|Q16654|P62979|P62158|P48735
GO:0044763	single-organism cellular process	2.58E-25	1.04E-23	168	19209	190	35980	O75947|P10916|O43674|Q9H3K2|P16615|O96000|P16860|P13073|O14949|P09493|P07195|P46783|P83731|P62913|P62753|P24752|O15239|P20674|O95169|P13639|P48047|Q9Y277|P04406|O14561|Q9Y5N6|O95167|Q08431|P19105|P84098|O14958|P63173|P09382|P08574|P47985|P46777|Q13772|P60866|P46776|P62249|Q13011|Q8N335|P07602|P52179|P25398|P68363|P10176|O95178|P12235|P13693|P62891|P19429|P25705|P18859|Q14192|P68871|Q16654|P56381|Q01628|P00441|O00217|Q14116|O15273|Q08493|P08708|Q14515|O75116|P68133|P18077|P17661|Q6P6C2|P17535|P12883|Q8N8D1|P42677|P41222|P15880|P17540|Q02543|Q9NWT8|Q9Y3D2|P62263|Q14240|P62945|P62269|P0CG47|P06732|Q9NSD9|P0DMV8|P0DMV9|P01160|P40926|P40925|P62280|P36542|P35613|P0CG48|P39019|P63244|P05090|P00505|P39023|P62273|P24310|P69905|P62158|P45379|P24311|Q8WYQ3|P01034|Q07020|P01033|P10606|Q14896|O14880|P19021|P19022|P14406|P37108|O43920|P68032|P08493|P61353|P60660|P02511|P05386|P17081|P05387|P13929|P02792|P09669|P51684|O00483|P14854|P42766|Q02878|P84243|P60033|Q9NPI5|P17174|P61247|P62857|P23327|P22352|Q16698|P62979|Q15004|P62851|P51452|P02144|Q14315|P51970|Q9P0U1|O75954|P62906|P15954|P51857|P13533|Q00325|P08590|P62987|P04275|P63313|P04792|P25116|P20585|P06576|P63316|P48735
GO:0044764	multi-organism cellular process	1.95E-34	1.29E-32	46	787	190	35980	P0CG47|P25398|P0DMV8|P0DMV9|P62280|P12235|P0CG48|P39019|P62891|P42766|P63244|Q02878|P60033|P39023|P62273|P46783|P61247|P83731|P62857|P62913|P62979|P62851|P62753|Q13765|Q07020|P08708|P62906|P18077|Q08431|P42677|P84098|P15880|P63173|Q02543|P61353|P62263|P62987|Q14240|P62945|P46777|P05386|P60866|P46776|P62269|P05387|P62249
GO:0044765	single-organism transport	7.96E-25	3.00E-23	87	5265	190	35980	P0CG47|O75947|P0DMV8|P16615|P62280|P36542|P35613|P0CG48|P39019|P16860|P05090|P13073|P00505|O14949|P39023|P62273|P46783|P83731|P62913|P24310|P69905|P62158|P24311|P62753|P20674|Q07020|P01033|P10606|P48047|Q9Y277|P19021|P14406|P37108|Q9Y6H1|Q08431|P84098|O14958|P63173|P61353|P47985|P46777|P05386|P60866|P46776|P05387|P62249|P07602|P02792|P25398|P09669|P68363|O00483|P14854|P10176|P12235|P13693|P62891|P42766|Q02878|P25705|P18859|P60033|P68871|P61247|P62857|P62979|P56381|P62851|P00441|P02144|Q9P0U1|P08708|P62906|P18077|P15954|Q6P6C2|P42677|P15880|Q02543|Q00325|P62263|P62987|P62945|P04275|P62269|P25116|P06576
GO:0044766	multi-organism transport	3.89E-05	2.81E-04	4	36	190	35980	P0CG47|P62987|P62979|P0CG48
GO:0044767	single-organism developmental process	2.08E-08	2.47E-07	63	6002	190	35980	P0CG47|P10916|P01160|P16615|P62280|P35613|P0CG48|P39019|P63244|P05090|O14949|P09493|P83731|P62158|P45379|P24311|P24752|P01034|Q13765|P01033|Q14896|P13639|P19021|P19022|P16989|P68032|Q08431|P19105|O14958|P63173|P08493|P60660|P09382|P02511|Q13772|P07602|P13929|P14854|P19429|P25705|P84243|P18859|Q14192|P61247|P62979|P00441|P02144|Q14315|Q14116|O15273|O75116|P68133|Q6P6C2|P13533|P17535|P12883|P08590|P62263|P62987|P04275|P20585|P06576|P63316
GO:0044770	cell cycle phase transition	6.77E-03	2.28E-02	6	315	190	35980	P0CG47|P62987|Q9Y5N6|P83731|P62979|P0CG48
GO:0044772	mitotic cell cycle phase transition	6.38E-03	2.17E-02	6	311	190	35980	P0CG47|P62987|Q9Y5N6|P83731|P62979|P0CG48
GO:0044773	mitotic DNA damage checkpoint	2.22E-03	9.26E-03	4	103	190	35980	P0CG47|P62987|P62979|P0CG48
GO:0044774	mitotic DNA integrity checkpoint	2.81E-03	1.12E-02	4	110	190	35980	P0CG47|P62987|P62979|P0CG48
GO:0044783	G1 DNA damage checkpoint	8.31E-04	4.08E-03	4	79	190	35980	P0CG47|P62987|P62979|P0CG48
GO:0044802	single-organism membrane organization	1.46E-28	7.10E-27	44	957	190	35980	P25398|P0DMV8|P16615|P62280|P35613|P39019|P62891|P42766|Q02878|P39023|P62273|P46783|P61247|P83731|P62857|P62913|P62979|P62851|P62753|P00441|Q07020|Q9P0U1|P08708|O75116|P62906|P19022|P18077|P37108|P42677|P84098|P15880|P63173|Q02543|P61353|P62263|P62987|P62945|P46777|P05386|P60866|P46776|P62269|P05387|P62249
GO:0044819	mitotic G1/S transition checkpoint	8.31E-04	4.08E-03	4	79	190	35980	P0CG47|P62987|P62979|P0CG48
GO:0044839	cell cycle G2/M phase transition	6.95E-03	2.33E-02	4	142	190	35980	P0CG47|P62987|P62979|P0CG48
GO:0044843	cell cycle G1/S phase transition	2.50E-03	1.02E-02	5	176	190	35980	P0CG47|P62987|Q9Y5N6|P62979|P0CG48
GO:0045047	protein targeting to ER	1.33E-54	4.39E-52	37	127	190	35980	P25398|P62280|P39019|P62891|P42766|Q02878|P39023|P62273|P46783|P61247|P83731|P62857|P62913|P62979|P62851|P62753|Q07020|P08708|P62906|P18077|P37108|P42677|P84098|P15880|P63173|Q02543|P61353|P62263|P62987|P62945|P46777|P05386|P60866|P46776|P62269|P05387|P62249
GO:0045088	regulation of innate immune response	5.02E-04	2.62E-03	9	430	190	35980	P0CG47|P51452|P0DMV8|P0DMV9|P62987|P62979|P62158|P0CG48|P39019
GO:0045089	positive regulation of innate immune response	1.34E-03	6.17E-03	7	309	190	35980	P0CG47|P51452|P62987|P62979|P62158|P0CG48|P39019
GO:0045137	development of primary sexual characteristics	8.55E-03	2.76E-02	5	236	190	35980	P0CG47|P16989|Q13772|P00441|P01034
GO:0045184	establishment of protein localization	4.30E-17	9.13E-16	45	1970	190	35980	P25398|Q969T9|P62280|P35613|P39019|P62891|P42766|Q02878|P39023|P62273|P46783|P61247|P83731|P62857|P62913|P62979|P62851|P62753|Q13765|Q07020|Q9P0U1|P08708|O75116|P62906|P19022|P18077|P37108|Q9Y6H1|P42677|P84098|P15880|P63173|Q02543|P61353|P62263|P62987|P62945|P46777|P05386|P60866|P46776|P62269|P05387|P25116|P62249
GO:0045214	sarcomere organization	3.42E-08	3.96E-07	6	36	190	35980	O15273|Q14896|O14958|P09493|P45379|P13533
GO:0045333	cellular respiration	6.27E-36	5.04E-34	32	231	190	35980	O75947|P09669|O43674|P40926|P40925|O00483|P14854|P36542|P10176|O95178|O96000|P13073|P25705|P18859|O14949|P56381|P24311|O15239|P20674|O00217|P10606|O95169|P51970|P48047|O14561|O43920|P15954|O95167|P08574|P47985|P06576|P48735
GO:0045471	response to ethanol	6.95E-03	2.33E-02	4	142	190	35980	P13639|P00505|P68032|P00441
GO:0045732	positive regulation of protein catabolic process	4.90E-04	2.57E-03	7	260	190	35980	P0CG47|P54368|P63244|P60033|P62987|P62979|P0CG48
GO:0045787	positive regulation of cell cycle	1.40E-02	4.02E-02	6	369	190	35980	P0CG47|P51452|O75116|P62987|P62979|P0CG48
GO:0045823	positive regulation of heart contraction	3.89E-05	2.81E-04	4	36	190	35980	P01160|P09493|P16615|P23327
GO:0045859	regulation of protein kinase activity	1.29E-02	3.79E-02	11	954	190	35980	P0CG47|P51452|P63244|P60033|P62987|P62979|P62158|P04792|P25116|P00441|P0CG48
GO:0045862	positive regulation of proteolysis	3.72E-03	1.43E-02	7	371	190	35980	P0CG47|P63244|Q9NWT8|P62987|P62979|P25116|P0CG48
GO:0045893	positive regulation of transcription, DNA-templated	9.65E-03	3.05E-02	16	1575	190	35980	P0CG47|Q13765|P0DMV8|P0DMV9|Q969T9|Q8TAK5|Q9Y6H1|P17535|P0CG48|P60033|Q14192|P62987|P62979|Q13772|P17081|P25116
GO:0045926	negative regulation of growth	2.04E-03	8.66E-03	6	246	190	35980	P63244|P16860|P10916|P01160|P80297|P02511
GO:0045930	negative regulation of mitotic cell cycle	3.17E-03	1.25E-02	6	269	190	35980	P0CG47|Q9NWT8|P62987|P83731|P62979|P0CG48
GO:0045935	positive regulation of nucleobase-containing compound metabolic process	4.94E-03	1.83E-02	19	1870	190	35980	P0CG47|Q13765|P0DMV8|P0DMV9|Q969T9|Q8TAK5|Q9Y6H1|P17535|P0CG48|P63244|P60033|Q14192|P62987|P62979|Q13772|P62158|P17081|P25116|P01034
GO:0045937	positive regulation of phosphate metabolic process	5.72E-03	1.98E-02	14	1224	190	35980	P07602|P0CG47|Q14116|O75116|P19022|P0CG48|P63244|Q05639|P60033|P62987|P62979|P62158|P25116|P00441
GO:0046034	ATP metabolic process	3.65E-26	1.55E-24	28	311	190	35980	P13929|O75947|P0DMV8|P0DMV9|O43674|O00483|P36542|P10176|O95178|O96000|P13073|P25705|P18859|P56381|Q8WYQ3|O15239|P20674|O00217|O95169|P51970|P48047|P04406|O14561|O43920|P13533|O95167|P12883|P06576
GO:0046128	purine ribonucleoside metabolic process	1.82E-22	5.20E-21	29	468	190	35980	P13929|O75947|P0DMV8|P0DMV9|O43674|O00483|P36542|P10176|O95178|O96000|P13073|P25705|P18859|P56381|Q8WYQ3|O15239|P20674|O00217|O95169|P51970|P48047|P04406|O14561|O43920|P13533|O95167|P12883|P17081|P06576
GO:0046129	purine ribonucleoside biosynthetic process	1.45E-06	1.38E-05	8	150	190	35980	O75947|P25705|P48047|P18859|P36542|P56381|Q8WYQ3|P06576
GO:0046364	monosaccharide biosynthetic process	5.79E-06	5.02E-05	6	84	190	35980	P13929|P00505|P40926|P04406|P40925|P17174

GO:0046390	ribose phosphate biosynthetic process	3.73E-07	3.78E-06	10	225	190	35980	O75947|P16860|P25705|P48047|P18859|P01160|P36542|P56381|Q8WYQ3|P06576
GO:0046395	carboxylic acid catabolic process	2.39E-03	9.85E-03	6	254	190	35980	P00505|P17174|Q16698|P51857|P24752|Q13011
GO:0046483	heterocycle metabolic process	2.66E-24	9.40E-23	99	6901	190	35980	O75947|O43674|O96000|P16860|P13073|P07195|P46783|P83731|P62913|P62753|O15239|P20674|Q13765|O95169|P48047|P04406|O14561|Q9Y5N6|P16989|Q9Y6H1|O95167|P84098|P63173|P68104|P46777|Q13772|P60866|P46776|P60228|P62249|Q8N335|P25398|P10176|O95178|P62891|P25705|P18859|Q14192|P56381|O00217|Q08493|P08708|P18077|Q6P6C2|P17535|P12883|Q8N8D1|P42677|P15880|Q02543|P62263|Q14240|P62945|P62269|P0CG47|Q9NSD9|P0DMV8|P0DMV9|P01160|P40926|P40925|P62280|Q8TAK5|P36542|P0CG48|P39019|P00505|P39023|P62273|P24311|Q8WYQ3|Q07020|P10606|Q96H40|O43920|P61353|P05386|P17081|P05387|P13929|P09669|O00483|P14854|P42766|Q02878|P84243|Q9NPI5|P61247|P62857|P62979|Q15004|P62851|P51970|P62906|P15954|P13533|P62987|P20585|P06576
GO:0046496	nicotinamide nucleotide metabolic process	2.23E-04	1.30E-03	7	228	190	35980	P13929|P40926|P04406|Q9NPI5|P07195|P40925|Q8N335
GO:0046546	development of primary male sexual characteristics	6.29E-03	2.14E-02	4	138	190	35980	P0CG47|P16989|Q13772|P01034
GO:0046620	regulation of organ growth	1.01E-02	3.17E-02	3	84	190	35980	P16989|P13533|P00441
GO:0046661	male sex differentiation	1.11E-02	3.32E-02	4	163	190	35980	P0CG47|P16989|Q13772|P01034
GO:0046700	heterocycle catabolic process	6.67E-36	5.22E-34	40	480	190	35980	P25398|P62280|P39019|P62891|P42766|Q02878|P00505|P39023|P62273|P46783|P61247|P83731|P62857|P62913|P62979|P62851|P62753|Q07020|Q08493|P08708|P62906|P18077|P42677|P84098|P15880|P63173|Q02543|P61353|P62263|P62987|Q14240|P62945|P46777|P05386|P60866|P46776|P62269|P05387|P60228|P62249
GO:0046718	viral entry into host cell	1.08E-02	3.25E-02	3	86	190	35980	P0DMV8|P0DMV9|P60033
GO:0046794	transport of virus	3.47E-05	2.57E-04	4	35	190	35980	P0CG47|P62987|P62979|P0CG48
GO:0046907	intracellular transport	4.79E-25	1.83E-23	56	2060	190	35980	P0CG47|O75947|P0DMV8|P16615|P62280|P36542|P35613|P0CG48|P39019|P39023|P62273|P46783|P83731|P62913|P62753|Q07020|P48047|P37108|Q9Y6H1|P84098|P63173|P61353|P46777|P05386|P60866|P46776|P05387|P62249|P02792|P25398|P68363|P62891|P42766|Q02878|P25705|P18859|P61247|P62857|P62979|P56381|P62851|P00441|Q9P0U1|P08708|P62906|P18077|Q6P6C2|P42677|P15880|Q02543|P62263|P62987|P62945|P62269|P25116|P06576
GO:0048010	vascular endothelial growth factor receptor signaling	9.88E-04	4.75E-03	7	293	190	35980	P0CG47|O75116|P62987|P62979|P62158|P04792|P0CG48
GO:0048468	cell development	9.22E-07	9.01E-06	28	1903	190	35980	P0CG47|P10916|P01160|P16615|P0CG48|P05090|O14949|Q14192|P09493|P83731|P62979|P62158|P45379|P00441|P01034|Q14315|O15273|Q14896|O75116|P68133|P68032|P13533|P17535|P19105|O14958|P60660|P62987|P02511
GO:0048511	rhythmic process	1.02E-05	8.41E-05	10	326	190	35980	P0CG47|P63244|O75116|P19021|P62987|P62979|P17535|P00441|P0CG48|P01034
GO:0048513	animal organ development	2.68E-09	3.60E-08	43	3073	190	35980	P07602|P0CG47|P10916|P01160|P14854|P35613|P39019|P05090|P19429|P84243|P18859|O14949|Q14192|P09493|P83731|P62158|P45379|P24752|P00441|P01034|Q13765|P01033|P02144|O15273|Q14896|P13639|P19021|P19022|P68133|P16989|P68032|P13533|P12883|P63173|P08493|P60660|P09382|P08590|P62263|P02511|P04275|Q13772|P63316
GO:0048518	positive regulation of biological process	1.67E-04	1.01E-03	56	6674	190	35980	P0CG47|P0DMV8|P0DMV9|Q969T9|P01160|P16615|Q8TAK5|P0CG48|P39019|P63244|P16860|P09493|P69905|P62158|P45379|P62753|P01034|Q13765|P01033|Q14896|P13639|P19022|P16989|Q9Y6H1|P68032|Q08431|O14958|Q05639|P09382|Q13772|P17081|Q8N335|P07602|P51684|P84243|P60033|Q14192|P68871|P23327|P62979|P00441|P51452|P54368|Q14116|Q9P0U1|O75116|P68133|P17535|Q8N8D1|P15880|Q9NWT8|P08590|P62987|P04792|P25116|P20585
GO:0048519	negative regulation of biological process	6.46E-09	8.28E-08	59	5261	190	35980	P0CG47|P10916|P0DMV8|P0DMV9|P01160|P16615|P0CG48|P39019|P63244|P16860|P05090|P09493|P83731|P62158|P45379|Q8WYQ3|P01034|Q13765|P01033|P04406|P80297|P19022|P16989|P68032|O14958|P09382|P02511|P17081|P60228|Q8N335|P07602|Q8N3K9|P12235|P13693|P19429|P25705|P84243|Q14192|Q9NPI5|P61247|Q16654|P62979|Q01628|P00441|P51452|P54368|Q14116|Q08493|O75116|P17535|P41222|Q9NWT8|P62263|P62987|Q14240|P04792|P25116|P20585|P06576
GO:0048522	positive regulation of cellular process	7.64E-04	3.82E-03	45	5319	190	35980	P07602|P0CG47|P51684|P0DMV8|P0DMV9|Q969T9|P01160|Q8TAK5|P0CG48|P39019|P63244|P84243|P60033|Q14192|P68871|P09493|P62979|P69905|P62158|P62753|P00441|P01034|P51452|Q13765|P01033|Q14116|P13639|Q9P0U1|O75116|P19022|P16989|Q9Y6H1|Q08431|P17535|Q8N8D1|O14958|Q05639|Q9NWT8|P09382|P62987|Q13772|P17081|P04792|P25116|Q8N335
GO:0048523	negative regulation of cellular process	7.72E-08	8.36E-07	53	4782	190	35980	P0CG47|P10916|P0DMV8|P0DMV9|P01160|P0CG48|P63244|P16860|P05090|P09493|P83731|P62158|P01034|Q13765|P01033|P04406|P19022|P16989|P68032|O14958|P09382|P02511|P17081|P60228|Q8N335|P07602|Q8N3K9|P12235|P13693|P25705|P84243|Q14192|Q9NPI5|P61247|Q16654|P62979|Q01628|P00441|P51452|P54368|Q14116|Q08493|O75116|P17535|P41222|Q9NWT8|P62263|P62987|Q14240|P04792|P25116|P20585|P06576
GO:0048545	response to steroid hormone	2.99E-06	2.71E-05	14	582	190	35980	P13639|Q969T9|O75116|P19021|P68133|Q08431|Q8N8D1|P41222|Q14192|P17174|P02511|Q13772|P62158|P01034
GO:0048583	regulation of response to stimulus	2.67E-04	1.52E-03	41	4465	190	35980	P07602|P0CG47|P52179|Q6GTX8|P51684|P0DMV8|P0DMV9|P40925|P16615|Q8N3K9|P10176|P0CG48|P39019|P13693|P63244|P05090|P60033|Q16654|P23327|P62979|P62158|P00441|P51452|P01033|Q14116|P19022|P16989|Q9Y6H1|Q6P6C2|P12883|Q8N8D1|P41222|O14958|P09382|P62987|P02511|P17081|P04792|P25116|Q8N335|P63316
GO:0048585	negative regulation of response to stimulus	4.34E-04	2.34E-03	19	1510	190	35980	P07602|P0CG47|P51452|P0DMV8|P0DMV9|P19022|P16989|Q8N3K9|P0CG48|P39019|P13693|P63244|P05090|O14958|P62987|P62979|P62158|P04792|Q8N335
GO:0048608	reproductive structure development	2.61E-03	1.06E-02	8	444	190	35980	P07602|P0CG47|P16989|P04275|Q13772|P35613|P00441|P01034
GO:0048644	muscle organ morphogenesis	2.83E-13	4.94E-12	11	76	190	35980	O15273|Q14896|P10916|P19429|P08590|P09493|P68032|P45379|P13533|P12883|P63316
GO:0048646	anatomical structure formation involved in morphogenesis	2.47E-03	1.01E-02	14	1112	190	35980	Q14116|O15273|Q14896|P10916|P68133|P68032|P13533|Q08431|P05090|O14958|Q14192|P09493|P45379|P06576
GO:0048666	neuron development	1.55E-02	4.33E-02	12	1112	190	35980	P0CG47|P05090|O14949|P60660|O75116|P62987|P83731|P62979|P62158|P19105|P00441|P0CG48
GO:0048667	cell morphogenesis involved in neuron differentiation	7.06E-03	2.36E-02	10	754	190	35980	P0CG47|P60660|O75116|P62987|P83731|P62979|P62158|P19105|P00441|P0CG48
GO:0048678	response to axon injury	2.08E-04	1.23E-03	4	55	190	35980	P05090|P09382|P00441|P01034
GO:0048729	tissue morphogenesis	1.17E-04	7.37E-04	12	616	190	35980	O15273|Q14896|P10916|P19429|P08590|P09493|P68133|P68032|P45379|P13533|P12883|P63316
GO:0048731	system development	1.06E-08	1.33E-07	53	4508	190	35980	P0CG47|P10916|P01160|P35613|P0CG48|P39019|P05090|O14949|P09493|P83731|P62158|P45379|P24311|P24752|P01034|Q13765|P01033|Q14896|P13639|P19021|P19022|P16989|P68032|Q08431|P19105|P63173|P08493|P60660|P09382|P02511|Q13772|P07602|P14854|P19429|P84243|P18859|Q14192|P62979|P00441|P02144|Q14116|O15273|O75116|P68133|P13533|P12883|P08590|P62263|P62987|P04275|P20585|P06576|P63316
GO:0048738	cardiac muscle tissue development	5.01E-12	8.07E-11	13	164	190	35980	O15273|Q14896|P10916|P01160|P68032|P13533|P12883|P19429|Q14192|P08590|P09493|P45379|P63316
GO:0048747	muscle fiber development	6.81E-06	5.80E-05	5	50	190	35980	Q14315|O15273|P10916|P68133|P13533
GO:0048856	anatomical structure development	1.21E-09	1.65E-08	61	5308	190	35980	P0CG47|P10916|P01160|P16615|P35613|P0CG48|P39019|P63244|P05090|O14949|P09493|P83731|P62158|P45379|P24311|P24752|P01034|Q13765|P01033|Q14896|P13639|P19021|P19022|P16989|P68032|Q08431|P19105|O14958|P63173|P08493|P60660|P09382|P02511|Q13772|P07602|P13929|P14854|P19429|P25705|P84243|P18859|Q14192|P62979|P00441|P02144|Q14315|Q14116|O15273|O75116|P68133|P13533|P17535|P12883|P08590|P62263|P62987|P04275|P25116|P20585|P06576|P63316
GO:0048857	neural nucleus development	5.88E-03	2.02E-02	3	69	190	35980	P18859|P14854|P62158
GO:0048869	cellular developmental process	7.14E-06	6.01E-05	42	3946	190	35980	P07602|P0CG47|P10916|P01160|P16615|P62280|P0CG48|P39019|P63244|P05090|P19429|O14949|Q14192|P09493|P61247|P83731|P62979|P62158|P45379|P00441|P01034|P02144|Q14315|O15273|Q14896|P13639|O75116|P19022|P68133|P68032|Q6P6C2|P13533|P17535|P19105|O14958|P08493|P60660|P09382|P62263|P62987|P02511|P06576
GO:0048872	homeostasis of number of cells	1.16E-04	7.31E-04	7	205	190	35980	P02144|P08708|P62263|P19022|P25116|P00441|P39019
GO:0048878	chemical homeostasis	2.40E-04	1.38E-03	15	980	190	35980	P02792|P51684|P16615|P13693|P63244|O14958|P19429|P09382|P17174|Q16654|P23327|P62753|P25116|P00441|P06576
GO:0050688	regulation of defense response to virus	4.98E-04	2.61E-03	6	187	190	35980	P52179|P40925|P62987|Q16654|Q6P6C2|P10176
GO:0050691	regulation of defense response to virus by host	6.10E-05	4.20E-04	6	127	190	35980	P52179|P40925|P62987|Q16654|Q6P6C2|P10176

<table>
<tr><td>GO:0050789</td><td>regulation of biological process</td><td>1.24E-02</td><td>3.66E-02</td><td>108</td><td>17440</td><td>190</td><td>35980</td><td>P10916|Q969T9|P16615|P16860|P09493|P83731|P62753|Q13765|P13639|Q9Y277|P04406|P80297|P16989|Q9Y6H1|Q08431|P19105|O14958|P63173|P68104|P09382|Q13772|P60228|O14558|Q8N335|P07602|P52179|Q6GTX8|Q8N3K9|P10176|P12235|P13693|P19429|P25705|Q14192|P68871|Q16654|Q01628|P00441|P54368|Q14116|Q08493|Q14515|O75116|P68133|P17661|Q6P6C2|P17535|P12883|Q8N8D1|P42677|P41222|P15880|Q9NWT8|Q9Y3D2|P62263|Q14240|P0CG47|P0DMV8|P0DMV9|P01160|P40925|Q8TAK5|P35613|P0CG48|P39019|P63244|P05090|Q14094|Q02818|P69905|P62158|P45379|Q8WYQ3|P01034|P01033|Q14896|Q96H40|O14880|P19021|P19022|P68032|Q05639|P08493|P60660|P02511|P17081|P51684|Q02878|P84243|P60033|Q9UBY9|Q9NPI5|P17174|P61247|P23327|P62979|Q15004|P51452|Q9P0U1|O75954|P13533|P08590|P62987|P04792|P25116|P20585|P06576|P63316</td></tr>
<tr><td>GO:0050790</td><td>regulation of catalytic activity</td><td>7.58E-04</td><td>3.80E-03</td><td>31</td><td>3212</td><td>190</td><td>35980</td><td>P07602|P0CG47|P0DMV8|Q8N3K9|P0CG48|P63244|P19429|P60033|P09493|P62979|P62158|P45379|Q8WYQ3|P00441|P01034|P51452|P54368|P01033|Q14896|O75116|P13533|P15880|Q05639|P08590|P62987|Q14240|P02511|P04792|P25116|P20585|P63316</td></tr>
<tr><td>GO:0050801</td><td>ion homeostasis</td><td>1.79E-04</td><td>1.07E-03</td><td>12</td><td>645</td><td>190</td><td>35980</td><td>P13693|P02792|P51684|O14958|P19429|P17174|P16615|Q16654|P23327|P25116|P00441|P06576</td></tr>
<tr><td>GO:0050817</td><td>coagulation</td><td>3.54E-05</td><td>2.62E-04</td><td>12</td><td>543</td><td>190</td><td>35980</td><td>P07602|P01033|P84243|P68871|P16615|P04275|P62158|P04792|P35613|P19105|P25116|P00441</td></tr>
<tr><td>GO:0050848</td><td>regulation of calcium-mediated signaling</td><td>9.13E-04</td><td>4.44E-03</td><td>4</td><td>81</td><td>190</td><td>35980</td><td>O14958|P23327|Q8N3K9|P62158</td></tr>
<tr><td>GO:0050849</td><td>negative regulation of calcium-mediated signaling</td><td>1.32E-04</td><td>8.21E-04</td><td>3</td><td>19</td><td>190</td><td>35980</td><td>O14958|Q8N3K9|P62158</td></tr>
<tr><td>GO:0050852</td><td>T cell receptor signaling pathway</td><td>1.52E-02</td><td>4.33E-02</td><td>4</td><td>179</td><td>190</td><td>35980</td><td>P0CG47|P62987|P62979|P0CG48</td></tr>
<tr><td>GO:0050878</td><td>regulation of body fluid levels</td><td>4.33E-05</td><td>3.10E-04</td><td>14</td><td>740</td><td>190</td><td>35980</td><td>P07602|P01033|P19021|P16615|P35613|P19105|P16860|P84243|P68871|P04275|P62158|P04792|P25116|P00441</td></tr>
<tr><td>GO:0050879</td><td>multicellular organismal movement</td><td>7.72E-08</td><td>8.36E-07</td><td>6</td><td>41</td><td>190</td><td>35980</td><td>P02144|O15273|P19429|P13639|P12883|P63316</td></tr>
<tr><td>GO:0050880</td><td>regulation of blood vessel size</td><td>1.94E-03</td><td>8.31E-03</td><td>5</td><td>166</td><td>190</td><td>35980</td><td>P16860|P01160|P68871|P25116|P00441</td></tr>
<tr><td>GO:0050881</td><td>musculoskeletal movement</td><td>7.72E-08</td><td>8.36E-07</td><td>6</td><td>41</td><td>190</td><td>35980</td><td>P02144|O15273|P19429|P13639|P12883|P63316</td></tr>
<tr><td>GO:0050896</td><td>response to stimulus</td><td>5.92E-08</td><td>6.60E-07</td><td>96</td><td>11437</td><td>190</td><td>35980</td><td>P0CG47|P0DMV8|P0DMV9|Q12988|Q969T9|P01160|P40925|P16615|P35613|P0CG48|P39019|P63244|P16860|P05090|P13073|P00505|P39023|P09493|Q02818|P83731|P69905|P62158|P45379|P62753|P24752|P01034|Q13765|P01033|P13639|P04406|O14880|P80297|P19021|P37108|P16989|P68032|Q08431|P19105|O14958|Q05639|P68104|P60660|P09382|P08574|P47985|P02511|Q13772|P17081|P07602|P52179|P13929|Q6GTX8|P51684|P68363|Q6PI73|P10176|P13693|P62891|P84243|P60033|Q14192|P68871|Q9UBY9|P17174|Q16654|P23327|P22352|P62979|Q15004|Q01628|P00441|P51452|O00217|P02144|Q14116|O15273|Q08493|Q9P0U1|O75954|Q14515|O75116|P68133|Q6P6C2|P13533|P17535|Q8N8D1|P42677|P41222|Q9Y3D2|P62987|Q14240|P04275|P04792|P25116|P20585|P63316</td></tr>
<tr><td>GO:0051049</td><td>regulation of transport</td><td>5.36E-05</td><td>3.72E-04</td><td>25</td><td>1991</td><td>190</td><td>35980</td><td>P0DMV8|P01160|P16615|P12235|P63244|P16860|P05090|Q02818|P23327|P62158|P45379|P54368|Q14116|Q08493|Q14896|Q9P0U1|Q9Y277|P19021|Q08431|O14958|P02511|P17081|P25116|Q8N335|P63316</td></tr>
<tr><td>GO:0051050</td><td>positive regulation of transport</td><td>1.31E-02</td><td>3.83E-02</td><td>11</td><td>956</td><td>190</td><td>35980</td><td>P54368|Q14116|P63244|P16860|Q9P0U1|P01160|P62158|P17081|Q08431|P25116|Q8N335</td></tr>
<tr><td>GO:0051051</td><td>negative regulation of transport</td><td>1.15E-03</td><td>5.45E-03</td><td>9</td><td>484</td><td>190</td><td>35980</td><td>P54368|P63244|Q08493|P05090|O14958|P0DMV8|P02511|P62158|P25116</td></tr>
<tr><td>GO:0051091</td><td>positive regulation of sequence-specific DNA binding transcription factor activity</td><td>2.49E-03</td><td>1.02E-02</td><td>6</td><td>256</td><td>190</td><td>35980</td><td>P0CG47|P0DMV8|P0DMV9|P62987|P62979|P0CG48</td></tr>
<tr><td>GO:0051092</td><td>positive regulation of NF-kappaB transcription factor</td><td>1.58E-04</td><td>9.68E-04</td><td>6</td><td>151</td><td>190</td><td>35980</td><td>P0CG47|P0DMV8|P0DMV9|P62987|P62979|P0CG48</td></tr>
<tr><td>GO:0051129</td><td>negative regulation of cellular component organization</td><td>1.37E-02</td><td>3.97E-02</td><td>9</td><td>711</td><td>190</td><td>35980</td><td>P63244|P05090|P0DMV8|P0DMV9|Q9NWT8|P09382|O75116|P17081|P01034</td></tr>
<tr><td>GO:0051146</td><td>striated muscle cell differentiation</td><td>1.19E-10</td><td>1.74E-09</td><td>13</td><td>211</td><td>190</td><td>35980</td><td>Q14315|O15273|Q14896|P10916|P01160|P19022|P68133|P68032|P13533|O14958|Q14192|P09493|P45379</td></tr>
<tr><td>GO:0051147</td><td>regulation of muscle cell differentiation</td><td>1.16E-02</td><td>3.45E-02</td><td>4</td><td>165</td><td>190</td><td>35980</td><td>Q13765|Q14116|Q9NPI5|P19022</td></tr>
<tr><td>GO:0051173</td><td>positive regulation of nitrogen compound metabolic process</td><td>7.49E-04</td><td>3.76E-03</td><td>22</td><td>1967</td><td>190</td><td>35980</td><td>P0CG47|Q13765|P0DMV8|P13639|P0DMV9|Q969T9|P16989|Q8TAK5|Q9Y6H1|P17535|P0CG48|P63244|P60033|Q14192|P68871|P62987|P62979|Q13772|P62158|P17081|P25116|P01034</td></tr>
<tr><td>GO:0051174</td><td>regulation of phosphorus metabolic process</td><td>2.94E-03</td><td>1.16E-02</td><td>20</td><td>1917</td><td>190</td><td>35980</td><td>P07602|P0CG47|P51452|Q14116|O75116|P19022|Q8N3K9|P0CG48|P63244|Q05639|P60033|P62987|Q16654|P23327|P62979|P62158|P04792|P25116|Q8N335|P00441</td></tr>
<tr><td>GO:0051179</td><td>localization</td><td>3.78E-16</td><td>7.65E-15</td><td>94</td><td>8120</td><td>190</td><td>35980</td><td>P0CG47|O75947|P0DMV8|Q969T9|P16615|P62280|P36542|P35613|P0CG48|P39019|P16860|P05090|P13073|P00505|O14949|P39023|P62273|P46783|P83731|P62913|P24310|P69905|P62158|P24311|P62753|P20674|Q13765|Q07020|P01033|P10606|P48047|Q9Y277|P19021|P19022|P14406|P37108|Q9Y6H1|Q08431|P84098|O14958|P63173|P61353|P47985|P46777|P05386|P60866|P46776|P05387|P62249|P07602|P02792|P25398|P09669|P51684|P68363|O00483|P14854|P10176|P12235|P13693|P62891|P42766|Q02878|P25705|P18859|P60033|P68871|P61247|P62857|P62979|P56381|P62851|P00441|P54368|P02144|Q9P0U1|P08708|O75116|P62906|P18077|P15954|Q6P6C2|P42677|P41222|P15880|Q02543|Q00325|P62263|P62987|P62945|P04275|P62269|P25116|P06576</td></tr>
<tr><td>GO:0051223</td><td>regulation of protein transport</td><td>1.07E-02</td><td>3.23E-02</td><td>10</td><td>803</td><td>190</td><td>35980</td><td>P54368|Q14116|P63244|Q08493|P05090|Q9P0U1|P19021|Q02818|P12235|P25116</td></tr>
<tr><td>GO:0051234</td><td>establishment of localization</td><td>1.06E-19</td><td>2.56E-18</td><td>93</td><td>7073</td><td>190</td><td>35980</td><td>P0CG47|O75947|P0DMV8|Q969T9|P16615|P62280|P36542|P35613|P0CG48|P39019|P16860|P05090|P13073|P00505|O14949|P39023|P62273|P46783|P83731|P62913|P24310|P69905|P62158|P24311|P62753|P20674|Q13765|Q07020|P01033|P10606|P48047|Q9Y277|P19021|P19022|P14406|P37108|Q9Y6H1|Q08431|P84098|O14958|P63173|P61353|P47985|P46777|P05386|P60866|P46776|P05387|P62249|P07602|P02792|P25398|P09669|P68363|O00483|P14854|P10176|P12235|P13693|P62891|P42766|Q02878|P25705|P18859|P60033|P68871|P61247|P62857|P62979|P56381|P62851|P00441|P54368|P02144|Q9P0U1|P08708|O75116|P62906|P18077|P15954|Q6P6C2|P42677|P41222|P15880|Q02543|Q00325|P62263|P62987|P62945|P04275|P62269|P25116|P06576</td></tr>
<tr><td>GO:0051239</td><td>regulation of multicellular organismal process</td><td>1.13E-11</td><td>1.76E-10</td><td>45</td><td>2799</td><td>190</td><td>35980</td><td>P0CG47|P10916|P51684|P0DMV8|P0DMV9|P01160|P16615|Q8N3K9|P0CG48|P39019|P63244|P16860|P05090|P19429|Q9UBY9|P09493|Q16654|P23327|P62979|P62158|P45379|P00441|P01034|Q13765|P01033|Q14116|Q14896|O75116|P19022|P16989|P17661|P13533|P17535|P12883|P41222|O14958|P08493|P09382|P08590|P62987|P04792|P25116|O14558|Q8N335|P63316</td></tr>
<tr><td>GO:0051240</td><td>positive regulation of multicellular organismal process</td><td>1.55E-05</td><td>1.23E-04</td><td>22</td><td>1498</td><td>190</td><td>35980</td><td>P0CG47|Q13765|Q14116|P51684|P0DMV8|P0DMV9|P01160|O75116|P16615|P16989|P17535|P0CG48|P39019|P63244|P16860|P09493|P62987|P23327|P62979|P04792|P25116|P00441</td></tr>
<tr><td>GO:0051241</td><td>negative regulation of multicellular organismal process</td><td>4.59E-05</td><td>3.26E-04</td><td>17</td><td>1049</td><td>190</td><td>35980</td><td>P0CG47|P01033|P01160|O75116|P16615|P16989|P0CG48|P39019|P16860|P41222|P05090|P09382|P62987|P62979|P25116|P00441|P01034</td></tr>
<tr><td>GO:0051246</td><td>regulation of protein metabolic process</td><td>3.23E-06</td><td>2.92E-05</td><td>36</td><td>3024</td><td>190</td><td>35980</td><td>P07602|P0CG47|P0DMV8|P0DMV9|Q969T9|Q8N3K9|P0CG48|P63244|P05090|P60033|P23327|P62979|P62158|P00441|P01034|P51452|P54368|P01033|Q14116|P13639|P04406|O75116|P19022|P16989|Q05639|P63173|P68104|Q9NWT8|P62263|P62987|Q14240|P02511|P04792|P60228|P25116|Q8N335</td></tr>
<tr><td>GO:0051247</td><td>positive regulation of protein metabolic process</td><td>1.30E-03</td><td>6.03E-03</td><td>19</td><td>1657</td><td>190</td><td>35980</td><td>P07602|P0CG47|P54368|Q14116|P13639|Q969T9|O75116|P19022|P16989|P0CG48|P63244|Q9NWT8|P60033|P62987|P62979|P62158|P04792|P25116|P00441</td></tr>
<tr><td>GO:0051248</td><td>negative regulation of protein metabolic process</td><td>6.43E-05</td><td>4.41E-04</td><td>19</td><td>1299</td><td>190</td><td>35980</td><td>P0CG47|P51452|P01033|P0DMV8|P0DMV9|P04406|O75116|Q8N3K9|P0CG48|P63244|P05090|P62987|P62979|P02511|P62158|P04792|P60228|Q8N335|P01034</td></tr>
<tr><td>GO:0051254</td><td>positive regulation of RNA metabolic process</td><td>1.40E-02</td><td>4.03E-02</td><td>16</td><td>1644</td><td>190</td><td>35980</td><td>P0CG47|Q13765|P0DMV8|P0DMV9|Q969T9|Q8TAK5|Q9Y6H1|P17535|P0CG48|P60033|Q14192|P62987|P62979|Q13772|P17081|P25116</td></tr>
<tr><td>GO:0051259</td><td>protein oligomerization</td><td>6.08E-05</td><td>4.19E-04</td><td>11</td><td>486</td><td>190</td><td>35980</td><td>P68871|P19021|P19022|P22352|Q16698|P02511|P04275|P69905|P45379|P24752|P39019</td></tr>
<tr><td>GO:0051260</td><td>protein homooligomerization</td><td>5.55E-03</td><td>1.93E-02</td><td>6</td><td>302</td><td>190</td><td>35980</td><td>P19021|P22352|Q16698|P02511|P04275|P24752</td></tr>
<tr><td>GO:0051270</td><td>regulation of cellular component movement</td><td>1.26E-04</td><td>7.90E-04</td><td>14</td><td>819</td><td>190</td><td>35980</td><td>P01033|Q14896|P51684|O75116|P16615|P39019|P63244|P05090|P60033|P09493|P04792|P45379|P25116|P63316</td></tr>
<tr><td>GO:0051279</td><td>regulation of release of sequestered calcium ion into</td><td>7.92E-04</td><td>3.92E-03</td><td>4</td><td>78</td><td>190</td><td>35980</td><td>O14958|P23327|P62158|P25116</td></tr>
<tr><td>GO:0051282</td><td>regulation of sequestering of calcium ion</td><td>3.51E-03</td><td>1.36E-02</td><td>4</td><td>117</td><td>190</td><td>35980</td><td>O14958|P23327|P62158|P25116</td></tr>
<tr><td>GO:0051291</td><td>protein heterooligomerization</td><td>1.41E-03</td><td>6.42E-03</td><td>4</td><td>91</td><td>190</td><td>35980</td><td>P68871|P19022|P69905|P45379</td></tr>
<tr><td>GO:0051336</td><td>regulation of hydrolase activity</td><td>8.67E-03</td><td>2.78E-02</td><td>18</td><td>1834</td><td>190</td><td>35980</td><td>P01033|Q14896|P0DMV8|O75116|Q8N3K9|P13533|P63244|P19429|P08590|P09493|P02511|P62158|P45379|P25116|P20585|P00441|P63316|P01034</td></tr>
<tr><td>GO:0051338</td><td>regulation of transferase activity</td><td>1.49E-03</td><td>6.66E-03</td><td>15</td><td>1172</td><td>190</td><td>35980</td><td>P0CG47|P51452|P0CG48|P63244|P15880|Q05639|P60033|P62987|Q14240|P62979|P62158|P04792|Q8WYQ3|P25116|P00441</td></tr>
<tr><td>GO:0051340</td><td>regulation of ligase activity</td><td>1.00E-02</td><td>3.16E-02</td><td>4</td><td>158</td><td>190</td><td>35980</td><td>P0CG47|P62987|P62979|P0CG48</td></tr>
<tr><td>GO:0051346</td><td>negative regulation of hydrolase activity</td><td>1.63E-02</td><td>4.45E-02</td><td>7</td><td>493</td><td>190</td><td>35980</td><td>P01033|P19429|O75116|Q8N3K9|P02511|P45379|P01034</td></tr>
<tr><td>GO:0051347</td><td>positive regulation of transferase activity</td><td>6.94E-03</td><td>2.33E-02</td><td>10</td><td>752</td><td>190</td><td>35980</td><td>P0CG47|P15880|Q05639|P60033|P62987|P62979|P62158|P25116|P00441|P0CG48</td></tr>
<tr><td>GO:0051348</td><td>negative regulation of transferase activity</td><td>2.77E-04</td><td>1.58E-03</td><td>9</td><td>396</td><td>190</td><td>35980</td><td>P0CG47|P51452|P63244|P62987|Q14240|P62979|P04792|Q8WYQ3|P0CG48</td></tr>
<tr><td>GO:0051351</td><td>positive regulation of ligase activity</td><td>2.22E-03</td><td>9.26E-03</td><td>4</td><td>103</td><td>190</td><td>35980</td><td>P0CG47|P62987|P62979|P0CG48</td></tr>
</table>

GO:0051352	negative regulation of ligase activity	2.55E-03	1.04E-02	4	107	190	35980	P0CG47|P62987|P62979|P0CG48
GO:0051384	response to glucocorticoid	1.99E-03	8.47E-03	5	167	190	35980	P41222|P19021|P17174|P62158|Q8N8D1
GO:0051403	stress-activated MAPK cascade	7.56E-05	5.12E-04	6	132	190	35980	P0CG47|P51452|P62987|P62979|P02511|P0CG48
GO:0051436	negative regulation of ubiquitin-protein ligase activity involved in mitotic cell cycle	1.85E-03	7.97E-03	4	98	190	35980	P0CG47|P62987|P62979|P0CG48
GO:0051437	positive regulation of ubiquitin-protein ligase activity involved in regulation of mitotic cell cycle transition	6.50E-04	3.28E-03	4	74	190	35980	P0CG47|P62987|P62979|P0CG48
GO:0051438	regulation of ubiquitin-protein transferase activity	9.81E-03	3.10E-02	4	157	190	35980	P0CG47|P62987|P62979|P0CG48
GO:0051439	regulation of ubiquitin-protein ligase activity involved in	2.63E-03	1.06E-02	4	108	190	35980	P0CG47|P62987|P62979|P0CG48
GO:0051443	positive regulation of ubiquitin-protein transferase activity	1.85E-03	7.97E-03	4	98	190	35980	P0CG47|P62987|P62979|P0CG48
GO:0051444	negative regulation of ubiquitin-protein transferase activity	2.38E-03	9.82E-03	4	105	190	35980	P0CG47|P62987|P62979|P0CG48
GO:0051592	response to calcium ion	3.62E-03	1.39E-02	4	118	190	35980	O14958|P62158|P45379|P17535
GO:0051604	protein maturation	7.62E-03	2.51E-02	6	323	190	35980	P0CG47|P63244|P62987|P62979|P25116|P0CG48
GO:0051641	cellular localization	6.56E-22	1.81E-20	63	3063	190	35980	P0CG47|O75947|P0DMV8|P16615|P62280|P36542|P35613|P0CG48|P39019|P39023|P62273|P46783|P83731|P62913|P62158|P62753|Q07020|P01033|P48047|P19022|P37108|Q9Y6H1|P84098|P63173|P61353|P46777|P05386|P60866|P46776|P05387|P62249|P07602|P02792|P25398|P68363|P62891|P42766|Q02878|P25705|P18859|P60033|P61247|P62857|P62979|P56381|P62851|P00441|Q9P0U1|P08708|O75116|P62906|P18077|Q6P6C2|P42677|P15880|Q02543|P62263|P62987|P62945|P04275|P62269|P25116|P06576
GO:0051649	establishment of localization in cell	2.66E-23	8.07E-22	60	2598	190	35980	P0CG47|O75947|P0DMV8|P16615|P62280|P36542|P35613|P0CG48|P39019|P39023|P62273|P46783|P83731|P62913|P62158|P62753|Q07020|P01033|P48047|P37108|Q9Y6H1|P84098|P63173|P61353|P46777|P05386|P60866|P46776|P05387|P62249|P07602|P02792|P25398|P68363|P62891|P42766|Q02878|P25705|P18859|P61247|P62857|P62979|P56381|P62851|P00441|Q9P0U1|P08708|P62906|P18077|Q6P6C2|P42677|P15880|Q02543|P62263|P62987|P62945|P04275|P62269|P25116|P06576
GO:0051704	multi-organism process	7.81E-27	3.46E-25	64	2565	190	35980	P0CG47|P0DMV8|P0DMV9|P01160|P40925|P62280|P35613|P0CG48|P39019|P63244|Q14094|P39023|P62273|P46783|P83731|P62913|P62753|P01034|Q13765|Q07020|P19021|P16989|Q08431|P84098|P63173|P61353|P46777|P05386|P60866|P46776|P05387|P62249|P52179|P25398|P10176|P12235|P13693|P62891|P42766|Q02878|P60033|P61247|P62857|Q16654|P62979|P62851|Q01628|P00441|Q14116|P08708|P62906|P18077|Q6P6C2|P17535|P42677|P15880|Q02543|P62263|P62987|Q14240|P62945|P04792|P62269|P25116
GO:0051707	response to other organism	1.43E-03	6.49E-03	13	931	190	35980	P52179|Q14116|P40925|Q6P6C2|P10176|P17535|P13693|P62891|P62987|Q16654|P04792|Q01628|P25116
GO:0051726	regulation of cell cycle	1.68E-02	4.58E-02	13	1260	190	35980	P0CG47|P51452|O75116|P17535|P0CG48|P63244|Q14094|Q9NWT8|P62987|P83731|P62979|Q15004|P62158
GO:0051806	entry into cell of other organism involved in symbiotic	1.11E-02	3.31E-02	3	87	190	35980	P0DMV8|P0DMV9|P60033
GO:0051828	entry into other organism involved in symbiotic interaction	1.11E-02	3.31E-02	3	87	190	35980	P0DMV8|P0DMV9|P60033
GO:0051881	regulation of mitochondrial membrane potential	3.61E-03	1.39E-02	3	58	190	35980	P0CG47|P63244|P00441
GO:0051924	regulation of calcium ion transport	8.55E-03	2.76E-02	5	236	190	35980	O14958|P01160|P23327|P62158|P25116
GO:0052126	movement in host environment	1.11E-02	3.31E-02	3	87	190	35980	P0DMV8|P0DMV9|P60033
GO:0052192	movement in environment of other organism involved in	1.11E-02	3.31E-02	3	87	190	35980	P0DMV8|P0DMV9|P60033
GO:0055001	muscle cell development	2.06E-12	3.42E-11	13	153	190	35980	Q14315|O15273|Q14896|P10916|P01160|P68133|P16615|P68032|P13533|O14958|Q14192|P09493|P45379
GO:0055002	striated muscle cell development	1.34E-11	2.09E-10	12	140	190	35980	Q14315|O15273|Q14896|P10916|O14958|Q14192|P01160|P09493|P68133|P68032|P45379|P13533
GO:0055003	cardiac myofibril assembly	7.72E-05	5.20E-04	3	16	190	35980	O15273|P10916|P68032
GO:0055006	cardiac cell development	1.16E-06	1.14E-05	6	64	190	35980	O15273|P10916|Q14192|P01160|P68032|P13533
GO:0055007	cardiac muscle cell differentiation	1.04E-05	8.52E-05	6	93	190	35980	O15273|P10916|Q14192|P01160|P68032|P13533
GO:0055008	cardiac muscle tissue morphogenesis	9.93E-15	1.92E-13	11	57	190	35980	O15273|Q14896|P10916|P19429|P08590|P09493|P68032|P45379|P13533|P12883|P63316
GO:0055010	ventricular cardiac muscle tissue morphogenesis	7.99E-13	1.36E-11	9	41	190	35980	Q14896|P10916|P19429|P08590|P09493|P45379|P13533|P12883|P63316
GO:0055013	cardiac muscle cell development	8.74E-07	8.60E-06	6	61	190	35980	O15273|P10916|Q14192|P01160|P68032|P13533
GO:0055065	metal ion homeostasis	2.28E-03	9.45E-03	9	535	190	35980	P13693|P02792|P51684|O14958|P19429|P16615|P23327|P25116|P00441
GO:0055074	calcium ion homeostasis	2.15E-03	9.05E-03	7	336	190	35980	P13693|P51684|O14958|P19429|P16615|P23327|P25116
GO:0055080	cation homeostasis	3.81E-04	2.10E-03	11	601	190	35980	P13693|P02792|P51684|O14958|P19429|P16615|Q16654|P23327|P25116|P00441|P06576
GO:0055082	cellular chemical homeostasis	1.36E-04	8.43E-04	12	626	190	35980	P13693|P02792|P63244|P51684|O14958|P19429|P09382|P16615|P23327|P25116|P00441|P06576
GO:0055085	transmembrane transport	1.58E-06	1.49E-05	32	2432	190	35980	P0CG47|O75947|P02792|P09669|O00483|P14854|P16615|P36542|P10176|P12235|P0CG48|P13073|P25705|P18859|O14949|P62979|P56381|P24310|P24311|P20674|P10606|Q9P0U1|P48047|Q9Y277|P14406|P15954|O14958|Q00325|P62987|P47985|P25116|P06576
GO:0055086	nucleobase-containing small molecule metabolic process	3.05E-22	8.48E-21	37	906	190	35980	P13929|O75947|P0DMV8|P0DMV9|P01160|O43674|P40926|P40925|O00483|P36542|P10176|O95178|O96000|P16860|P13073|P25705|P18859|Q9NPI5|P07195|P56381|Q8WYQ3|O15239|P20674|O00217|Q08493|O95169|P51970|P48047|P04406|O14561|O43920|P13533|O95167|P12883|P17081|Q8N335|P06576
GO:0055114	oxidation-reduction process	9.69E-20	2.36E-18	51	2195	190	35980	P0CG47|O75947|O43674|P40926|P40925|P36542|P0CG48|O96000|P13073|O14949|P07195|P62158|P24311|O15239|P20674|P10606|O95169|P48047|P04406|O14561|O14880|P19021|O43920|O95167|P08574|P47985|Q13011|Q8N335|P13929|P09669|O00483|P14854|P10176|O95178|P12235|P25705|P18859|P22352|Q16698|P62979|P56381|P00441|O00217|P51970|P15954|P51857|Q6P6C2|Q9Y3D2|P62987|P06576|P48735
GO:0055117	regulation of cardiac muscle contraction	2.68E-05	2.05E-04	5	66	190	35980	O14958|P01160|P16615|P23327|P62158
GO:0060047	heart contraction	8.97E-18	1.96E-16	14	80	190	35980	O15273|Q14896|P10916|P68032|P13533|P12883|O14958|P19429|P08590|P09493|P45379|Q8N335|P00441|P63316
GO:0060048	cardiac muscle contraction	4.89E-17	1.03E-15	13	69	190	35980	O15273|Q14896|P10916|P68032|P13533|P12883|O14958|P19429|P08590|P09493|P45379|Q8N335|P63316
GO:0060314	regulation of ryanodine-sensitive calcium-release channel	3.45E-04	1.91E-03	3	26	190	35980	O14958|P23327|P62158
GO:0060322	head development	8.84E-03	2.83E-02	10	780	190	35980	P0CG47|P05090|P84243|P18859|O14949|P19022|P14854|P62158|P24752|P01034
GO:0060341	regulation of cellular localization	3.42E-06	3.07E-05	22	1360	190	35980	P54368|Q14116|Q08493|Q14896|P0DMV8|Q9P0U1|P19021|P19022|P16615|P12235|P63244|P05090|O14958|Q02818|P23327|P02511|P62158|P17081|P45379|P25116|Q8N335|P63316
GO:0060415	muscle tissue morphogenesis	9.29E-14	1.69E-12	11	69	190	35980	O15273|Q14896|P10916|P19429|P08590|P09493|P68032|P45379|P13533|P12883|P63316
GO:0060537	muscle tissue development	1.12E-11	1.76E-10	16	309	190	35980	O15273|Q14896|P10916|P13639|P01160|P68133|P68032|P13533|P12883|P19429|Q14192|P60660|P08590|P09493|P45379|P63316
GO:0060538	skeletal muscle organ development	8.39E-03	2.72E-02	4	150	190	35980	P13639|P60660|P08590|P68133
GO:0060548	negative regulation of cell death	2.58E-08	3.02E-07	22	1017	190	35980	P07602|P0CG47|Q13765|P01033|P0DMV8|P0DMV9|P16989|P68032|P12235|P0CG48|P13693|P63244|Q14192|P62987|P61247|Q16654|P62979|P02511|P04792|P25116|P00441|P01034
GO:0060759	regulation of response to cytokine stimulus	1.46E-05	1.17E-04	7	148	190	35980	P0CG47|P63244|P0DMV8|P0DMV9|P62987|P62979|P0CG48
GO:0060828	regulation of canonical Wnt signaling pathway	1.17E-02	3.46E-02	5	255	190	35980	P0CG47|P19022|P62987|P62979|P0CG48
GO:0061024	membrane organization	2.31E-29	1.16E-27	49	1211	190	35980	P0CG47|P0DMV8|P16615|P62280|P35613|P0CG48|P39019|P39023|P62273|P46783|P83731|P62913|P62158|P62753|Q07020|P19022|P37108|Q08431|P84098|P63173|P61353|P46777|P05386|P60866|P46776|P05387|P62249|P02792|P25398|P62891|P42766|Q02878|P61247|P62857|P62979|P62851|P00441|Q9P0U1|P08708|O75116|P62906|P18077|P42677|P15880|Q02543|P62263|P62987|P62945|P62269
GO:0061061	muscle structure development	3.78E-14	6.98E-13	22	502	190	35980	Q14315|O15273|Q14896|P10916|P13639|P01160|P19022|P68133|P16615|P68032|P13533|P12883|O14958|P19429|Q14192|P60660|P09382|P08590|P09493|P02511|P45379|P63316
GO:0061337	cardiac conduction	4.57E-03	1.71E-02	4	126	190	35980	O14958|P19429|P01160|P16615
GO:0061418	regulation of transcription from RNA polymerase II promoter in response to hypoxia	2.73E-05	2.08E-04	4	33	190	35980	P0CG47|P62987|P62979|P0CG48
GO:0061458	reproductive system development	2.80E-03	1.11E-02	8	449	190	35980	P07602|P0CG47|P16989|P04275|Q13772|P35613|P00441|P01034
GO:0061564	axon development	5.84E-03	2.01E-02	10	733	190	35980	P0CG47|P05090|P60660|O75116|P62987|P83731|P62979|P62158|P19105|P0CG48
GO:0061726	mitochondrion disassembly	6.34E-06	5.43E-05	8	183	190	35980	P0CG47|P52179|Q9P0U1|P40925|P62987|P62979|P10176|P0CG48
GO:0065003	macromolecular complex assembly	1.27E-13	2.30E-12	37	1678	190	35980	O43674|O95178|P39019|O96000|Q02878|P84243|P39023|P68871|P46783|P83731|P62913|P22352|Q16698|P69905|P45379|P24752|O15239|O00217|O15273|Q14896|O95169|P51970|P08708|O14561|P19021|P19022|O43920|O95167|P42677|O14958|P63173|Q9Y3D2|P62263|P46777|P02511|P04275|P60228

GO:0065007	biological regulation	8.63E-03	2.77E-02	113	18213	190	35980	P10916|Q969T9|P16615|P16860|P09493|P83731|P62753|Q13765|P13639|Q9Y277|P04406|P80297|P16989|Q9Y6H1|Q08431|P19105|O14958|P63173|P68104|P09382|Q13772|P60228|O14558|Q8N335|P07602|P52179|Q6GTX8|Q8N3K9|P10176|P12235|P13693|P19429|P25705|Q14192|P68871|Q16654|Q01628|P00441|P54368|Q14116|Q08493|P08708|Q14515|O75116|P68133|P17661|Q6P6C2|P17535|P12883|Q8N8D1|P42677|P41222|P15880|Q9NWT8|Q9Y3D2|P62263|Q14240|P0CG47|P0DMV8|P0DMV9|P01160|P40925|Q8TAK5|P35613|P0CG48|P39019|P63244|P05090|Q14094|Q02818|P69905|P62158|P45379|Q8WYQ3|P01034|P01033|Q14896|Q96H40|O14880|P19021|P19022|P68032|Q05639|P08493|P60660|P02511|P17081|P02792|P51684|Q02878|P84243|P60033|Q9UBY9|Q9NPI5|P17174|P61247|P23327|P62979|Q15004|P51452|P02144|Q9P0U1|O75954|P51857|P13533|P08590|P62987|P04275|P04792|P25116|P20585|P06576|P63316
GO:0065008	regulation of biological quality	1.17E-10	1.72E-09	55	4225	190	35980	P0CG47|P10916|P0DMV8|P0DMV9|P01160|P16615|P35613|P0CG48|P39019|P63244|P16860|P09493|P62158|P45379|P62753|P01033|Q14896|P04406|P19021|P19022|P16989|P19105|O14958|P09382|P17081|Q8N335|P07602|P02792|P51684|P12235|P13693|P19429|P84243|P60033|P68871|P17174|Q16654|P23327|P62979|P00441|P02144|Q08493|Q9P0U1|P08708|P51857|P13533|P12883|P08590|P62263|P62987|P04275|P04792|P25116|P06576|P63316
GO:0065009	regulation of molecular function	3.10E-04	1.74E-03	36	3759	190	35980	P07602|P0CG47|P0DMV8|P0DMV9|P01160|Q8N3K9|P0CG48|P63244|P19429|P60033|P09493|P23327|P62979|P62158|P45379|Q8WYQ3|P00441|P01034|P51452|P54368|P01033|Q14896|O75116|P13533|O14958|P15880|Q05639|P08590|P62987|Q14240|P02511|P04792|P25116|P20585|Q8N335|P63316
GO:0070201	regulation of establishment of protein localization	7.26E-03	2.41E-02	11	878	190	35980	P54368|Q14116|P63244|Q08493|P05090|Q9P0U1|P19021|Q02818|P17081|P12235|P25116
GO:0070252	actin-mediated cell contraction	8.46E-20	2.08E-18	15	76	190	35980	O15273|Q14896|P10916|P68133|P17661|P68032|P13533|P12883|P19429|P60660|P08590|P09493|P45379|Q8N335|P63316
GO:0070265	necrotic cell death	4.83E-05	3.41E-04	4	38	190	35980	P0CG47|P62987|P62979|P0CG48
GO:0070266	necroptotic process	1.61E-05	1.28E-04	4	29	190	35980	P0CG47|P62987|P62979|P0CG48
GO:0070271	protein complex biogenesis	1.70E-08	2.08E-07	26	1366	190	35980	O43674|O95178|P39019|O96000|P84243|P68871|P22352|Q16698|P69905|P45379|P24752|O15239|O00217|O15273|Q14896|O95169|P51970|O14561|P19021|P19022|O43920|O95167|O14958|Q9Y3D2|P02511|P04275
GO:0070423	nucleotide-binding oligomerization domain containing	1.40E-05	1.12E-04	4	28	190	35980	P0CG47|P62987|P62979|P0CG48
GO:0070482	response to oxygen levels	1.14E-05	9.25E-05	10	330	190	35980	P0CG47|P02144|P01160|P19021|P62987|P62979|P02511|Q6P6C2|P0CG48|P01034
GO:0070527	platelet aggregation	1.43E-03	6.49E-03	3	42	190	35980	P68871|P04792|P19105
GO:0070727	cellular macromolecule localization	3.47E-19	8.12E-18	44	1652	190	35980	P25398|P62280|P35613|P39019|P62891|P42766|Q02878|P60033|P39023|P62273|P46783|P61247|P83731|P62857|P62913|P62979|P62851|P62753|Q07020|Q9P0U1|P08708|O75116|P62906|P19022|P18077|P37108|Q9Y6H1|P42677|P84098|P15880|P63173|Q02543|P61353|P62263|P62987|P62945|P46777|P05386|P60866|P46776|P62269|P05387|P25116|P62249
GO:0070848	response to growth factor	8.00E-03	2.62E-02	11	890	190	35980	P0CG47|P51452|P63244|P13639|P68104|P62987|P62979|P62158|P04792|P13533|P0CG48
GO:0070887	cellular response to chemical stimulus	2.34E-08	2.76E-07	41	3060	190	35980	P07602|P0CG47|P51684|P0DMV8|P0DMV9|Q969T9|P68363|P16615|P0CG48|P39019|P63244|P60033|Q14192|P39023|P09493|P17174|Q16654|P62979|P62158|P62753|Q01628|P00441|P01034|P51452|Q14116|P13639|P04406|O75116|O14880|P80297|P16989|P13533|P17535|O14958|P68104|P09382|P62987|Q14240|Q13772|P17081|P04792
GO:0070911	global genome nucleotide-excision repair	3.31E-04	1.86E-03	4	62	190	35980	P0CG47|P62987|P62979|P0CG48
GO:0070925	organelle assembly	3.32E-10	4.72E-09	20	651	190	35980	O15273|Q14896|P10916|P08708|P68133|P68032|P13533|P39019|P42677|Q02878|O14958|P63173|P39023|P09493|P62263|P46783|P83731|P62913|P46777|P45379
GO:0070972	protein localization to endoplasmic reticulum	2.64E-51	6.55E-49	37	152	190	35980	P25398|P62280|P39019|P62891|P42766|Q02878|P39023|P62273|P46783|P61247|P83731|P62857|P62913|P62979|P62851|P62753|Q07020|P08708|P62906|P18077|P37108|P42677|P84098|P15880|P63173|Q02543|P61353|P62263|P62987|P62945|P46777|P05386|P60866|P46776|P62269|P05387|P62249
GO:0070987	error-free translesion synthesis	2.74E-06	2.52E-05	4	19	190	35980	P0CG47|P62987|P62979|P0CG48
GO:0071156	regulation of cell cycle arrest	4.57E-03	1.71E-02	4	126	190	35980	P0CG47|P62987|P62979|P0CG48
GO:0071158	positive regulation of cell cycle arrest	1.35E-03	6.20E-03	4	90	190	35980	P0CG47|P62987|P62979|P0CG48
GO:0071310	cellular response to organic substance	4.16E-07	4.21E-06	34	2525	190	35980	P07602|P0CG47|P51684|Q969T9|P68363|P0CG48|P63244|P60033|Q14192|P39023|P17174|Q16654|P62979|P62158|P62753|Q01628|P00441|P51452|Q14116|P13639|P04406|O75116|P80297|P16989|P13533|P17535|O14958|P68104|P09382|P62987|Q14240|Q13772|P17081|P04792
GO:0071345	cellular response to cytokine stimulus	7.78E-04	3.88E-03	13	870	190	35980	P0CG47|P51684|P04406|P80297|P68363|P16989|P0CG48|P63244|P39023|P62987|Q14240|P62979|Q01628
GO:0071356	cellular response to tumor necrosis factor	2.21E-03	9.25E-03	6	250	190	35980	P0CG47|P63244|P62987|P16989|P62979|P0CG48
GO:0071363	cellular response to growth factor stimulus	6.53E-03	2.21E-02	11	865	190	35980	P0CG47|P51452|P63244|P13639|P68104|P62987|P62979|P62158|P04792|P13533|P0CG48
GO:0071375	cellular response to peptide hormone stimulus	1.06E-03	5.04E-03	9	478	190	35980	P0CG47|P17174|P62987|Q16654|P62979|P62158|P17081|P62753|P0CG48
GO:0071407	cellular response to organic cyclic compound	1.17E-02	3.46E-02	8	574	190	35980	Q14116|O14958|Q969T9|Q14192|P09382|O75116|Q13772|P00441
GO:0071417	cellular response to organonitrogen compound	7.94E-04	3.92E-03	11	657	190	35980	P0CG47|O14958|P17174|P62987|Q16654|P62979|P62158|P17081|P62753|P00441|P0CG48
GO:0071453	cellular response to oxygen levels	8.39E-03	2.72E-02	4	150	190	35980	P0CG47|P62987|P62979|P0CG48
GO:0071456	cellular response to hypoxia	5.68E-03	1.97E-02	4	134	190	35980	P0CG47|P62987|P62979|P0CG48
GO:0071495	cellular response to endogenous stimulus	5.29E-05	3.71E-04	20	1393	190	35980	P0CG47|P13639|Q969T9|O75116|P13533|P17535|P0CG48|O14958|P68104|P60033|Q14192|P17174|P62987|Q16654|P62979|Q13772|P62158|P17081|P62753|P00441
GO:0071702	organic substance transport	3.46E-14	6.43E-13	50	2889	190	35980	P62280|P35613|P39019|P05090|P00505|P39023|P62273|P46783|P83731|P62913|P69905|P62753|Q13765|Q07020|Q9Y277|P37108|Q9Y6H1|P84098|P63173|P61353|P46777|P05386|P60866|P46776|P05387|P62249|P07602|P25398|P12235|P62891|P42766|Q02878|P68871|P61247|P62857|P62979|P62851|Q9P0U1|P08708|P62906|P18077|Q6P6C2|P42677|P15880|Q02543|P62263|P62987|P62945|P62269|P25116
GO:0071704	organic substance metabolic process	3.03E-14	5.73E-13	137	16191	190	35980	O75947|O43674|O96000|P16860|P13073|P07195|P46783|P83731|P62913|P62753|P24752|O15239|P20674|Q13765|O95169|P13639|P48047|P04406|O14561|Q9Y5N6|P16989|Q9Y6H1|O95167|Q08431|P84098|P63173|P68104|P46777|Q13772|P60866|P46776|P60228|P62249|Q13011|Q8N335|P07602|P25398|P68363|P10176|O95178|P12235|P62891|P25705|P18859|Q14192|Q16654|P56381|P00441|P54368|O00217|Q14116|Q08493|P08708|O75116|P18077|Q6P6C2|P17535|P12883|Q8N8D1|P42677|P41222|P15880|P17540|Q02543|Q9NWT8|Q9Y3D2|P62263|Q14240|P62945|P62269|P0CG47|P06732|Q9NSD9|P0DMV8|P0DMV9|P01160|P40926|P40925|P62280|Q8TAK5|P36542|P35613|P0CG48|P39019|P63244|P05090|P00505|P39023|P62273|P62158|P24311|Q8WYQ3|P01034|Q07020|P10606|Q96H40|O14880|P19021|P37108|O43920|Q05639|P61353|P02511|P05386|P17081|P05387|P13929|P09669|O00483|P14854|P42766|Q02878|P84243|P60033|Q9NPI5|P17174|P61247|P62857|Q16698|P62979|Q15004|P62851|P51452|P51970|Q9P0U1|P62906|P15954|P51857|P13533|Q00325|P62987|P04275|P04792|P25116|P20585|P06576|P48735
GO:0071822	protein complex subunit organization	1.39E-34	9.58E-33	66	2022	190	35980	O43674|P62280|P39019|O96000|P39023|P62273|P46783|P83731|P62913|P69905|P45379|P62753|P24752|O15239|Q07020|Q14896|O95169|O14561|P19021|P19022|O43920|P68032|O95167|P84098|O14958|P63173|P61353|P46777|P02511|P05386|P60866|P46776|P05387|P62249|P25398|O95178|P62891|P42766|Q02878|P84243|P68871|P61247|P62857|P22352|Q16698|P62979|P62851|O00217|O15273|P51970|P08708|P62906|P68133|P18077|P17661|P42677|P15880|Q02543|Q9NWT8|Q9Y3D2|P62263|P62987|P62945|P04275|P63313|P62269
GO:0071826	ribonucleoprotein complex subunit organization	1.64E-08	2.01E-07	12	260	190	35980	P42677|Q02878|P63173|P08708|P39023|P62263|P46783|P83731|P62913|P46777|P60228|P39019
GO:0071840	cellular component organization or biogenesis	2.36E-23	7.22E-22	101	7374	190	35980	P10916|O43674|P16615|O96000|P09493|P46783|P83731|P62913|P62753|P24752|O15239|O95169|P04406|O14561|Q9Y6H1|O95167|Q08431|P19105|P84098|O14958|P63173|P46777|P60866|P46776|P60228|P62249|P52179|P25398|P68363|P10176|O95178|P12235|P62891|P68871|P00441|O00217|O15273|P08708|O75116|P68133|P18077|P17661|P42677|P15880|Q02543|Q9NWT8|Q9Y3D2|P62263|P62945|P62269|P0CG47|P0DMV8|P40925|P62280|P35613|P0CG48|P39019|P05090|P39023|P62273|P69905|P62158|P45379|Q8WYQ3|P01034|Q07020|P01033|Q14896|P19021|P19022|P37108|O43920|P68032|P61353|P60660|P02511|P05386|P17081|P05387|P02792|P42766|Q02878|P84243|P61247|P62857|P22352|Q16698|P62979|Q15004|P62851|Q14315|P51970|Q9P0U1|P62906|P13533|P62987|P04275|P63313|P25116|P20585|P06576
GO:0071897	DNA biosynthetic process	7.01E-04	3.54E-03	5	132	190	35980	P0CG47|P62987|P62979|Q15004|P0CG48
GO:0071900	regulation of protein serine/threonine kinase activity	3.07E-03	1.21E-02	10	668	190	35980	P0CG47|P51452|P60033|P62987|P62979|P62158|P04792|P25116|P00441|P0CG48

<table>
<tr><td>GO:0071902</td><td>positive regulation of protein serine/threonine kinase</td><td>1.47E-03</td><td>6.62E-03</td><td>7</td><td>314</td><td>190</td><td>35980</td><td>P0CG47|P60033|P62987|P62979|P62158|P00441|P0CG48</td></tr>
<tr><td>GO:0072329</td><td>monocarboxylic acid catabolic process</td><td>2.72E-03</td><td>1.09E-02</td><td>4</td><td>109</td><td>190</td><td>35980</td><td>P00505|Q16698|P51857|Q13011</td></tr>
<tr><td>GO:0072331</td><td>signal transduction by p53 class mediator</td><td>6.45E-03</td><td>2.19E-02</td><td>4</td><td>139</td><td>190</td><td>35980</td><td>P0CG47|P62987|P62979|P0CG48</td></tr>
<tr><td>GO:0072350</td><td>tricarboxylic acid metabolic process</td><td>4.77E-03</td><td>1.77E-02</td><td>3</td><td>64</td><td>190</td><td>35980</td><td>P40926|P40925|P48735</td></tr>
<tr><td>GO:0072358</td><td>cardiovascular system development</td><td>2.01E-08</td><td>2.40E-07</td><td>21</td><td>915</td><td>190</td><td>35980</td><td>Q13765|P02144|Q14116|O15273|Q14896|P10916|P01160|P19021|P19022|P68032|P13533|Q08431|P12883|P05090|P19429|Q14192|P08590|P09493|P45379|P06576|P63316</td></tr>
<tr><td>GO:0072359</td><td>circulatory system development</td><td>2.01E-08</td><td>2.40E-07</td><td>21</td><td>915</td><td>190</td><td>35980</td><td>Q13765|P02144|Q14116|O15273|Q14896|P10916|P01160|P19021|P19022|P68032|P13533|Q08431|P12883|P05090|P19429|Q14192|P08590|P09493|P45379|P06576|P63316</td></tr>
<tr><td>GO:0072395</td><td>signal transduction involved in cell cycle checkpoint</td><td>5.55E-04</td><td>2.86E-03</td><td>4</td><td>71</td><td>190</td><td>35980</td><td>P0CG47|P62987|P62979|P0CG48</td></tr>
<tr><td>GO:0072401</td><td>signal transduction involved in DNA integrity checkpoint</td><td>5.26E-04</td><td>2.73E-03</td><td>4</td><td>70</td><td>190</td><td>35980</td><td>P0CG47|P62987|P62979|P0CG48</td></tr>
<tr><td>GO:0072413</td><td>signal transduction involved in mitotic cell cycle</td><td>4.71E-04</td><td>2.49E-03</td><td>4</td><td>68</td><td>190</td><td>35980</td><td>P0CG47|P62987|P62979|P0CG48</td></tr>
<tr><td>GO:0072422</td><td>signal transduction involved in DNA damage checkpoint</td><td>5.26E-04</td><td>2.73E-03</td><td>4</td><td>70</td><td>190</td><td>35980</td><td>P0CG47|P62987|P62979|P0CG48</td></tr>
<tr><td>GO:0072431</td><td>signal transduction involved in mitotic G1 DNA damage</td><td>4.45E-04</td><td>2.39E-03</td><td>4</td><td>67</td><td>190</td><td>35980</td><td>P0CG47|P62987|P62979|P0CG48</td></tr>
<tr><td>GO:0072503</td><td>cellular divalent inorganic cation homeostasis</td><td>2.23E-03</td><td>9.29E-03</td><td>7</td><td>338</td><td>190</td><td>35980</td><td>P13693|P51684|O14958|P19429|P16615|P23327|P25116</td></tr>
<tr><td>GO:0072507</td><td>divalent inorganic cation homeostasis</td><td>3.21E-03</td><td>1.26E-02</td><td>7</td><td>361</td><td>190</td><td>35980</td><td>P13693|P51684|O14958|P19429|P16615|P23327|P25116</td></tr>
<tr><td>GO:0072521</td><td>purine-containing compound metabolic process</td><td>2.48E-22</td><td>6.94E-21</td><td>32</td><td>619</td><td>190</td><td>35980</td><td>P13929|O75947|P0DMV8|P0DMV9|P01160|O43674|O00483|P36542|P10176|O95178|O96000|P16860|P13073|P25705|P18859|P56381|Q8WYQ3|O15239|P20674|O00217|Q08493|O95169|P51970|P48047|P04406|O14561|O43920|P13533|O95167|P12883|P17081|P06576</td></tr>
<tr><td>GO:0072522</td><td>purine-containing compound biosynthetic process</td><td>7.26E-07</td><td>7.19E-06</td><td>10</td><td>242</td><td>190</td><td>35980</td><td>O75947|P16860|P25705|P48047|P18859|P01160|P36542|P56381|Q8WYQ3|P06576</td></tr>
<tr><td>GO:0072524</td><td>pyridine-containing compound metabolic process</td><td>2.89E-04</td><td>1.64E-03</td><td>7</td><td>238</td><td>190</td><td>35980</td><td>P13929|P40926|P04406|Q9NPI5|P07195|P40925|Q8N335</td></tr>
<tr><td>GO:0072593</td><td>reactive oxygen species metabolic process</td><td>8.92E-05</td><td>5.80E-04</td><td>6</td><td>136</td><td>190</td><td>35980</td><td>P68871|Q16654|P22352|P69905|P62158|P00441</td></tr>
<tr><td>GO:0072594</td><td>establishment of protein localization to organelle</td><td>9.04E-38</td><td>9.27E-36</td><td>40</td><td>431</td><td>190</td><td>35980</td><td>P25398|P62280|P39019|P62891|P42766|Q02878|P39023|P62273|P46783|P61247|P83731|P62857|P62913|P62979|P62851|P62753|Q07020|Q9P0U1|P08708|P62906|P18077|P37108|Q9Y6H1|P42677|P84098|P15880|P63173|Q02543|P61353|P62263|P62987|P62945|P46777|P05386|P60866|P46776|P62269|P05387|P25116|P62249</td></tr>
<tr><td>GO:0072599</td><td>establishment of protein localization to endoplasmic reticulum</td><td>5.02E-54</td><td>1.36E-51</td><td>37</td><td>131</td><td>190</td><td>35980</td><td>P25398|P62280|P39019|P62891|P42766|Q02878|P39023|P62273|P46783|P61247|P83731|P62857|P62913|P62979|P62851|P62753|Q07020|P08708|P62906|P18077|P37108|P42677|P84098|P15880|P63173|Q02543|P61353|P62263|P62987|P62945|P46777|P05386|P60866|P46776|P62269|P05387|P62249</td></tr>
<tr><td>GO:0072657</td><td>protein localization to membrane</td><td>8.43E-40</td><td>9.29E-38</td><td>41</td><td>416</td><td>190</td><td>35980</td><td>P25398|P62280|P35613|P39019|P62891|P42766|Q02878|P39023|P62273|P46783|P61247|P83731|P62857|P62913|P62979|P62851|P62753|Q07020|Q9P0U1|P08708|O75116|P62906|P19022|P18077|P37108|P42677|P84098|P15880|P63173|Q02543|P61353|P62263|P62987|P62945|P46777|P05386|P60866|P46776|P62269|P05387|P62249</td></tr>
<tr><td>GO:0075733</td><td>intracellular transport of virus</td><td>2.41E-05</td><td>1.85E-04</td><td>4</td><td>32</td><td>190</td><td>35980</td><td>P0CG47|P62987|P62979|P0CG48</td></tr>
<tr><td>GO:0080134</td><td>regulation of response to stress</td><td>6.52E-07</td><td>6.50E-06</td><td>25</td><td>1540</td><td>190</td><td>35980</td><td>P07602|P0CG47|P52179|P0DMV8|P0DMV9|P40925|P10176|P0CG48|P39019|P13693|P63244|P05090|Q16654|P62979|P62158|P00441|P51452|Q14116|P16989|Q9Y6H1|Q6P6C2|P62987|P02511|P04792|P25116</td></tr>
<tr><td>GO:0080135</td><td>regulation of cellular response to stress</td><td>1.14E-03</td><td>5.39E-03</td><td>11</td><td>687</td><td>190</td><td>35980</td><td>P07602|P51452|P13693|P63244|P0DMV8|P0DMV9|P16989|Q9Y6H1|P02511|P04792|P00441</td></tr>
<tr><td>GO:0090002</td><td>establishment of protein localization to plasma membrane</td><td>1.33E-02</td><td>3.86E-02</td><td>3</td><td>93</td><td>190</td><td>35980</td><td>O75116|P19022|P35613</td></tr>
<tr><td>GO:0090068</td><td>positive regulation of cell cycle process</td><td>1.44E-02</td><td>4.13E-02</td><td>5</td><td>269</td><td>190</td><td>35980</td><td>P0CG47|O75116|P62987|P62979|P0CG48</td></tr>
<tr><td>GO:0090090</td><td>negative regulation of canonical Wnt signaling pathway</td><td>2.15E-03</td><td>9.05E-03</td><td>5</td><td>170</td><td>190</td><td>35980</td><td>P0CG47|P19022|P62987|P62979|P0CG48</td></tr>
<tr><td>GO:0090101</td><td>negative regulation of transmembrane receptor protein serine/threonine kinase signaling pathway</td><td>4.08E-03</td><td>1.55E-02</td><td>4</td><td>122</td><td>190</td><td>35980</td><td>P0CG47|P62987|P62979|P0CG48</td></tr>
<tr><td>GO:0090150</td><td>establishment of protein localization to membrane</td><td>7.76E-46</td><td>1.10E-43</td><td>41</td><td>299</td><td>190</td><td>35980</td><td>P25398|P62280|P35613|P39019|P62891|P42766|Q02878|P39023|P62273|P46783|P61247|P83731|P62857|P62913|P62979|P62851|P62753|Q07020|Q9P0U1|P08708|O75116|P62906|P19022|P18077|P37108|P42677|P84098|P15880|P63173|Q02543|P61353|P62263|P62987|P62945|P46777|P05386|P60866|P46776|P62269|P05387|P62249</td></tr>
<tr><td>GO:0090257</td><td>regulation of muscle system process</td><td>7.59E-15</td><td>1.48E-13</td><td>17</td><td>230</td><td>190</td><td>35980</td><td>Q14896|P10916|P01160|P16615|Q8N3K9|P12883|O14958|P19429|P08590|P09493|P23327|P62158|P45379|P25116|O14558|P00441|P63316</td></tr>
<tr><td>GO:0090263</td><td>positive regulation of canonical Wnt signaling pathway</td><td>6.29E-03</td><td>2.14E-02</td><td>4</td><td>138</td><td>190</td><td>35980</td><td>P0CG47|P62987|P62979|P0CG48</td></tr>
<tr><td>GO:0090279</td><td>regulation of calcium ion import</td><td>2.63E-03</td><td>1.06E-02</td><td>4</td><td>108</td><td>190</td><td>35980</td><td>O14958|P23327|P62158|P25116</td></tr>
<tr><td>GO:0090288</td><td>negative regulation of cellular response to growth factor</td><td>8.59E-03</td><td>2.76E-02</td><td>4</td><td>151</td><td>190</td><td>35980</td><td>P0CG47|P62987|P62979|P0CG48</td></tr>
<tr><td>GO:0090304</td><td>nucleic acid metabolic process</td><td>9.55E-11</td><td>1.41E-09</td><td>66</td><td>5665</td><td>190</td><td>35980</td><td>P0CG47|Q9NSD9|P01160|P62280|Q8TAK5|P0CG48|P39019|P13073|P39023|P62273|P46783|P83731|P62913|P24311|P62753|P20674|Q13765|Q07020|P10606|Q96H40|Q9Y5N6|P16989|Q9Y6H1|P84098|P63173|P68104|P61353|P46777|P05386|Q13772|P60866|P46776|P05387|P60228|P62249|P25398|P09669|O00483|P14854|P10176|P62891|P42766|Q02878|P84243|Q14192|P61247|P62857|P62979|Q15004|P62851|P08708|P62906|P18077|P15954|Q6P6C2|P17535|Q8N8D1|P42677|P15880|Q02543|P62263|P62987|Q14240|P62945|P62269|P20585</td></tr>
<tr><td>GO:0090407</td><td>organophosphate biosynthetic process</td><td>8.01E-05</td><td>5.35E-04</td><td>13</td><td>686</td><td>190</td><td>35980</td><td>O75947|P06732|P48047|P01160|P36542|P16860|P25705|P18859|Q9NPI5|P56381|Q8WYQ3|Q8N335|P06576</td></tr>
<tr><td>GO:0097031</td><td>mitochondrial respiratory chain complex I biogenesis</td><td>1.13E-12</td><td>1.88E-11</td><td>10</td><td>62</td><td>190</td><td>35980</td><td>O00217|O95169|P51970|O43674|O14561|O43920|O95167|O95178|O96000|O15239</td></tr>
<tr><td>GO:0097300</td><td>programmed necrotic cell death</td><td>4.34E-05</td><td>3.10E-04</td><td>4</td><td>37</td><td>190</td><td>35980</td><td>P0CG47|P62987|P62979|P0CG48</td></tr>
<tr><td>GO:0097305</td><td>response to alcohol</td><td>1.96E-04</td><td>1.17E-03</td><td>9</td><td>378</td><td>190</td><td>35980</td><td>P13639|P00505|O75116|P19021|P68032|P02511|P62158|P00441|P01034</td></tr>
<tr><td>GO:0097485</td><td>neuron projection guidance</td><td>4.57E-03</td><td>1.71E-02</td><td>9</td><td>595</td><td>190</td><td>35980</td><td>P0CG47|P60660|O75116|P62987|P83731|P62979|P62158|P19105|P0CG48</td></tr>
<tr><td>GO:0098542</td><td>defense response to other organism</td><td>8.90E-03</td><td>2.84E-02</td><td>8</td><td>547</td><td>190</td><td>35980</td><td>P52179|P62891|P40925|P62987|Q16654|Q6P6C2|P10176|Q01628</td></tr>
<tr><td>GO:0098655</td><td>cation transmembrane transport</td><td>7.73E-08</td><td>8.36E-07</td><td>22</td><td>1083</td><td>190</td><td>35980</td><td>O75947|P10606|P09669|P48047|P14406|O00483|P14854|P16615|P36542|P15954|P10176|P13073|P25705|P18859|O14949|P47985|P56381|P24310|P24311|P25116|P06576|P20674</td></tr>
<tr><td>GO:0098660</td><td>inorganic ion transmembrane transport</td><td>2.08E-08</td><td>2.47E-07</td><td>23</td><td>1095</td><td>190</td><td>35980</td><td>O75947|P10606|P09669|P48047|P14406|O00483|P14854|P16615|P36542|P15954|P10176|P13073|P25705|P18859|O14949|Q00325|P47985|P56381|P24310|P24311|P25116|P06576|P20674</td></tr>
<tr><td>GO:0098662</td><td>inorganic cation transmembrane transport</td><td>5.46E-09</td><td>7.02E-08</td><td>22</td><td>932</td><td>190</td><td>35980</td><td>O75947|P10606|P09669|P48047|P14406|O00483|P14854|P16615|P36542|P15954|P10176|P13073|P25705|P18859|O14949|P47985|P56381|P24310|P24311|P25116|P06576|P20674</td></tr>
<tr><td>GO:0098754</td><td>detoxification</td><td>1.44E-03</td><td>6.49E-03</td><td>5</td><td>155</td><td>190</td><td>35980</td><td>P68871|O14880|P22352|P69905|P00441</td></tr>
<tr><td>GO:0098771</td><td>inorganic ion homeostasis</td><td>4.62E-04</td><td>2.46E-03</td><td>11</td><td>615</td><td>190</td><td>35980</td><td>P13693|P02792|P51684|O14958|P19429|P16615|Q16654|P23327|P25116|P00441|P06576</td></tr>
<tr><td>GO:0098779</td><td>mitophagy in response to mitochondrial depolarization</td><td>5.83E-03</td><td>2.01E-02</td><td>4</td><td>135</td><td>190</td><td>35980</td><td>P52179|Q9P0U1|P40925|P10176</td></tr>
<tr><td>GO:0098780</td><td>response to mitochondrial depolarisation</td><td>5.83E-03</td><td>2.01E-02</td><td>4</td><td>135</td><td>190</td><td>35980</td><td>P52179|Q9P0U1|P40925|P10176</td></tr>
<tr><td>GO:0098792</td><td>xenophagy</td><td>1.33E-05</td><td>1.06E-04</td><td>6</td><td>97</td><td>190</td><td>35980</td><td>P52179|P40925|P62987|Q16654|Q6P6C2|P10176</td></tr>
<tr><td>GO:0098869</td><td>cellular oxidant detoxification</td><td>1.21E-03</td><td>5.67E-03</td><td>5</td><td>149</td><td>190</td><td>35980</td><td>P68871|O14880|P22352|P69905|P00441</td></tr>
<tr><td>GO:1900034</td><td>regulation of cellular response to heat</td><td>7.68E-03</td><td>2.52E-02</td><td>3</td><td>76</td><td>190</td><td>35980</td><td>P0DMV8|P0DMV9|P02511</td></tr>
<tr><td>GO:1900407</td><td>regulation of cellular response to oxidative stress</td><td>2.92E-04</td><td>1.65E-03</td><td>4</td><td>60</td><td>190</td><td>35980</td><td>P07602|P63244|P04792|P00441</td></tr>
<tr><td>GO:1900408</td><td>negative regulation of cellular response to oxidative</td><td>9.87E-04</td><td>4.75E-03</td><td>3</td><td>37</td><td>190</td><td>35980</td><td>P07602|P63244|P04792</td></tr>
<tr><td>GO:1901019</td><td>regulation of calcium ion transmembrane transporter</td><td>9.13E-04</td><td>4.44E-03</td><td>4</td><td>81</td><td>190</td><td>35980</td><td>O14958|P01160|P23327|P62158</td></tr>
<tr><td>GO:1901135</td><td>carbohydrate derivative metabolic process</td><td>3.37E-11</td><td>5.14E-10</td><td>34</td><td>1731</td><td>190</td><td>35980</td><td>P07602|P13929|O75947|P0DMV8|P0DMV9|P01160|O43674|O00483|P36542|P10176|O95178|O96000|P16860|P13073|P25705|P18859|P56381|Q8WYQ3|O15239|P20674|O00217|Q08493|O95169|P51970|P48047|P04406|O14561|O43920|P13533|O95167|P12883|P17081|Q8N335|P06576</td></tr>
<tr><td>GO:1901184</td><td>regulation of ERBB signaling pathway</td><td>1.09E-03</td><td>5.20E-03</td><td>4</td><td>85</td><td>190</td><td>35980</td><td>P0CG47|P62987|P62979|P0CG48</td></tr>
<tr><td>GO:1901185</td><td>negative regulation of ERBB signaling pathway</td><td>7.91E-05</td><td>5.31E-04</td><td>4</td><td>43</td><td>190</td><td>35980</td><td>P0CG47|P62987|P62979|P0CG48</td></tr>
<tr><td>GO:1901293</td><td>nucleoside phosphate biosynthetic process</td><td>3.25E-06</td><td>2.92E-05</td><td>11</td><td>354</td><td>190</td><td>35980</td><td>O75947|P16860|P25705|P48047|P18859|P01160|Q9NPI5|P36542|P56381|Q8WYQ3|P06576</td></tr>
<tr><td>GO:1901360</td><td>organic cyclic compound metabolic process</td><td>2.05E-23</td><td>6.35E-22</td><td>100</td><td>7222</td><td>190</td><td>35980</td><td>O75947|O43674|O96000|P16860|P13073|P07195|P46783|P83731|P62913|P62753|O15239|P20674|Q13765|O95169|P48047|P04406|O14561|Q9Y5N6|P16989|Q9Y6H1|O95167|P84098|P63173|P68104|P46777|Q13772|P60866|P46776|P60228|P62249|Q8N335|P25398|P10176|O95178|P62891|P25705|P18859|Q14192|P56381|O00217|Q08493|P08708|P18077|Q6P6C2|P17535|P12883|Q8N8D1|P42677|P15880|Q02543|P62263|Q14240|P62945|P62269|P0CG47|Q9NSD9|P0DMV8|P0DMV9|P01160|P40926|P40925|P62280|Q8TAK5|P36542|P0CG48|P39019|P00505|P39023|P62273|P24311|Q8WYQ3|Q07020|P10606|Q96H40|O43920|P61353|P05386|P17081|P05387|P13929|P09669|O00483|P14854|P42766|Q02878|P84243|Q9NPI5|P61247|P62857|P62979|Q15004|P62851|P51970|P62906|P15954|P51857|P13533|P62987|P20585|P06576</td></tr>
<tr><td>GO:1901361</td><td>organic cyclic compound catabolic process</td><td>1.35E-35</td><td>1.03E-33</td><td>41</td><td>527</td><td>190</td><td>35980</td><td>P25398|P62280|P39019|P62891|P42766|Q02878|P00505|P39023|P62273|P46783|P61247|P83731|P62857|P62913|P62979|P62851|P62753|Q07020|Q08493|P08708|P62906|P18077|P51857|P42677|P84098|P15880|P63173|Q02543|P61353|P62263|P62987|Q14240|P62945|P46777|P05386|P60866|P46776|P62269|P05387|P60228|P62249</td></tr>
</table>

GO:1901362	organic cyclic compound biosynthetic process	4.07E-19	9.46E-18	69	4141	190	35980	P0CG47|O75947|P01160|P62280|Q8TAK5|P36542|P0CG48|P39019|P16860|P13073|P39023|P62273|P46783|P83731|P62913|P24311|P62753|Q8WYQ3|P20674|Q13765|Q07020|P10606|Q96H40|P48047|P16989|Q9Y6H1|P84098|P63173|P68104|P61353|P46777|P05386|Q13772|P60866|P46776|P05387|P62249|P25398|P09669|O00483|P14854|P10176|P62891|P42766|Q02878|P25705|P18859|Q14192|Q9NPI5|P61247|P62857|P62979|P56381|Q15004|P62851|P08708|P62906|P18077|P15954|P51857|P17535|P42677|P15880|Q02543|P62263|P62987|P62945|P62269|P06576
GO:1901564	organonitrogen compound metabolic process	5.24E-49	9.74E-47	94	3102	190	35980	O75947|P06732|Q9NSD9|P0DMV8|P0DMV9|P01160|O43674|P40926|P40925|P62280|P36542|P39019|O96000|P16860|P13073|P00505|P39023|P62273|P07195|P46783|P83731|P62913|P62753|Q8WYQ3|P24752|O15239|P20674|Q13765|Q07020|O95169|P13639|P48047|P04406|O14561|O14880|P19021|P37108|O43920|O95167|P84098|Q05639|P63173|P68104|P61353|P46777|P05386|P17081|P60866|P46776|P05387|P60228|P62249|Q8N335|P07602|P13929|P25398|O00483|P10176|O95178|P12235|P62891|P42766|Q02878|P25705|P18859|Q9NPI5|P17174|P61247|P62857|P62979|P56381|P62851|P00441|P54368|O00217|Q08493|P51970|P08708|P62906|P18077|P13533|P12883|P42677|P15880|P17540|Q02543|Q9NWT8|Q00325|P62263|P62987|Q14240|P62945|P62269|P06576
GO:1901566	organonitrogen compound biosynthetic process	2.34E-33	1.42E-31	63	1893	190	35980	O75947|P06732|Q9NSD9|P01160|P62280|P36542|P39019|P16860|P00505|P39023|P62273|P46783|P83731|P62913|P62753|Q8WYQ3|Q13765|Q07020|P13639|P48047|O14880|P37108|P84098|Q05639|P63173|P68104|P61353|P46777|P05386|P60866|P46776|P05387|P60228|P62249|P25398|P12235|P62891|P42766|Q02878|P25705|P18859|Q9NPI5|P17174|P61247|P62857|P62979|P56381|P62851|P54368|P08708|P62906|P18077|P42677|P15880|Q02543|Q9NWT8|Q00325|P62263|P62987|Q14240|P62945|P62269|P06576
GO:1901575	organic substance catabolic process	4.99E-21	1.33E-19	51	2049	190	35980	P0CG47|P62280|P0CG48|P39019|P00505|P39023|P62273|P46783|P83731|P62913|P62158|P62753|P24752|Q07020|P04406|P84098|P63173|P61353|P46777|P05386|P60866|P46776|P05387|P60228|P62249|Q13011|Q8N335|P13929|P25398|P62891|P42766|Q02878|P17174|P61247|P62857|Q16698|P62979|P62851|Q08493|P08708|P62906|P18077|P51857|P42677|P15880|Q02543|P62263|P62987|Q14240|P62945|P62269
GO:1901576	organic substance biosynthetic process	2.09E-20	5.31E-19	92	6775	190	35980	P0CG47|O75947|P06732|Q9NSD9|P01160|P40926|P40925|P62280|Q8TAK5|P36542|P0CG48|P39019|P16860|P13073|P00505|P39023|P62273|P46783|P83731|P62913|P24311|P62753|Q8WYQ3|P24752|P20674|Q13765|Q07020|P10606|Q96H40|P13639|P48047|P04406|O14561|O14880|Q9Y5N6|P37108|P16989|Q9Y6H1|P84098|Q05639|P63173|P68104|P61353|P46777|P05386|Q13772|P60866|P46776|P05387|P60228|P62249|Q8N335|P13929|P25398|P09669|O00483|P14854|P10176|P12235|P62891|P42766|Q02878|P25705|P18859|Q14192|Q9NPI5|P17174|P61247|P62857|P62979|P56381|Q15004|P62851|P54368|P08708|P62906|P18077|P15954|P51857|P17535|P42677|P41222|P15880|Q02543|Q9NWT8|Q00325|P62263|P62987|Q14240|P62945|P62269|P06576
GO:1901605	alpha-amino acid metabolic process	7.25E-37	6.74E-35	40	454	190	35980	P06732|P25398|P62280|P39019|P62891|P42766|Q02878|P00505|P39023|P62273|P17174|P46783|P61247|P83731|P62857|P62913|P62979|P62851|P62753|Q07020|P08708|P62906|P18077|P42677|P84098|P15880|P17540|P63173|Q02543|P61353|P62263|P62987|P62945|P46777|P05386|P60866|P46776|P62269|P05387|P62249
GO:1901652	response to peptide	1.40E-06	1.35E-05	15	627	190	35980	P0CG47|P01033|P01160|P16615|P35613|P17535|P0CG48|P17174|P08574|P62987|Q16654|P62979|P62158|P17081|P62753
GO:1901653	cellular response to peptide	1.37E-03	6.26E-03	9	496	190	35980	P0CG47|P17174|P62987|Q16654|P62979|P62158|P17081|P62753|P0CG48
GO:1901657	glycosyl compound metabolic process	3.11E-20	7.78E-19	29	564	190	35980	P13929|O75947|P0DMV8|P0DMV9|O43674|O00483|P36542|P10176|O95178|O96000|P13073|P25705|P18859|P56381|Q8WYQ3|O15239|P20674|O00217|O95169|P51970|P48047|P04406|O14561|O43920|P13533|O95167|P12883|P17081|P06576
GO:1901659	glycosyl compound biosynthetic process	8.35E-06	6.93E-05	8	190	190	35980	O75947|P25705|P48047|P18859|P36542|P56381|Q8WYQ3|P06576
GO:1901698	response to nitrogen compound	6.90E-06	5.86E-05	19	1103	190	35980	P0CG47|P01033|P13639|P01160|O14880|P16615|P35613|P17535|P0CG48|O14958|P17174|P08574|P62987|Q16654|P62979|P62158|P17081|P62753|P00441
GO:1901699	cellular response to nitrogen compound	1.72E-03	7.52E-03	11	724	190	35980	P0CG47|O14958|P17174|P62987|Q16654|P62979|P62158|P17081|P62753|P00441|P0CG48
GO:1901700	response to oxygen-containing compound	2.74E-11	4.20E-10	33	1622	190	35980	P0CG47|P01160|P16615|P35613|P0CG48|P63244|P05090|P00505|P68871|P09493|P17174|Q16654|P22352|P62979|P69905|P62158|P62753|P00441|P01034|P01033|P02144|Q14116|P13639|O75116|P19021|P68032|P17535|P09382|P08574|P62987|P02511|P17081|P25116
GO:1901701	cellular response to oxygen-containing compound	1.15E-04	7.28E-04	16	1022	190	35980	P0CG47|Q14116|O75116|P0CG48|P63244|P09382|P09493|P17174|P62987|Q16654|P62979|P62158|P17081|P62753|P00441|P01034
GO:1902400	intracellular signal transduction involved in G1 DNA	4.45E-04	2.39E-03	4	67	190	35980	P0CG47|P62987|P62979|P0CG48
GO:1902402	signal transduction involved in mitotic DNA damage	4.71E-04	2.49E-03	4	68	190	35980	P0CG47|P62987|P62979|P0CG48
GO:1902403	signal transduction involved in mitotic DNA integrity	4.71E-04	2.49E-03	4	68	190	35980	P0CG47|P62987|P62979|P0CG48
GO:1902531	regulation of intracellular signal transduction	8.47E-05	5.54E-04	24	1924	190	35980	P07602|P0CG47|P51452|Q14116|P0DMV8|P0DMV9|P19022|P16989|Q8N3K9|P0CG48|P13693|P63244|O14958|P60033|P09382|P62987|P23327|P62979|P62158|P17081|P04792|P25116|Q8N335|P00441
GO:1902532	negative regulation of intracellular signal transduction	2.24E-04	1.30E-03	10	472	190	35980	P51452|P13693|P63244|O14958|P0DMV8|P16989|Q8N3K9|P62158|P04792|Q8N335
GO:1902533	positive regulation of intracellular signal transduction	5.14E-04	2.67E-03	15	1054	190	35980	P07602|P0CG47|Q14116|P0DMV8|P0DMV9|P19022|P0CG48|P63244|P60033|P09382|P62987|P62979|P62158|P25116|P00441
GO:1902578	single-organism localization	1.10E-24	4.00E-23	89	5534	190	35980	P0CG47|O75947|P0DMV8|P16615|P62280|P36542|P35613|P0CG48|P39019|P16860|P05090|P13073|P00505|O14949|P39023|P62273|P46783|P83731|P62913|P24310|P69905|P62158|P24311|P62753|P20674|Q07020|P01033|P10606|P48047|Q9Y277|P19021|P19022|P14406|P37108|Q9Y6H1|Q08431|P84098|O14958|P63173|P61353|P47985|P46777|P05386|P60866|P46776|P05387|P62249|P07602|P02792|P25398|P09669|P68363|O00483|P14854|P10176|P12235|P13693|P62891|P42766|Q02878|P25705|P18859|P60033|P68871|P61247|P62857|P62979|P56381|P62851|P00441|P02144|Q9P0U1|P08708|O75116|P62906|P18077|P15954|Q6P6C2|P42677|P15880|Q02543|Q00325|P62263|P62987|P62945|P04275|P62269|P25116|P06576
GO:1902579	multi-organism localization	3.89E-05	2.81E-04	4	36	190	35980	P0CG47|P62987|P62979|P0CG48
GO:1902580	single-organism cellular localization	1.56E-27	7.13E-26	44	1014	190	35980	P0CG47|P25398|P62280|P35613|P39019|P62891|P42766|Q02878|P39023|P62273|P46783|P61247|P83731|P62857|P62913|P62979|P62851|P62753|Q07020|Q9P0U1|P08708|O75116|P62906|P19022|P18077|P37108|Q9Y6H1|P42677|P84098|P15880|P63173|Q02543|P61353|P62263|P62987|P62945|P46777|P05386|P60866|P46776|P62269|P05387|P25116|P62249
GO:1902582	single-organism intracellular transport	7.99E-31	4.40E-29	56	1578	190	35980	P0CG47|O75947|P0DMV8|P16615|P62280|P36542|P35613|P0CG48|P39019|P39023|P62273|P46783|P83731|P62913|P62753|Q07020|P48047|P37108|Q9Y6H1|P84098|P63173|P61353|P46777|P05386|P60866|P46776|P05387|P62249|P02792|P25398|P68363|P62891|P42766|Q02878|P25705|P18859|P61247|P62857|P62979|P56381|P62851|P00441|Q9P0U1|P08708|P62906|P18077|Q6P6C2|P42677|P15880|Q02543|P62263|P62987|P62945|P62269|P25116|P06576
GO:1902583	multi-organism intracellular transport	2.41E-05	1.85E-04	4	32	190	35980	P0CG47|P62987|P62979|P0CG48
GO:1902589	single-organism organelle organization	4.88E-04	2.56E-03	31	3128	190	35980	P0CG47|P52179|P10916|P0DMV8|P40925|P68363|P10176|P0CG48|P09493|P83731|P62979|Q15004|P45379|P00441|O15273|Q14896|Q9P0U1|P04406|O75116|P68133|P17661|P68032|P13533|O14958|Q9NWT8|Q9Y3D2|P62987|P02511|P63313|P17081|P20585
GO:1902600	hydrogen ion transmembrane transport	1.97E-18	4.44E-17	20	236	190	35980	O75947|P10606|P09669|P48047|P14406|O00483|P14854|P36542|P15954|P10176|P13073|P25705|P18859|O14949|P47985|P56381|P24310|P24311|P06576|P20674
GO:1902680	positive regulation of RNA biosynthetic process	1.13E-02	3.36E-02	16	1603	190	35980	P0CG47|Q13765|P0DMV8|P0DMV9|Q969T9|Q8TAK5|Q9Y6H1|P17535|P0CG48|P60033|Q14192|P62987|P62979|Q13772|P17081|P25116
GO:1902806	regulation of cell cycle G1/S phase transition	1.31E-02	3.82E-02	4	171	190	35980	P0CG47|P62987|P62979|P0CG48
GO:1902807	negative regulation of cell cycle G1/S phase transition	3.96E-03	1.51E-02	4	121	190	35980	P0CG47|P62987|P62979|P0CG48
GO:1902882	regulation of response to oxidative stress	4.20E-04	2.27E-03	4	66	190	35980	P07602|P63244|P04792|P00441
GO:1902883	negative regulation of response to oxidative stress	9.87E-04	4.75E-03	3	37	190	35980	P07602|P63244|P04792
GO:1903008	organelle disassembly	1.26E-05	1.01E-04	8	201	190	35980	P0CG47|P52179|Q9P0U1|P40925|P62987|P62979|P10176|P0CG48
GO:1903050	regulation of proteolysis involved in cellular protein	1.50E-02	4.29E-02	5	272	190	35980	P0CG47|P63244|P62987|P62979|P0CG48
GO:1903052	positive regulation of proteolysis involved in cellular	2.15E-03	9.05E-03	5	170	190	35980	P0CG47|P63244|P62987|P62979|P0CG48
GO:1903115	regulation of actin filament-based movement	2.12E-05	1.65E-04	4	31	190	35980	Q14896|P16615|P45379|P63316
GO:1903169	regulation of calcium ion transmembrane transport	7.25E-04	3.65E-03	5	133	190	35980	O14958|P01160|P23327|P62158|P25116
GO:1903201	regulation of oxidative stress-induced cell death	1.13E-04	7.13E-04	4	47	190	35980	P07602|P63244|P04792|P00441
GO:1903202	negative regulation of oxidative stress-induced cell death	7.69E-04	3.84E-03	3	34	190	35980	P07602|P63244|P04792
GO:1903320	regulation of protein modification by small protein	7.19E-03	2.39E-02	6	319	190	35980	P0CG47|P0DMV8|P0DMV9|P62987|P62979|P0CG48
GO:1903321	negative regulation of protein modification by small protein conjugation or removal	2.47E-04	1.42E-03	6	164	190	35980	P0CG47|P0DMV8|P0DMV9|P62987|P62979|P0CG48

<table>
<tr><td>GO:1903362</td><td>regulation of cellular protein catabolic process</td><td>4.80E-03</td><td>1.79E-02</td><td>6</td><td>293</td><td>190</td><td>35980</td><td>P0CG47|P63244|P60033|P62987|P62979|P0CG48</td></tr>
<tr><td>GO:1903364</td><td>positive regulation of cellular protein catabolic process</td><td>4.70E-04</td><td>2.49E-03</td><td>6</td><td>185</td><td>190</td><td>35980</td><td>P0CG47|P63244|P60033|P62987|P62979|P0CG48</td></tr>
<tr><td>GO:1903508</td><td>positive regulation of nucleic acid-templated transcription</td><td>9.65E-03</td><td>3.05E-02</td><td>16</td><td>1575</td><td>190</td><td>35980</td><td>P0CG47|Q13765|P0DMV8|P0DMV9|Q969T9|Q8TAK5|Q9Y6H1|P17535|P0CG48|P60033|Q14192|P62987|P62979|Q13772|P17081|P25116</td></tr>
<tr><td>GO:1903522</td><td>regulation of blood circulation</td><td>1.84E-13</td><td>3.31E-12</td><td>18</td><td>326</td><td>190</td><td>35980</td><td>Q14896|P10916|P01160|P16615|P17661|P13533|P12883|P16860|O14958|P19429|Q9UBY9|P08590|P09493|P23327|P62158|P45379|P25116|Q8N335</td></tr>
<tr><td>GO:1903524</td><td>positive regulation of blood circulation</td><td>1.69E-04</td><td>1.02E-03</td><td>5</td><td>97</td><td>190</td><td>35980</td><td>P01160|P09493|P16615|P23327|P25116</td></tr>
<tr><td>GO:1903649</td><td>regulation of cytoplasmic transport</td><td>3.32E-03</td><td>1.30E-02</td><td>8</td><td>462</td><td>190</td><td>35980</td><td>Q14116|P63244|P05090|O14958|Q9P0U1|P23327|P62158|P25116</td></tr>
<tr><td>GO:1903651</td><td>positive regulation of cytoplasmic transport</td><td>1.50E-02</td><td>4.29E-02</td><td>5</td><td>272</td><td>190</td><td>35980</td><td>Q14116|P63244|Q9P0U1|P62158|P25116</td></tr>
<tr><td>GO:1903779</td><td>regulation of cardiac conduction</td><td>1.67E-06</td><td>1.57E-05</td><td>6</td><td>68</td><td>190</td><td>35980</td><td>O14958|P19429|P01160|P16615|P23327|P62158</td></tr>
<tr><td>GO:1903827</td><td>regulation of cellular protein localization</td><td>3.12E-03</td><td>1.23E-02</td><td>9</td><td>561</td><td>190</td><td>35980</td><td>P54368|Q14116|P63244|P05090|Q9P0U1|Q02818|P19022|P17081|Q8N335</td></tr>
<tr><td>GO:1903844</td><td>regulation of cellular response to transforming growth</td><td>3.41E-03</td><td>1.33E-02</td><td>4</td><td>116</td><td>190</td><td>35980</td><td>P0CG47|P62987|P62979|P0CG48</td></tr>
<tr><td>GO:1903845</td><td>negative regulation of cellular response to transforming growth factor beta stimulus</td><td>9.56E-04</td><td>4.63E-03</td><td>4</td><td>82</td><td>190</td><td>35980</td><td>P0CG47|P62987|P62979|P0CG48</td></tr>
<tr><td>GO:1904062</td><td>regulation of cation transmembrane transport</td><td>1.45E-03</td><td>6.56E-03</td><td>6</td><td>230</td><td>190</td><td>35980</td><td>O14958|P01160|P23327|P62158|P25116|Q8N335</td></tr>
<tr><td>GO:1990267</td><td>response to transition metal nanoparticle</td><td>1.61E-03</td><td>7.10E-03</td><td>5</td><td>159</td><td>190</td><td>35980</td><td>P80297|P19021|Q02818|P35613|P00441</td></tr>
<tr><td>GO:1990542</td><td>mitochondrial transmembrane transport</td><td>1.79E-09</td><td>2.44E-08</td><td>8</td><td>64</td><td>190</td><td>35980</td><td>O75947|P25705|Q9P0U1|P48047|P18859|P36542|P56381|P06576</td></tr>
<tr><td>GO:1990748</td><td>cellular detoxification</td><td>1.32E-03</td><td>6.07E-03</td><td>5</td><td>152</td><td>190</td><td>35980</td><td>P68871|O14880|P22352|P69905|P00441</td></tr>
<tr><td>GO:2000021</td><td>regulation of ion homeostasis</td><td>1.38E-04</td><td>8.56E-04</td><td>7</td><td>211</td><td>190</td><td>35980</td><td>P63244|P16860|O14958|P23327|P62158|P25116|Q8N335</td></tr>
<tr><td>GO:2000045</td><td>regulation of G1/S transition of mitotic cell cycle</td><td>1.07E-02</td><td>3.23E-02</td><td>4</td><td>161</td><td>190</td><td>35980</td><td>P0CG47|P62987|P62979|P0CG48</td></tr>
<tr><td>GO:2000058</td><td>regulation of protein ubiquitination involved in ubiquitin-dependent protein catabolic process</td><td>1.78E-03</td><td>7.70E-03</td><td>4</td><td>97</td><td>190</td><td>35980</td><td>P0CG47|P62987|P62979|P0CG48</td></tr>
<tr><td>GO:2000060</td><td>positive regulation of protein ubiquitination involved in ubiquitin-dependent protein catabolic process</td><td>1.24E-03</td><td>5.79E-03</td><td>4</td><td>88</td><td>190</td><td>35980</td><td>P0CG47|P62987|P62979|P0CG48</td></tr>
<tr><td>GO:2000134</td><td>negative regulation of G1/S transition of mitotic cell cycle</td><td>3.51E-03</td><td>1.36E-02</td><td>4</td><td>117</td><td>190</td><td>35980</td><td>P0CG47|P62987|P62979|P0CG48</td></tr>
<tr><td>GO:2001233</td><td>regulation of apoptotic signaling pathway</td><td>3.09E-04</td><td>1.74E-03</td><td>9</td><td>402</td><td>190</td><td>35980</td><td>P0CG47|P13693|P63244|P0DMV8|P0DMV9|P16989|P04792|P00441|Q8N8D1</td></tr>
<tr><td>GO:2001234</td><td>negative regulation of apoptotic signaling pathway</td><td>5.83E-03</td><td>2.01E-02</td><td>5</td><td>215</td><td>190</td><td>35980</td><td>P13693|P0DMV8|P0DMV9|P16989|P04792</td></tr>
<tr><td>GO:2001242</td><td>regulation of intrinsic apoptotic signaling pathway</td><td>1.97E-05</td><td>1.54E-04</td><td>7</td><td>155</td><td>190</td><td>35980</td><td>P0CG47|P13693|P63244|P0DMV8|P16989|P04792|P00441</td></tr>
<tr><td>GO:2001243</td><td>negative regulation of intrinsic apoptotic signaling</td><td>1.65E-03</td><td>7.26E-03</td><td>4</td><td>95</td><td>190</td><td>35980</td><td>P13693|P0DMV8|P16989|P04792</td></tr>
<tr><td>GO:2001244</td><td>positive regulation of intrinsic apoptotic signaling pathway</td><td>3.79E-03</td><td>1.45E-02</td><td>3</td><td>59</td><td>190</td><td>35980</td><td>P0CG47|P63244|P00441</td></tr>
<tr><td>GO:2001257</td><td>regulation of cation channel activity</td><td>2.14E-03</td><td>9.02E-03</td><td>4</td><td>102</td><td>190</td><td>35980</td><td>O14958|P01160|P23327|P62158</td></tr>
</table>

Supplemental Table S5C. hA-VCMs BinGO 2011. BinGO analysis of the adult ventricular cardiomyocyte (hA-VCMs) data[19] using the 2011 GO annotation dataset. **x** is the number of IDs in both the submitted list and associated with the GO term, **n** indicates the number of protein IDs associated with the GO term, **X** is the number of protein IDs submitted for analysis, **N** is the number of protein IDs used as the background set.

<table>
<tr><th>GO-ID</th><th>Description</th><th>p-value</th><th>corr p-value</th><th>x</th><th>n</th><th>X</th><th>N</th><th>Proteins submitted in analysis that are associated with the GO term</th></tr>
<tr><td>GO:0000028</td><td>ribosomal small subunit assembly</td><td>5.69E-08</td><td>8.67E-07</td><td>4</td><td>7</td><td>178</td><td>27688</td><td>P08708|P62263|P62851|P62753</td></tr>
<tr><td>GO:0000075</td><td>cell cycle checkpoint</td><td>5.93E-03</td><td>2.83E-02</td><td>6</td><td>252</td><td>178</td><td>27688</td><td>P0CG47|P62987|Q9Y5N6|P83731|P62979|P0CG48</td></tr>
<tr><td>GO:0000082</td><td>G1/S transition of mitotic cell cycle</td><td>4.58E-03</td><td>2.28E-02</td><td>5</td><td>167</td><td>178</td><td>27688</td><td>P0CG47|P62987|Q9Y5N6|P62979|P0CG48</td></tr>
<tr><td>GO:0000084</td><td>mitotic S phase</td><td>1.13E-03</td><td>7.77E-03</td><td>5</td><td>121</td><td>178</td><td>27688</td><td>P0CG47|P62987|Q9Y5N6|P62979|P0CG48</td></tr>
<tr><td>GO:0000165</td><td>MAPK cascade</td><td>9.25E-03</td><td>3.89E-02</td><td>5</td><td>198</td><td>178</td><td>27688</td><td>P0CG47|P51452|P62987|P62979|P0CG48</td></tr>
<tr><td>GO:0000187</td><td>activation of MAPK activity</td><td>3.35E-04</td><td>2.84E-03</td><td>6</td><td>143</td><td>178</td><td>27688</td><td>P0CG47|P60033|P62987|P62979|P00441|P0CG48</td></tr>
<tr><td>GO:0000302</td><td>response to reactive oxygen species</td><td>7.31E-05</td><td>7.33E-04</td><td>7</td><td>157</td><td>178</td><td>27688</td><td>P02144|Q14116|P68871|P09493|P69905|P00441|P12883</td></tr>
<tr><td>GO:0001817</td><td>regulation of cytokine production</td><td>4.05E-03</td><td>2.09E-02</td><td>8</td><td>393</td><td>178</td><td>27688</td><td>P0CG47|Q14116|P62987|P62979|P04792|P25116|P00441|P0CG48</td></tr>
<tr><td>GO:0002027</td><td>regulation of heart rate</td><td>2.01E-03</td><td>1.19E-02</td><td>3</td><td>39</td><td>178</td><td>27688</td><td>P09493|P13533|P12883</td></tr>
<tr><td>GO:0002218</td><td>activation of innate immune response</td><td>1.66E-03</td><td>1.03E-02</td><td>5</td><td>132</td><td>178</td><td>27688</td><td>P0CG47|P51452|P62987|P62979|P0CG48</td></tr>
<tr><td>GO:0002221</td><td>pattern recognition receptor signaling pathway</td><td>5.71E-04</td><td>4.50E-03</td><td>5</td><td>104</td><td>178</td><td>27688</td><td>P0CG47|P51452|P62987|P62979|P0CG48</td></tr>
<tr><td>GO:0002224</td><td>toll-like receptor signaling pathway</td><td>3.59E-04</td><td>3.02E-03</td><td>5</td><td>94</td><td>178</td><td>27688</td><td>P0CG47|P51452|P62987|P62979|P0CG48</td></tr>
<tr><td>GO:0002262</td><td>myeloid cell homeostasis</td><td>3.31E-05</td><td>3.53E-04</td><td>6</td><td>94</td><td>178</td><td>27688</td><td>P01033|P02144|P08708|P62263|P00441|P39019</td></tr>
<tr><td>GO:0002576</td><td>platelet degranulation</td><td>1.79E-04</td><td>1.61E-03</td><td>5</td><td>81</td><td>178</td><td>27688</td><td>P07602|P01033|P04275|P62158|P00441</td></tr>
<tr><td>GO:0002753</td><td>cytoplasmic pattern recognition receptor signaling</td><td>2.58E-05</td><td>2.84E-04</td><td>4</td><td>27</td><td>178</td><td>27688</td><td>P0CG47|P62987|P62979|P0CG48</td></tr>
<tr><td>GO:0002755</td><td>MyD88-dependent toll-like receptor signaling</td><td>1.09E-04</td><td>1.06E-03</td><td>5</td><td>73</td><td>178</td><td>27688</td><td>P0CG47|P51452|P62987|P62979|P0CG48</td></tr>
<tr><td>GO:0002756</td><td>MyD88-independent toll-like receptor signaling</td><td>6.27E-05</td><td>6.35E-04</td><td>5</td><td>65</td><td>178</td><td>27688</td><td>P0CG47|P51452|P62987|P62979|P0CG48</td></tr>
<tr><td>GO:0002758</td><td>innate immune response-activating signal</td><td>7.37E-04</td><td>5.59E-03</td><td>5</td><td>110</td><td>178</td><td>27688</td><td>P0CG47|P51452|P62987|P62979|P0CG48</td></tr>
<tr><td>GO:0003007</td><td>heart morphogenesis</td><td>2.38E-09</td><td>4.02E-08</td><td>12</td><td>181</td><td>178</td><td>27688</td><td>O15273|Q14896|P10916|P19429|Q14192|P08590|P09493|P68032|P45379|P13533|P12883|P63316</td></tr>
<tr><td>GO:0003008</td><td>system process</td><td>1.08E-09</td><td>1.94E-08</td><td>30</td><td>1297</td><td>178</td><td>27688</td><td>P52179|P10916|P01160|P54296|P16860|Q01449|P19429|P68871|P09493|P23327|P62158|P45379|P00441|P02144|Q14116|O15273|Q14896|O75116|P68133|P17661|P68032|P13533|P12883|O14958|P17540|P60660|P08590|P02511|P25116|P63316</td></tr>
<tr><td>GO:0003012</td><td>muscle system process</td><td>2.35E-23</td><td>1.38E-21</td><td>25</td><td>233</td><td>178</td><td>27688</td><td>P52179|P10916|P54296|Q01449|P19429|P09493|P23327|P62158|P45379|P00441|P02144|O15273|Q14896|O75116|P68133|P17661|P68032|P13533|P12883|O14958|P17540|P60660|P08590|P02511|P63316</td></tr>
<tr><td>GO:0003013</td><td>circulatory system process</td><td>2.76E-09</td><td>4.59E-08</td><td>15</td><td>320</td><td>178</td><td>27688</td><td>Q14116|O15273|Q14896|P10916|P01160|P68032|P13533|P16860|P19429|P68871|P08590|P09493|P25116|P00441|P63316</td></tr>
<tr><td>GO:0003015</td><td>heart process</td><td>2.75E-12</td><td>5.64E-11</td><td>9</td><td>39</td><td>178</td><td>27688</td><td>O15273|Q14896|P10916|P19429|P08590|P09493|P68032|P00441|P63316</td></tr>
<tr><td>GO:0003018</td><td>vascular process in circulatory system</td><td>6.07E-04</td><td>4.74E-03</td><td>6</td><td>160</td><td>178</td><td>27688</td><td>Q14116|P16860|P01160|P68871|P25116|P00441</td></tr>
<tr><td>GO:0003205</td><td>cardiac chamber development</td><td>1.08E-09</td><td>1.94E-08</td><td>10</td><td>101</td><td>178</td><td>27688</td><td>Q14896|P10916|P19429|Q14192|P08590|P09493|P45379|P13533|P12883|P63316</td></tr>
<tr><td>GO:0003206</td><td>cardiac chamber morphogenesis</td><td>4.77E-10</td><td>8.79E-09</td><td>10</td><td>93</td><td>178</td><td>27688</td><td>Q14896|P10916|P19429|Q14192|P08590|P09493|P45379|P13533|P12883|P63316</td></tr>
<tr><td>GO:0003208</td><td>cardiac ventricle morphogenesis</td><td>1.99E-10</td><td>3.80E-09</td><td>9</td><td>61</td><td>178</td><td>27688</td><td>Q14896|P10916|P19429|P08590|P09493|P45379|P13533|P12883|P63316</td></tr>
<tr><td>GO:0003229</td><td>ventricular cardiac muscle tissue development</td><td>1.11E-11</td><td>2.22E-10</td><td>9</td><td>45</td><td>178</td><td>27688</td><td>Q14896|P10916|P19429|P08590|P09493|P45379|P13533|P12883|P63316</td></tr>
<tr><td>GO:0003231</td><td>cardiac ventricle development</td><td>3.34E-09</td><td>5.52E-08</td><td>9</td><td>83</td><td>178</td><td>27688</td><td>Q14896|P10916|P19429|P08590|P09493|P45379|P13533|P12883|P63316</td></tr>
<tr><td>GO:0005996</td><td>monosaccharide metabolic process</td><td>3.09E-04</td><td>2.63E-03</td><td>8</td><td>262</td><td>178</td><td>27688</td><td>P13929|P00505|P40926|P04406|P40925|P17174|Q16654|P62158</td></tr>
<tr><td>GO:0006006</td><td>glucose metabolic process</td><td>1.38E-05</td><td>1.58E-04</td><td>8</td><td>168</td><td>178</td><td>27688</td><td>P13929|P00505|P40926|P04406|P40925|P17174|Q16654|P62158</td></tr>
<tr><td>GO:0006082</td><td>organic acid metabolic process</td><td>8.24E-04</td><td>6.19E-03</td><td>20</td><td>1417</td><td>178</td><td>27688</td><td>P13929|P06732|Q9NSD9|P40926|P04406|O14561|P40925|O14880|P51857|P35613|P41222|P17540|P00505|P07195|P17174|Q16654|Q16698|P24752|Q13011|P48735</td></tr>
<tr><td>GO:0006090</td><td>pyruvate metabolic process</td><td>2.29E-03</td><td>1.31E-02</td><td>5</td><td>142</td><td>178</td><td>27688</td><td>P13929|P04406|P07195|Q16654|P35613</td></tr>
<tr><td>GO:0006091</td><td>generation of precursor metabolites and energy</td><td>2.08E-30</td><td>1.89E-28</td><td>40</td><td>552</td><td>178</td><td>27688</td><td>P13929|O75947|P09669|O43674|P40926|P40925|O00483|P14854|P36542|P10176|O95178|P12235|O96000|P13073|P25705|P18859|O14949|P07195|P56381|P24310|P62158|P24311|O15239|P20674|O00217|P10606|O95169|P51970|P48047|P04406|O14561|O43920|P15954|O95167|Q00325|P08574|P47985|Q13011|P06576|P48735</td></tr>
<tr><td>GO:0006094</td><td>gluconeogenesis</td><td>1.79E-06</td><td>2.40E-05</td><td>6</td><td>57</td><td>178</td><td>27688</td><td>P13929|P00505|P40926|P04406|P40925|P17174</td></tr>
<tr><td>GO:0006099</td><td>tricarboxylic acid cycle</td><td>6.52E-03</td><td>2.87E-02</td><td>3</td><td>59</td><td>178</td><td>27688</td><td>P40926|P40925|P48735</td></tr>
<tr><td>GO:0006101</td><td>citrate metabolic process</td><td>8.17E-03</td><td>3.49E-02</td><td>3</td><td>64</td><td>178</td><td>27688</td><td>P40926|P40925|P48735</td></tr>
<tr><td>GO:0006103</td><td>2-oxoglutarate metabolic process</td><td>4.79E-04</td><td>3.87E-03</td><td>3</td><td>24</td><td>178</td><td>27688</td><td>P00505|P17174|P48735</td></tr>
<tr><td>GO:0006107</td><td>oxaloacetate metabolic process</td><td>1.12E-04</td><td>1.08E-03</td><td>3</td><td>15</td><td>178</td><td>27688</td><td>P00505|P40926|P40925</td></tr>
<tr><td>GO:0006119</td><td>oxidative phosphorylation</td><td>2.59E-13</td><td>6.19E-12</td><td>12</td><td>84</td><td>178</td><td>27688</td><td>O00217|O95169|P51970|O43674|O14561|O00483|O43920|P36542|O95167|O95178|O96000|O15239</td></tr>
<tr><td>GO:0006120</td><td>mitochondrial electron transport, NADH to ubiquinone</td><td>1.34E-14</td><td>3.69E-13</td><td>11</td><td>49</td><td>178</td><td>27688</td><td>O00217|O95169|P51970|O43674|O14561|O00483|O43920|O95167|O95178|O96000|O15239</td></tr>
<tr><td>GO:0006139</td><td>nucleobase-containing compound metabolic process</td><td>1.07E-21</td><td>5.27E-20</td><td>75</td><td>3654</td><td>178</td><td>27688</td><td>P0CG47|O75947|Q9NSD9|P01160|O43674|P40926|P40925|P16615|P62280|P36542|P0CG48|P39019|O96000|P16860|P39023|P62273|P07195|P46783|P83731|P62913|P62753|O15239|Q13765|Q07020|O95169|P48047|P04406|O14561|Q9Y5N6|O43920|O95167|P84098|P63173|P61353|P46777|P05386|P60866|P46776|P05387|P60228|P62249|P13929|P25398|O00483|O95178|P62891|P42766|Q02878|P25705|P18859|Q9NPI5|P61247|P62857|P62979|P56381|P62851|P00441|O00217|Q08493|P51970|P08708|P62906|P18077|Q8N8D1|P42677|P15880|Q02543|P62263|P62987|Q14240|P62945|P04792|P62269|P20585|P06576</td></tr>
<tr><td>GO:0006163</td><td>purine nucleotide metabolic process</td><td>6.01E-15</td><td>1.79E-13</td><td>25</td><td>522</td><td>178</td><td>27688</td><td>P13929|O75947|P01160|O43674|O00483|P16615|P36542|O95178|O96000|P16860|P25705|P18859|P07195|P56381|O15239|O00217|Q08493|O95169|P51970|P48047|P04406|O14561|O43920|O95167|P06576</td></tr>
<tr><td>GO:0006164</td><td>purine nucleotide biosynthetic process</td><td>2.80E-06</td><td>3.61E-05</td><td>10</td><td>232</td><td>178</td><td>27688</td><td>O75947|P16860|P25705|P48047|P18859|P01160|P16615|P36542|P56381|P06576</td></tr>
<tr><td>GO:0006364</td><td>rRNA processing</td><td>9.33E-08</td><td>1.36E-06</td><td>9</td><td>121</td><td>178</td><td>27688</td><td>P08708|P62263|P18077|P62857|P62913|P46777|P62753|P62249|P39019</td></tr>
<tr><td>GO:0006412</td><td>translation</td><td>3.88E-31</td><td>4.02E-29</td><td>43</td><td>648</td><td>178</td><td>27688</td><td>P25398|Q9NSD9|P62280|P39019|P62891|P42766|Q02878|P39023|P62273|P46783|P61247|P83731|P62857|P62913|P62979|P62851|P62753|Q13765|Q07020|P13639|P08708|P62906|P18077|P42677|P84098|P15880|Q05639|P63173|P68104|Q02543|P61353|P62263|P62987|Q14240|P62945|P46777|P05386|P60866|P46776|P62269|P05387|P60228|P62249</td></tr>
<tr><td>GO:0006413</td><td>translational initiation</td><td>2.58E-04</td><td>2.23E-03</td><td>4</td><td>48</td><td>178</td><td>27688</td><td>P08708|Q14240|P61247|P60228</td></tr>
<tr><td>GO:0006414</td><td>translational elongation</td><td>2.57E-56</td><td>1.40E-53</td><td>38</td><td>110</td><td>178</td><td>27688</td><td>P25398|P62280|P39019|P62891|P42766|Q02878|P39023|P62273|P46783|P61247|P83731|P62857|P62913|P62979|P62851|P62753|Q07020|P13639|P08708|P62906|P18077|P42677|P84098|P15880|P63173|P68104|Q02543|P61353|P62263|P62987|P62945|P46777|P05386|P60866|P46776|P62269|P05387|P62249</td></tr>
<tr><td>GO:0006415</td><td>translational termination</td><td>4.21E-55</td><td>1.83E-52</td><td>36</td><td>95</td><td>178</td><td>27688</td><td>P25398|P62280|P39019|P62891|P42766|Q02878|P39023|P62273|P46783|P61247|P83731|P62857|P62913|P62979|P62851|P62753|Q07020|P08708|P62906|P18077|P42677|P84098|P15880|P63173|Q02543|P61353|P62263|P62987|P62945|P46777|P05386|P60866|P46776|P62269|P05387|P62249</td></tr>
<tr><td>GO:0006417</td><td>regulation of translation</td><td>3.36E-03</td><td>1.79E-02</td><td>6</td><td>224</td><td>178</td><td>27688</td><td>P63244|P62263|Q14240|P37108|P04792|P60228</td></tr>
<tr><td>GO:0006446</td><td>regulation of translational initiation</td><td>4.83E-03</td><td>2.38E-02</td><td>3</td><td>53</td><td>178</td><td>27688</td><td>Q14240|P04792|P60228</td></tr>
<tr><td>GO:0006518</td><td>peptide metabolic process</td><td>6.77E-31</td><td>6.41E-29</td><td>46</td><td>789</td><td>178</td><td>27688</td><td>P25398|Q9NSD9|P62280|P39019|P62891|P42766|Q02878|P39023|P62273|P46783|P61247|P83731|P62857|P62913|P22352|P62979|P62851|P62753|P00441|Q13765|Q07020|P13639|P08708|P62906|P19021|P18077|P42677|P84098|P15880|Q05639|P63173|P68104|Q02543|P61353|P62263|P62987|Q14240|P62945|P46777|P05386|P60866|P46776|P62269|P05387|P60228|P62249</td></tr>
<tr><td>GO:0006725</td><td>cellular aromatic compound metabolic process</td><td>9.69E-20</td><td>3.98E-18</td><td>75</td><td>3941</td><td>178</td><td>27688</td><td>P0CG47|O75947|Q9NSD9|P01160|O43674|P40926|P40925|P16615|P62280|P36542|P0CG48|P39019|O96000|P16860|P39023|P62273|P07195|P46783|P83731|P62913|P62753|O15239|Q13765|Q07020|O95169|P48047|P04406|O14561|Q9Y5N6|O43920|O95167|P84098|P63173|P61353|P46777|P05386|P60866|P46776|P05387|P60228|P62249|P13929|P25398|O00483|O95178|P62891|P42766|Q02878|P25705|P18859|Q9NPI5|P61247|P62857|P62979|P56381|P62851|P00441|O00217|Q08493|P51970|P08708|P62906|P18077|Q8N8D1|P42677|P15880|Q02543|P62263|P62987|Q14240|P62945|P04792|P62269|P20585|P06576</td></tr>
<tr><td>GO:0006733</td><td>oxidoreduction coenzyme metabolic process</td><td>2.17E-03</td><td>1.26E-02</td><td>6</td><td>205</td><td>178</td><td>27688</td><td>P13929|P40926|P04406|Q9NPI5|P07195|P40925</td></tr>
</table>

GO:0006753	nucleoside phosphate metabolic process	5.62E-14	1.46E-12	28	747	178	27688	P13929|O75947|P01160|O43674|P40926|P40925|O00483|P16615|P36542|O95178|O96000|P16860|P25705|P18859|Q9NPI5|P07195|P56381|O15239|O00217|Q08493|O95169|P51970|P48047|P04406|O14561|O43920|O95167|P06576
GO:0006754	ATP biosynthetic process	5.08E-07	7.08E-06	8	108	178	27688	O75947|P25705|P48047|P18859|P16615|P36542|P56381|P06576
GO:0006793	phosphorus metabolic process	8.59E-09	1.37E-07	39	2244	178	27688	P0CG47|P13929|O75947|P06732|P01160|O43674|P40926|P40925|O00483|P16615|P36542|O95178|P0CG48|O96000|P16860|P25705|P18859|P60033|Q9NPI5|P07195|Q16654|P62979|P56381|O15239|P51452|O00217|Q08493|O95169|P51970|P48047|P04406|O14561|O75116|O43920|O95167|P62987|P25116|Q8N335|P06576
GO:0006796	phosphate-containing compound metabolic process	3.96E-09	6.43E-08	39	2182	178	27688	P0CG47|P13929|O75947|P06732|P01160|O43674|P40926|P40925|O00483|P16615|P36542|O95178|P0CG48|O96000|P16860|P25705|P18859|P60033|Q9NPI5|P07195|Q16654|P62979|P56381|O15239|P51452|O00217|Q08493|O95169|P51970|P48047|P04406|O14561|O75116|O43920|O95167|P62987|P25116|Q8N335|P06576
GO:0006807	nitrogen compound metabolic process	9.93E-21	4.60E-19	88	5152	178	27688	P0CG47|O75947|P06732|Q9NSD9|P01160|O43674|P40926|P40925|P16615|P62280|P36542|P0CG48|P39019|O96000|P16860|P00505|P39023|P62273|P07195|P46783|P83731|P62913|P62158|P62753|P24752|O15239|Q13765|Q07020|O95169|P13639|P48047|P04406|O14561|P19021|Q9Y5N6|O43920|O95167|P84098|Q05639|P63173|P68104|P61353|P46777|P05386|P60866|P46776|P05387|P60228|P62249|P07602|P13929|P25398|O00483|O95178|P62891|P42766|Q02878|P25705|P18859|Q9NPI5|P17174|P61247|P62857|P22352|P62979|P56381|P62851|P00441|P54368|O00217|Q08493|P51970|P08708|P62906|P18077|Q8N8D1|P42677|P15880|P17540|Q02543|P62263|P62987|Q14240|P62945|P04792|P62269|P20585|P06576
GO:0006810	transport	1.02E-04	1.00E-03	53	5023	178	27688	P0CG47|O75947|O43674|P16615|P36542|P35613|P0CG48|P39019|O96000|P16860|P05090|P00505|O14949|P62913|P69905|P62158|O15239|Q13765|P01033|O95169|P48047|Q9Y277|O14561|P37108|O43920|O95167|Q08431|P19105|P08574|P47985|P07602|P02792|P68363|O00483|O95178|P12235|P13693|P25705|P18859|P68871|P62979|P56381|P00441|O00217|P02144|P51970|Q9P0U1|P41222|Q00325|P62987|P04275|P25116|P06576
GO:0006818	hydrogen transport	4.23E-05	4.44E-04	7	144	178	27688	O75947|P25705|P48047|P18859|P36542|P56381|P06576
GO:0006839	mitochondrial transport	1.74E-06	2.34E-05	8	127	178	27688	O75947|P25705|Q9P0U1|P48047|P18859|P36542|P56381|P06576
GO:0006928	movement of cell or subcellular component	3.21E-05	3.44E-04	23	1392	178	27688	O15273|Q14896|P10916|P51684|O75116|P68363|P19022|P68133|P17661|P68032|P13533|P35613|P12883|Q01449|P19429|P60660|P08590|P09493|P83731|P04792|P45379|P06576|P63316
GO:0006936	muscle contraction	2.25E-23	1.36E-21	24	205	178	27688	P52179|P02144|O15273|Q14896|P10916|P54296|O75116|P68133|P17661|P68032|P13533|P12883|Q01449|O14958|P19429|P17540|P60660|P08590|P09493|P23327|P02511|P62158|P45379|P63316
GO:0006937	regulation of muscle contraction	1.47E-08	2.31E-07	9	98	178	27688	Q14896|P10916|P19429|P08590|P09493|P25116|O14558|P00441|P63316
GO:0006940	regulation of smooth muscle contraction	3.87E-03	2.02E-02	3	49	178	27688	P19429|P25116|P00441
GO:0006941	striated muscle contraction	6.35E-12	1.28E-10	10	61	178	27688	P02144|O15273|Q14896|O14958|P19429|P08590|P09493|P68032|P13533|P63316
GO:0006942	regulation of striated muscle contraction	1.87E-03	1.14E-02	3	38	178	27688	Q14896|P10916|P08590
GO:0006950	response to stress	7.81E-04	5.91E-03	39	3628	178	27688	P07602|P0CG47|P13929|P51684|Q12988|P16615|P35613|P0CG48|P16860|P84243|P60033|P68871|Q9UBY9|P09493|P22352|P62979|P69905|P62158|P24752|Q01628|P00441|P01034|P51452|O00217|P01033|P02144|Q14116|O15273|O14880|P19021|P16989|P12883|P09382|P62987|P04275|P04792|P25116|P20585|O14558
GO:0006977	DNA damage response, signal transduction by p53 class mediator resulting in cell cycle arrest	8.72E-04	6.35E-03	4	66	178	27688	P0CG47|P62987|P62979|P0CG48
GO:0006979	response to oxidative stress	5.01E-04	4.02E-03	9	354	178	27688	O00217|P02144|Q14116|P68871|P09493|P22352|P69905|P00441|P12883
GO:0006986	response to unfolded protein	1.30E-02	4.95E-02	3	76	178	27688	Q12988|Q9UBY9|P04792
GO:0007093	mitotic cell cycle checkpoint	2.74E-03	1.52E-02	5	148	178	27688	P0CG47|P62987|P83731|P62979|P0CG48
GO:0007167	enzyme linked receptor protein signaling pathway	9.64E-03	4.01E-02	11	752	178	27688	P0CG47|P51452|P16860|P01160|P62987|P62979|P62158|P17081|P13533|P62753|P0CG48
GO:0007173	epidermal growth factor receptor signaling	2.04E-03	1.20E-02	4	83	178	27688	P0CG47|P62987|P62979|P0CG48
GO:0007249	I-kappaB kinase/NF-kappaB signaling	4.67E-04	3.80E-03	4	56	178	27688	P0CG47|P62987|P62979|P0CG48
GO:0007254	JNK cascade	3.09E-03	1.66E-02	4	93	178	27688	P0CG47|P62987|P62979|P0CG48
GO:0007275	multicellular organism development	2.80E-14	7.62E-13	76	5038	178	27688	P10916|P62280|P35613|P39019|P63244|P16860|O14949|P39023|P62273|P09493|P46783|P83731|P62913|P45379|P62753|P24752|Q07020|P01033|Q14896|P19021|P19022|P16989|P68032|Q08431|P84098|O14958|P63173|P08493|P61353|P60660|P09382|P46777|P05386|Q13772|P60866|P46776|P05387|P62249|P07602|P25398|P62891|P42766|Q02878|P19429|P25705|P84243|Q14192|P61247|P62857|P62979|P62851|P00441|P51452|P02144|Q14116|O15273|P08708|O75116|P62906|P68133|P18077|P13533|P12883|P42677|P15880|Q02543|P08590|P62263|P62987|P62945|P04275|P04792|P62269|P20585|P06576|P63316
GO:0007346	regulation of mitotic cell cycle	4.19E-03	2.12E-02	7	312	178	27688	P0CG47|P51452|Q9NWT8|P62987|P83731|P62979|P0CG48
GO:0007507	heart development	1.24E-07	1.78E-06	16	489	178	27688	P02144|O15273|Q14896|P10916|P19021|P68032|P13533|P12883|P16860|O14958|P19429|Q14192|P08590|P09493|P45379|P63316
GO:0007512	adult heart development	1.38E-04	1.28E-03	3	16	178	27688	O15273|P13533|P12883
GO:0007517	muscle organ development	2.19E-08	3.40E-07	13	268	178	27688	O15273|Q14896|P10916|P68133|P68032|P13533|P12883|P19429|P60660|P08590|P09493|P45379|P63316
GO:0007596	blood coagulation	2.00E-03	1.19E-02	10	518	178	27688	P07602|P01033|P84243|P68871|P16615|P04275|P62158|P35613|P25116|P00441
GO:0007599	hemostasis	2.18E-03	1.26E-02	10	524	178	27688	P07602|P01033|P84243|P68871|P16615|P04275|P62158|P35613|P25116|P00441
GO:0008015	blood circulation	2.65E-09	4.44E-08	15	319	178	27688	Q14116|O15273|Q14896|P10916|P01160|P68032|P13533|P16860|P19429|P68871|P08590|P09493|P25116|P00441|P63316
GO:0008016	regulation of heart contraction	1.04E-05	1.22E-04	7	116	178	27688	Q9UBY9|P08590|P09493|P17661|P45379|P13533|P12883
GO:0008063	Toll signaling pathway	1.41E-04	1.30E-03	5	77	178	27688	P0CG47|P51452|P62987|P62979|P0CG48
GO:0008152	metabolic process	8.85E-14	2.24E-12	123	11504	178	27688	O75947|O43674|P16615|O96000|P16860|P13073|O14949|P07195|P46783|P83731|P62913|P62753|P24752|O15239|P20674|Q13765|O95169|P13639|P48047|P04406|O14561|Q9Y5N6|O95167|P84098|P63173|P68104|P08574|P47985|P46777|P60866|P46776|P60228|P62249|Q13011|Q8N335|P07602|P25398|P68363|P10176|O95178|P12235|P62891|P25705|P18859|Q14192|P68871|Q16654|P56381|P00441|P54368|O00217|Q14116|Q08493|P08708|O75116|P18077|Q8N8D1|P42677|P41222|P15880|P17540|Q02543|Q9Y3D2|P62263|Q14240|P62945|P62269|P0CG47|P06732|Q9NSD9|P01160|P40926|P40925|P62280|P36542|P35613|P0CG48|P39019|P05090|P00505|P39023|P62273|P24310|P69905|P62158|P24311|Q07020|P10606|O14880|P19021|O43920|Q05639|P61353|P05386|P05387|P13929|P09669|O00483|P14854|P42766|Q02878|P60033|Q9NPI5|P17174|P61247|P62857|P22352|Q16698|P62979|P62851|P51452|P51970|P62906|P15954|P51857|Q00325|P62987|P04275|P04792|P25116|P20585|P06576|P48735
GO:0008217	regulation of blood pressure	5.48E-05	5.65E-04	7	150	178	27688	P16860|P19429|P01160|P68871|P09493|P13533|P00441
GO:0008219	cell death	1.13E-02	4.59E-02	15	1206	178	27688	P0CG47|P02792|Q12988|P04406|Q9H3K2|P68032|P0CG48|Q8N8D1|P09382|P62987|P61247|P62979|P04792|P25116|P00441
GO:0009056	catabolic process	1.09E-03	7.56E-03	22	1668	178	27688	P0CG47|P13929|Q08493|P04406|P51857|P0CG48|P00505|P68871|P07195|P17174|P62987|Q14240|P22352|Q16698|P62979|P69905|P62158|P60228|P24752|Q13011|Q8N335|P00441

<table>
<tr><td>GO:0009058</td><td>biosynthetic process</td><td>9.26E-20</td><td>3.94E-18</td><td>71</td><td>3544</td><td>178</td><td>27688</td><td>O75947|P06732|Q9NSD9|P01160|P40926|P40925|P16615|P62280|P36542|P39019|P16860|P00505|P39023|P62273|P46783|P83731|P62913|P62753|P24752|Q13765|Q07020|P13639|P48047|P04406|O14561|O14880|Q9Y5N6|P84098|Q05639|P63173|P68104|P61353|P46777|P05386|P60866|P46776|P05387|P60228|P62249|P13929|P25398|P62891|P42766|Q02878|P25705|P18859|P60033|Q9NPI5|P17174|P61247|P62857|P62979|P56381|P62851|P00441|P54368|Q14116|P08708|P62906|P18077|P51857|P42677|P41222|P15880|Q02543|P62263|P62987|Q14240|P62945|P62269|P06576</td></tr>
<tr><td>GO:0009059</td><td>macromolecule biosynthetic process</td><td>7.24E-13</td><td>1.63E-11</td><td>44</td><td>2050</td><td>178</td><td>27688</td><td>P25398|Q9NSD9|P62280|P39019|P62891|P42766|Q02878|P39023|P62273|P46783|P61247|P83731|P62857|P62913|P62979|P62851|P62753|Q13765|Q07020|P13639|P08708|P62906|Q9Y5N6|P18077|P42677|P84098|P15880|Q05639|P63173|P68104|Q02543|P61353|P62263|P62987|Q14240|P62945|P46777|P05386|P60866|P46776|P62269|P05387|P60228|P62249</td></tr>
<tr><td>GO:0009060</td><td>aerobic respiration</td><td>1.21E-02</td><td>4.83E-02</td><td>3</td><td>74</td><td>178</td><td>27688</td><td>P40926|P40925|P48735</td></tr>
<tr><td>GO:0009116</td><td>nucleoside metabolic process</td><td>2.46E-12</td><td>5.20E-11</td><td>22</td><td>512</td><td>178</td><td>27688</td><td>O00217|P13929|O75947|O95169|P51970|P48047|O43674|P04406|O14561|O00483|P16615|O43920|P36542|O95167|O95178|O96000|P25705|P18859|P07195|P56381|P06576|O15239</td></tr>
<tr><td>GO:0009117</td><td>nucleotide metabolic process</td><td>2.99E-14</td><td>8.02E-13</td><td>28</td><td>728</td><td>178</td><td>27688</td><td>P13929|O75947|P01160|O43674|P40926|P40925|O00483|P16615|P36542|O95178|O96000|P16860|P25705|P18859|Q9NPI5|P07195|P56381|O15239|O00217|Q08493|O95169|P51970|P48047|P04406|O14561|O43920|O95167|P06576</td></tr>
<tr><td>GO:0009119</td><td>ribonucleoside metabolic process</td><td>4.92E-13</td><td>1.14E-11</td><td>22</td><td>472</td><td>178</td><td>27688</td><td>O00217|P13929|O75947|O95169|P51970|P48047|O43674|P04406|O14561|O00483|P16615|O43920|P36542|O95167|O95178|O96000|P25705|P18859|P07195|P56381|P06576|O15239</td></tr>
<tr><td>GO:0009123</td><td>nucleoside monophosphate metabolic process</td><td>1.10E-14</td><td>3.10E-13</td><td>22</td><td>391</td><td>178</td><td>27688</td><td>O00217|P13929|O75947|O95169|P51970|P48047|O43674|P04406|O14561|O00483|P16615|O43920|P36542|O95167|O95178|O96000|P25705|P18859|P07195|P56381|P06576|O15239</td></tr>
<tr><td>GO:0009124</td><td>nucleoside monophosphate biosynthetic process</td><td>1.32E-05</td><td>1.52E-04</td><td>8</td><td>167</td><td>178</td><td>27688</td><td>O75947|P25705|P48047|P18859|P16615|P36542|P56381|P06576</td></tr>
<tr><td>GO:0009126</td><td>purine nucleoside monophosphate metabolic process</td><td>3.73E-15</td><td>1.14E-13</td><td>22</td><td>371</td><td>178</td><td>27688</td><td>O00217|P13929|O75947|O95169|P51970|P48047|O43674|P04406|O14561|O00483|P16615|O43920|P36542|O95167|O95178|O96000|P25705|P18859|P07195|P56381|P06576|O15239</td></tr>
<tr><td>GO:0009127</td><td>purine nucleoside monophosphate biosynthetic</td><td>6.33E-06</td><td>7.74E-05</td><td>8</td><td>151</td><td>178</td><td>27688</td><td>O75947|P25705|P48047|P18859|P16615|P36542|P56381|P06576</td></tr>
<tr><td>GO:0009132</td><td>nucleoside diphosphate metabolic process</td><td>2.51E-03</td><td>1.41E-02</td><td>5</td><td>145</td><td>178</td><td>27688</td><td>P13929|P25705|P04406|P07195|P06576</td></tr>
<tr><td>GO:0009135</td><td>purine nucleoside diphosphate metabolic process</td><td>1.55E-03</td><td>9.84E-03</td><td>5</td><td>130</td><td>178</td><td>27688</td><td>P13929|P25705|P04406|P07195|P06576</td></tr>
<tr><td>GO:0009141</td><td>nucleoside triphosphate metabolic process</td><td>5.19E-15</td><td>1.57E-13</td><td>22</td><td>377</td><td>178</td><td>27688</td><td>O00217|P13929|O75947|O95169|P51970|P48047|O43674|P04406|O14561|O00483|P16615|O43920|P36542|O95167|O95178|O96000|P25705|P18859|P07195|P56381|P06576|O15239</td></tr>
<tr><td>GO:0009142</td><td>nucleoside triphosphate biosynthetic process</td><td>5.46E-06</td><td>6.75E-05</td><td>8</td><td>148</td><td>178</td><td>27688</td><td>O75947|P25705|P48047|P18859|P16615|P36542|P56381|P06576</td></tr>
<tr><td>GO:0009144</td><td>purine nucleoside triphosphate metabolic process</td><td>2.51E-15</td><td>8.17E-14</td><td>22</td><td>364</td><td>178</td><td>27688</td><td>O00217|P13929|O75947|O95169|P51970|P48047|O43674|P04406|O14561|O00483|P16615|O43920|P36542|O95167|O95178|O96000|P25705|P18859|P07195|P56381|P06576|O15239</td></tr>
<tr><td>GO:0009145</td><td>purine nucleoside triphosphate biosynthetic</td><td>4.23E-06</td><td>5.30E-05</td><td>8</td><td>143</td><td>178</td><td>27688</td><td>O75947|P25705|P48047|P18859|P16615|P36542|P56381|P06576</td></tr>
<tr><td>GO:0009150</td><td>purine ribonucleotide metabolic process</td><td>8.79E-16</td><td>3.04E-14</td><td>25</td><td>480</td><td>178</td><td>27688</td><td>P13929|O75947|P01160|O43674|O00483|P16615|P36542|O95178|O96000|P16860|P25705|P18859|P07195|P56381|O15239|O00217|Q08493|O95169|P51970|P48047|P04406|O14561|O43920|O95167|P06576</td></tr>
<tr><td>GO:0009152</td><td>purine ribonucleotide biosynthetic process</td><td>1.36E-06</td><td>1.83E-05</td><td>10</td><td>214</td><td>178</td><td>27688</td><td>O75947|P16860|P25705|P48047|P18859|P01160|P16615|P36542|P56381|P06576</td></tr>
<tr><td>GO:0009156</td><td>ribonucleoside monophosphate biosynthetic</td><td>1.11E-05</td><td>1.28E-04</td><td>8</td><td>163</td><td>178</td><td>27688</td><td>O75947|P25705|P48047|P18859|P16615|P36542|P56381|P06576</td></tr>
<tr><td>GO:0009161</td><td>ribonucleoside monophosphate metabolic process</td><td>7.17E-15</td><td>2.08E-13</td><td>22</td><td>383</td><td>178</td><td>27688</td><td>O00217|P13929|O75947|O95169|P51970|P48047|O43674|P04406|O14561|O00483|P16615|O43920|P36542|O95167|O95178|O96000|P25705|P18859|P07195|P56381|P06576|O15239</td></tr>
<tr><td>GO:0009163</td><td>nucleoside biosynthetic process</td><td>7.49E-05</td><td>7.48E-04</td><td>8</td><td>213</td><td>178</td><td>27688</td><td>O75947|P25705|P48047|P18859|P16615|P36542|P56381|P06576</td></tr>
<tr><td>GO:0009165</td><td>nucleotide biosynthetic process</td><td>1.41E-05</td><td>1.60E-04</td><td>11</td><td>341</td><td>178</td><td>27688</td><td>O75947|P16860|P25705|P48047|P18859|P01160|Q9NPI5|P16615|P36542|P56381|P06576</td></tr>
<tr><td>GO:0009167</td><td>purine ribonucleoside monophosphate metabolic process</td><td>3.73E-15</td><td>1.14E-13</td><td>22</td><td>371</td><td>178</td><td>27688</td><td>O00217|P13929|O75947|O95169|P51970|P48047|O43674|P04406|O14561|O00483|P16615|O43920|P36542|O95167|O95178|O96000|P25705|P18859|P07195|P56381|P06576|O15239</td></tr>
<tr><td>GO:0009168</td><td>purine ribonucleoside monophosphate</td><td>6.33E-06</td><td>7.74E-05</td><td>8</td><td>151</td><td>178</td><td>27688</td><td>O75947|P25705|P48047|P18859|P16615|P36542|P56381|P06576</td></tr>
<tr><td>GO:0009179</td><td>purine ribonucleoside diphosphate metabolic</td><td>1.55E-03</td><td>9.84E-03</td><td>5</td><td>130</td><td>178</td><td>27688</td><td>P13929|P25705|P04406|P07195|P06576</td></tr>
<tr><td>GO:0009185</td><td>ribonucleoside diphosphate metabolic process</td><td>1.66E-03</td><td>1.03E-02</td><td>5</td><td>132</td><td>178</td><td>27688</td><td>P13929|P25705|P04406|P07195|P06576</td></tr>
<tr><td>GO:0009187</td><td>cyclic nucleotide metabolic process</td><td>1.30E-02</td><td>4.95E-02</td><td>3</td><td>76</td><td>178</td><td>27688</td><td>P16860|Q08493|P01160</td></tr>
<tr><td>GO:0009199</td><td>ribonucleoside triphosphate metabolic process</td><td>1.89E-15</td><td>6.23E-14</td><td>22</td><td>359</td><td>178</td><td>27688</td><td>O00217|P13929|O75947|O95169|P51970|P48047|O43674|P04406|O14561|O00483|P16615|O43920|P36542|O95167|O95178|O96000|P25705|P18859|P07195|P56381|P06576|O15239</td></tr>
<tr><td>GO:0009201</td><td>ribonucleoside triphosphate biosynthetic process</td><td>4.23E-06</td><td>5.30E-05</td><td>8</td><td>143</td><td>178</td><td>27688</td><td>O75947|P25705|P48047|P18859|P16615|P36542|P56381|P06576</td></tr>
<tr><td>GO:0009205</td><td>purine ribonucleoside triphosphate metabolic process</td><td>1.78E-15</td><td>5.97E-14</td><td>22</td><td>358</td><td>178</td><td>27688</td><td>O00217|P13929|O75947|O95169|P51970|P48047|O43674|P04406|O14561|O00483|P16615|O43920|P36542|O95167|O95178|O96000|P25705|P18859|P07195|P56381|P06576|O15239</td></tr>
<tr><td>GO:0009206</td><td>purine ribonucleoside triphosphate biosynthetic</td><td>4.02E-06</td><td>5.09E-05</td><td>8</td><td>142</td><td>178</td><td>27688</td><td>O75947|P25705|P48047|P18859|P16615|P36542|P56381|P06576</td></tr>
<tr><td>GO:0009259</td><td>ribonucleotide metabolic process</td><td>1.35E-15</td><td>4.59E-14</td><td>25</td><td>489</td><td>178</td><td>27688</td><td>P13929|O75947|P01160|O43674|O00483|P16615|P36542|O95178|O96000|P16860|P25705|P18859|P07195|P56381|O15239|O00217|Q08493|O95169|P51970|P48047|P04406|O14561|O43920|O95167|P06576</td></tr>
<tr><td>GO:0009260</td><td>ribonucleotide biosynthetic process</td><td>1.89E-06</td><td>2.51E-05</td><td>10</td><td>222</td><td>178</td><td>27688</td><td>O75947|P16860|P25705|P48047|P18859|P01160|P16615|P36542|P56381|P06576</td></tr>
<tr><td>GO:0009611</td><td>response to wounding</td><td>1.05E-03</td><td>7.37E-03</td><td>14</td><td>835</td><td>178</td><td>27688</td><td>P07602|P01033|P13929|P16615|P35613|P84243|P60033|P09382|P68871|P09493|P04275|P62158|P25116|P00441</td></tr>
<tr><td>GO:0009653</td><td>anatomical structure morphogenesis</td><td>3.41E-03</td><td>1.82E-02</td><td>26</td><td>2300</td><td>178</td><td>27688</td><td>P10916|P35613|P19429|Q14192|P09493|P83731|P45379|P00441|Q14116|O15273|Q14896|O75116|P62906|P19022|P68133|P68032|P13533|Q08431|P12883|P08493|P60660|P08590|P04792|P25116|P06576|P63316</td></tr>
<tr><td>GO:0009719</td><td>response to endogenous stimulus</td><td>3.17E-05</td><td>3.41E-04</td><td>24</td><td>1489</td><td>178</td><td>27688</td><td>P01033|P02144|Q14116|P01160|P19021|P68133|P13533|P35613|Q08431|Q8N8D1|P16860|P41222|P84243|P08493|Q14192|P17174|P08574|P47985|P22352|Q13772|P17081|P62753|P24752|P00441</td></tr>
<tr><td>GO:0009725</td><td>response to hormone</td><td>2.04E-06</td><td>2.69E-05</td><td>22</td><td>1086</td><td>178</td><td>27688</td><td>P01033|P02144|Q14116|P01160|P19021|P68133|P35613|Q08431|Q8N8D1|P16860|P41222|P84243|P08493|Q14192|P17174|P08574|P47985|P22352|Q13772|P17081|P62753|P24752</td></tr>
<tr><td>GO:0009887</td><td>organ morphogenesis</td><td>1.78E-03</td><td>1.10E-02</td><td>15</td><td>983</td><td>178</td><td>27688</td><td>O15273|Q14896|P10916|P68032|P13533|P35613|P12883|P19429|P08493|Q14192|P08590|P09493|P45379|P00441|P63316</td></tr>
<tr><td>GO:0009888</td><td>tissue development</td><td>8.40E-04</td><td>6.26E-03</td><td>21</td><td>1526</td><td>178</td><td>27688</td><td>P07602|P13929|O15273|Q14896|P10916|P68133|P16615|P68032|P13533|P35613|P12883|P19429|P08493|Q14192|P60660|P08590|P09493|P45379|P24752|P00441|P63316</td></tr>
<tr><td>GO:0009894</td><td>regulation of catabolic process</td><td>7.82E-03</td><td>3.38E-02</td><td>7</td><td>351</td><td>178</td><td>27688</td><td>P0CG47|P01033|P63244|P62987|P62979|P0CG48|P01034</td></tr>
<tr><td>GO:0009896</td><td>positive regulation of catabolic process</td><td>9.63E-03</td><td>4.01E-02</td><td>5</td><td>200</td><td>178</td><td>27688</td><td>P0CG47|P63244|P62987|P62979|P0CG48</td></tr>
</table>

GO:0009987	cellular process	1.03E-14	2.94E-13	163	18611	178	27688	O75947|P10916|O43674|Q9H3K2|P16615|O96000|P16860|P13073|O14949|P09493|P07195|P46783|P83731|P62913|P62753|P24752|O15239|P20674|Q13765|O95169|P13639|P48047|Q9Y277|P04406|O14561|P80297|Q9Y5N6|O95167|Q08431|P19105|P84098|P63173|P68104|P09382|P08574|P47985|P46777|Q13772|P60866|P46776|P60228|P62249|Q13011|Q8N335|P07602|P25398|P68363|P10176|O95178|P12235|P13693|P62891|P19429|P25705|P18859|Q14192|P68871|Q16654|P56381|Q01628|P00441|P54368|O00217|Q14116|O15273|Q08493|P08708|Q14515|O75116|P68133|P18077|P17661|P17535|P12883|Q8N8D1|P42677|P41222|P15880|P17540|Q02543|Q9Y3D2|P62263|Q14240|P62945|P62269|P0CG47|P06732|Q9NSD9|Q12988|P01160|P40926|P40925|P62280|P36542|P35613|P0CG48|P39019|Q01449|P00505|P39023|P62273|P24310|P69905|P62158|P45379|P24311|P01034|Q07020|P01033|P10606|Q14896|O14880|P19021|P19022|P37108|O43920|P68032|Q05639|P08493|P61353|P60660|P02511|P05386|P17081|P05387|P13929|P02792|P09669|P51684|O00483|P14854|P42766|Q02878|P84243|P60033|Q9NPI5|P17174|P61247|P62857|P22352|Q16698|P62979|P62851|P51452|P02144|Q14315|P51970|Q9P0U1|P62906|P15954|P51857|P13533|Q00325|P08590|P62987|P04275|P63313|P04792|P25116|P20585|P06576|P63316|P48735
GO:0010033	response to organic substance	7.05E-08	1.06E-06	39	2426	178	27688	P0CG47|Q12988|P01160|P35613|P0CG48|P16860|P84243|P00505|Q14192|Q9UBY9|P17174|P22352|P62979|P62158|P62753|P24752|Q01628|P00441|P51452|P01033|P02144|Q14116|P80297|P19021|P68133|P68032|P13533|Q08431|Q8N8D1|P41222|P08493|P09382|P08574|P62987|P47985|Q13772|P17081|P04792|P25116
GO:0010035	response to inorganic substance	6.06E-08	9.16E-07	16	464	178	27688	P02144|Q14116|P80297|P19021|P68133|P35613|P17535|P16860|Q05639|P08493|P68871|P22352|P69905|P62158|P45379|P00441
GO:0010038	response to metal ion	3.57E-05	3.80E-04	10	311	178	27688	P16860|P08493|P80297|P19021|P68133|P62158|P45379|P35613|P17535|P00441
GO:0010467	gene expression	2.14E-12	4.58E-11	45	2206	178	27688	P25398|Q9NSD9|P62280|P39019|P62891|P42766|Q02878|P39023|P62273|P46783|P61247|P83731|P62857|P62913|P62979|P62851|P62753|Q13765|Q07020|P13639|P08708|P62906|P18077|Q8N8D1|P42677|P84098|P15880|Q05639|P63173|P68104|Q02543|P61353|P62263|P62987|Q14240|P62945|P46777|P05386|P60866|P46776|P62269|P05387|P60228|P25116|P62249
GO:0010564	regulation of cell cycle process	7.94E-03	3.42E-02	7	352	178	27688	P0CG47|Q9NWT8|O75116|P62987|P62979|P62158|P0CG48
GO:0010608	posttranscriptional regulation of gene expression	7.12E-03	3.11E-02	6	262	178	27688	P63244|P62263|Q14240|P37108|P04792|P60228
GO:0010712	regulation of collagen metabolic process	1.99E-04	1.76E-03	3	18	178	27688	Q14116|P25116|P01034
GO:0010927	cellular component assembly involved in	4.47E-04	3.65E-03	6	151	178	27688	O15273|P10916|P09493|P68133|P68032|P13533
GO:0010941	regulation of cell death	4.11E-04	3.38E-03	20	1340	178	27688	P0CG47|P01033|Q14116|P16989|P12235|P0CG48|P13693|P63244|Q05639|Q14192|P09382|P68871|P62987|P62979|P02511|P69905|P04792|P62753|P25116|P00441
GO:0010948	negative regulation of cell cycle process	5.70E-03	2.73E-02	5	176	178	27688	P0CG47|Q9NWT8|P62987|P62979|P0CG48
GO:0014070	response to organic cyclic compound	3.88E-03	2.02E-02	14	963	178	27688	Q14116|P19021|P68133|P35613|Q08431|Q8N8D1|P16860|P41222|Q14192|P09382|P17174|P22352|Q13772|P24752
GO:0014706	striated muscle tissue development	5.13E-10	9.38E-09	14	238	178	27688	O15273|Q14896|P10916|P68133|P68032|P13533|P12883|P19429|Q14192|P60660|P08590|P09493|P45379|P63316
GO:0014866	skeletal myofibril assembly	1.43E-05	1.62E-04	3	8	178	27688	O15273|P68133|P68032
GO:0015669	gas transport	1.12E-04	1.08E-03	3	15	178	27688	P68871|P69905|P39019
GO:0015980	energy derivation by oxidation of organic compounds	1.47E-29	1.23E-27	34	363	178	27688	O75947|P09669|O43674|P40926|P40925|O00483|P14854|P36542|P10176|O95178|P12235|O96000|P13073|P25705|P18859|O14949|P56381|P62158|P24311|O15239|P20674|O00217|P10606|O95169|P51970|P48047|O14561|O43920|P15954|O95167|P08574|P47985|P06576|P48735
GO:0015985	energy coupled proton transport, down	1.50E-09	2.63E-08	7	33	178	27688	O75947|P25705|P48047|P18859|P36542|P56381|P06576
GO:0015986	ATP synthesis coupled proton transport	1.50E-09	2.63E-08	7	33	178	27688	O75947|P25705|P48047|P18859|P36542|P56381|P06576
GO:0015992	proton transport	3.87E-05	4.08E-04	7	142	178	27688	O75947|P25705|P48047|P18859|P36542|P56381|P06576
GO:0016032	viral process	1.39E-33	1.79E-31	40	458	178	27688	P0CG47|P25398|P62280|P12235|P0CG48|P39019|P62891|P42766|Q02878|P60033|P39023|P62273|P46783|P61247|P83731|P62857|P62913|P62979|P62851|P62753|Q07020|P08708|P62906|P18077|P42677|P84098|P15880|P63173|Q02543|P61353|P62263|P62987|P62945|P46777|P05386|P60866|P46776|P62269|P05387|P62249
GO:0016043	cellular component organization	3.41E-14	9.05E-13	72	4606	178	27688	P0CG47|P10916|P62280|P0CG48|P39019|P39023|P62273|P09493|P46783|P83731|P62913|P69905|P62753|P24752|P01034|Q07020|P19022|P37108|O43920|P68032|Q08431|P19105|P84098|P63173|P61353|P60660|P46777|P02511|P05386|P17081|P60866|P46776|P05387|P62249|P02792|P25398|P68363|P12235|P62891|P42766|Q02878|P84243|P68871|P61247|P62857|P22352|Q16698|P62979|P62851|P00441|O00217|Q14315|O15273|Q9P0U1|P08708|O75116|P62906|P68133|P18077|P17661|P13533|P42677|P15880|Q02543|P62263|P62987|P62945|P04275|P63313|P62269|P25116|P20585
GO:0016051	carbohydrate biosynthetic process	1.06E-03	7.37E-03	6	178	178	27688	P13929|P00505|P40926|P04406|P40925|P17174
GO:0016053	organic acid biosynthetic process	9.71E-03	4.03E-02	7	366	178	27688	P41222|P00505|O14561|O14880|P17174|P51857|P24752
GO:0016054	organic acid catabolic process	4.16E-03	2.11E-02	6	234	178	27688	P00505|P17174|Q16698|P51857|P24752|Q13011
GO:0016070	RNA metabolic process	3.19E-15	1.01E-13	44	1755	178	27688	P0CG47|P25398|Q9NSD9|P62280|P0CG48|P39019|P62891|P42766|Q02878|P39023|P62273|P46783|P61247|P83731|P62857|P62913|P62979|P62851|P62753|Q13765|Q07020|P08708|P62906|P18077|Q8N8D1|P42677|P84098|P15880|P63173|Q02543|P61353|P62263|P62987|Q14240|P62945|P46777|P05386|P04792|P60866|P46776|P62269|P05387|P60228|P62249
GO:0016071	mRNA metabolic process	8.91E-29	6.93E-27	41	651	178	27688	P0CG47|P25398|P62280|P0CG48|P39019|P62891|P42766|Q02878|P39023|P62273|P46783|P61247|P83731|P62857|P62913|P62979|P62851|P62753|Q07020|P08708|P62906|P18077|P42677|P84098|P15880|P63173|Q02543|P61353|P62263|P62987|Q14240|P62945|P46777|P05386|P04792|P60866|P46776|P62269|P05387|P60228|P62249
GO:0016072	rRNA metabolic process	1.73E-07	2.48E-06	9	130	178	27688	P08708|P62263|P18077|P62857|P62913|P46777|P62753|P62249|P39019
GO:0016197	endosomal transport	9.25E-03	3.89E-02	4	127	178	27688	P0CG47|P62987|P62979|P0CG48
GO:0016265	death	1.18E-02	4.76E-02	15	1212	178	27688	P0CG47|P02792|Q12988|P04406|Q9H3K2|P68032|P0CG48|Q8N8D1|P09382|P62987|P61247|P62979|P04792|P25116|P00441
GO:0016310	phosphorylation	7.16E-07	9.86E-06	23	1103	178	27688	P0CG47|P51452|O00217|P13929|O95169|P51970|O43674|P04406|O14561|O75116|O00483|O43920|P36542|O95167|O95178|P0CG48|O96000|P07195|P62987|Q16654|P62979|P25116|O15239
GO:0017148	negative regulation of translation	1.26E-02	4.91E-02	3	75	178	27688	P63244|P37108|P60228
GO:0018130	heterocycle biosynthetic process	9.73E-25	6.23E-23	48	1218	178	27688	O75947|P25398|P01160|P16615|P62280|P36542|P39019|P62891|P42766|Q02878|P16860|P25705|P18859|P39023|P62273|Q9NPI5|P46783|P61247|P83731|P62857|P62913|P62979|P56381|P62851|P62753|Q13765|Q07020|P48047|P08708|P62906|P18077|P42677|P84098|P15880|P63173|Q02543|P61353|P62263|P62987|P62945|P46777|P05386|P60866|P46776|P62269|P05387|P62249|P06576
GO:0019058	viral life cycle	1.56E-49	4.24E-47	37	142	178	27688	P25398|P62280|P39019|P62891|P42766|Q02878|P60033|P39023|P62273|P46783|P61247|P83731|P62857|P62913|P62979|P62851|P62753|Q07020|P08708|P62906|P18077|P42677|P84098|P15880|P63173|Q02543|P61353|P62263|P62987|P62945|P46777|P05386|P60866|P46776|P62269|P05387|P62249
GO:0019080	viral gene expression	1.60E-57	1.74E-54	36	84	178	27688	P25398|P62280|P39019|P62891|P42766|Q02878|P39023|P62273|P46783|P61247|P83731|P62857|P62913|P62979|P62851|P62753|Q07020|P08708|P62906|P18077|P42677|P84098|P15880|P63173|Q02543|P61353|P62263|P62987|P62945|P46777|P05386|P60866|P46776|P62269|P05387|P62249
GO:0019083	viral transcription	1.60E-57	1.74E-54	36	84	178	27688	P25398|P62280|P39019|P62891|P42766|Q02878|P39023|P62273|P46783|P61247|P83731|P62857|P62913|P62979|P62851|P62753|Q07020|P08708|P62906|P18077|P42677|P84098|P15880|P63173|Q02543|P61353|P62263|P62987|P62945|P46777|P05386|P60866|P46776|P62269|P05387|P62249

GO:0019318	hexose metabolic process	1.28E-04	1.20E-03	8	230	178	27688	P13929|P00505|P40926|P04406|P40925|P17174|Q16654|P62158
GO:0019319	hexose biosynthetic process	2.96E-06	3.79E-05	6	62	178	27688	P13929|P00505|P40926|P04406|P40925|P17174
GO:0019362	pyridine nucleotide metabolic process	1.52E-03	9.70E-03	6	191	178	27688	P13929|P40926|P04406|Q9NPI5|P07195|P40925
GO:0019438	aromatic compound biosynthetic process	9.06E-25	5.98E-23	48	1216	178	27688	O75947|P25398|P01160|P16615|P62280|P36542|P39019|P62891|P42766|Q02878|P16860|P25705|P18859|P39023|P62273|Q9NPI5|P46783|P61247|P83731|P62857|P62913|P62979|P56381|P62851|P62753|Q13765|Q07020|P48047|P08708|P62906|P18077|P42677|P84098|P15880|P63173|Q02543|P61353|P62263|P62987|P62945|P46777|P05386|P60866|P46776|P62269|P05387|P62249|P06576
GO:0019538	protein metabolic process	1.05E-05	1.22E-04	56	5004	178	27688	P0CG47|Q9NSD9|P40926|P62280|P0CG48|P39019|P39023|P62273|P46783|P83731|P62913|P62753|Q13765|Q07020|P13639|P04406|P19021|P84098|Q05639|P63173|P68104|P61353|P46777|P05386|P60866|P46776|P05387|P60228|P62249|P25398|P68363|P62891|P42766|Q02878|P61247|P62857|Q16654|P62979|P62851|P51452|Q14116|P08708|O75116|P62906|P18077|P42677|P15880|Q02543|Q9Y3D2|P62263|P62987|Q14240|P62945|P04275|P62269|P25116
GO:0019637	organophosphate metabolic process	1.41E-11	2.79E-10	31	1156	178	27688	P13929|O75947|P06732|P01160|O43674|P40926|P40925|O00483|P16615|P36542|O95178|O96000|P16860|P25705|P18859|P60033|Q9NPI5|P07195|P56381|O15239|O00217|Q08493|O95169|P51970|P48047|P04406|O14561|O43920|O95167|Q8N335|P06576
GO:0019674	NAD metabolic process	3.87E-03	2.02E-02	3	49	178	27688	P40926|P07195|P40925
GO:0019693	ribose phosphate metabolic process	3.09E-15	9.89E-14	25	507	178	27688	P13929|O75947|P01160|O43674|O00483|P16615|P36542|O95178|O96000|P16860|P25705|P18859|P07195|P56381|O15239|O00217|Q08493|O95169|P51970|P48047|P04406|O14561|O43920|O95167|P06576
GO:0019752	carboxylic acid metabolic process	4.15E-04	3.39E-03	20	1341	178	27688	P13929|P06732|Q9NSD9|P40926|P04406|O14561|P40925|O14880|P51857|P35613|P41222|P17540|P00505|P07195|P17174|Q16654|Q16698|P24752|Q13011|P48735
GO:0022403	cell cycle phase	1.31E-02	4.96E-02	6	299	178	27688	P0CG47|P42677|P62987|Q9Y5N6|P62979|P0CG48
GO:0022411	cellular component disassembly	9.54E-42	1.73E-39	37	221	178	27688	P25398|P62280|P39019|P62891|P42766|Q02878|P39023|P62273|P46783|P61247|P83731|P62857|P62913|P62979|P62851|P62753|P00441|Q07020|P08708|P62906|P18077|P42677|P84098|P15880|P63173|Q02543|P61353|P62263|P62987|P62945|P46777|P05386|P60866|P46776|P62269|P05387|P62249
GO:0022607	cellular component assembly	1.37E-04	1.28E-03	24	1636	178	27688	O00217|Q14315|O15273|P10916|P08708|P68363|P19022|P68133|O43920|P68032|P13533|P84243|P68871|P09493|P62263|P83731|P22352|Q16698|P02511|P04275|P69905|P62851|P62753|P24752
GO:0022613	ribonucleoprotein complex biogenesis	2.43E-07	3.44E-06	12	275	178	27688	P08708|P62263|P18077|P83731|P62857|P62913|P46777|P62851|P62269|P62753|P62249|P39019
GO:0022618	ribonucleoprotein complex assembly	1.01E-03	7.10E-03	5	118	178	27688	P08708|P62263|P83731|P62851|P62753
GO:0022900	electron transport chain	2.50E-32	2.72E-30	29	179	178	27688	O75947|P09669|O43674|O00483|P14854|P36542|P10176|O95178|O96000|P13073|P25705|P18859|O14949|P56381|P24311|O15239|P20674|O00217|P10606|O95169|P51970|P48047|O14561|O43920|P15954|O95167|P08574|P47985|P06576
GO:0022904	respiratory electron transport chain	3.78E-36	5.48E-34	29	135	178	27688	O75947|P09669|O43674|O00483|P14854|P36542|P10176|O95178|O96000|P13073|P25705|P18859|O14949|P56381|P24311|O15239|P20674|O00217|P10606|O95169|P51970|P48047|O14561|O43920|P15954|O95167|P08574|P47985|P06576
GO:0023014	signal transduction by protein phosphorylation	9.25E-03	3.89E-02	5	198	178	27688	P0CG47|P51452|P62987|P62979|P0CG48
GO:0030029	actin filament-based process	3.25E-08	5.02E-07	17	505	178	27688	O15273|Q14896|P10916|P68133|P17661|P68032|P13533|P12883|Q01449|P19429|P60660|P08590|P09493|P63313|P17081|P45379|P63316
GO:0030036	actin cytoskeleton organization	9.83E-03	4.07E-02	8	458	178	27688	O15273|P10916|P09493|P68133|P68032|P63313|P17081|P13533
GO:0030048	actin filament-based movement	3.00E-20	1.33E-18	15	60	178	27688	O15273|Q14896|P10916|P68133|P17661|P68032|P13533|P12883|Q01449|P19429|P60660|P08590|P09493|P45379|P63316
GO:0030049	muscle filament sliding	1.04E-21	5.24E-20	14	38	178	27688	O15273|Q14896|P10916|P68133|P17661|P68032|P13533|P12883|P19429|P60660|P08590|P09493|P45379|P63316
GO:0030162	regulation of proteolysis	4.30E-03	2.17E-02	9	485	178	27688	P0CG47|P01033|P63244|Q9NWT8|P62987|P62979|P25116|P0CG48|P01034
GO:0030168	platelet activation	1.36E-03	9.02E-03	7	255	178	27688	P07602|P01033|P16615|P04275|P62158|P25116|P00441
GO:0030218	erythrocyte differentiation	1.15E-03	7.85E-03	4	71	178	27688	P01033|P02144|P62263|P39019
GO:0030239	myofibril assembly	4.91E-07	6.89E-06	6	46	178	27688	O15273|P10916|P09493|P68133|P68032|P13533
GO:0030240	skeletal muscle thin filament assembly	8.97E-06	1.07E-04	3	7	178	27688	O15273|P68133|P68032
GO:0030330	DNA damage response, signal transduction by p53	2.14E-03	1.25E-02	4	84	178	27688	P0CG47|P62987|P62979|P0CG48
GO:0030522	intracellular receptor signaling pathway	5.50E-04	4.35E-03	6	157	178	27688	P0CG47|Q14192|P62987|P62979|Q13772|P0CG48
GO:0031016	pancreas development	3.90E-43	7.71E-41	36	185	178	27688	P25398|P62280|P39019|P62891|P42766|Q02878|P39023|P62273|P46783|P61247|P83731|P62857|P62913|P62979|P62851|P62753|Q07020|P08708|P62906|P18077|P42677|P84098|P15880|P63173|Q02543|P61353|P62263|P62987|P62945|P46777|P05386|P60866|P46776|P62269|P05387|P62249
GO:0031018	endocrine pancreas development	1.05E-47	2.29E-45	36	142	178	27688	P25398|P62280|P39019|P62891|P42766|Q02878|P39023|P62273|P46783|P61247|P83731|P62857|P62913|P62979|P62851|P62753|Q07020|P08708|P62906|P18077|P42677|P84098|P15880|P63173|Q02543|P61353|P62263|P62987|P62945|P46777|P05386|P60866|P46776|P62269|P05387|P62249
GO:0031032	actomyosin structure organization	1.05E-05	1.23E-04	6	77	178	27688	O15273|P10916|P09493|P68133|P68032|P13533
GO:0031098	stress-activated protein kinase signaling cascade	1.96E-03	1.17E-02	5	137	178	27688	P0CG47|P51452|P62987|P62979|P0CG48
GO:0031145	anaphase-promoting complex-dependent proteasomal ubiquitin-dependent protein	2.04E-03	1.20E-02	4	83	178	27688	P0CG47|P62987|P62979|P0CG48
GO:0031329	regulation of cellular catabolic process	1.45E-03	9.50E-03	7	258	178	27688	P0CG47|P01033|P63244|P62987|P62979|P0CG48|P01034
GO:0031331	positive regulation of cellular catabolic process	4.58E-03	2.28E-02	5	167	178	27688	P0CG47|P63244|P62987|P62979|P0CG48
GO:0031349	positive regulation of defense response	4.60E-03	2.28E-02	6	239	178	27688	P0CG47|P51452|Q14116|P62987|P62979|P0CG48
GO:0031397	negative regulation of protein ubiquitination	4.01E-03	2.07E-02	4	100	178	27688	P0CG47|P62987|P62979|P0CG48
GO:0031398	positive regulation of protein ubiquitination	7.39E-03	3.22E-02	4	119	178	27688	P0CG47|P62987|P62979|P0CG48
GO:0031571	mitotic G1 DNA damage checkpoint	1.27E-03	8.50E-03	4	73	178	27688	P0CG47|P62987|P62979|P0CG48
GO:0032147	activation of protein kinase activity	1.42E-03	9.32E-03	7	257	178	27688	P0CG47|P60033|P62987|P62979|P25116|P00441|P0CG48
GO:0032269	negative regulation of cellular protein metabolic process	5.22E-03	2.53E-02	11	690	178	27688	P0CG47|P51452|P01033|P63244|P62987|P37108|P62979|P04792|P60228|P0CG48|P01034
GO:0032479	regulation of type I interferon production	5.34E-04	4.28E-03	4	58	178	27688	P0CG47|P62987|P62979|P0CG48
GO:0032480	negative regulation of type I interferon	5.16E-05	5.37E-04	4	32	178	27688	P0CG47|P62987|P62979|P0CG48
GO:0032501	multicellular organismal process	3.81E-17	1.43E-15	94	6554	178	27688	P10916|P01160|P54296|P16615|P62280|P35613|P39019|P63244|P16860|Q01449|Q14094|O14949|P39023|P62273|P09493|P46783|P83731|P62913|P62158|P45379|P62753|P24752|Q07020|P01033|P10606|Q14896|O14880|P19021|P19022|P16989|P68032|Q08431|P84098|O14958|P63173|P08493|P61353|P60660|P09382|P46777|P02511|P05386|Q13772|P60866|P46776|P05387|P62249|P07602|P52179|P25398|P62891|P42766|Q02878|P19429|P25705|P84243|Q14192|P68871|P61247|P62857|P23327|P22352|P62979|P62851|P00441|P51452|P02144|Q14116|O15273|P08708|O75116|P62906|P68133|P18077|P17661|P51857|P13533|P17535|P12883|P42677|P15880|P17540|Q02543|P08590|P62263|P62987|P62945|P04275|P04792|P62269|P25116|P20585|P06576|P63316
GO:0032502	developmental process	1.69E-13	4.09E-12	80	5680	178	27688	P10916|P16615|P62280|P35613|P39019|P63244|P16860|O14949|P39023|P62273|P09493|P46783|P83731|P62913|P45379|P62753|P24752|Q07020|P01033|Q14896|P19021|P19022|P16989|P68032|Q08431|P84098|O14958|P63173|P08493|P61353|P60660|P09382|P46777|P05386|Q13772|P60866|P46776|P05387|P62249|P07602|P13929|P25398|P62891|P42766|Q02878|P19429|P25705|P84243|Q14192|P61247|P62857|P62979|P62851|P00441|P51452|P02144|Q14116|O15273|P08708|O75116|P62906|P68133|P18077|P13533|P17535|P12883|P42677|P15880|Q02543|P08590|P62263|P62987|P62945|P04275|P04792|P62269|P25116|P20585|P06576|P63316
GO:0032651	regulation of interleukin-1 beta production	2.33E-03	1.33E-02	3	41	178	27688	Q14116|P04792|P25116
GO:0032652	regulation of interleukin-1 production	2.85E-03	1.57E-02	3	44	178	27688	Q14116|P04792|P25116

GO:0032774	RNA biosynthetic process	5.54E-24	3.45E-22	37	669	178	27688	P25398|P62280|P39019|P62891|P42766|Q02878|P39023|P62273|P46783|P61247|P83731|P62857|P62913|P62979|P62851|P62753|Q13765|Q07020|P08708|P62906|P18077|P42677|P84098|P15880|P63173|Q02543|P61353|P62263|P62987|P62945|P46777|P05386|P60866|P46776|P62269|P05387|P62249
GO:0032781	positive regulation of ATPase activity	2.13E-06	2.78E-05	4	15	178	27688	Q14896|P08590|P09493|P45379
GO:0032787	monocarboxylic acid metabolic process	1.62E-04	1.48E-03	13	607	178	27688	P13929|P04406|O14561|O14880|P51857|P35613|P41222|P07195|Q16654|Q16698|P24752|Q13011|P48735
GO:0032970	regulation of actin filament-based process	1.91E-03	1.16E-02	6	200	178	27688	Q14896|P09493|O75116|P63313|P17081|P63316
GO:0032984	macromolecular complex disassembly	2.05E-49	4.96E-47	36	129	178	27688	P25398|P62280|P39019|P62891|P42766|Q02878|P39023|P62273|P46783|P61247|P83731|P62857|P62913|P62979|P62851|P62753|Q07020|P08708|P62906|P18077|P42677|P84098|P15880|P63173|Q02543|P61353|P62263|P62987|P62945|P46777|P05386|P60866|P46776|P62269|P05387|P62249
GO:0033275	actin-myosin filament sliding	1.04E-21	5.24E-20	14	38	178	27688	O15273|Q14896|P10916|P68133|P17661|P68032|P13533|P12883|P19429|P60660|P08590|P09493|P45379|P63316
GO:0034101	erythrocyte homeostasis	1.41E-04	1.30E-03	5	77	178	27688	P01033|P02144|P08708|P62263|P39019
GO:0034130	toll-like receptor 1 signaling pathway	8.36E-05	8.24E-04	5	69	178	27688	P0CG47|P51452|P62987|P62979|P0CG48
GO:0034134	toll-like receptor 2 signaling pathway	8.36E-05	8.24E-04	5	69	178	27688	P0CG47|P51452|P62987|P62979|P0CG48
GO:0034138	toll-like receptor 3 signaling pathway	5.40E-05	5.59E-04	5	63	178	27688	P0CG47|P51452|P62987|P62979|P0CG48
GO:0034142	toll-like receptor 4 signaling pathway	1.33E-04	1.24E-03	5	76	178	27688	P0CG47|P51452|P62987|P62979|P0CG48
GO:0034220	ion transmembrane transport	3.44E-04	2.90E-03	9	336	178	27688	O75947|P25705|P48047|P18859|P16615|P36542|P56381|P35613|P06576
GO:0034248	regulation of cellular amide metabolic process	3.90E-03	2.03E-02	6	231	178	27688	P63244|P62263|Q14240|P37108|P04792|P60228
GO:0034249	negative regulation of cellular amide metabolic	1.30E-02	4.95E-02	3	76	178	27688	P63244|P37108|P60228
GO:0034470	ncRNA processing	5.64E-05	5.79E-04	9	264	178	27688	P08708|P62263|P18077|P62857|P62913|P46777|P62753|P62249|P39019
GO:0034614	cellular response to reactive oxygen species	4.83E-03	2.38E-02	3	53	178	27688	Q14116|P09493|P00441
GO:0034622	cellular macromolecular complex assembly	6.37E-03	2.83E-02	10	611	178	27688	O00217|O15273|P84243|P08708|P62263|P68363|P83731|O43920|P62851|P62753
GO:0034641	cellular nitrogen compound metabolic process	1.36E-22	7.57E-21	86	4630	178	27688	P0CG47|O75947|P06732|Q9NSD9|P01160|O43674|P40926|P40925|P16615|P62280|P36542|P0CG48|P39019|O96000|P16860|P00505|P39023|P62273|P07195|P46783|P83731|P62913|P62753|P24752|O15239|Q13765|Q07020|O95169|P13639|P48047|P04406|O14561|P19021|Q9Y5N6|O43920|O95167|P84098|Q05639|P63173|P68104|P61353|P46777|P05386|P60866|P46776|P05387|P60228|P62249|P13929|P25398|O00483|O95178|P62891|P42766|Q02878|P25705|P18859|Q9NPI5|P17174|P61247|P62857|P22352|P62979|P56381|P62851|P00441|P54368|O00217|Q08493|P51970|P08708|P62906|P18077|Q8N8D1|P42677|P15880|P17540|Q02543|P62263|P62987|Q14240|P62945|P04792|P62269|P20585|P06576
GO:0034645	cellular macromolecule biosynthetic process	3.69E-13	8.64E-12	44	2010	178	27688	P25398|Q9NSD9|P62280|P39019|P62891|P42766|Q02878|P39023|P62273|P46783|P61247|P83731|P62857|P62913|P62979|P62851|P62753|Q13765|Q07020|P13639|P08708|P62906|Q9Y5N6|P18077|P42677|P84098|P15880|Q05639|P63173|P68104|Q02543|P61353|P62263|P62987|Q14240|P62945|P46777|P05386|P60866|P46776|P62269|P05387|P60228|P62249
GO:0034654	nucleobase-containing compound biosynthetic process	4.24E-27	3.08E-25	48	1075	178	27688	O75947|P25398|P01160|P16615|P62280|P36542|P39019|P62891|P42766|Q02878|P16860|P25705|P18859|P39023|P62273|Q9NPI5|P46783|P61247|P83731|P62857|P62913|P62979|P56381|P62851|P62753|Q13765|Q07020|P48047|P08708|P62906|P18077|P42677|P84098|P15880|P63173|Q02543|P61353|P62263|P62987|P62945|P46777|P05386|P60866|P46776|P62269|P05387|P62249|P06576
GO:0034660	ncRNA metabolic process	1.62E-03	1.02E-02	10	503	178	27688	Q9NSD9|P08708|P62263|P18077|P62857|P62913|P46777|P62753|P62249|P39019
GO:0035051	cardiocyte differentiation	7.07E-04	5.38E-03	5	109	178	27688	O15273|P10916|Q14192|P68032|P13533
GO:0035150	regulation of tube size	2.36E-03	1.34E-02	5	143	178	27688	P16860|P01160|P68871|P25116|P00441
GO:0035270	endocrine system development	8.16E-38	1.27E-35	36	255	178	27688	P25398|P62280|P39019|P62891|P42766|Q02878|P39023|P62273|P46783|P61247|P83731|P62857|P62913|P62979|P62851|P62753|Q07020|P08708|P62906|P18077|P42677|P84098|P15880|P63173|Q02543|P61353|P62263|P62987|P62945|P46777|P05386|P60866|P46776|P62269|P05387|P62249
GO:0035872	nucleotide-binding domain, leucine rich repeat containing receptor signaling pathway	2.58E-05	2.84E-04	4	27	178	27688	P0CG47|P62987|P62979|P0CG48
GO:0038127	ERBB signaling pathway	2.04E-03	1.20E-02	4	83	178	27688	P0CG47|P62987|P62979|P0CG48
GO:0038179	neurotrophin signaling pathway	2.87E-03	1.58E-02	6	217	178	27688	P0CG47|P51452|P62987|P62979|P62158|P0CG48
GO:0042058	regulation of epidermal growth factor receptor	1.48E-03	9.54E-03	4	76	178	27688	P0CG47|P62987|P62979|P0CG48
GO:0042059	negative regulation of epidermal growth factor receptor signaling pathway	1.53E-04	1.40E-03	4	42	178	27688	P0CG47|P62987|P62979|P0CG48
GO:0042060	wound healing	1.51E-03	9.68E-03	12	677	178	27688	P07602|P01033|P13929|P84243|P68871|P09493|P16615|P04275|P62158|P35613|P25116|P00441
GO:0042176	regulation of protein catabolic process	1.24E-03	8.37E-03	7	251	178	27688	P0CG47|P01033|P63244|P62987|P62979|P0CG48|P01034
GO:0042221	response to chemical	4.01E-10	7.45E-09	54	3507	178	27688	P0CG47|Q12988|P01160|P35613|P0CG48|P16860|P13073|P00505|P09493|P83731|P69905|P62158|P45379|P62753|P24752|P01033|O14880|P80297|P19021|P37108|P68032|Q08431|Q05639|P08493|P60660|P09382|P08574|P47985|Q13772|P17081|P13929|P51684|P84243|Q14192|P68871|Q9UBY9|P17174|P22352|P62979|Q01628|P00441|P51452|P02144|Q14116|O75116|P68133|P13533|P17535|P12883|Q8N8D1|P41222|P62987|P04792|P25116
GO:0042254	ribosome biogenesis	2.23E-09	3.80E-08	12	180	178	27688	P08708|P62263|P18077|P83731|P62857|P62913|P46777|P62851|P62269|P62753|P62249|P39019
GO:0042255	ribosome assembly	1.94E-07	2.76E-06	5	21	178	27688	P08708|P62263|P83731|P62851|P62753
GO:0042273	ribosomal large subunit biogenesis	7.85E-07	1.08E-05	4	12	178	27688	P18077|P83731|P62913|P46777
GO:0042274	ribosomal small subunit biogenesis	1.90E-11	3.73E-10	7	19	178	27688	P08708|P62263|P62857|P62851|P62753|P62249|P39019
GO:0042278	purine nucleoside metabolic process	1.58E-13	3.87E-12	22	446	178	27688	O00217|P13929|O75947|O95169|P51970|P48047|O43674|P04406|O14561|O00483|P16615|O43920|P36542|O95167|O95178|O96000|P25705|P18859|P07195|P56381|P06576|O15239
GO:0042451	purine nucleoside biosynthetic process	2.66E-05	2.90E-04	8	184	178	27688	O75947|P25705|P48047|P18859|P16615|P36542|P56381|P06576
GO:0042455	ribonucleoside biosynthetic process	4.65E-05	4.86E-04	8	199	178	27688	O75947|P25705|P48047|P18859|P16615|P36542|P56381|P06576
GO:0042493	response to drug	8.47E-03	3.60E-02	9	540	178	27688	P13929|P16860|P09382|P19021|P47985|P37108|P22352|P68032|P00441
GO:0042542	response to hydrogen peroxide	5.46E-04	4.34E-03	5	103	178	27688	P02144|Q14116|P68871|P69905|P00441
GO:0042692	muscle cell differentiation	3.16E-05	3.41E-04	9	245	178	27688	O15273|P10916|Q14192|P09382|P09493|P19022|P68133|P68032|P13533
GO:0042743	hydrogen peroxide metabolic process	1.26E-04	1.19E-03	4	40	178	27688	P68871|P22352|P69905|P00441
GO:0042744	hydrogen peroxide catabolic process	6.82E-04	5.23E-03	3	27	178	27688	P68871|P22352|P69905
GO:0042770	signal transduction in response to DNA damage	3.60E-03	1.89E-02	4	97	178	27688	P0CG47|P62987|P62979|P0CG48
GO:0042773	ATP synthesis coupled electron transport	1.67E-12	3.60E-11	11	74	178	27688	O00217|O95169|P51970|O43674|O14561|O00483|O43920|O95167|O95178|O96000|O15239
GO:0042775	mitochondrial ATP synthesis coupled electron transport	6.33E-13	1.45E-11	11	68	178	27688	O00217|O95169|P51970|O43674|O14561|O00483|O43920|O95167|O95178|O96000|O15239
GO:0042776	mitochondrial ATP synthesis coupled proton	2.48E-12	5.20E-11	7	15	178	27688	O75947|P25705|P48047|P18859|P36542|P56381|P06576
GO:0042981	regulation of apoptotic process	3.51E-03	1.85E-02	17	1270	178	27688	P0CG47|P01033|Q14116|P16989|P0CG48|P13693|P63244|Q05639|Q14192|P09382|P62987|P62979|P02511|P04792|P62753|P25116|P00441
GO:0043043	peptide biosynthetic process	2.35E-30	2.05E-28	43	677	178	27688	P25398|Q9NSD9|P62280|P39019|P62891|P42766|Q02878|P39023|P62273|P46783|P61247|P83731|P62857|P62913|P62979|P62851|P62753|Q13765|Q07020|P13639|P08708|P62906|P18077|P42677|P84098|P15880|Q05639|P63173|P68104|Q02543|P61353|P62263|P62987|Q14240|P62945|P46777|P05386|P60866|P46776|P62269|P05387|P60228|P62249
GO:0043066	negative regulation of apoptotic process	9.84E-04	6.93E-03	13	735	178	27688	P0CG47|P01033|P16989|P0CG48|P13693|Q05639|Q14192|P62987|P62979|P02511|P04792|P25116|P00441
GO:0043067	regulation of programmed cell death	3.88E-03	2.02E-02	17	1283	178	27688	P0CG47|P01033|Q14116|P16989|P0CG48|P13693|P63244|Q05639|Q14192|P09382|P62987|P62979|P02511|P04792|P62753|P25116|P00441
GO:0043069	negative regulation of programmed cell death	1.06E-03	7.37E-03	13	741	178	27688	P0CG47|P01033|P16989|P0CG48|P13693|Q05639|Q14192|P62987|P62979|P02511|P04792|P25116|P00441
GO:0043085	positive regulation of catalytic activity	6.42E-04	4.95E-03	15	887	178	27688	P07602|P0CG47|Q14896|P0CG48|P63244|P60033|P08590|P09493|P62987|P62979|P62158|P45379|P25116|P20585|P00441

GO:0043086	negative regulation of catalytic activity	3.45E-03	1.83E-02	10	559	178	27688	P0CG47|P51452|P63244|P19429|P62987|P62979|P04792|P45379|P0CG48|P01034
GO:0043122	regulation of I-kappaB kinase/NF-kappaB signaling	4.88E-04	3.94E-03	7	214	178	27688	P0CG47|P09382|P62987|P62979|P04792|P25116|P0CG48
GO:0043123	positive regulation of I-kappaB kinase/NF-kappaB	1.09E-03	7.55E-03	6	179	178	27688	P0CG47|P09382|P62987|P62979|P25116|P0CG48
GO:0043170	macromolecule metabolic process	1.20E-02	4.80E-02	62	7451	178	27688	P0CG47|Q9NSD9|P40926|P62280|P0CG48|P39019|P39023|P62273|P46783|P83731|P62913|P62158|P62753|Q13765|Q07020|P13639|P04406|P19021|Q9Y5N6|P84098|Q05639|P63173|P68104|P61353|P46777|P05386|P60866|P46776|P05387|P60228|P62249|P25398|P68363|P62891|P42766|Q02878|P61247|P62857|Q16654|P62979|P62851|P00441|P51452|Q14116|P08708|O75116|P62906|P18077|Q8N8D1|P42677|P15880|Q02543|Q9Y3D2|P62263|P62987|Q14240|P62945|P04275|P04792|P62269|P25116|P20585
GO:0043241	protein complex disassembly	3.97E-50	1.24E-47	36	124	178	27688	P25398|P62280|P39019|P62891|P42766|Q02878|P39023|P62273|P46783|P61247|P83731|P62857|P62913|P62979|P62851|P62753|Q07020|P08708|P62906|P18077|P42677|P84098|P15880|P63173|Q02543|P61353|P62263|P62987|P62945|P46777|P05386|P60866|P46776|P62269|P05387|P62249
GO:0043405	regulation of MAP kinase activity	1.19E-03	8.04E-03	8	322	178	27688	P0CG47|P51452|P60033|P62987|P62979|P25116|P00441|P0CG48
GO:0043406	positive regulation of MAP kinase activity	2.27E-03	1.31E-02	6	207	178	27688	P0CG47|P60033|P62987|P62979|P00441|P0CG48
GO:0043408	regulation of MAPK cascade	7.55E-03	3.27E-02	9	530	178	27688	P07602|P0CG47|P51452|P60033|P62987|P62979|P25116|P00441|P0CG48
GO:0043410	positive regulation of MAPK cascade	1.26E-02	4.91E-02	7	385	178	27688	P0CG47|P60033|P62987|P62979|P25116|P00441|P0CG48
GO:0043434	response to peptide hormone	7.28E-03	3.18E-02	9	527	178	27688	P01033|Q14116|P16860|P01160|P17174|P08574|P17081|P62753|P35613
GO:0043436	oxoacid metabolic process	7.04E-04	5.38E-03	20	1399	178	27688	P13929|P06732|Q9NSD9|P40926|P04406|O14561|P40925|O14880|P51857|P35613|P41222|P17540|P00505|P07195|P17174|Q16654|Q16698|P24752|Q13011|P48735
GO:0043462	regulation of ATPase activity	6.34E-11	1.23E-09	7	22	178	27688	Q14896|P19429|P08590|P09493|P45379|P13533|P63316
GO:0043603	cellular amide metabolic process	9.34E-27	6.56E-25	46	985	178	27688	P25398|Q9NSD9|P62280|P39019|P62891|P42766|Q02878|P39023|P62273|P46783|P61247|P83731|P62857|P62913|P22352|P62979|P62851|P62753|P00441|Q13765|Q07020|P13639|P08708|P62906|P19021|P18077|P42677|P84098|P15880|Q05639|P63173|P68104|Q02543|P61353|P62263|P62987|Q14240|P62945|P46777|P05386|P60866|P46776|P62269|P05387|P60228|P62249
GO:0043604	amide biosynthetic process	4.48E-28	3.36E-26	43	770	178	27688	P25398|Q9NSD9|P62280|P39019|P62891|P42766|Q02878|P39023|P62273|P46783|P61247|P83731|P62857|P62913|P62979|P62851|P62753|Q13765|Q07020|P13639|P08708|P62906|P18077|P42677|P84098|P15880|Q05639|P63173|P68104|Q02543|P61353|P62263|P62987|Q14240|P62945|P46777|P05386|P60866|P46776|P62269|P05387|P60228|P62249
GO:0043624	cellular protein complex disassembly	7.68E-53	2.79E-50	36	107	178	27688	P25398|P62280|P39019|P62891|P42766|Q02878|P39023|P62273|P46783|P61247|P83731|P62857|P62913|P62979|P62851|P62753|Q07020|P08708|P62906|P18077|P42677|P84098|P15880|P63173|Q02543|P61353|P62263|P62987|P62945|P46777|P05386|P60866|P46776|P62269|P05387|P62249
GO:0043648	dicarboxylic acid metabolic process	8.66E-04	6.35E-03	5	114	178	27688	P00505|P40926|P40925|P17174|P48735
GO:0043933	macromolecular complex subunit organization	2.95E-19	1.19E-17	50	1794	178	27688	P62280|P39019|P39023|P62273|P46783|P83731|P62913|P69905|P62753|P24752|Q07020|O43920|P68032|P84098|P63173|P61353|P46777|P02511|P05386|P60866|P46776|P05387|P62249|P25398|P68363|P62891|P42766|Q02878|P84243|P68871|P61247|P62857|P22352|Q16698|P62979|P62851|O00217|O15273|P08708|P62906|P68133|P18077|P42677|P15880|Q02543|P62263|P62987|P62945|P04275|P62269
GO:0044033	multi-organism metabolic process	4.71E-57	3.42E-54	36	86	178	27688	P25398|P62280|P39019|P62891|P42766|Q02878|P39023|P62273|P46783|P61247|P83731|P62857|P62913|P62979|P62851|P62753|Q07020|P08708|P62906|P18077|P42677|P84098|P15880|P63173|Q02543|P61353|P62263|P62987|P62945|P46777|P05386|P60866|P46776|P62269|P05387|P62249
GO:0044057	regulation of system process	1.45E-08	2.28E-07	15	362	178	27688	Q14896|P10916|P17661|P13533|P12883|P16860|P19429|Q9UBY9|P08590|P09493|P45379|P25116|O14558|P00441|P63316
GO:0044085	cellular component biogenesis	5.26E-07	7.30E-06	31	1810	178	27688	P10916|P68363|P39019|P84243|P68871|P09493|P83731|P62857|P62913|P22352|Q16698|P69905|P62851|P62753|P24752|O00217|Q14315|O15273|P08708|P19022|P68133|P18077|O43920|P68032|P13533|P62263|P46777|P02511|P04275|P62269|P62249
GO:0044092	negative regulation of molecular function	6.16E-03	2.83E-02	11	706	178	27688	P0CG47|P51452|P63244|P19429|P62987|P62979|P62158|P04792|P45379|P0CG48|P01034
GO:0044093	positive regulation of molecular function	4.16E-03	2.11E-02	15	1076	178	27688	P07602|P0CG47|Q14896|P0CG48|P63244|P60033|P08590|P09493|P62987|P62979|P62158|P45379|P25116|P20585|P00441
GO:0044237	cellular metabolic process	2.81E-22	1.49E-20	120	8856	178	27688	O75947|O43674|P16615|O96000|P16860|P13073|O14949|P07195|P46783|P83731|P62913|P62753|P24752|O15239|P20674|Q13765|O95169|P13639|P48047|P04406|O14561|Q9Y5N6|O95167|P84098|P63173|P68104|P08574|P47985|P46777|P60866|P46776|P60228|P62249|Q13011|Q8N335|P07602|P25398|P68363|P10176|O95178|P12235|P62891|P25705|P18859|Q14192|P68871|Q16654|P56381|P00441|P54368|O00217|Q08493|P08708|O75116|P18077|Q8N8D1|P42677|P41222|P15880|P17540|Q02543|Q9Y3D2|P62263|Q14240|P62945|P62269|P0CG47|P06732|Q9NSD9|P01160|P40926|P40925|P62280|P36542|P35613|P0CG48|P39019|P00505|P39023|P62273|P24310|P69905|P62158|P24311|Q07020|P10606|O14880|P19021|O43920|Q05639|P61353|P05386|P05387|P13929|P09669|O00483|P14854|P42766|Q02878|P60033|Q9NPI5|P17174|P61247|P62857|P22352|Q16698|P62979|P62851|P51452|P51970|P62906|P15954|P51857|Q00325|P62987|P04792|P25116|P20585|P06576|P48735
GO:0044238	primary metabolic process	1.87E-09	3.21E-08	104	10090	178	27688	O75947|O43674|P16615|O96000|P16860|P07195|P46783|P83731|P62913|P62753|P24752|O15239|Q13765|O95169|P13639|P48047|P04406|O14561|Q9Y5N6|O95167|P84098|P63173|P68104|P46777|P60866|P46776|P60228|P62249|Q13011|Q8N335|P07602|P25398|P68363|O95178|P62891|P25705|P18859|Q14192|Q16654|P56381|P00441|O00217|Q14116|Q08493|P08708|O75116|P18077|Q8N8D1|P42677|P41222|P15880|P17540|Q02543|Q9Y3D2|P62263|Q14240|P62945|P62269|P0CG47|P06732|Q9NSD9|P01160|P40926|P40925|P62280|P36542|P0CG48|P39019|P05090|P00505|P39023|P62273|P62158|Q07020|O14880|P19021|O43920|Q05639|P61353|P05386|P05387|P13929|O00483|P42766|Q02878|P60033|Q9NPI5|P17174|P61247|P62857|Q16698|P62979|P62851|P51452|P51970|P62906|P51857|P62987|P04275|P04792|P25116|P20585|P06576|P48735
GO:0044246	regulation of multicellular organismal metabolic	1.99E-04	1.76E-03	3	18	178	27688	Q14116|P25116|P01034
GO:0044248	cellular catabolic process	1.88E-03	1.14E-02	18	1301	178	27688	P0CG47|Q08493|P51857|P0CG48|P00505|P68871|P17174|P62987|Q14240|P22352|Q16698|P62979|P69905|P62158|P60228|P24752|Q13011|P00441
GO:0044249	cellular biosynthetic process	4.55E-18	1.74E-16	65	3216	178	27688	O75947|Q9NSD9|P01160|P16615|P62280|P36542|P39019|P16860|P00505|P39023|P62273|P46783|P83731|P62913|P62753|P24752|Q13765|Q07020|P13639|P48047|O14561|O14880|Q9Y5N6|P84098|Q05639|P63173|P68104|P61353|P46777|P05386|P60866|P46776|P05387|P60228|P62249|P25398|P62891|P42766|Q02878|P25705|P18859|P60033|Q9NPI5|P17174|P61247|P62857|P62979|P56381|P62851|P00441|P54368|P08708|P62906|P18077|P51857|P42677|P41222|P15880|Q02543|P62263|P62987|Q14240|P62945|P62269|P06576
GO:0044260	cellular macromolecule metabolic process	6.27E-05	6.35E-04	60	5842	178	27688	P0CG47|Q9NSD9|P40926|P62280|P0CG48|P39019|P39023|P62273|P46783|P83731|P62913|P62158|P62753|Q13765|Q07020|P13639|P04406|P19021|Q9Y5N6|P84098|Q05639|P63173|P68104|P61353|P46777|P05386|P60866|P46776|P05387|P60228|P62249|P25398|P68363|P62891|P42766|Q02878|P61247|P62857|Q16654|P62979|P62851|P00441|P51452|P08708|O75116|P62906|P18077|Q8N8D1|P42677|P15880|Q02543|Q9Y3D2|P62263|P62987|Q14240|P62945|P04792|P62269|P25116|P20585
GO:0044262	cellular carbohydrate metabolic process	1.00E-02	4.13E-02	5	202	178	27688	P40926|P07195|P40925|P62158|P48735

GO:0044267	cellular protein metabolic process	2.11E-10	4.00E-09	54	3447	178	27688	P0CG47|Q9NSD9|P40926|P62280|P0CG48|P39019|P39023|P62273|P46783|P83731|P62913|P62753|Q13765|Q07020|P13639|P04406|P19021|P84098|Q05639|P63173|P68104|P61353|P46777|P05386|P60866|P46776|P05387|P60228|P62249|P25398|P68363|P62891|P42766|Q02878|P61247|P62857|Q16654|P62979|P62851|P51452|P08708|O75116|P62906|P18077|P42677|P15880|Q02543|Q9Y3D2|P62263|P62987|Q14240|P62945|P62269|P25116
GO:0044271	cellular nitrogen compound biosynthetic process	2.76E-22	1.49E-20	55	1879	178	27688	O75947|Q9NSD9|P01160|P16615|P62280|P36542|P39019|P16860|P39023|P62273|P46783|P83731|P62913|P62753|Q13765|Q07020|P13639|P48047|P84098|Q05639|P63173|P68104|P61353|P46777|P05386|P60866|P46776|P05387|P60228|P62249|P25398|P62891|P42766|Q02878|P25705|P18859|Q9NPI5|P61247|P62857|P62979|P56381|P62851|P54368|P08708|P62906|P18077|P42677|P15880|Q02543|P62263|P62987|Q14240|P62945|P62269|P06576
GO:0044281	small molecule metabolic process	5.11E-09	8.24E-08	43	2596	178	27688	P13929|O75947|P06732|Q9NSD9|P01160|O43674|P40926|P40925|O00483|P16615|P36542|O95178|P35613|O96000|P16860|P25705|P00505|P18859|Q9NPI5|P07195|P17174|Q16654|Q16698|P56381|P62158|P24752|O15239|O00217|Q08493|O95169|P51970|P48047|P04406|O14561|O14880|O43920|P51857|O95167|P41222|P17540|Q13011|P06576|P48735
GO:0044283	small molecule biosynthetic process	8.96E-04	6.50E-03	11	549	178	27688	P13929|P41222|P00505|P40926|P04406|O14561|P40925|O14880|P17174|P51857|P24752
GO:0044403	symbiosis, encompassing mutualism through parasitism	2.34E-32	2.68E-30	40	492	178	27688	P0CG47|P25398|P62280|P12235|P0CG48|P39019|P62891|P42766|Q02878|P60033|P39023|P62273|P46783|P61247|P83731|P62857|P62913|P62979|P62851|P62753|Q07020|P08708|P62906|P18077|P42677|P84098|P15880|P63173|Q02543|P61353|P62263|P62987|P62945|P46777|P05386|P60866|P46776|P62269|P05387|P62249
GO:0044419	interspecies interaction between organisms	6.70E-29	5.40E-27	44	781	178	27688	P0CG47|P25398|P62280|P12235|P0CG48|P39019|P62891|P42766|P63244|Q02878|P60033|P39023|P62273|P46783|P61247|P83731|P62857|P62913|P62979|P62851|P62753|Q13765|Q07020|P08708|P62906|P18077|Q08431|P42677|P84098|P15880|P63173|Q02543|P61353|P62263|P62987|Q14240|P62945|P46777|P05386|P60866|P46776|P62269|P05387|P62249
GO:0044699	single-organism process	1.49E-12	3.27E-11	155	17596	178	27688	O75947|P10916|O43674|Q9H3K2|P16615|O96000|P16860|P13073|O14949|P09493|P07195|P46783|P83731|P62913|P62753|P24752|O15239|P20674|O95169|P48047|Q9Y277|P04406|O14561|Q9Y5N6|P16989|O95167|Q08431|P19105|P84098|O14958|P63173|P09382|P08574|P47985|P46777|Q13772|P60866|P46776|P62249|Q13011|P07602|P25398|P68363|P10176|O95178|P12235|P13693|P62891|P19429|P25705|P18859|Q14192|P68871|Q16654|P56381|Q01628|P00441|O00217|Q14116|O15273|Q08493|P08708|Q14515|O75116|P68133|P18077|P17661|P17535|P12883|Q8N8D1|P42677|P41222|P15880|P17540|Q02543|P62263|P62945|P62269|P0CG47|P06732|Q9NSD9|Q12988|P01160|P40926|P40925|P62280|P36542|P35613|P0CG48|P39019|P63244|P05090|Q01449|Q14094|P00505|P39023|P62273|P69905|P62158|P45379|P24311|P01034|Q07020|P01033|P10606|Q14896|O14880|P19021|P19022|P37108|O43920|P68032|P08493|P61353|P60660|P05386|P17081|P05387|P13929|P02792|P09669|P51684|O00483|P14854|P42766|Q02878|P84243|P60033|Q9NPI5|P17174|P61247|P62857|P22352|Q16698|P62979|P62851|P51452|P02144|Q14315|P51970|Q9P0U1|P62906|P15954|P51857|P13533|P08590|P62987|P04275|P63313|P04792|P25116|P20585|P06576|P63316|P48735
GO:0044707	single-multicellular organism process	6.41E-15	1.89E-13	84	5832	178	27688	P10916|P16615|P62280|P35613|P39019|P63244|P16860|O14949|P39023|P62273|P09493|P46783|P83731|P62913|P62158|P45379|P62753|P24752|Q07020|P01033|P10606|Q14896|O14880|P19021|P19022|P16989|P68032|Q08431|P84098|O14958|P63173|P08493|P61353|P60660|P09382|P46777|P05386|Q13772|P60866|P46776|P05387|P62249|P07602|P25398|P62891|P42766|Q02878|P19429|P25705|P84243|Q14192|P68871|P61247|P62857|P62979|P62851|P00441|P51452|P02144|Q14116|O15273|P08708|O75116|P62906|P68133|P18077|P51857|P13533|P17535|P12883|P42677|P15880|Q02543|P08590|P62263|P62987|P62945|P04275|P04792|P62269|P25116|P20585|P06576|P63316
GO:0044710	single-organism metabolic process	1.74E-09	3.00E-08	70	5507	178	27688	P0CG47|O75947|P06732|Q9NSD9|P01160|O43674|P40926|P40925|P16615|P36542|P35613|P0CG48|O96000|P16860|P05090|P13073|P00505|O14949|P07195|P69905|P62158|P24311|P24752|O15239|P20674|P10606|O95169|P48047|P04406|O14561|O14880|O43920|O95167|P08574|P47985|Q13011|P07602|P13929|P09669|O00483|P14854|P10176|O95178|P12235|P25705|P18859|P60033|Q14192|P68871|Q9NPI5|P17174|Q16654|P22352|Q16698|P62979|P56381|P00441|P51452|O00217|Q08493|P51970|P15954|P51857|P41222|P17540|P62987|P04275|P20585|P06576|P48735
GO:0044711	single-organism biosynthetic process	1.01E-04	9.86E-04	23	1501	178	27688	P13929|O75947|P48047|P01160|P40926|P04406|O14561|P40925|O14880|P16615|P36542|P51857|P16860|P41222|P25705|P00505|P18859|P60033|Q9NPI5|P17174|P56381|P24752|P06576
GO:0044712	single-organism catabolic process	9.51E-04	6.74E-03	14	826	178	27688	P13929|Q08493|P04406|P51857|P00505|P68871|P07195|P17174|P22352|Q16698|P69905|P62158|P24752|Q13011
GO:0044763	single-organism cellular process	8.67E-17	3.15E-15	147	14664	178	27688	O75947|P10916|O43674|Q9H3K2|P16615|O96000|P16860|P13073|O14949|P09493|P07195|P46783|P83731|P62913|P62753|P24752|O15239|P20674|O95169|P48047|Q9Y277|P04406|O14561|Q9Y5N6|O95167|Q08431|P19105|P84098|P63173|P09382|P08574|P47985|P46777|Q13772|P60866|P46776|P62249|Q13011|P07602|P25398|P68363|P10176|O95178|P12235|P13693|P62891|P19429|P25705|P18859|Q14192|P68871|Q16654|P56381|Q01628|P00441|O00217|Q14116|O15273|Q08493|P08708|Q14515|O75116|P68133|P18077|P17661|P17535|P12883|Q8N8D1|P42677|P41222|P15880|P17540|Q02543|P62263|P62945|P62269|P0CG47|P06732|Q9NSD9|Q12988|P01160|P40926|P40925|P62280|P36542|P35613|P0CG48|P39019|Q01449|P00505|P39023|P62273|P69905|P62158|P45379|P24311|P01034|Q07020|P01033|P10606|Q14896|O14880|P19022|P37108|O43920|P68032|P08493|P61353|P60660|P05386|P17081|P05387|P13929|P02792|P09669|P51684|O00483|P14854|P42766|Q02878|P60033|Q9NPI5|P17174|P61247|P62857|P22352|Q16698|P62979|P62851|P51452|P02144|Q14315|P51970|P62906|P15954|P51857|P13533|P08590|P62987|P04275|P63313|P04792|P25116|P20585|P06576|P63316|P48735
GO:0044764	multi-organism cellular process	2.14E-33	2.59E-31	40	463	178	27688	P0CG47|P25398|P62280|P12235|P0CG48|P39019|P62891|P42766|Q02878|P60033|P39023|P62273|P46783|P61247|P83731|P62857|P62913|P62979|P62851|P62753|Q07020|P08708|P62906|P18077|P42677|P84098|P15880|P63173|Q02543|P61353|P62263|P62987|P62945|P46777|P05386|P60866|P46776|P62269|P05387|P62249
GO:0044765	single-organism transport	1.12E-02	4.54E-02	35	3688	178	27688	P07602|P0CG47|O75947|P02792|P68363|P16615|P36542|P35613|P12235|P0CG48|P39019|P13693|P16860|P05090|P25705|P00505|P18859|P68871|P62913|P62979|P56381|P69905|P62158|P00441|P01033|Q9P0U1|P48047|Q9Y277|P37108|Q08431|P19105|P62987|P04275|P25116|P06576
GO:0044767	single-organism developmental process	6.84E-13	1.55E-11	78	5585	178	27688	P10916|P62280|P35613|P39019|P63244|P16860|O14949|P39023|P62273|P09493|P46783|P83731|P62913|P45379|P62753|P24752|Q07020|P01033|Q14896|P19021|P19022|P16989|P68032|Q08431|P84098|O14958|P63173|P08493|P61353|P60660|P09382|P46777|P05386|Q13772|P60866|P46776|P05387|P62249|P07602|P13929|P25398|P62891|P42766|Q02878|P19429|P25705|P84243|Q14192|P61247|P62857|P62979|P62851|P00441|P51452|P02144|Q14116|O15273|P08708|O75116|P62906|P68133|P18077|P13533|P17535|P12883|P42677|P15880|Q02543|P08590|P62263|P62987|P62945|P04275|P04792|P62269|P20585|P06576|P63316
GO:0044770	cell cycle phase transition	1.16E-02	4.67E-02	6	291	178	27688	P0CG47|P62987|Q9Y5N6|P83731|P62979|P0CG48
GO:0044772	mitotic cell cycle phase transition	1.16E-02	4.67E-02	6	291	178	27688	P0CG47|P62987|Q9Y5N6|P83731|P62979|P0CG48

<table>
<tr><th></th><th></th><th></th><th></th><th></th><th></th><th></th><th></th><th></th></tr>
<tr><td>GO:0044773</td><td>mitotic DNA damage checkpoint</td><td>2.97E-03</td><td>1.62E-02</td><td>4</td><td>92</td><td>178</td><td>27688</td><td>P0CG47|P62987|P62979|P0CG48</td></tr>
<tr><td>GO:0044774</td><td>mitotic DNA integrity checkpoint</td><td>3.60E-03</td><td>1.89E-02</td><td>4</td><td>97</td><td>178</td><td>27688</td><td>P0CG47|P62987|P62979|P0CG48</td></tr>
<tr><td>GO:0044783</td><td>G1 DNA damage checkpoint</td><td>1.27E-03</td><td>8.50E-03</td><td>4</td><td>73</td><td>178</td><td>27688</td><td>P0CG47|P62987|P62979|P0CG48</td></tr>
<tr><td>GO:0044819</td><td>mitotic G1/S transition checkpoint</td><td>1.27E-03</td><td>8.50E-03</td><td>4</td><td>73</td><td>178</td><td>27688</td><td>P0CG47|P62987|P62979|P0CG48</td></tr>
<tr><td>GO:0044843</td><td>cell cycle G1/S phase transition</td><td>4.58E-03</td><td>2.28E-02</td><td>5</td><td>167</td><td>178</td><td>27688</td><td>P0CG47|P62987|Q9Y5N6|P62979|P0CG48</td></tr>
<tr><td>GO:0045089</td><td>positive regulation of innate immune response</td><td>4.70E-03</td><td>2.32E-02</td><td>5</td><td>168</td><td>178</td><td>27688</td><td>P0CG47|P51452|P62987|P62979|P0CG48</td></tr>
<tr><td>GO:0045214</td><td>sarcomere organization</td><td>6.09E-04</td><td>4.74E-03</td><td>3</td><td>26</td><td>178</td><td>27688</td><td>O15273|P09493|P13533</td></tr>
<tr><td>GO:0045333</td><td>cellular respiration</td><td>5.13E-35</td><td>6.99E-33</td><td>32</td><td>206</td><td>178</td><td>27688</td><td>O75947|P09669|O43674|P40926|P40925|O00483|P14854|P36542|P10176|O95178|O96000|P13073|P25705|P18859|O14949|P56381|P24311|O15239|P20674|O00217|P10606|O95169|P51970|P48047|O14561|O43920|P15954|O95167|P08574|P47985|P06576|P48735</td></tr>
<tr><td>GO:0045732</td><td>positive regulation of protein catabolic process</td><td>2.82E-03</td><td>1.56E-02</td><td>5</td><td>149</td><td>178</td><td>27688</td><td>P0CG47|P63244|P62987|P62979|P0CG48</td></tr>
<tr><td>GO:0045787</td><td>positive regulation of cell cycle</td><td>4.89E-03</td><td>2.40E-02</td><td>6</td><td>242</td><td>178</td><td>27688</td><td>P0CG47|P51452|O75116|P62987|P62979|P0CG48</td></tr>
<tr><td>GO:0045859</td><td>regulation of protein kinase activity</td><td>9.52E-03</td><td>3.98E-02</td><td>10</td><td>649</td><td>178</td><td>27688</td><td>P0CG47|P51452|P63244|P60033|P62987|P62979|P04792|P25116|P00441|P0CG48</td></tr>
<tr><td>GO:0045860</td><td>positive regulation of protein kinase activity</td><td>9.85E-03</td><td>4.07E-02</td><td>7</td><td>367</td><td>178</td><td>27688</td><td>P0CG47|P60033|P62987|P62979|P25116|P00441|P0CG48</td></tr>
<tr><td>GO:0045862</td><td>positive regulation of proteolysis</td><td>1.52E-03</td><td>9.70E-03</td><td>7</td><td>260</td><td>178</td><td>27688</td><td>P0CG47|P63244|Q9NWT8|P62987|P62979|P25116|P0CG48</td></tr>
<tr><td>GO:0045930</td><td>negative regulation of mitotic cell cycle</td><td>8.59E-04</td><td>6.34E-03</td><td>6</td><td>171</td><td>178</td><td>27688</td><td>P0CG47|Q9NWT8|P62987|P83731|P62979|P0CG48</td></tr>
<tr><td>GO:0046031</td><td>ADP metabolic process</td><td>1.40E-03</td><td>9.28E-03</td><td>5</td><td>127</td><td>178</td><td>27688</td><td>P13929|P25705|P04406|P07195|P06576</td></tr>
<tr><td>GO:0046034</td><td>ATP metabolic process</td><td>2.22E-16</td><td>7.81E-15</td><td>22</td><td>324</td><td>178</td><td>27688</td><td>O00217|P13929|O75947|O95169|P51970|P48047|O43674|P04406|O14561|O00483|P16615|O43920|P36542|O95167|O95178|O96000|P25705|P18859|P07195|P56381|P06576|O15239</td></tr>
<tr><td>GO:0046128</td><td>purine ribonucleoside metabolic process</td><td>1.38E-13</td><td>3.46E-12</td><td>22</td><td>443</td><td>178</td><td>27688</td><td>O00217|P13929|O75947|O95169|P51970|P48047|O43674|P04406|O14561|O00483|P16615|O43920|P36542|O95167|O95178|O96000|P25705|P18859|P07195|P56381|P06576|O15239</td></tr>
<tr><td>GO:0046129</td><td>purine ribonucleoside biosynthetic process</td><td>2.66E-05</td><td>2.90E-04</td><td>8</td><td>184</td><td>178</td><td>27688</td><td>O75947|P25705|P48047|P18859|P16615|P36542|P56381|P06576</td></tr>
<tr><td>GO:0046364</td><td>monosaccharide biosynthetic process</td><td>7.73E-06</td><td>9.35E-05</td><td>6</td><td>73</td><td>178</td><td>27688</td><td>P13929|P00505|P40926|P04406|P40925|P17174</td></tr>
<tr><td>GO:0046390</td><td>ribose phosphate biosynthetic process</td><td>2.31E-06</td><td>2.99E-05</td><td>10</td><td>227</td><td>178</td><td>27688</td><td>O75947|P16860|P25705|P48047|P18859|P01160|P16615|P36542|P56381|P06576</td></tr>
<tr><td>GO:0046394</td><td>carboxylic acid biosynthetic process</td><td>9.71E-03</td><td>4.03E-02</td><td>7</td><td>366</td><td>178</td><td>27688</td><td>P41222|P00505|O14561|O14880|P17174|P51857|P24752</td></tr>
<tr><td>GO:0046395</td><td>carboxylic acid catabolic process</td><td>4.16E-03</td><td>2.11E-02</td><td>6</td><td>234</td><td>178</td><td>27688</td><td>P00505|P17174|Q16698|P51857|P24752|Q13011</td></tr>
<tr><td>GO:0046483</td><td>heterocycle metabolic process</td><td>5.23E-20</td><td>2.28E-18</td><td>75</td><td>3900</td><td>178</td><td>27688</td><td>P0CG47|O75947|Q9NSD9|P01160|O43674|P40926|P40925|P16615|P62280|P36542|P0CG48|P39019|O96000|P16860|P39023|P62273|P07195|P46783|P83731|P62913|P62753|O15239|Q13765|Q07020|O95169|P48047|P04406|O14561|Q9Y5N6|O43920|O95167|P84098|P63173|P61353|P46777|P05386|P60866|P46776|P05387|P60228|P62249|P13929|P25398|O00483|O95178|P62891|P42766|Q02878|P25705|P18859|Q9NPI5|P61247|P62857|P62979|P56381|P62851|P00441|O00217|Q08493|P51970|P08708|P62906|P18077|Q8N8D1|P42677|P15880|Q02543|P62263|P62987|Q14240|P62945|P04792|P62269|P20585|P06576</td></tr>
<tr><td>GO:0046496</td><td>nicotinamide nucleotide metabolic process</td><td>7.49E-03</td><td>3.25E-02</td><td>5</td><td>188</td><td>178</td><td>27688</td><td>P13929|P40926|P04406|P07195|P40925</td></tr>
<tr><td>GO:0046907</td><td>intracellular transport</td><td>1.88E-03</td><td>1.14E-02</td><td>19</td><td>1409</td><td>178</td><td>27688</td><td>P0CG47|O75947|P02792|Q9P0U1|P48047|P68363|P37108|P16615|P36542|P19105|P0CG48|P25705|P18859|P62987|P62913|P62979|P56381|P25116|P06576</td></tr>
<tr><td>GO:0048011</td><td>neurotrophin TRK receptor signaling pathway</td><td>2.87E-03</td><td>1.58E-02</td><td>6</td><td>217</td><td>178</td><td>27688</td><td>P0CG47|P51452|P62987|P62979|P62158|P0CG48</td></tr>
<tr><td>GO:0048513</td><td>animal organ development</td><td>4.79E-21</td><td>2.27E-19</td><td>66</td><td>2908</td><td>178</td><td>27688</td><td>P10916|P62280|P35613|P39019|P16860|O14949|P39023|P62273|P09493|P46783|P83731|P62913|P45379|P62753|P24752|Q07020|P01033|Q14896|P19021|P16989|P68032|P84098|O14958|P63173|P08493|P61353|P60660|P46777|P05386|Q13772|P60866|P46776|P05387|P62249|P07602|P25398|P62891|P42766|Q02878|P19429|P84243|Q14192|P61247|P62857|P62979|P62851|P00441|P02144|Q14116|O15273|P08708|P62906|P68133|P18077|P13533|P12883|P42677|P15880|Q02543|P08590|P62263|P62987|P62945|P04275|P62269|P63316</td></tr>
<tr><td>GO:0048519</td><td>negative regulation of biological process</td><td>1.80E-03</td><td>1.10E-02</td><td>39</td><td>3792</td><td>178</td><td>27688</td><td>P0CG47|P10916|P01160|P12235|P0CG48|P13693|P63244|P16860|P19429|P25705|Q14192|Q9NPI5|P09493|P83731|P62979|P62158|P45379|P00441|P01034|P51452|P01033|Q08493|O75116|P80297|P37108|P16989|Q05639|Q9NWT8|P09382|P62263|P62987|P02511|P63313|P17081|P04792|P60228|P25116|P20585|P06576</td></tr>
<tr><td>GO:0048523</td><td>negative regulation of cellular process</td><td>4.42E-03</td><td>2.22E-02</td><td>35</td><td>3471</td><td>178</td><td>27688</td><td>P0CG47|P10916|P01160|P12235|P0CG48|P13693|P63244|P16860|P25705|Q14192|Q9NPI5|P09493|P83731|P62979|P62158|P00441|P01034|P51452|P01033|Q08493|P37108|P16989|Q05639|Q9NWT8|P09382|P62263|P62987|P02511|P63313|P17081|P04792|P60228|P25116|P20585|P06576</td></tr>
<tr><td>GO:0048545</td><td>response to steroid hormone</td><td>1.07E-02</td><td>4.37E-02</td><td>9</td><td>561</td><td>178</td><td>27688</td><td>P41222|Q14192|P19021|P17174|P68133|P22352|Q13772|Q08431|Q8N8D1</td></tr>
<tr><td>GO:0048644</td><td>muscle organ morphogenesis</td><td>2.62E-13</td><td>6.19E-12</td><td>11</td><td>63</td><td>178</td><td>27688</td><td>O15273|Q14896|P10916|P19429|P08590|P09493|P68032|P45379|P13533|P12883|P63316</td></tr>
<tr><td>GO:0048729</td><td>tissue morphogenesis</td><td>5.42E-04</td><td>4.33E-03</td><td>12</td><td>601</td><td>178</td><td>27688</td><td>O15273|Q14896|P10916|P19429|P08493|P08590|P09493|P68032|P45379|P13533|P12883|P63316</td></tr>
<tr><td>GO:0048731</td><td>system development</td><td>6.13E-17</td><td>2.26E-15</td><td>73</td><td>4195</td><td>178</td><td>27688</td><td>P10916|P62280|P35613|P39019|P16860|O14949|P39023|P62273|P09493|P46783|P83731|P62913|P45379|P62753|P24752|Q07020|P01033|Q14896|P19021|P19022|P16989|P68032|Q08431|P84098|O14958|P63173|P08493|P61353|P60660|P09382|P46777|P05386|Q13772|P60866|P46776|P05387|P62249|P07602|P25398|P62891|P42766|Q02878|P19429|P84243|Q14192|P61247|P62857|P62979|P62851|P00441|P02144|Q14116|O15273|P08708|O75116|P62906|P68133|P18077|P13533|P12883|P42677|P15880|Q02543|P08590|P62263|P62987|P62945|P04275|P04792|P62269|P20585|P06576|P63316</td></tr>
<tr><td>GO:0048738</td><td>cardiac muscle tissue development</td><td>1.03E-10</td><td>1.99E-09</td><td>12</td><td>138</td><td>178</td><td>27688</td><td>O15273|Q14896|P10916|P19429|Q14192|P08590|P09493|P68032|P45379|P13533|P12883|P63316</td></tr>
<tr><td>GO:0048747</td><td>muscle fiber development</td><td>9.33E-04</td><td>6.64E-03</td><td>3</td><td>30</td><td>178</td><td>27688</td><td>O15273|P68133|P13533</td></tr>
<tr><td>GO:0048856</td><td>anatomical structure development</td><td>1.15E-16</td><td>4.09E-15</td><td>79</td><td>4893</td><td>178</td><td>27688</td><td>P10916|P16615|P62280|P35613|P39019|P16860|O14949|P39023|P62273|P09493|P46783|P83731|P62913|P45379|P62753|P24752|Q07020|P01033|Q14896|P19021|P19022|P16989|P68032|Q08431|P84098|O14958|P63173|P08493|P61353|P60660|P09382|P46777|P05386|Q13772|P60866|P46776|P05387|P62249|P07602|P13929|P25398|P62891|P42766|Q02878|P19429|P25705|P84243|Q14192|P61247|P62857|P62979|P62851|P00441|P51452|P02144|Q14116|O15273|P08708|O75116|P62906|P68133|P18077|P13533|P17535|P12883|P42677|P15880|Q02543|P08590|P62263|P62987|P62945|P04275|P04792|P62269|P25116|P20585|P06576|P63316</td></tr>
<tr><td>GO:0048872</td><td>homeostasis of number of cells</td><td>1.71E-04</td><td>1.54E-03</td><td>7</td><td>180</td><td>178</td><td>27688</td><td>P01033|P02144|P08708|P62263|P25116|P00441|P39019</td></tr>
<tr><td>GO:0050790</td><td>regulation of catalytic activity</td><td>1.54E-03</td><td>9.78E-03</td><td>21</td><td>1602</td><td>178</td><td>27688</td><td>P07602|P0CG47|P51452|Q14896|P13533|P0CG48|P63244|P19429|P60033|P08590|P09493|P62987|P62979|P62158|P04792|P45379|P25116|P20585|P00441|P63316|P01034</td></tr>
<tr><td>GO:0050817</td><td>coagulation</td><td>2.00E-03</td><td>1.19E-02</td><td>10</td><td>518</td><td>178</td><td>27688</td><td>P07602|P01033|P84243|P68871|P16615|P04275|P62158|P35613|P25116|P00441</td></tr>
<tr><td>GO:0050851</td><td>antigen receptor-mediated signaling pathway</td><td>1.17E-02</td><td>4.71E-02</td><td>4</td><td>136</td><td>178</td><td>27688</td><td>P0CG47|P62987|P62979|P0CG48</td></tr>
<tr><td>GO:0050852</td><td>T cell receptor signaling pathway</td><td>5.62E-03</td><td>2.70E-02</td><td>4</td><td>110</td><td>178</td><td>27688</td><td>P0CG47|P62987|P62979|P0CG48</td></tr>
<tr><td>GO:0050878</td><td>regulation of body fluid levels</td><td>5.22E-03</td><td>2.53E-02</td><td>11</td><td>690</td><td>178</td><td>27688</td><td>P07602|P01033|P16860|P84243|P68871|P16615|P04275|P62158|P35613|P25116|P00441</td></tr>
<tr><td>GO:0050880</td><td>regulation of blood vessel size</td><td>2.29E-03</td><td>1.31E-02</td><td>5</td><td>142</td><td>178</td><td>27688</td><td>P16860|P01160|P68871|P25116|P00441</td></tr>
<tr><td>GO:0051092</td><td>positive regulation of NF-kappaB transcription</td><td>2.43E-03</td><td>1.37E-02</td><td>4</td><td>87</td><td>178</td><td>27688</td><td>P0CG47|P62987|P62979|P0CG48</td></tr>
<tr><td>GO:0051146</td><td>striated muscle cell differentiation</td><td>5.96E-05</td><td>6.09E-04</td><td>7</td><td>152</td><td>178</td><td>27688</td><td>O15273|P10916|Q14192|P09493|P68133|P68032|P13533</td></tr>
<tr><td>GO:0051179</td><td>localization</td><td>2.83E-04</td><td>2.42E-03</td><td>58</td><td>5886</td><td>178</td><td>27688</td><td>P0CG47|O75947|O43674|P16615|P36542|P35613|P0CG48|P39019|O96000|P16860|P05090|P00505|O14949|P62913|P69905|P62158|O15239|Q13765|P01033|O95169|P48047|Q9Y277|O14561|P19022|P37108|O43920|O95167|Q08431|P19105|P08574|P47985|P07602|P02792|P51684|P68363|O00483|O95178|P12235|P13693|P25705|P18859|P60033|P68871|P62979|P56381|P00441|O00217|P02144|P51970|Q9P0U1|P41222|Q00325|P62987|P04275|P63313|P04792|P25116|P06576</td></tr>
</table>

GO:0051234	establishment of localization	1.66E-04	1.50E-03	53	5116	178	27688	P0CG47|O75947|O43674|P16615|P36542|P35613|P0CG48|P39019|O96000|P16860|P05090|P00505|O14949|P62913|P69905|P62158|O15239|Q13765|P01033|O95169|P48047|Q9Y277|O14561|P37108|O43920|O95167|Q08431|P19105|P08574|P47985|P07602|P02792|P68363|O00483|O95178|P12235|P13693|P25705|P18859|P68871|P62979|P56381|P00441|O00217|P02144|P51970|Q9P0U1|P41222|Q00325|P62987|P04275|P25116|P06576
GO:0051239	regulation of multicellular organismal process	1.52E-05	1.71E-04	30	2027	178	27688	P0CG47|P10916|P0CG48|P63244|P16860|P19429|Q9UBY9|P09493|P62979|P45379|P00441|P01034|Q14116|Q14896|O75116|P19022|P16989|P17661|P13533|P17535|P12883|P41222|P08493|P09382|P08590|P62987|P04792|P25116|O14558|P63316
GO:0051248	negative regulation of protein metabolic process	9.20E-03	3.89E-02	11	747	178	27688	P0CG47|P51452|P01033|P63244|P62987|P37108|P62979|P04792|P60228|P0CG48|P01034
GO:0051259	protein oligomerization	3.97E-03	2.06E-02	7	309	178	27688	P68871|P22352|Q16698|P02511|P04275|P69905|P24752
GO:0051260	protein homooligomerization	8.16E-03	3.49E-02	5	192	178	27688	P22352|Q16698|P02511|P04275|P24752
GO:0051320	S phase	1.17E-03	7.98E-03	5	122	178	27688	P0CG47|P62987|Q9Y5N6|P62979|P0CG48
GO:0051325	interphase	3.34E-03	1.79E-02	5	155	178	27688	P0CG47|P62987|Q9Y5N6|P62979|P0CG48
GO:0051329	mitotic interphase	2.90E-03	1.59E-02	5	150	178	27688	P0CG47|P62987|Q9Y5N6|P62979|P0CG48
GO:0051336	regulation of hydrolase activity	4.46E-03	2.23E-02	12	774	178	27688	P63244|Q14896|P19429|P08590|P09493|P62158|P45379|P13533|P25116|P20585|P63316|P01034
GO:0051340	regulation of ligase activity	4.93E-03	2.41E-02	4	106	178	27688	P0CG47|P62987|P62979|P0CG48
GO:0051345	positive regulation of hydrolase activity	4.43E-03	2.22E-02	8	399	178	27688	P63244|Q14896|P08590|P09493|P62158|P45379|P25116|P20585
GO:0051348	negative regulation of transferase activity	2.22E-03	1.28E-02	7	278	178	27688	P0CG47|P51452|P63244|P62987|P62979|P04792|P0CG48
GO:0051351	positive regulation of ligase activity	2.14E-03	1.25E-02	4	84	178	27688	P0CG47|P62987|P62979|P0CG48
GO:0051352	negative regulation of ligase activity	1.96E-03	1.17E-02	4	82	178	27688	P0CG47|P62987|P62979|P0CG48
GO:0051384	response to glucocorticoid	1.19E-02	4.79E-02	5	211	178	27688	P41222|P19021|P17174|P22352|Q8N8D1
GO:0051403	stress-activated MAPK cascade	1.61E-03	1.01E-02	5	131	178	27688	P0CG47|P51452|P62987|P62979|P0CG48
GO:0051436	negative regulation of ubiquitin-protein ligase activity involved in mitotic cell cycle	1.48E-03	9.54E-03	4	76	178	27688	P0CG47|P62987|P62979|P0CG48
GO:0051437	positive regulation of ubiquitin-protein ligase activity involved in regulation of mitotic cell cycle	1.21E-03	8.17E-03	4	72	178	27688	P0CG47|P62987|P62979|P0CG48
GO:0051438	regulation of ubiquitin-protein transferase activity	4.93E-03	2.41E-02	4	106	178	27688	P0CG47|P62987|P62979|P0CG48
GO:0051439	regulation of ubiquitin-protein ligase activity involved in mitotic cell cycle	2.43E-03	1.37E-02	4	87	178	27688	P0CG47|P62987|P62979|P0CG48
GO:0051443	positive regulation of ubiquitin-protein	1.79E-03	1.10E-02	4	80	178	27688	P0CG47|P62987|P62979|P0CG48
GO:0051444	negative regulation of ubiquitin-protein	1.96E-03	1.17E-02	4	82	178	27688	P0CG47|P62987|P62979|P0CG48
GO:0051592	response to calcium ion	5.97E-04	4.67E-03	5	105	178	27688	P16860|P08493|P62158|P45379|P17535
GO:0051641	cellular localization	6.34E-04	4.91E-03	26	2041	178	27688	P07602|P0CG47|O75947|P02792|P68363|P16615|P36542|P12235|P0CG48|P25705|P18859|P62913|P62979|P56381|P62158|P00441|P01033|Q9P0U1|P48047|P37108|P19105|P62987|P04275|P63313|P25116|P06576
GO:0051649	establishment of localization in cell	2.60E-04	2.24E-03	25	1816	178	27688	P07602|P0CG47|O75947|P02792|P68363|P16615|P36542|P12235|P0CG48|P25705|P18859|P62913|P62979|P56381|P62158|P00441|P01033|Q9P0U1|P48047|P37108|P19105|P62987|P04275|P25116|P06576
GO:0051704	multi-organism process	2.54E-18	9.87E-17	54	2209	178	27688	P0CG47|P62280|P35613|P0CG48|P39019|P63244|Q14094|P39023|P62273|P46783|P83731|P62913|P62753|Q13765|Q07020|P16989|Q08431|P84098|P63173|P61353|P46777|P05386|P60866|P46776|P05387|P62249|P25398|P12235|P13693|P62891|P42766|Q02878|P60033|P61247|P62857|P22352|P62979|P62851|Q01628|P00441|Q14116|P08708|P62906|P18077|P42677|P15880|Q02543|P62263|P62987|Q14240|P62945|P04792|P62269|P25116
GO:0055001	muscle cell development	3.41E-06	4.34E-05	7	98	178	27688	O15273|P10916|Q14192|P09493|P68133|P68032|P13533
GO:0055002	striated muscle cell development	2.07E-06	2.72E-05	7	91	178	27688	O15273|P10916|Q14192|P09493|P68133|P68032|P13533
GO:0055003	cardiac myofibril assembly	2.35E-04	2.08E-03	3	19	178	27688	O15273|P10916|P68032
GO:0055006	cardiac cell development	9.20E-06	1.09E-04	5	44	178	27688	O15273|P10916|Q14192|P68032|P13533
GO:0055007	cardiac muscle cell differentiation	2.37E-04	2.09E-03	5	86	178	27688	O15273|P10916|Q14192|P68032|P13533
GO:0055008	cardiac muscle tissue morphogenesis	1.34E-14	3.69E-13	11	49	178	27688	O15273|Q14896|P10916|P19429|P08590|P09493|P68032|P45379|P13533|P12883|P63316
GO:0055010	ventricular cardiac muscle tissue morphogenesis	1.63E-12	3.55E-11	9	37	178	27688	Q14896|P10916|P19429|P08590|P09493|P45379|P13533|P12883|P63316
GO:0055013	cardiac muscle cell development	9.20E-06	1.09E-04	5	44	178	27688	O15273|P10916|Q14192|P68032|P13533
GO:0055082	cellular chemical homeostasis	1.01E-02	4.15E-02	8	460	178	27688	P13693|P02792|P51684|P19429|P09382|P25116|P00441|P06576
GO:0055086	nucleobase-containing small molecule metabolic process	8.87E-13	1.97E-11	28	837	178	27688	P13929|O75947|P01160|O43674|P40926|P40925|O00483|P16615|P36542|O95178|O96000|P16860|P25705|P18859|Q9NPI5|P07195|P56381|O15239|O00217|Q08493|O95169|P51970|P48047|P04406|O14561|O43920|O95167|P06576
GO:0055114	oxidation-reduction process	1.59E-20	7.20E-19	37	845	178	27688	O75947|P09669|O43674|P40926|P40925|O00483|P14854|P36542|P10176|O95178|P12235|O96000|P13073|P25705|P18859|O14949|Q16698|P56381|P62158|P24311|O15239|P20674|O00217|P10606|O95169|P51970|P48047|O14561|O43920|P15954|P51857|O95167|P08574|P47985|Q13011|P06576|P48735
GO:0060047	heart contraction	2.75E-12	5.64E-11	9	39	178	27688	O15273|Q14896|P10916|P19429|P08590|P09493|P68032|P00441|P63316
GO:0060048	cardiac muscle contraction	7.24E-10	1.31E-08	7	30	178	27688	O15273|Q14896|P19429|P08590|P09493|P68032|P63316
GO:0060415	muscle tissue morphogenesis	5.31E-14	1.39E-12	11	55	178	27688	O15273|Q14896|P10916|P19429|P08590|P09493|P68032|P45379|P13533|P12883|P63316
GO:0060537	muscle tissue development	1.70E-09	2.96E-08	14	261	178	27688	O15273|Q14896|P10916|P68133|P68032|P13533|P12883|P19429|Q14192|P60660|P08590|P09493|P45379|P63316
GO:0060548	negative regulation of cell death	5.75E-04	4.52E-03	14	784	178	27688	P0CG47|P01033|P16989|P12235|P0CG48|P13693|Q05639|Q14192|P62987|P62979|P02511|P04792|P25116|P00441
GO:0061024	membrane organization	2.41E-03	1.37E-02	9	444	178	27688	P0CG47|P02792|P62987|P37108|P62979|Q08431|P19105|P00441|P0CG48
GO:0061061	muscle structure development	3.86E-08	5.91E-07	16	449	178	27688	O15273|Q14896|P10916|P19022|P68133|P68032|P13533|P12883|P19429|Q14192|P60660|P09382|P08590|P09493|P45379|P63316
GO:0065003	macromolecular complex assembly	6.70E-04	5.15E-03	17	1086	178	27688	O00217|O15273|P08708|P68363|O43920|P84243|P68871|P62263|P83731|P22352|Q16698|P02511|P04275|P69905|P62851|P62753|P24752
GO:0065008	regulation of biological quality	2.65E-04	2.27E-03	36	3074	178	27688	P07602|P02792|P51684|P01160|P16615|P35613|P12235|P39019|P13693|P16860|P19429|P84243|P68871|P09493|P62158|P62753|P00441|P01033|P02144|Q14116|Q08493|P08708|P04406|P51857|P13533|P19105|P12883|P09382|P08590|P62263|P04275|P63313|P17081|P25116|P06576|P63316
GO:0070252	actin-mediated cell contraction	2.46E-21	1.19E-19	14	40	178	27688	O15273|Q14896|P10916|P68133|P17661|P68032|P13533|P12883|P19429|P60660|P08590|P09493|P45379|P63316
GO:0070423	nucleotide-binding oligomerization domain containing signaling pathway	2.58E-05	2.84E-04	4	27	178	27688	P0CG47|P62987|P62979|P0CG48
GO:0070887	cellular response to chemical stimulus	2.74E-03	1.52E-02	22	1795	178	27688	P0CG47|P51452|Q14116|P51684|O14880|P80297|P13533|P17535|P0CG48|Q14192|P09382|P09493|P17174|P62987|P62979|Q13772|P62158|P17081|P04792|P62753|Q01628|P00441
GO:0070925	organelle assembly	8.42E-07	1.15E-05	11	254	178	27688	O15273|P10916|P08708|P09493|P62263|P68133|P83731|P68032|P62851|P13533|P62753
GO:0071156	regulation of cell cycle arrest	5.27E-03	2.54E-02	4	108	178	27688	P0CG47|P62987|P62979|P0CG48
GO:0071158	positive regulation of cell cycle arrest	1.79E-03	1.10E-02	4	80	178	27688	P0CG47|P62987|P62979|P0CG48
GO:0071704	organic substance metabolic process	3.42E-09	5.60E-08	107	10632	178	27688	O75947|O43674|P16615|O96000|P16860|P07195|P46783|P83731|P62913|P62753|P24752|O15239|Q13765|O95169|P13639|P48047|P04406|O14561|Q9Y5N6|O95167|P84098|P63173|P68104|P46777|P60866|P46776|P60228|P62249|Q13011|Q8N335|P07602|P25398|P68363|O95178|P62891|P25705|P18859|Q14192|Q16654|P56381|P00441|P54368|O00217|Q14116|Q08493|P08708|O75116|P18077|Q8N8D1|P42677|P41222|P15880|P17540|Q02543|Q9Y3D2|P62263|Q14240|P62945|P62269|P0CG47|P06732|Q9NSD9|P01160|P40926|P40925|P62280|P36542|P35613|P0CG48|P39019|P05090|P00505|P39023|P62273|P62158|Q07020|O14880|P19021|O43920|Q05639|P61353|P05386|P05387|P13929|O00483|P42766|Q02878|P60033|Q9NPI5|P17174|P61247|P62857|P22352|Q16698|P62979|P62851|P51452|P51970|P62906|P51857|P62987|P04275|P04792|P25116|P20585|P06576|P48735

<table>
<tr><td>GO:0071822</td><td>protein complex subunit organization</td><td>5.81E-26</td><td>3.95E-24</td><td>50</td><td>1259</td><td>178</td><td>27688</td><td>P62280|P39019|P39023|P62273|P46783|P83731|P62913|P69905|P62753|P24752|Q07020|O43920|P68032|P84098|P63173|P61353|P46777|P02511|P05386|P60866|P46776|P05387|P62249|P25398|P68363|P62891|P42766|Q02878|P84243|P68871|P61247|P62857|P22352|Q16698|P62979|P62851|O00217|O15273|P08708|P62906|P68133|P18077|P42677|P15880|Q02543|P62263|P62987|P62945|P04275|P62269</td></tr>
<tr><td>GO:0071826</td><td>ribonucleoprotein complex subunit organization</td><td>1.31E-03</td><td>8.70E-03</td><td>5</td><td>125</td><td>178</td><td>27688</td><td>P08708|P62263|P83731|P62851|P62753</td></tr>
<tr><td>GO:0071840</td><td>cellular component organization or biogenesis</td><td>1.43E-13</td><td>3.53E-12</td><td>72</td><td>4736</td><td>178</td><td>27688</td><td>P0CG47|P10916|P62280|P0CG48|P39019|P39023|P62273|P09493|P46783|P83731|P62913|P69905|P62753|P24752|P01034|Q07020|P19022|P37108|O43920|P68032|Q08431|P19105|P84098|P63173|P61353|P60660|P46777|P02511|P05386|P17081|P60866|P46776|P05387|P62249|P02792|P25398|P68363|P12235|P62891|P42766|Q02878|P84243|P68871|P61247|P62857|P22352|Q16698|P62979|P62851|P00441|O00217|Q14315|O15273|Q9P0U1|P08708|O75116|P62906|P68133|P18077|P17661|P13533|P42677|P15880|Q02543|P62263|P62987|P62945|P04275|P63313|P62269|P25116|P20585</td></tr>
<tr><td>GO:0071900</td><td>regulation of protein serine/threonine kinase</td><td>1.26E-02</td><td>4.91E-02</td><td>8</td><td>479</td><td>178</td><td>27688</td><td>P0CG47|P51452|P60033|P62987|P62979|P25116|P00441|P0CG48</td></tr>
<tr><td>GO:0071902</td><td>positive regulation of protein serine/threonine</td><td>5.71E-03</td><td>2.73E-02</td><td>6</td><td>250</td><td>178</td><td>27688</td><td>P0CG47|P60033|P62987|P62979|P00441|P0CG48</td></tr>
<tr><td>GO:0072330</td><td>monocarboxylic acid biosynthetic process</td><td>1.04E-02</td><td>4.27E-02</td><td>5</td><td>204</td><td>178</td><td>27688</td><td>P41222|O14561|O14880|P51857|P24752</td></tr>
<tr><td>GO:0072331</td><td>signal transduction by p53 class mediator</td><td>4.15E-03</td><td>2.11E-02</td><td>4</td><td>101</td><td>178</td><td>27688</td><td>P0CG47|P62987|P62979|P0CG48</td></tr>
<tr><td>GO:0072350</td><td>tricarboxylic acid metabolic process</td><td>1.04E-02</td><td>4.27E-02</td><td>3</td><td>70</td><td>178</td><td>27688</td><td>P40926|P40925|P48735</td></tr>
<tr><td>GO:0072358</td><td>cardiovascular system development</td><td>8.45E-08</td><td>1.25E-06</td><td>21</td><td>822</td><td>178</td><td>27688</td><td>P02144|Q14116|O15273|Q14896|P10916|P19021|P19022|P68032|P13533|Q08431|P12883|P16860|O14958|P19429|Q14192|P08590|P09493|P04792|P45379|P06576|P63316</td></tr>
<tr><td>GO:0072359</td><td>circulatory system development</td><td>8.45E-08</td><td>1.25E-06</td><td>21</td><td>822</td><td>178</td><td>27688</td><td>P02144|Q14116|O15273|Q14896|P10916|P19021|P19022|P68032|P13533|Q08431|P12883|P16860|O14958|P19429|Q14192|P08590|P09493|P04792|P45379|P06576|P63316</td></tr>
<tr><td>GO:0072395</td><td>signal transduction involved in cell cycle</td><td>9.76E-04</td><td>6.90E-03</td><td>4</td><td>68</td><td>178</td><td>27688</td><td>P0CG47|P62987|P62979|P0CG48</td></tr>
<tr><td>GO:0072401</td><td>signal transduction involved in DNA integrity</td><td>9.23E-04</td><td>6.59E-03</td><td>4</td><td>67</td><td>178</td><td>27688</td><td>P0CG47|P62987|P62979|P0CG48</td></tr>
<tr><td>GO:0072413</td><td>signal transduction involved in mitotic cell cycle</td><td>9.23E-04</td><td>6.59E-03</td><td>4</td><td>67</td><td>178</td><td>27688</td><td>P0CG47|P62987|P62979|P0CG48</td></tr>
<tr><td>GO:0072422</td><td>signal transduction involved in DNA damage</td><td>9.23E-04</td><td>6.59E-03</td><td>4</td><td>67</td><td>178</td><td>27688</td><td>P0CG47|P62987|P62979|P0CG48</td></tr>
<tr><td>GO:0072431</td><td>signal transduction involved in mitotic G1 DNA</td><td>8.72E-04</td><td>6.35E-03</td><td>4</td><td>66</td><td>178</td><td>27688</td><td>P0CG47|P62987|P62979|P0CG48</td></tr>
<tr><td>GO:0072521</td><td>purine-containing compound metabolic process</td><td>6.75E-14</td><td>1.73E-12</td><td>25</td><td>581</td><td>178</td><td>27688</td><td>P13929|O75947|P01160|O43674|O00483|P16615|P36542|O95178|O96000|P16860|P25705|P18859|P07195|P56381|O15239|O00217|Q08493|O95169|P51970|P48047|P04406|O14561|O43920|O95167|P06576</td></tr>
<tr><td>GO:0072522</td><td>purine-containing compound biosynthetic process</td><td>7.41E-06</td><td>9.01E-05</td><td>10</td><td>259</td><td>178</td><td>27688</td><td>O75947|P16860|P25705|P48047|P18859|P01160|P16615|P36542|P56381|P06576</td></tr>
<tr><td>GO:0072524</td><td>pyridine-containing compound metabolic process</td><td>2.01E-03</td><td>1.19E-02</td><td>6</td><td>202</td><td>178</td><td>27688</td><td>P13929|P40926|P04406|Q9NPI5|P07195|P40925</td></tr>
<tr><td>GO:0072593</td><td>reactive oxygen species metabolic process</td><td>1.17E-03</td><td>7.98E-03</td><td>5</td><td>122</td><td>178</td><td>27688</td><td>P68871|P22352|P69905|P62158|P00441</td></tr>
<tr><td>GO:0090068</td><td>positive regulation of cell cycle process</td><td>3.43E-03</td><td>1.82E-02</td><td>5</td><td>156</td><td>178</td><td>27688</td><td>P0CG47|O75116|P62987|P62979|P0CG48</td></tr>
<tr><td>GO:0090257</td><td>regulation of muscle system process</td><td>1.00E-07</td><td>1.45E-06</td><td>9</td><td>122</td><td>178</td><td>27688</td><td>Q14896|P10916|P19429|P08590|P09493|P25116|O14558|P00441|P63316</td></tr>
<tr><td>GO:0090304</td><td>nucleic acid metabolic process</td><td>3.01E-10</td><td>5.66E-09</td><td>47</td><td>2757</td><td>178</td><td>27688</td><td>P0CG47|P25398|Q9NSD9|P62280|P0CG48|P39019|P62891|P42766|Q02878|P39023|P62273|P46783|P61247|P83731|P62857|P62913|P62979|P62851|P62753|P00441|Q13765|Q07020|P08708|P62906|Q9Y5N6|P18077|Q8N8D1|P42677|P84098|P15880|P63173|Q02543|P61353|P62263|P62987|Q14240|P62945|P46777|P05386|P04792|P60866|P46776|P62269|P05387|P60228|P20585|P62249</td></tr>
<tr><td>GO:0090407</td><td>organophosphate biosynthetic process</td><td>6.93E-05</td><td>6.98E-04</td><td>13</td><td>557</td><td>178</td><td>27688</td><td>O75947|P06732|P48047|P01160|P16615|P36542|P16860|P25705|P18859|P60033|Q9NPI5|P56381|P06576</td></tr>
<tr><td>GO:0098655</td><td>cation transmembrane transport</td><td>1.49E-03</td><td>9.57E-03</td><td>7</td><td>259</td><td>178</td><td>27688</td><td>O75947|P25705|P48047|P18859|P36542|P56381|P06576</td></tr>
<tr><td>GO:0098660</td><td>inorganic ion transmembrane transport</td><td>1.42E-03</td><td>9.32E-03</td><td>7</td><td>257</td><td>178</td><td>27688</td><td>O75947|P25705|P48047|P18859|P36542|P56381|P06576</td></tr>
<tr><td>GO:0098662</td><td>inorganic cation transmembrane transport</td><td>1.42E-03</td><td>9.32E-03</td><td>7</td><td>257</td><td>178</td><td>27688</td><td>O75947|P25705|P48047|P18859|P36542|P56381|P06576</td></tr>
<tr><td>GO:0098763</td><td>mitotic cell cycle phase</td><td>7.00E-03</td><td>3.06E-02</td><td>6</td><td>261</td><td>178</td><td>27688</td><td>P0CG47|P42677|P62987|Q9Y5N6|P62979|P0CG48</td></tr>
<tr><td>GO:1901135</td><td>carbohydrate derivative metabolic process</td><td>9.17E-08</td><td>1.35E-06</td><td>27</td><td>1313</td><td>178</td><td>27688</td><td>P07602|P13929|O75947|P01160|O43674|O00483|P16615|P36542|O95178|O96000|P16860|P25705|P18859|P07195|P56381|O15239|O00217|Q08493|O95169|P51970|P48047|P04406|O14561|O43920|O95167|Q8N335|P06576</td></tr>
<tr><td>GO:1901184</td><td>regulation of ERBB signaling pathway</td><td>1.48E-03</td><td>9.54E-03</td><td>4</td><td>76</td><td>178</td><td>27688</td><td>P0CG47|P62987|P62979|P0CG48</td></tr>
<tr><td>GO:1901185</td><td>negative regulation of ERBB signaling pathway</td><td>1.53E-04</td><td>1.40E-03</td><td>4</td><td>42</td><td>178</td><td>27688</td><td>P0CG47|P62987|P62979|P0CG48</td></tr>
<tr><td>GO:1901293</td><td>nucleoside phosphate biosynthetic process</td><td>2.20E-05</td><td>2.46E-04</td><td>11</td><td>358</td><td>178</td><td>27688</td><td>O75947|P16860|P25705|P48047|P18859|P01160|Q9NPI5|P16615|P36542|P56381|P06576</td></tr>
<tr><td>GO:1901360</td><td>organic cyclic compound metabolic process</td><td>1.51E-18</td><td>5.96E-17</td><td>76</td><td>4234</td><td>178</td><td>27688</td><td>P0CG47|O75947|Q9NSD9|P01160|O43674|P40926|P40925|P16615|P62280|P36542|P0CG48|P39019|O96000|P16860|P39023|P62273|P07195|P46783|P83731|P62913|P62753|O15239|Q13765|Q07020|O95169|P48047|P04406|O14561|Q9Y5N6|O43920|O95167|P84098|P63173|P61353|P46777|P05386|P60866|P46776|P05387|P60228|P62249|P13929|P25398|O00483|O95178|P62891|P42766|Q02878|P25705|P18859|Q9NPI5|P61247|P62857|P62979|P56381|P62851|P00441|O00217|Q08493|P51970|P08708|P62906|P18077|P51857|Q8N8D1|P42677|P15880|Q02543|P62263|P62987|Q14240|P62945|P04792|P62269|P20585|P06576</td></tr>
<tr><td>GO:1901362</td><td>organic cyclic compound biosynthetic process</td><td>2.44E-23</td><td>1.40E-21</td><td>49</td><td>1377</td><td>178</td><td>27688</td><td>O75947|P01160|P16615|P62280|P36542|P39019|P16860|P39023|P62273|P46783|P83731|P62913|P62753|Q13765|Q07020|P48047|P84098|P63173|P61353|P46777|P05386|P60866|P46776|P05387|P62249|P25398|P62891|P42766|Q02878|P25705|P18859|Q9NPI5|P61247|P62857|P62979|P56381|P62851|P08708|P62906|P18077|P51857|P42677|P15880|Q02543|P62263|P62987|P62945|P62269|P06576</td></tr>
<tr><td>GO:1901564</td><td>organonitrogen compound metabolic process</td><td>4.07E-38</td><td>6.81E-36</td><td>81</td><td>2460</td><td>178</td><td>27688</td><td>O75947|P06732|Q9NSD9|P01160|O43674|P40926|P40925|P16615|P62280|P36542|P39019|O96000|P16860|P00505|P39023|P62273|P07195|P46783|P83731|P62913|P62753|P24752|O15239|Q13765|Q07020|O95169|P13639|P48047|P04406|O14561|P19021|O43920|O95167|P84098|Q05639|P63173|P68104|P61353|P46777|P05386|P60866|P46776|P05387|P60228|P62249|P07602|P13929|P25398|O00483|O95178|P62891|P42766|Q02878|P25705|P18859|Q9NPI5|P17174|P61247|P62857|P22352|P62979|P56381|P62851|P00441|P54368|O00217|Q08493|P51970|P08708|P62906|P18077|P42677|P15880|P17540|Q02543|P62263|P62987|Q14240|P62945|P62269|P06576</td></tr>
<tr><td>GO:1901566</td><td>organonitrogen compound biosynthetic process</td><td>4.65E-31</td><td>4.60E-29</td><td>58</td><td>1418</td><td>178</td><td>27688</td><td>O75947|P06732|Q9NSD9|P01160|P16615|P62280|P36542|P39019|P16860|P00505|P39023|P62273|P46783|P83731|P62913|P62753|Q13765|Q07020|P13639|P48047|P84098|Q05639|P63173|P68104|P61353|P46777|P05386|P60866|P46776|P05387|P60228|P62249|P25398|P62891|P42766|Q02878|P25705|P18859|Q9NPI5|P17174|P61247|P62857|P62979|P56381|P62851|P54368|P08708|P62906|P18077|P42677|P15880|Q02543|P62263|P62987|Q14240|P62945|P62269|P06576</td></tr>
<tr><td>GO:1901575</td><td>organic substance catabolic process</td><td>6.40E-03</td><td>2.83E-02</td><td>19</td><td>1578</td><td>178</td><td>27688</td><td>P0CG47|P13929|Q08493|P04406|P51857|P0CG48|P00505|P07195|P17174|P62987|Q14240|Q16698|P62979|P62158|P60228|P24752|Q13011|Q8N335|P00441</td></tr>
<tr><td>GO:1901576</td><td>organic substance biosynthetic process</td><td>9.42E-20</td><td>3.94E-18</td><td>69</td><td>3354</td><td>178</td><td>27688</td><td>O75947|P06732|Q9NSD9|P01160|P40926|P40925|P16615|P62280|P36542|P39019|P16860|P00505|P39023|P62273|P46783|P83731|P62913|P62753|P24752|Q13765|Q07020|P13639|P48047|P04406|O14561|O14880|Q9Y5N6|P84098|Q05639|P63173|P68104|P61353|P46777|P05386|P60866|P46776|P05387|P60228|P62249|P13929|P25398|P62891|P42766|Q02878|P25705|P18859|P60033|Q9NPI5|P17174|P61247|P62857|P62979|P56381|P62851|P54368|P08708|P62906|P18077|P51857|P42677|P41222|P15880|Q02543|P62263|P62987|Q14240|P62945|P62269|P06576</td></tr>
<tr><td>GO:1901652</td><td>response to peptide</td><td>8.37E-03</td><td>3.57E-02</td><td>9</td><td>539</td><td>178</td><td>27688</td><td>P01033|Q14116|P16860|P01160|P17174|P08574|P17081|P62753|P35613</td></tr>
<tr><td>GO:1901657</td><td>glycosyl compound metabolic process</td><td>3.10E-12</td><td>6.30E-11</td><td>22</td><td>518</td><td>178</td><td>27688</td><td>O00217|P13929|O75947|O95169|P51970|P48047|O43674|P04406|O14561|O00483|P16615|O43920|P36542|O95167|O95178|O96000|P25705|P18859|P07195|P56381|P06576|O15239</td></tr>
<tr><td>GO:1901659</td><td>glycosyl compound biosynthetic process</td><td>7.74E-05</td><td>7.69E-04</td><td>8</td><td>214</td><td>178</td><td>27688</td><td>O75947|P25705|P48047|P18859|P16615|P36542|P56381|P06576</td></tr>
</table>

GO:1901700	response to oxygen-containing compound	1.64E-04	1.49E-03	21	1348	178	27688	P01033|P02144|Q14116|P01160|P19021|P68032|P35613|P12883|P16860|P00505|P09382|P68871|P09493|P17174|P08574|P22352|P69905|P17081|P62753|P25116|P00441
GO:1901988	negative regulation of cell cycle phase transition	8.29E-03	3.54E-02	4	123	178	27688	P0CG47|P62987|P62979|P0CG48
GO:1901991	negative regulation of mitotic cell cycle phase	7.61E-03	3.29E-02	4	120	178	27688	P0CG47|P62987|P62979|P0CG48
GO:1902400	intracellular signal transduction involved in G1	8.72E-04	6.35E-03	4	66	178	27688	P0CG47|P62987|P62979|P0CG48
GO:1902402	signal transduction involved in mitotic DNA	9.23E-04	6.59E-03	4	67	178	27688	P0CG47|P62987|P62979|P0CG48
GO:1902403	signal transduction involved in mitotic DNA	9.23E-04	6.59E-03	4	67	178	27688	P0CG47|P62987|P62979|P0CG48
GO:1902578	single-organism localization	1.20E-02	4.81E-02	36	3840	178	27688	P07602|P0CG47|O75947|P02792|P68363|P16615|P36542|P35613|P12235|P0CG48|P39019|P13693|P16860|P05090|P25705|P00505|P18859|P68871|P62913|P62979|P56381|P69905|P62158|P00441|P01033|Q9P0U1|P48047|Q9Y277|P37108|Q08431|P19105|P62987|P04275|P63313|P25116|P06576
GO:1902582	single-organism intracellular transport	1.83E-05	2.05E-04	19	975	178	27688	P0CG47|O75947|P02792|Q9P0U1|P48047|P68363|P37108|P16615|P36542|P19105|P0CG48|P25705|P18859|P62987|P62913|P62979|P56381|P25116|P06576
GO:1902600	hydrogen ion transmembrane transport	8.78E-06	1.06E-04	7	113	178	27688	O75947|P25705|P48047|P18859|P36542|P56381|P06576
GO:1902806	regulation of cell cycle G1/S phase transition	3.09E-03	1.66E-02	4	93	178	27688	P0CG47|P62987|P62979|P0CG48
GO:1902807	negative regulation of cell cycle G1/S phase	1.63E-03	1.02E-02	4	78	178	27688	P0CG47|P62987|P62979|P0CG48
GO:1903050	regulation of proteolysis involved in cellular	2.82E-03	1.56E-02	5	149	178	27688	P0CG47|P63244|P62987|P62979|P0CG48
GO:1903052	positive regulation of proteolysis involved in cellular protein catabolic process	7.99E-04	6.02E-03	5	112	178	27688	P0CG47|P63244|P62987|P62979|P0CG48
GO:1903321	negative regulation of protein modification by small protein conjugation or removal	4.30E-03	2.17E-02	4	102	178	27688	P0CG47|P62987|P62979|P0CG48
GO:1903322	positive regulation of protein modification by small protein conjugation or removal	9.50E-03	3.98E-02	4	128	178	27688	P0CG47|P62987|P62979|P0CG48
GO:1903362	regulation of cellular protein catabolic process	3.25E-03	1.74E-02	5	154	178	27688	P0CG47|P63244|P62987|P62979|P0CG48
GO:1903364	positive regulation of cellular protein catabolic	8.32E-04	6.23E-03	5	113	178	27688	P0CG47|P63244|P62987|P62979|P0CG48
GO:1903522	regulation of blood circulation	4.52E-06	5.62E-05	9	192	178	27688	P16860|Q9UBY9|P08590|P09493|P17661|P45379|P13533|P25116|P12883
GO:1990267	response to transition metal nanoparticle	9.25E-03	3.89E-02	4	127	178	27688	P80297|P19021|P35613|P00441
GO:1990542	mitochondrial transmembrane transport	6.28E-09	1.01E-07	7	40	178	27688	O75947|P25705|P48047|P18859|P36542|P56381|P06576
GO:2000045	regulation of G1/S transition of mitotic cell cycle	3.09E-03	1.66E-02	4	93	178	27688	P0CG47|P62987|P62979|P0CG48
GO:2000058	regulation of protein ubiquitination involved in ubiquitin-dependent protein catabolic process	1.79E-03	1.10E-02	4	80	178	27688	P0CG47|P62987|P62979|P0CG48
GO:2000060	positive regulation of protein ubiquitination involved in ubiquitin-dependent protein catabolic	1.48E-03	9.54E-03	4	76	178	27688	P0CG47|P62987|P62979|P0CG48
GO:2000134	negative regulation of G1/S transition of mitotic	1.63E-03	1.02E-02	4	78	178	27688	P0CG47|P62987|P62979|P0CG48

Supplemental Table S5D. 2011 v 2016 comparison. A selection of *Biological Process* GO terms extracted from two BinGO analyses, using either the 2016 or the 2011 GO annotation dataset and adult ventricular cardiomyocyte data[19] (2016-hA-VCMs and 2011-hA-VCMs, respectively). GO terms selected either align with the GO terms identified by Poon et al., 2013, or a descriptive GO term associated with a large proportion of the analysed dataset, or are terms from the '*cardiac conduction*' domain of the ontology. **n** indicates the number of protein IDs associated with the GO term, **corr p-value** is the p-value after Benjamini & Hochberg False Discovery Rate correction, **x** is the number of IDs in both the submitted list and associated with the GO term, #N/A indicates that this term was not significantly enriched in the dataset. The corrected p-values in the 2016 analysis are highlighted in green if the p-value is more significant in the 2016 analysis, and orange if the p-value is more significant in the 2011 analysis.

Description	2016-hA-VCMs n	2016-hA-VCMs corr p-value	2016-hA-VCMs x	2011-hA-VCMs n	2011-hA-VCMs corr p-value	2011-hA-VCMs x
GO:0006614 SRP-dependent cotranslational protein targeting to membrane	123	1.98E-52	37		#N/A	#N/A
GO:0045047 protein targeting to ER	127	4.39E-52	37		#N/A	#N/A
GO:0072599 establishment of protein localization to endoplasmic reticulum	131	1.36E-51	37		#N/A	#N/A
GO:0006414 translational elongation	223	2.52E-47	40	110	1.40E-53	38
GO:0006414 translational elongation	223	1.06E-34	43	110	1.40E-53	38
GO:0006091 generation of precursor metabolites and energy	574	1.06E-34	43	552	1.89E-28	40
GO:0022904 respiratory electron transport chain	157	1.67E-34	29	135	5.48E-34	29
GO:0022411 cellular component disassembly	673	2.76E-34	45	221	1.73E-39	37
GO:0045333 cellular respiration	231	5.04E-34	32	206	6.99E-33	32
GO:0044419 interspecies interaction between organisms	830	1.26E-31	46	781	5.40E-27	44
GO:0061024 membrane organization	1211	1.16E-27	49	444	1.37E-02	9
GO:0003012 muscle system process	319	7.11E-27	30	233	1.38E-21	25
GO:0003012 muscle system process	319	7.11E-27	30	233	1.38E-21	25
GO:0016071 mRNA metabolic process	743	7.53E-27	40	651	6.93E-27	41
GO:0006936 muscle contraction	264	4.09E-25	27	205	1.36E-21	24
GO:0033275 actin-myosin filament sliding	39	3.13E-21	14	38	5.24E-20	14
GO:0070252 actin-mediated cell contraction	76	2.08E-18	15	40	1.19E-19	14
GO:0051234 establishment of localization	7073	2.56E-18	93	5116	1.50E-03	53
GO:0006810 transport	6916	3.12E-17	90	5023	1.00E-03	53
GO:0042775 mitochondrial ATP synthesis coupled electron transport	77	1.12E-16	14	68	1.45E-11	11
GO:0006941 striated muscle contraction	101	1.74E-16	15	61	1.28E-10	10
GO:0060047 heart contraction	80	1.96E-16	14	39	5.64E-11	9
GO:0060048 cardiac muscle contraction	69	1.03E-15	13	30	1.31E-08	7
GO:0032774 RNA biosynthetic process	3310	7.53E-15	57	669	3.45E-22	37
GO:0006937 regulation of muscle contraction	179	4.80E-14	16	98	2.31E-07	9
GO:0010467 gene expression	5483	1.09E-13	73	2206	4.58E-11	45
GO:0090257 regulation of muscle system process	230	1.48E-13	17	122	1.45E-06	9
GO:0061061 muscle structure development	502	6.98E-13	22	449	5.91E-07	16
GO:1903522 regulation of blood circulation	326	3.31E-12	18	192	5.62E-05	9
GO:0009725 response to hormone	1187	3.76E-12	31	1086	2.69E-05	22
GO:0007005 mitochondrion organization	679	4.56E-12	24		#N/A	#N/A
GO:0008016 regulation of heart contraction	243	5.04E-12	16	116	1.22E-04	7
GO:0055001 muscle cell development	153	3.42E-11	13	98	4.34E-05	7
GO:0048738 cardiac muscle tissue development	164	8.07E-11	13	138	1.99E-09	12
GO:0008015 blood circulation	401	9.50E-11	18	319	4.44E-08	15
GO:0042692 muscle cell differentiation	290	7.63E-10	15	245	3.41E-04	9
GO:0002027 regulation of heart rate	90	7.84E-10	10	39	1.19E-02	3
GO:0051146 striated muscle cell differentiation	211	1.74E-09	13	152	6.09E-04	7
GO:0044267 cellular protein metabolic process	6461	4.75E-08	68	3447	4.00E-09	54
GO:0009888 tissue development	1677	6.35E-08	30	1526	6.26E-03	21
GO:0032502 developmental process	6093	1.82E-07	64	5680	4.09E-12	80
GO:0030029 actin filament-based process	823	2.13E-07	20	505	5.02E-07	17
GO:0072358 cardiovascular system development	915	2.40E-07	21	822	1.25E-06	21
GO:0006950 response to stress	4615	2.77E-07	53	3628	5.91E-03	39
GO:0007507 heart development	537	3.74E-07	16	489	1.78E-06	16
GO:0009653 anatomical structure morphogenesis	2626	4.21E-07	37	2300	1.82E-02	26
GO:0034220 ion transmembrane transport	1636	4.90E-07	28	336	2.90E-03	9
GO:0009611 response to wounding	798	6.28E-07	19	835	7.37E-03	14
GO:0050896 response to stimulus	11437	6.60E-07	96		#N/A	#N/A
GO:0010941 regulation of cell death	1800	9.40E-07	29	1340	3.38E-03	20
GO:0006811 ion transport	2414	1.56E-06	34		#N/A	#N/A
GO:0002026 regulation of the force of heart contraction	31	5.93E-06	5		#N/A	#N/A
GO:0043434 response to peptide hormone	591	6.71E-06	15	527	3.18E-02	9
GO:0048468 cell development	1903	9.01E-06	28		#N/A	#N/A
GO:0030154 cell differentiation	3701	1.36E-05	42		#N/A	#N/A
GO:1903779 regulation of cardiac conduction	68	1.57E-05	6		#N/A	#N/A
GO:0048869 cellular developmental process	3946	6.01E-05	42		#N/A	#N/A
GO:0010882 regulation of cardiac muscle contraction by calcium ion signaling	24	6.19E-05	4		#N/A	#N/A
GO:0007596 blood coagulation	541	2.54E-04	12	518	1.19E-02	10
GO:0006366 transcription from RNA polymerase II promoter	833	2.90E-04	15		#N/A	#N/A
GO:0010881 regulation of cardiac muscle contraction by regulation of the release of sequestered calcium ion	20	9.48E-04	3		#N/A	#N/A
GO:0007010 cytoskeleton organization	1479	1.88E-03	19		#N/A	#N/A
GO:1902533 positive regulation of intracellular signal transduction	1054	2.67E-03	15		#N/A	#N/A
GO:0033554 cellular response to stress	2165	1.02E-02	22		#N/A	#N/A
GO:0061337 cardiac conduction	126	1.71E-02	4		#N/A	#N/A
GO:0012501 programmed cell death	1204	1.83E-02	14		#N/A	#N/A
GO:0045893 positive regulation of transcription, DNA-templated	1575	3.05E-02	16		#N/A	#N/A

Supplemental References

1. Day-Richter J, Harris MA, Haendel M, Gene Ontology OBO-Edit Working Group, Lewis S. OBO-Edit--an ontology editor for biologists. Bioinformatics. 2007;23:2198-200.
2. Gene Ontology Consortium. The Gene Ontology in 2010: extensions and refinements. Nucleic Acids Res. 2009;38:D331-5.
3. Mungall CJ, Bada M, Berardini TZ, Deegan J, Ireland A, Harris MA, et al. Cross-product extensions of the Gene Ontology. J Biomed Inform. 2011;44:80-6.
4. Huntley RP, Harris MA, Alam-Faruque Y, Blake JA, Carbon S, Dietze H, et al. A method for increasing expressivity of Gene Ontology annotations using a compositional approach. BMC Bioinformatics. 2014;15:155.
5. Huntley RP, Sitnikov D, Orlic-Milacic M, Balakrishnan R, D'Eustachio P, Gillespie ME, et al. Guidelines for the functional annotation of microRNAs using the Gene Ontology. RNA. 2016;22:667-76.
6. Dowell KG, McAndrews-Hill MS, Hill DP, Drabkin HJ, Blake JA. Integrating text mining into the MGI biocuration workflow. Database (Oxford). 2009;2009:bap019.
7. Patel S, Roncaglia P, Lovering RC. Using Gene Ontology to describe the role of the neurexin-neuroligin-SHANK complex in human, mouse and rat and its relevance to autism. BMC Bioinformatics. 2015;16:186.
8. Bard J, Rhee SY, Ashburner M. An ontology for cell types. Genome Biol. 2005;6:R21.
9. Meehan TF, Masci AM, Abdulla A, Cowell LG, Blake JA, Mungall CJ, et al. Logical development of the cell ontology. BMC Bioinformatics. 2011;12:6.
10. Diehl AD, Meehan TF, Bradford YM, Brush MH, Dahdul WM, Dougall DS, et al. The Cell Ontology 2016: enhanced content, modularization, and ontology interoperability. J Biomed Semantics. 2016;7:44.
11. Mungall CJ, Torniai C, Gkoutos GV, Lewis SE, Haendel MA. Uberon, an integrative multi-species anatomy ontology. Genome Biol. 2012;13:R5.
12. Haendel MA, Balhoff JP, Bastian FB, Blackburn DC, Blake JA, Bradford Y, et al. Unification of multi-species vertebrate anatomy ontologies for comparative biology in Uberon. J Biomed Semantics. 2014;5:21.
13. Binns D, Dimmer E, Huntley R, Barrell D, O'Donovan C, Apweiler R. QuickGO: a web-based tool for Gene Ontology searching. Bioinformatics. 2009;25:3045-6.
14. Gene Ontology Consortium. Gene Ontology Consortium: going forward. Nucleic Acids Res. 2015;43:D1049-56.
15. Balakrishnan R, Harris MA, Huntley R, Van Auken K, Cherry JM. A guide to best practices for Gene Ontology (GO) manual annotation. Database (Oxford). 2013;2013:bat054.
16. Vilella AJ, Severin J, Ureta-Vidal A, Heng L, Durbin R, Birney E. EnsemblCompara GeneTrees: Complete, duplication-aware phylogenetic trees in vertebrates. Genome research. 2009;19:327-35.
17. Eyre TA, Wright MW, Lush MJ, Bruford EA. HCOP: a searchable database of human orthology predictions. Briefings in bioinformatics. 2007;8:2-5.
18. Coordinators NR. Database Resources of the National Center for Biotechnology Information. Nucleic Acids Res. 2017;45:D12-D17.
19. Poon E, Yan B, Zhang S, Rushing S, Keung W, Ren L, et al. Transcriptome-guided functional analyses reveal novel biological properties and regulatory hierarchy of human embryonic stem cell-derived ventricular cardiomyocytes crucial for maturation. PLoS One. 2013;8:e77784.
20. Maere S, Heymans K, Kuiper M. BiNGO: a Cytoscape plugin to assess overrepresentation of gene ontology categories in biological networks. Bioinformatics. 2005;21:3448-9.
21. Smoot ME, Ono K, Ruscheinski J, Wang PL, Ideker T. Cytoscape 2.8: new features for data integration and network visualization. Bioinformatics. 2011;27:431-2.

22. De Giusti VC, Nolly MB, Yeves AM, Caldiz CI, Villa-Abrille MC, Chiappe de Cingolani GE, et al. Aldosterone stimulates the cardiac Na(+)/H(+) exchanger via transactivation of the epidermal growth factor receptor. Hypertension. 2011;58:912-9.
23. Arnolds DE, Chu A, McNally EM, Nobrega MA, Moskowitz IP. The emerging genetic landscape underlying cardiac conduction system function. Birth Defects Res A Clin Mol Teratol. 2011;91:578-85.
24. Campuzano O, Brugada R. Genetics of familial atrial fibrillation. Europace. 2009;11:1267-71.
25. Abriel H. Cardiac sodium channel Na(v)1.5 and interacting proteins: Physiology and pathophysiology. J Mol Cell Cardiol. 2010;48:2-11.
26. Donoso P, Sanchez G, Bull R, Hidalgo C. Modulation of cardiac ryanodine receptor activity by ROS and RNS. Front Biosci (Landmark Ed). 2011;16:553-67.
27. Dulhunty AF, Hewawasam R, Liu D, Casarotto MG, Board PG. Regulation of the cardiac muscle ryanodine receptor by glutathione transferases. Drug Metab Rev. 2011;43:236-52.
28. Latronico MV, Condorelli G. MicroRNAs and cardiac conduction. Curr Drug Targets. 2010;11:907-12.
29. Kim GH. MicroRNA regulation of cardiac conduction and arrhythmias. Transl Res. 2013;161:381-92.
30. Poudel P, Xu Y, Cui Z, Sharma D, Tian B, Paudel S. Atrial fibrillation: recent advances in understanding the role of microRNAs in atrial remodeling with an electrophysiological overview. Cardiology. 2015;131:58-67.
31. Willis BC, Ponce-Balbuena D, Jalife J. Protein assemblies of sodium and inward rectifier potassium channels control cardiac excitability and arrhythmogenesis. Am J Physiol Heart Circ Physiol. 2015;308:H1463-73.
32. Campuzano O, Allegue C, Fernandez A, Iglesias A, Brugada R. Determining the pathogenicity of genetic variants associated with cardiac channelopathies. Sci Rep. 2015;5:7953.
33. Arking DE, Pulit SL, Crotti L, van der Harst P, Munroe PB, Koopmann TT, et al. Genetic association study of QT interval highlights role for calcium signaling pathways in myocardial repolarization. Nat Genet. 2014;46:826-36.
34. Spears DA, Gollob MH. Genetics of inherited primary arrhythmia disorders. Appl Clin Genet. 2015;8:215-33.
35. Tucker NR, Ellinor PT. Emerging directions in the genetics of atrial fibrillation. Circ Res. 2014;114:1469-82.
36. Tucker NR, Clauss S, Ellinor PT. Common variation in atrial fibrillation: navigating the path from genetic association to mechanism. Cardiovasc Res. 2016;109:493-501.
